# Assessing and enhancing migration of human myogenic progenitors using directed iPS cell differentiation and advanced tissue modelling

**DOI:** 10.15252/emmm.202114526

**Published:** 2022-09-26

**Authors:** SungWoo Choi, Giulia Ferrari, Louise A Moyle, Kirsty Mackinlay, Naira Naouar, Salma Jalal, Sara Benedetti, Christine Wells, Francesco Muntoni, Francesco Saverio Tedesco

**Affiliations:** ^1^ The Francis Crick Institute London UK; ^2^ Department of Cell and Developmental Biology University College London London UK; ^3^ Institut de Biologie Paris Seine FR3631, Plateforme de Bioinformatique ARTbio Sorbonne Université Paris France; ^4^ UCL Great Ormond Street Institute of Child Health University College London London UK; ^5^ National Institute for Health Research Great Ormond Street Hospital Biomedical Research Centre London UK; ^6^ Centre for Stem Cell Systems The University of Melbourne Melbourne VIC Australia; ^7^ Dubowitz Neuromuscular Centre UCL Great Ormond Street Institute of Child Health & Great Ormond Street Hospital for Children London UK; ^8^ Present address: Institute of Biomedical Engineering University of Toronto Toronto ON Canada; ^9^ Present address: Department of Physiology, Development and Neuroscience University of Cambridge Cambridge UK

**Keywords:** cell migration, cell therapy, iPS cells, muscular dystrophy, tissue engineering, Musculoskeletal System, Stem Cells & Regenerative Medicine

## Abstract

Muscle satellite stem cells (MuSCs) are responsible for skeletal muscle growth and regeneration. Despite their differentiation potential, human MuSCs have limited *in vitro* expansion and *in vivo* migration capacity, limiting their use in cell therapies for diseases affecting multiple skeletal muscles. Several protocols have been developed to derive MuSC‐like progenitors from human induced pluripotent stem (iPS) cells (hiPSCs) to establish a source of myogenic cells with controllable proliferation and differentiation. However, current hiPSC myogenic derivatives also suffer from limitations of cell migration, ultimately delaying their clinical translation. Here we use a multi‐disciplinary approach including bioinformatics and tissue engineering to show that DLL4 and PDGF‐BB improve migration of hiPSC‐derived myogenic progenitors. Transcriptomic analyses demonstrate that this property is conserved across species and multiple hiPSC lines, consistent with results from single cell motility profiling. Treated cells showed enhanced trans‐endothelial migration in transwell assays. Finally, increased motility was detected in a novel humanised assay to study cell migration using 3D artificial muscles, harnessing advanced tissue modelling to move hiPSCs closer to future muscle gene and cell therapies.

## Graphical abstract

This study describes an advanced framework to assess, model and enhance migration of human skeletal myogenic progenitor cells using platforms including induced pluripotent stem (iPS) cells, RNAseq, single cell profiling and tissue engineering.

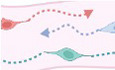

**The paper explained**

**Problem**

Cell therapies to treat severe muscular dystrophies remain inefficient to date. Major hurdles to clinical efficacy of such therapies include the limited ability to expand mature myogenic cells *in vitro*, as well as the minimal migration capacity of myogenic cells upon transplantation which reduces their ability to disperse into the affected tissues where they are needed.

**Results**

We used a developmentally‐inspired treatment (via DLL4 and PDGF‐BB) activating the NOTCH and PDGF pathways to impart higher migratory capacity to human myogenic cells derived from induced pluripotent stem cells (iPSCs; a source providing a potentially limitless supply of cells). We showed the efficacy of this treatment using a range of advanced and emerging technologies such as directed iPSC differentiation, single‐cell profiling, microfluidics and 3D tissue engineering.

**Impact**

In this work, we developed tools to assess and a strategy to induce and enhance migration of iPSC‐derived myogenic cells, with translational relevance for both intramuscular and intra‐vascular cell delivery. Enhancing extravasation is a key milestone to develop future efficacious muscle cell therapies. Moreover, the technological platforms we have used for the validation of this treatment can be applied further to wider drug and therapy screening purposes.

## Introduction

Muscle satellite stem cells (MuSCs) reside between the basal lamina and sarcolemma of muscle fibres and are responsible for growth and regeneration of skeletal myofibres. Upon activation, MuSCs give rise to an activated progeny named myoblasts, which then repair and regenerate myofibres (reviewed in Benedetti *et al*, 2013). Myoblasts have been tested in numerous clinical trials for Duchenne muscular dystrophy (DMD), the most common muscular dystrophy of childhood, which severely affects most skeletal muscles and remains incurable (reviewed in Tedesco *et al*, 2010). However, despite promising pre‐clinical results in animal models, to date, only myogenic cell therapies of localised muscular dystrophies such as oculopharyngeal muscular dystrophy (OPMD) have reported functional improvements upon myoblast transplantations in patients (Périé *et al*, 2014).

Skeletal myogenic cells have been delivered via the intramuscular or the intravascular route (Tedesco *et al*, 2010). However, the efficacy of both transplantation modalities is impeded by insufficient migration, leading to poor muscle biodistribution of donor cells. Intramuscular injections frequently result in generation of chimeric myofibres often limited to areas adjacent to the needle trajectory, necessitating multiple injections and making this strategy challenging for generalised myopathies such as DMD (Skuk, 2004). On the other hand, intravascular delivery of donor cells via major arteries may facilitate simultaneous targeting of multiple muscle groups. Intra‐arterial injections of mesoangioblasts, myogenic cells derived from a subset of muscle perivascular cells, ameliorated muscle pathology and function in pre‐clinical models of muscular dystrophy and was also translated into a phase I/IIa clinical trial in five DMD boys (Cossu *et al*, 2016). Although mesoangioblasts are still considered promising candidate cells for systemic delivery owing to their trans‐endothelial migration (also known as extravasation) capacity, they possess lower skeletal myogenic and self‐renewal capacity than MuSCs, which limits their long‐term translational potential. Therefore, an ideal cell type for myogenic cell therapies should possess the migratory capacity of perivascular cells as well as the differentiation and self‐renewing potential of MuSCs.

NOTCH signalling plays a pivotal role in cell fate specification during embryonic myogenesis, as well as in post‐natal MuSC self‐renewal and differentiation (Conboy & Rando, 2002; Schuster‐Gossler *et al*, 2007; Bjornson *et al*, 2012; Mourikis & Tajbakhsh, 2014; Baghdadi *et al*, 2018; Verma *et al*, 2018). Canonical NOTCH signalling involves interactions between NOTCH ligands (e.g., Delta‐like (DLL) 1, 3, 4 and Jagged (JAG) 1, 2) and receptors (NOTCH 1–4). Perturbation of NOTCH signalling in donor cells has shown context‐dependent effects on myogenic cell transplants. Treatment of mouse and human myoblasts with DLL1 and DLL4 did not enhance engraftment in *mdx* mice, a DMD mouse model (Sakai *et al*, 2017). However, DLL1 treatment of canine MuSCs maintained their engraftment potential during *in vitro* expansion (Parker *et al*, 2012). Furthermore, modulation of the DLL1‐NOTCH1 axis in both mouse and human mesoangioblasts supported improvement of the dystrophic phenotype after intra‐arterial delivery in mice (Quattrocelli *et al*, 2014). Platelet‐derived growth factor (PDGF) signalling is another regulator of myogenic cell behaviour. PDGF receptor‐β (PDGFR‐β) is expressed by cells derived from the mesenchyme (Dellavalle *et al*, 2007; Trojanowska, 2009). PDGF‐BB, the putative ligand of PDGFR‐β, is expressed by endothelial cells and dystrophic muscle fibres for recruitment of pericytes and MuSCs, respectively (Betsholtz, 2004; Piñol‐Jurado *et al*, 2017).

Previous work showed that mouse embryonic myoblasts in close proximity to blood vessels undergo a spontaneous fate shift into pericyte‐like cells *in vivo*; this phenomenon was mimicked *in vitro* by treating embryonic myoblasts with DLL4 and PDGF‐BB (Cappellari *et al*, 2013). More recently, we showed that modulation of NOTCH and PDGF pathways induces perivascular cell features while enhancing self‐renewal and migration in adult mouse and human MuSC‐derived myoblasts (Gerli *et al*, 2019). However, the translational potential of primary, tissue‐derived MuSCs is hindered by the need to obtain them invasively (i.e. via muscle biopsies), as well as by their limited expansion capacity and premature differentiation *in vitro*, which pose major hurdles to reach the cell number required to treat patients with disorders involving multiple muscles such as DMD (Cossu *et al*, 2016). Induced pluripotent stem cells (iPSCs) offer a solution to bypass these limitations.

Human iPSCs (hiPSCs) are becoming a key source of skeletal myogenic progenitor cells for disease modelling and transplantation studies, owing to their controllable proliferation and differentiation capacity, lack of significant ethical concerns and non‐invasive sampling of the starting primary cell population (Loperfido *et al*, 2015). Several protocols are currently available to generate skeletal myogenic derivatives from hiPSCs (reviewed in Selvaraj *et al*, 2019a). Starting from the pioneering studies based upon controlled expression of myogenic regulators to obtain transplantable skeletal myogenic cells from hiPSCs (e.g., Darabi *et al*, 2012; Goudenege *et al*, 2012; Tedesco *et al*, 2012), the field has refined transgene‐based protocols to direct hiPSC differentiation into skeletal muscle (e.g., Albini *et al*, 2013; Maffioletti *et al*, 2015; Shoji *et al*, 2016; Selvaraj *et al*, 2019b), whilst also developing genomic‐integration‐free, small molecule‐based methods to derive myogenic cells mimicking embryonic development (e.g., Borchin *et al*, 2013; Caron *et al*, 2016; Chal *et al*, 2016; Hicks *et al*, 2018). However, the focus on perfecting methods to obtain myogenic progenitors resembling self‐renewing MuSCs has neglected the critical need to enhance their migration capacity, which is essential to deliver cells to large or multiple muscle districts. Although some attempts have previously been made to deliver iPSC‐derived myogenic cells systemically (Tedesco *et al*, 2012; Matthias *et al*, 2015; Incitti *et al*, 2019), no specific methods are currently available to differentiate hiPSCs into myogenic progenitors with enhanced migratory and/or extravasation capacity.

**Figure 1:**
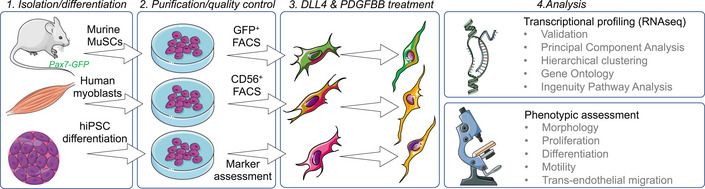
Schematic representation of the cell isolation, culture, treatment, differentiation and analysis pipeline underpinning this study Figure created using Servier Medical Art (https://smart.servier.com) in accordance with a Creative Commons Attribution 3.0 Unported Licence (https://creativecommons.org/licenses/by/3.0/).

Here we exploited directed hiPSC‐differentiation, bioinformatics and advanced tissue modelling (Jalal *et al*, 2021) to engineer a developmentally‐inspired, small‐molecule‐based, genomic‐integration‐free strategy to increase motility and trans‐endothelial migration of human myogenic progenitor cells via modulation of NOTCH and PDGF signalling. This study (summarised in Fig 1) provides a framework to model, test and enhance migration of human myogenic cells for future cell therapies of muscle diseases.

## Results

### Combined activation of NOTCH and PDGF signalling pathways induces conserved transcriptional changes in mouse and human tissue‐ and iPSC‐derived myogenic progenitors

**Figure 2:**
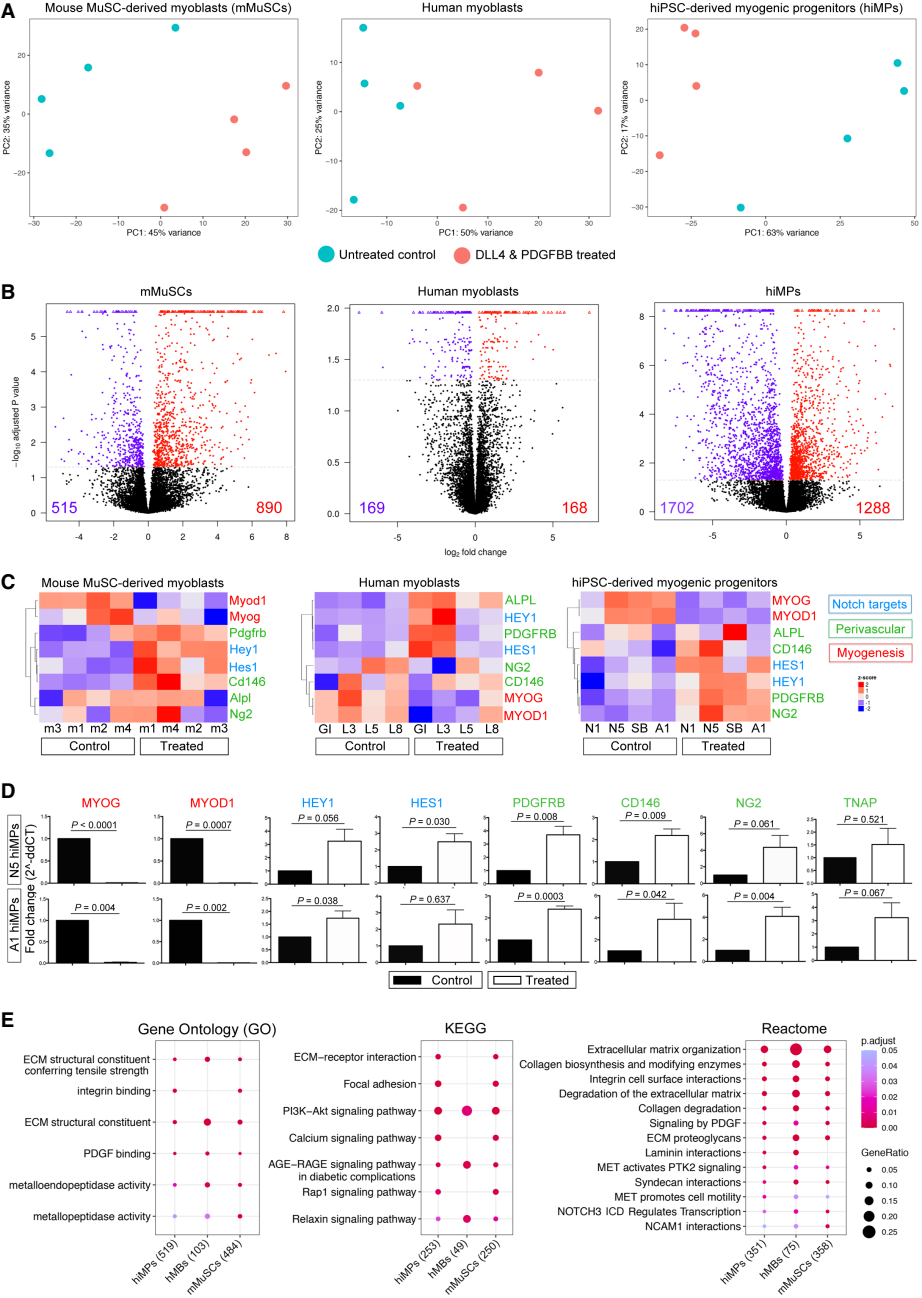
RNAseq‐based transcriptional profiling of mouse and human myogenic progenitors upon activation of NOTCH and PDGF signalling pathways Principal Component Analysis (PCA) showing mMuSC‐derived myoblasts (left), human myoblasts (centre) and hiMPs (right). Four cell lines were analysed by RNAseq in treated and untreated conditions for each cell population. Each point on the PCA represents a cell population. Additional information in Appendix Tables S1 and S2.Volcano plots showing differentially expressed genes between untreated and DLL4 and PDGFBB‐treated mMuSCs, human myoblasts and hiMPs. Red dots represent genes which display a positive fold‐change in expression upon treatment with DLL4 and PDGF‐BB whilst violet dots represent genes which are significantly downregulated. Differentially expressed genes required a *P* value of ≤ 0.05 to be considered significant.Heatmaps showing changes in expression of key myogenic (*MYOGENIN, MYOD1*), perivascular (*PDGFRB*, *CD146*, *NG2*, *ALPL*) and NOTCH target (*HEY1*, *HES1*) genes upon treatment with DLL4 and PDGF‐BB in mMuSC‐derived myoblasts (left), human myoblasts (middle) and hiMPs (right). Clustering was performed by genes/probes with Pearson correlation. Colour scale based on z‐scores: red regions indicate high expression whilst blue regions indicate low expression. Dendrograms indicate the similarity of clusters as well as the orders in which clusters were assembled.Validation of RNAseq data of panel (C) by real‐time PCR analysis of the same myogenic, perivascular and NOTCH target transcripts in treated and untreated hiMPs (experimental replicates = 3; error bars; SEM). Statistical analysis (paired *t*‐test) was performed on ΔCt values whilst graphs show fold change relative to untreated controls.Curated dot plot Gene Ontology (GO; left), Kyoto Encyclopaedia of Genes and Genomes (KEGG; centre) and Reactome (right) enrichment analyses showing shared gene functions amongst the cell groups; numbers in brackets: genes analysed with a *P* value threshold set at 0.05; full lists in a dedicated spreadsheet available in Dataset EV1. Source data are available online for this figure.

**Figure EV1:**
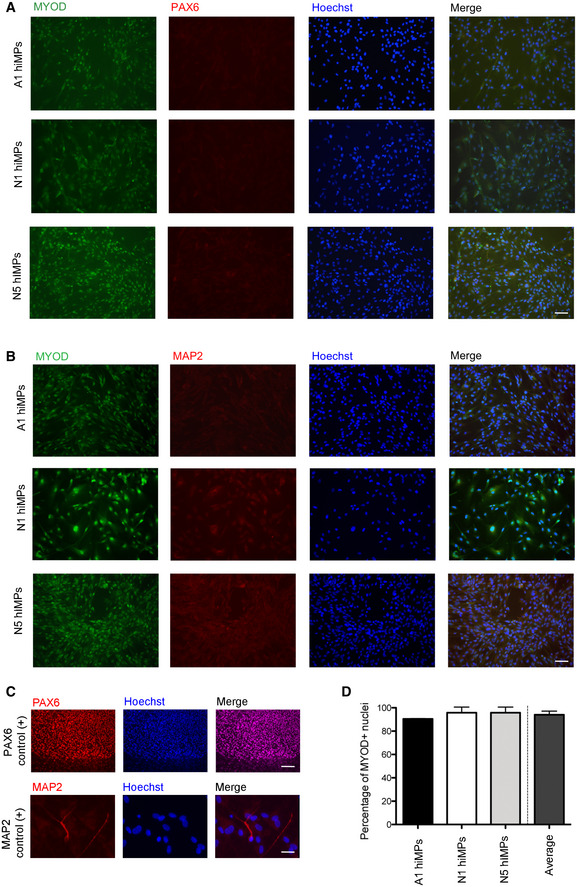
Assessment of purity of hiMP populations Representative immunofluorescence analysis of MYOD (skeletal myogenic lineage marker, green) and PAX6 (early neuroectodermal lineage marker, red) immunoreactivity in three of the four different hiMP lines used in this study.Immunofluorescence analysis of MYOD and MAP2 (late neuroectodermal/neuronal marker, red) in the same hiMPs shown in (A).Positive controls for the PAX6 and MAP2 staining shown in (A, B); top panel: spontaneously differentiating hiPSCs; bottom panel: hiPSC‐derived neurons.Bar graph quantifying the percentages of MYOD‐positive nuclei within three populations of hiMPs (experimental replicates = 3; error bars: SD). Data information: Scale bars: (A, B) 75 μm; (C) top 100 μm; bottom 20 μm. Source data are available online for this figure.

We aimed to identify targetable pathways to improve migration of human iPSC‐derived myogenic progenitors (hiMPs), and focused on NOTCH and PDGF (Hellström *et al*, 1999; Armulik *et al*, 2011) which have been shown to improve migration in tissue‐resident MuSCs (Gerli *et al*, 2019). To identify whether hiMPs respond to activation of the aforementioned pathways, we performed an unbiased assessment of global transcriptomic changes induced by DLL4 and PDGF‐BB in wild‐type mouse and human primary MuSC‐derived myoblasts, alongside hiMPs. Before performing bulk RNA‐sequencing (RNAseq) of these cell populations, we first assessed their purity. Four distinct mouse and four distinct human MuSC‐derived myoblast populations were isolated and FACS‐purified from skeletal muscles of Pax7‐nGFP mice and from healthy human muscle biopsies using green fluorescent protein (GFP) and CD56 (NCAM), respectively (Materials and Methods). hiMPs were derived from four distinct, fully‐characterised hiPSC lines generated with genomic‐integration‐free technologies using a validated transgene‐free, small molecule‐based protocol recapitulating skeletal muscle developmental specification and differentiation *in vitro* (Caron *et al*, 2016; Materials and Methods). Purity of the hiPSC derivatives was assessed by immunostaining for myogenic and other non‐myogenic markers. hiMPs were homogeneously positive for the skeletal myogenic determination factor MYOD and lacked contamination from neuroectodermal derivatives (PAX6 and MAP2; Fig EV1). After assessing their purity, the three groups of cells were treated for 7 days with DLL4 and PDGF‐BB (Materials and Methods) and then mRNA was extracted from treated and untreated samples for RNAseq. Principal component analysis (PCA) revealed distinct segregation between DLL4 and PDGF‐BB‐treated and untreated populations of the 3 cell types (Fig 2A, and Appendix Tables S1 and S2). Additionally, RNAseq analysis provided a total of 1,405, 337 and 2,990 differentially expressed genes between treated mouse MuSC‐derived myoblasts, human myoblasts and hiMPs and their untreated controls respectively (Fig 2B). Hierarchical clustering of top 50 differentially regulated genes in mouse and human samples showed overall consistency of transcriptional dynamics in those transcripts across all four lines analysed (Appendix Fig S1 and Table S2).

We then tested whether the observed global transcriptional changes were a consequence of NOTCH and PDGF signalling activation. To address this question, we looked at specific downstream targets of NOTCH and PDGF pathways, as well as key myogenic and perivascular markers known to be modulated by this treatment in murine myoblasts (Cappellari *et al*, 2013; Gerli *et al*, 2019). As shown in Fig 2C, treated mouse MuSCs (mMuSCs), human myoblasts and hiMPs shared similar dynamics of NOTCH targets and perivascular transcripts upregulation, coupled with downregulation of myogenic transcripts such as *Myogenin* and *MyoD* (also a downstream NOTCH signalling target (Kopan *et al*, 1994)). This was further validated via qRT‐PCR analysis in two representative hiMP lines (Fig 2D). We subsequently wanted to identify inter‐species similarities in transcriptional response to DLL4 and PDGF‐BB treatment. For this purpose, we selected the top 50 differentially regulated genes of treated mMuSC‐derived myoblasts, found the relative human orthologues and then performed hierarchical clustering on human myoblast and hiMP datasets. The resulting heatmaps show that the majority of transcripts in the treated human cells display a similar regulation in comparison to their murine counterparts (Appendix Fig S2 and Table S3), further indicating an overall conservation of the cellular response to DLL4 and PDGF‐BB in skeletal myogenic cells (albeit with some expected variability in human, non‐syngeneic cells). Finally, Gene Ontology (GO), Kegg and Reactome enrichment analyses showed shared gene functions amongst the cell groups, including pathways involved in extracellular matrix remodelling, integrin‐cell surface interactions, focal adhesion generation, in addition to the expected NOTCH and PDGF pathways (Fig 2E).Together these data demonstrate that DLL4 and PDGF‐BB induce transcriptional changes across skeletal myogenic progenitors from different species and developmental origins, with hiMPs showing the greatest transcriptional response.

### Analysis of morphology, proliferation and differentiation of DLL4 and PDGFBB‐treated hiMPS

**Figure 3:**
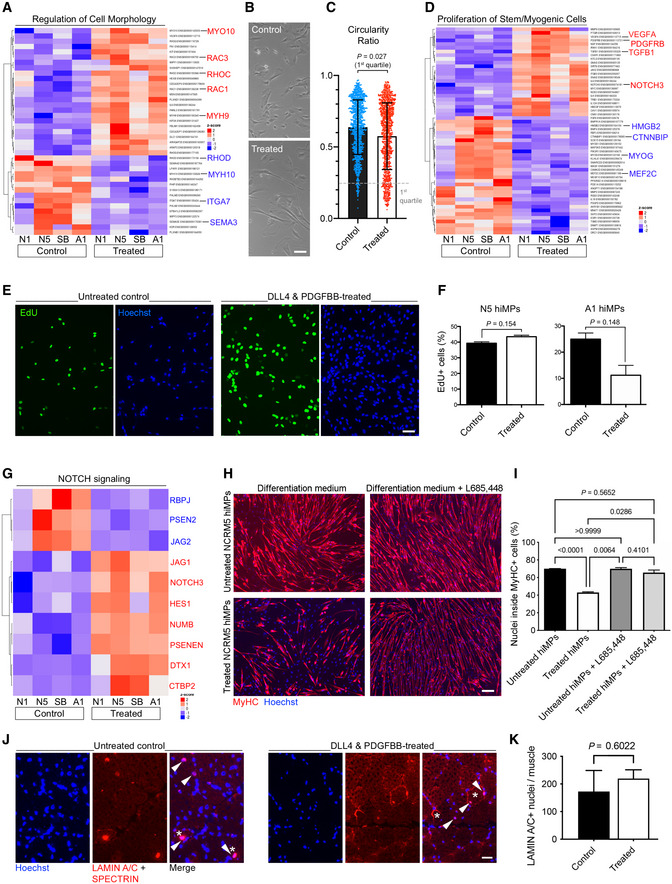
Analysis of morphology, proliferation and differentiation of DLL4 and PDGFBB‐treated hiMPs *P* value‐adjusted hierarchical clustering heatmap generated from a gene ontology list of genes involved in regulation of cell morphology (GO 0008360; *P* set at 0.05).Phase contrast images displaying morphology of untreated and treated hiMPs. Scale bar: 25 μm.Scatter plot showing morphometric analysis of treated and untreated hiMPs. Morphology was quantified using the circularity ratio, where 1 = perfect circle and 0 = line (experimental replicates = 3). Statistical analysis (paired *t*‐test) was performed on the first quartile to enhance detection of morphological changes (error bars: SD).
*P* value‐adjusted hierarchical clustering heatmap generated from a gene ontology list of genes involved in proliferation of stem and myogenic cell types (GO 2000291; 0048660; 0014857; 0072091; *P* set at 0.05).Immunofluorescence images of untreated and treated hiMPs incubated with EdU for 2 h. Scale bar: 75 μm.Bar graphs quantifying EdU experiment shown in (E) (experimental replicates = 3; error bars: SEM). Statistical analysis based on an unpaired *t*‐test.
*P* value‐adjusted hierarchical clustering heatmap of NOTCH signalling genes (Kegg pathway 04330).Immunofluorescence images of hiMPs expanded in control or treated conditions for 1 week, induced to differentiate for 4 days in the presence or absence of γ‐secretase inhibitor L685458 and immunostained for myosin heavy chain (MyHC). Scale bar: 75 μm.Bar graph quantifying the average percentage of nuclei within MyHC positive myotubes (experimental replicates = 3; error bars: SEM). Statistical significance based on one‐way ANOVA with Tukey's multiple comparison. Scale bar: 75 μm.Immunofluorescence panels showing human specific LAMIN A/C+ (nuclei) and SPECTRIN (sarcolemma) staining in tibialis anterior muscles of immunodeficient mice (*N* = 3) transplanted with treated (*n* = 3 muscles) and untreated (*n* = 3 muscles) N5 hiMPs.Quantification of LAMIN A/C+ grafted human cells across each muscle. Data information: full gene list for heatmaps in (A) and (D) available in Appendix Table S4. Source data are available online for this figure.

To identify whether the transcriptional response of DLL4 and PDGFBB‐treated hiMPs results in detectable, cellular phenotypic changes, we assessed specific transcriptional signatures alongside functional readouts such as morphology, proliferation and skeletal myogenic differentiation capacity. Hierarchical clustering analysis highlighted modulation of several regulators of cell morphology such as upregulation of *MYH9*, *MYO10*, *RAC1/3* and *RHOC*, alongside downregulation of *RHOD*, *MYH10*, *ITGA7* and *SEMA3* (Fig 3A; Appendix Table S4). After 1 week of treatment, hiMPs appeared more elongated than their untreated counterpart, in accordance with what was observed in mMuSCs (Gerli *et al*, 2019). Morphometric analysis confirmed this finding, revealing a higher number of cells falling within the first quartile (0–0.25) of cell circularity ratio (i.e., cells with marked protrusions; Fig 3B and C; mean ± SD: treated 45.33 ± 10.26, untreated 12.67 ± 10.60; *P* = 0.027, paired *t*‐test). We next assessed the impact of DLL4 and PDGF‐BB treatment on hiMP proliferation. A decrease in the proliferative capacity of myogenic cells could be detrimental for cell therapy, limiting the translational potential of donor cells. Hierarchical clustering analysis highlighted modulation of several regulators of cell proliferation and lineage commitment in at least 3 out of 4 hiMP lines, including upregulation of *PDGFRB*, *NOTCH3*, *VEGFA* and *TGFB1*, alongside downregulation of *CTNNBIP1*, *HMGB2* and the myogenic factors *MEF2C* and *MYOG* (Fig 3D). These transcriptional changes did not impact on the proliferative ability of hiMPs, with treated and untreated cells displaying a comparable cell cycle, as shown by functional EdU incorporation assay (Fig 3E and F; Appendix Table S4).

NOTCH activation inhibits myogenesis *in vitro* in embryonic and adult myoblasts (Kopan *et al*, 1994; Conboy & Rando, 2002; Mourikis & Tajbakhsh, 2014; Gerli *et al*, 2019). Although RNAseq analysis of the NOTCH pathway shows modulation of several effectors (Fig 3G), we wanted to functionally verify the conservation of this phenomenon in hiMPs. To achieve this aim, we induced myogenic differentiation of DLL4 and PDGF‐BB‐treated cells and observed a significant reduction in the percentage of nuclei within MyHC‐positive fibres, from 70.00 ± 0.29 to 43.06 ± 1.20% (Fig 3H and I; *P* < 0.0001; *N* = 3; mean ± SD). A similar reduction in myogenic differentiation was observed when hiMPs were expanded in an alternative medium, albeit with lower pre‐treatment differentiation capacity upon long‐term expansion (details in Appendix Fig S3). To further validate the NOTCH‐dependency of this finding, we blocked NOTCH pathway with the γ‐secretase inhibitor L685458, which selectively inhibits γ‐secretase‐dependent nuclear translocation of the NOTCH Intra‐Cellular Domain (NICD). Upon treatment with L685458, the impairment of differentiation was reverted from 43.06 ± 1.20 to 65.59 ± 5.11 (*P* 0.028; Fig 3H and I), thus confirming that hiMP myogenic differentiation potential is NOTCH‐dependent and could be restored to pre‐treatment levels. Moreover, reversion of differentiation impairment was also noted to take place spontaneously upon removal of DLL4 and PDGF‐BB, with increasing myogenic differentiation noticeable from day 3 onwards of removal of the stimuli (Appendix Fig S4). Finally, treated and untreated cells were intramuscularly transplanted in regenerating muscles of immunodeficient mice (*N* = 3) and no statistically significant differences were noted between the two groups, indicating that DLL4 and PDGF‐BB treatment does not negatively impact on the myogenic capacity of cells upon transplantation (Fig 3J and K).

### Combined DLL4 and PDGF‐BB treatment enhances motility of hiMPs

**Figure 4:**
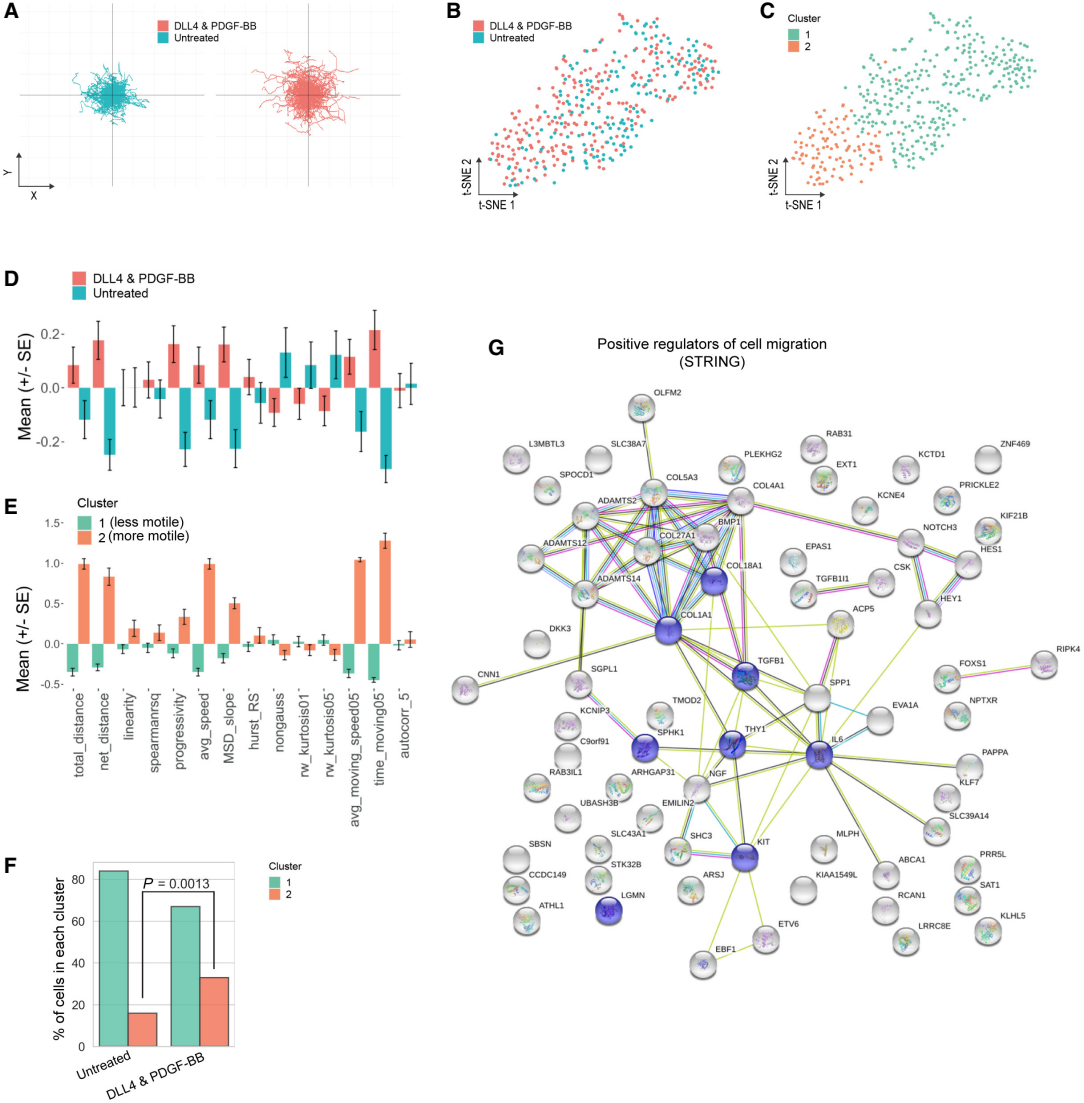
Analysis motile and migratory properties of DLL4 and PDGF‐BB treated hiMPs ATrajectory plots for visualisation of the migratory paths of treated and untreated cells over the duration of the motility assay. Each line represents the path of a single cell. DLL4 and PDGF‐BB treatment was completed prior to motility assessment and all cells were imaged on plastic dishes in absence of the two inducing factors.BVisualisation of the motility state space of untreated and DLL4 and PDGF‐BB‐treated hiMPs using t‐SNE plots (perplexity = 35).CUnsupervised hierarchical clustering (Ward's method) visualised with a t‐SNE plot showing two clusters (Silhouette S_*i*_ = 0.22).D, EBar charts demonstrating normalised values for comparison of motility features between conditions (D; untreated and DLL4 and PDGF‐BB) and clusters (E) (experimental replicates = 3; total 408 cells; error bars: SEM).FBar graph demonstrating proportions of control and DLL4 and PDGF‐BB‐treated cells within each cluster. Hypothesis testing was performed using the chi‐squared (χ^2^) test.GFunctional protein association network analysis (https://string‐db.org). The network view summarises predicted associations for proteins positively regulating cell migration common to all three datasets. The nodes are proteins and the edges represent the predicted functional associations. Red line: fusion evidence; Green line: neighbourhood evidence; Blue line: co‐occurrence evidence; Purple line: experimental evidence; Yellow line: text mining evidence; Light blue line: database evidence; Black line: co‐expression evidence. Blue nodes: GO:0030335 positive regulation of cell migration, Count in gene set: 8 of 452, false discovery rate: 0.0156. Source data are available online for this figure.

**Figure EV2:**
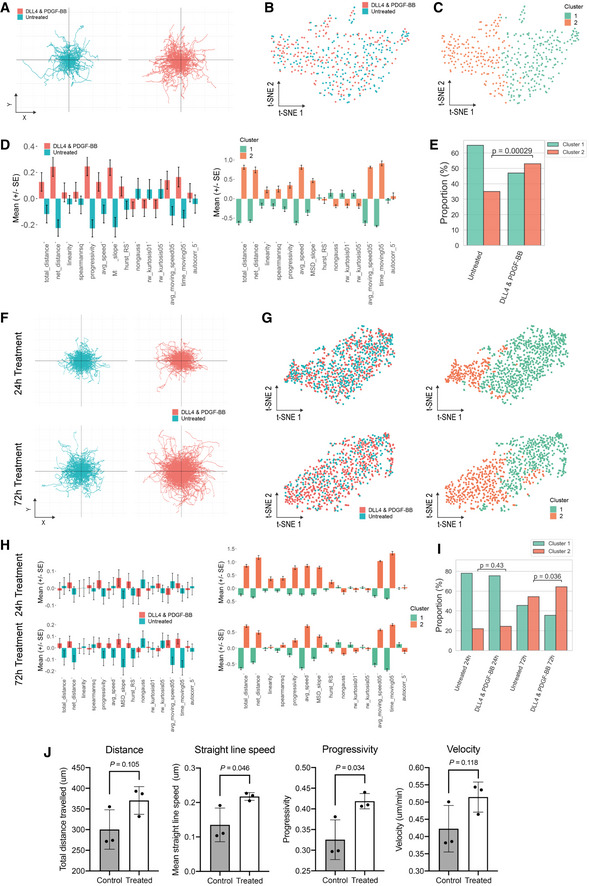
Additional *in vitro* motility and migration analyses of treated and untreated hiMPs ATrajectory plots for visualisation of the migratory paths of untreated and treated cells that were exposed to either 1% BSA or DLL4 and PDGF‐BB, respectively, over the course of the motility assay. Each line depicts the path of an individual cell.BVisualisation of the motility state space of untreated and treated hiMPs using t‐SNE (perplexity = 35).CHierarchical clustering of the first 30 principal components visualised with a t‐SNE plot showing two clusters (Silhouette S_*i*_ = 0.19).DBar charts displaying the normalised motility feature values for comparison between conditions: untreated and DLL4 and PDGF‐BB (left), cluster 1 and cluster 2 (right) (experimental replicates = 3; total 412 cells).EBar graph showing proportions of untreated and treated hiMPs within the two clusters. Hypothesis testing was performed with a chi‐squared (χ^2^) test.FTrajectory plots for visualisation of hiMP migration after 24 h of treatment (top row), or 72 h of treatment (bottom row).G, Ht‐SNE plots (perplexity = 35) for visualisation of the motility state space of hiMPs in two‐dimensions (left). Cluster assignments after hierarchical clustering (S_*i*_ = 0.13 (24 h); S_*i*_ = 0.18 (72 h)). (H) Bar plots showing normalised motility features for both 24 h (top row) and 72 h (bottom row) conditions (experimental replicates = 3; total 876 cells and total 478 cells analysed for 24 and 72 h conditions, respectively.).IBar graph displaying proportions of untreated and DLL4 and PDGF‐BB‐treated hiMPs treated for 24 and 72 h. Hypothesis testing was performed with a chi‐squared (χ^2^) test.JBar graphs depict quantification of parameters obtained from single cell tracking analysed using TrackMate. Motility statistics were calculated for untreated (grey bars) and treated (white bars) hiMPs (experimental replicates = 3; error bars: SD). *P* values within figure: *t*‐test. Source data are available online for this figure.

We and others have shown that NOTCH and PDGF pathways play a critical role in regulating developmental fate, regenerative potential and migration of primary, native myogenic cells (Betsholtz, 2004; Cappellari *et al*, 2013; Piñol‐Jurado *et al*, 2017; Camps *et al*, 2019; Gerli *et al*, 2019). Of the aforementioned properties, cell migration is of key relevance for cell therapy. To investigate whether DLL4 and PDGF‐BB had an effect on cell migration of hiMPs, we analysed the differentially expressed genes in our RNAseq dataset using Ingenuity Pathway Analysis (IPA). Amongst the most significantly modulated cellular functions upon DLL4 and PDGF‐BB treatment there was “Cellular Movement”, with a total of 578 differentially expressed genes (Table 1). To correlate these transcriptional changes to a phenotypic response, automated single cell tracking of cells exposed to DLL4 and PDGF‐BB was performed and track features were extracted using Heteromotility (Kimmel *et al*, 2018; Fig 4B–F; Materials and Methods). Motility assays were performed under conditions of continuous treatment, in which hiMPs were exposed to either 1% BSA (untreated) or DLL4 and PDGF‐BB treatment during the duration of the assay (Fig EV2), as well as in conditions of discontinued treatment, where hiMPs were plated on uncoated surfaces without addition of PDGF‐BB (Fig 4). For both conditions, single‐cell trajectories indicated an increase in motility mediated by the treatment (Figs 4A and EV2A). Visualisation of single‐cell motility features with t‐SNE plots revealed that both untreated and DLL4 and PDGF‐BB‐treated hiMPs shared the same motility state space (Figs 4B and EV2B). To identify heterogenous motility phenotypes, unsupervised hierarchical clustering (Ward's method) was performed with the first 30 principal components which captured > 95% variation to obtain two clusters (Fig 4C and EV2C). Cluster 1 was comprised of a less motile population of cells as indicated by lower total distance travelled, average speed and average time spent moving (Figs 4D and E and EV2D). Cluster 2 represents the motile population of cells, demonstrating higher distances travelled, average speeds and proportion of time spent moving. Additionally, cells within cluster 2 performed directed migration as shown by higher progressivity, linearity and mean squared displacement (MSD; Figs 4D and E, and EV2). In both conditions of treatment, a significant increase in the proportion of cells in cluster 2 was observed indicating that states of high motility are maintained for at least 24 h after the treatment was discontinued (Figs 4F and EV2E). Increased proportion of migratory cells were also detected after a shorter course of treatment of 72 h (Fig EV2F–I). Furthermore, analyses performed with Trackmate (Materials and Methods) validated these findings, showing increased trends in distance, straight line speed, progressivity and velocity in treated hiMPs (Fig EV2J).

**Table 1:** Top cellular and molecular functions associated with DLL4 and PDGF‐BB modulation generated via ingenuity pathway analysis (IPA).

IPA cellular and molecular function	*P*‐value range	Number of molecules
Cellular assembly and organisation	2.66E‐08–4.79E‐33	600
Cellular function and maintenance	3.69E‐08–4.79E‐33	710
Cellular movement	3.38E‐08–4.16E‐31	578
Cell death and survival	3.77E‐08–2.55E‐28	814
Cellular development	2.23E‐08–7.60E‐23	726

Genes upregulated in the DLL4 and PDGF‐BB‐treated hiMPs relative to the untreated control were subjected to IPA to reveal the predicted most significant associated functions.

To gain further insights on possible protein–protein interaction networks that might positively regulate cell migration, we analysed our RNAseq dataset with the STRING platform (https://string‐db.org; Szklarczyk *et al*, 2019; Fig 4G). Functional enrichment analysis highlighted that a number of candidate proteins with relevance in cell migration, which could be associated with the observed migratory phenotype, were upregulated in our datasets, such as TGFB1, ADAMTS2/12/14 and THY1 (Fig 4G; Barker *et al*, 2004; Sciorati *et al*, 2006; Li *et al*, 2020).

### Assessment of the effect of DLL4 and PDGF‐BB on trans‐endothelial migration of hiMPs

**Figure 5:**
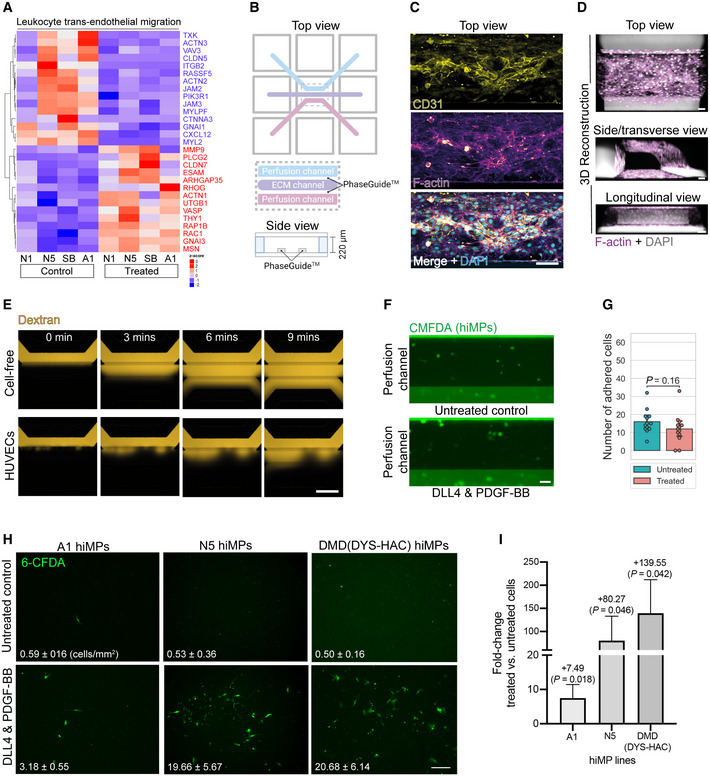
Modelling and assessing trans‐endothelial migration of hiMPs *P* value‐adjusted hierarchical clustering heatmap displaying hierarchical clustering of genes associated with leukocyte trans‐endothelial migration (KEGG pathway: hsa04670; *P* set at 0.05).Graphical representation of an individual chip of the OrganoPlate® (produced with BioRender, www.biorender.com). Each chip consists of a top perfusion channel, central ECM channel and bottom perfusion channel. Phase guides between channels allows for generation of surface tension after deposition of collagen‐I within the ECM channel so that there is no physical barrier between the collagen gel and perfusion channels. This facilitates generation of a 3D blood vessel that is in direct contact with the ECM channel.Maximum intensity projections of the top perfusion channel, 48 h after seeding HUVECs, immunostained for CD31 and F‐actin. Scale bar: 100 μm.3D projections of blood vessel‐like tubules of the top perfusion channel stained for F‐actin. Scale bar: 50 μm.Representative fluorescence images of 150 kDa TRITC‐conjugated dextran added to the top perfusion channel of OrganoPlate® chips with and without 3D endothelial monolayers generated by HUVECs. Chips were imaged every 3 min. See Appendix Fig EV3 for extended panel and quantification. Scale bar: 100 μm.Representative fluorescence images of CMFDA‐stained untreated and DLL4 and PDGF‐BB‐treated hiMPs within the top perfusion channel, 15 min after delivery and kept on the OrganoFlow®. Scale bar: 50 μm.Bar graph quantifying adhesion images in (E). Statistical significance was calculated based on a paired *t*‐test (experimental replicates = 3). Each point on the plot represents the number of adhered cells after 15 min within a single chip.Assessment of DLL4 and PDGF‐BB‐treated WT and genetically corrected DMD hiMP migration through a layer of endothelial cells. Representative images showing the lower side of the trans‐well membrane on which treated and untreated hiMPs (stained with the transient dye CFDA, in green) are simultaneously seeded on HUVECs for 8 h. Bar graphs quantifying the average number of CFDA‐positive cells/ mm^2^, that have migrated through the endothelial layer in each considered condition. (experimental replicates = 3). A minimum of 10 (1.5 mm^2^) fields per condition was quantified (error bars: SEM). Scale bar: 250 μm.Bar graph showing fold‐change in trans‐endothelial migration (error bars: SEM). Statistical significance based on one‐way ANOVA with Bonferroni's multiple comparison. Source data are available online for this figure.

**Figure EV3:**
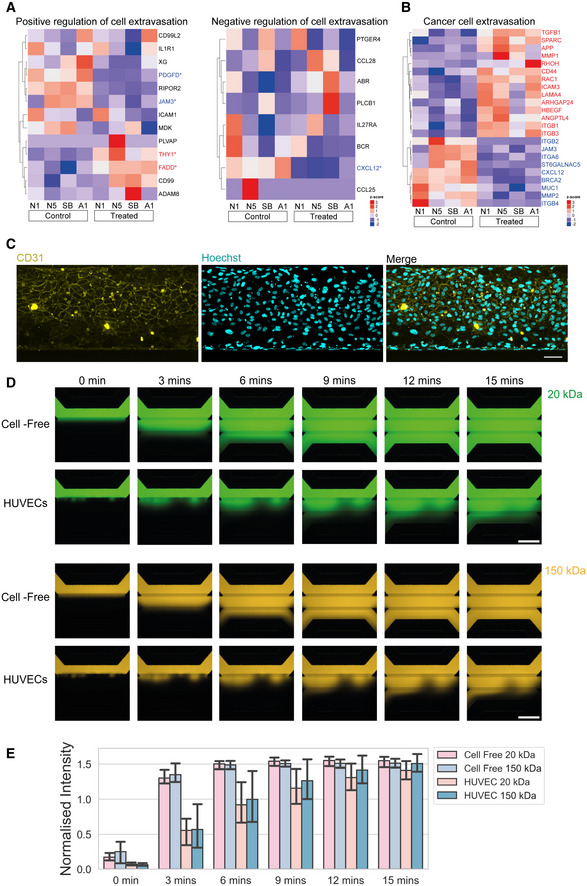
Additional *in silico* analyses and assessment of permeability of endothelialised 3D blood vessel‐like microfluidic channels AHeatmaps displaying genes that are involved in negative regulation of cellular extravasation (left; GO: 002692) and positive regulation of cellular extravasation (right; GO: 002693). *P < 0.05.B
*P* value‐adjusted hierarchical clustering heatmap showing a manually curated list of genes involved in enhanced trans‐endothelial migration of cancer cells (*P* set al 0.05).CMaximum intensity projection of a microfluidic channel immunostained for CD31 showing cobblestone‐like morphology of HUVECs lining the top perfusion channel. Scale bar = 30 μm.DRepresentative fluorescence images of 20 kDa FITC‐conjugated dextran (top two rows) and 150 kDa TRITC‐conjugated dextran (bottom two rows) added to the top perfusion channel of OrganoPlate^®^ chips with and without 3D endothelial monolayers generated by HUVECs. Chips were imaged every 3 min for 15 min. Scale bar = 100 μm.EBar chart quantifying images shown in (D) using the normalised intensity calculated as the ratio of fluorescence between the ECM channel and top perfusion channel at each time point for cell‐free and HUVEC chips containing 20 and 150 kDa fluorescent dextrans (technical replicates = 3; error bars; SD). Source data are available online for this figure.

Although encouraging, enhanced cellular motility may not be directly relevant in the context of cell therapies requiring intravascular cell delivery to target multiple large muscles. Therefore, we assessed the effect of DLL4 and PDGF‐BB treatment on trans‐endothelial migration which is essential for systemically delivered cells. Interestingly, RNAseq analysis showed positive modulation of several transcripts involved in cell adhesion and extravasation in treated hiMPs, such as *ESAM*, *ICAM3*, *JAM2*, *MMP9*, *PDGFD* and *THY1*, although some other mediators of extravasation such as *ITGB2* and *CXCL12* were downregulated (Figs 5A, and EV3A and B; Appendix Table S4). Trans‐endothelial migration is a multi‐step process which starts with cell adhesion to the endothelium under perfusion and then ends with diapedesis in target tissues. To recapitulate the complexity of this process and to functionally assess if DLL4 and PDGF‐BB have a role on hiMPs extravasation we first utilised an organ‐on‐chip system with artificial flow to assess cell–cell adhesion to endothelial cells. Each chip consists of three channels: a top perfusion channel, central extracellular matrix (ECM) channel and bottom perfusion channel (Fig 5B). Flow within channels is introduced using a rocker platform. Within the top perfusion channels, CD31^+^ 3D blood vessel‐like tubules were generated using human‐umbilical vein endothelial cells (HUVECs; Figs 5C and D, and EV3C). We first validated that the endothelial channels were functional using a barrier integrity assay, in which fluorescent dextrans of different molecular weights (20 and 150 kDa) were introduced in the top perfusion channel. Quantification of dextran diffusion into the ECM channel indicated that the layer of HUVECs reduced the channel's permeability to dextran molecules (Figs 5E and EV3D). To investigate the adhesive capacity of treated and untreated cells under conditions of flow, hiMPs were delivered through the top perfusion channel and after 15 min the number of fluorescent cells adhering to the endothelial cells was counted, revealing that DLL4 and PDGF‐BB had no effect on adhesion efficiency of hiMPs (Figs 5F and G; Movie EV1). Nonetheless, this finding does not rule out an effect of the treatment on trans‐endothelial migration which is independent from cell adhesion; however, this hypothesis was difficult to test in the same microfluidic platform, as HUVECs migrated in response to chemoattractants, biasing outcomes of the assay. To overcome this limitation, we assessed trans‐endothelial capacity of treated hiMPs using a trans‐well migration assay. After 7 days of treatment with DLL4 and PDGF‐BB, hiMPs were incubated with a transient fluorescent dye (CFDA, Materials and Methods) and plated onto a monolayer of HUVECs. After 8 h, membranes were fixed and CFDA‐positive, trans‐migrated cells were quantified. As shown in Fig 5H and I, treatment with DLL4 and PDGF‐BB significantly enhanced the ability of hiMPs to migrate through an endothelial monolayer (from 0.59 to 3.19 cells/mm^2^ (*P* = 0.0180) and from 0.50 to 20.68 cells/mm^2^ (*P* = 0.0464), in healthy donor‐derived hiMPs, respectively). Similar results were obtained with hiMPs derived from a DMD patient and genetically‐corrected with a human artificial chromosome containing the entire 2.5 Mb *DYSTROPHIN* genetic locus (DYS‐HAC, Materials and Methods; Choi *et al*, 2016), demonstrating that even after genetic correction, hiMPs remain responsive to the DLL4 and PDGF‐BB treatment (Fig 5H and I). Overall, these findings suggest that DLL4 and PDGF‐BB treatment likely mediates an increase in trans‐endothelial migration via modulation of the latter stages of extravasation, namely, crawling and/or diapedesis but not rolling or adhesion.

### Advanced modelling of 3D tissue migration of hiMPs treated with DLL4 and PDGF‐BB using bioengineered muscles

**Figure 6:**
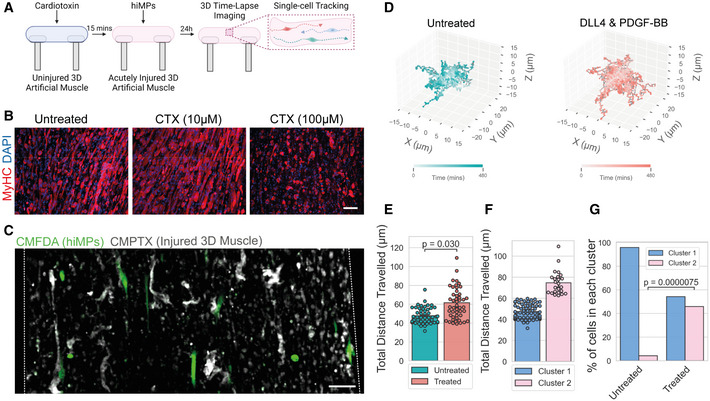
*Quasi vivo* modelling of hiMP tissue migration using 3D human bioengineered muscles Schematic representation of the experimental setup (produced with BioRender, www.biorender.com).Immunofluorescence images of 3D artificial muscles stained for myosin heavy chain (MyHC), after 15 min exposure to PBS (uninjured) or 10/100 μM cardiotoxin. Scale bar: 50 μm.Maximum intensity projections of fluorescence images of CMPTX‐labelled human 3D muscles after background subtraction, stained with CMPTX deposited with containing “transplanted” CMFDA‐labelled (green) hiMPs. Dotted lines demarcate the outline of the 3D construct. Scale bar: 100 μm. Time lapse video availabe in Movie EV2.3D trajectory plots for visualisation of single‐cell tracks of hiMPs on 3D muscles for 8 h for both untreated (left) and DLL4 and PDGF‐BB‐treated (right) conditions.Bar chart representing the total distances travelled of single‐cells tracked for DLL4 and PDGF‐BB and untreated hiMPs. Statistical testing was performed with an independent *t*‐test with each experimental replicate as data points (experimental replicates = 3). Velocities of individual cells are displayed as single points to visualise the distribution of data.Bar chart displaying the velocity of cells within clusters generated using hierarchical clustering of cells using total distance travelled as a feature (S_*i*_ = 0.67). Each point represents a single cell.Bar plots showing the proportions of untreated and DLL4 and PDGF‐BB‐treated cells within the two clusters shown in (F). Statistical test performed with a Chi‐squared (χ^2^) test. Source data are available online for this figure.

Irrespective of the delivery method (i.e. intramuscular or intravascular), transplanted cells eventually will have to migrate through a network of ECM to reach and fuse with degenerating‐regenerating myofibres. To recapitulate the complexity of this process, we developed a novel humanised *quasi vivo* assay by depositing untreated and treated hiMPs on 3D human artificial muscles (Maffioletti *et al*, 2018), and performed time‐lapse imaging and single‐cell tracking (Fig 6A). Additionally, to mimic the microenvironment of dystrophic, degenerating muscles, artificial muscles were acutely injured with the myonecrotic agent cardiotoxin (Fig 6B). Twenty‐four hours after cell deposition, artificial muscles were live‐imaged for 8 h and hiMPs tracked at the single‐cell level (Fig 6C and D; Movie EV2). This revealed a significant increase in the total distance travelled by hiMPs treated with DLL4 and PDGF‐BB in comparison to untreated hiMPs (Fig 6E). Furthermore, hierarchical clustering was performed to identify two clusters based on total distances travelled. Cells of cluster 1 travelled an average of 47.44 μm over 8 h, whilst cells of cluster 2 travelled 74.73 μm in the same timeframe (Fig 6F). After treatment with DLL4 and PDGF‐BB, there was a significant increase in the proportion of cells in cluster 2, from 4.17% in the untreated condition to 45.83% in the treated condition (*P* < 0.001; Fig 6G). Collectively, these results show that modelling intravascular delivery and intramuscular migration in complex humanised platforms validates the observation that modulation of NOTCH and PDGF signalling pathways improves migration of hiMPs across endothelial monolayers and in within regenerating myofibres. These findings lay the foundation for future studies aimed at elucidating and further enhancing the molecular mechanism underpinning this phenomenon.

## Discussion

In this work, we exploited directed iPSC‐differentiation, transcriptomics, single‐cell profiling, microfluidics and 3D tissue engineering to develop a strategy to induce and enhance hiMP migration properties. We show that DLL4 and PDGF‐BB treatment induces a transcriptional profile comparable to those detected in MuSCs from mouse and human primary samples, including key markers of myogenic commitment, downstream NOTCH signalling targets and perivascular markers. This transcriptional response is most likely caused by the role of DLL4 and PDGF‐BB as developmental determinants of skeletal muscle pericytes (Cappellari *et al*, 2013; Moyle *et al*, 2019). Notably, hiMPs responded to treatment more consistently than adult mouse and human myoblasts, possibly due to their relatively immature state compared with their adult counterpart. Interestingly a recent study showed that hiMPs are transcriptionally comparable to late embryonic and early foetal myoblasts (Xi *et al*, 2020). Secondary myogenesis occurs between E14.5–17.5 of mouse development during which foetal myoblasts either contribute to existing primary myofibres or fuse with each other to give rise to secondary muscle fibres (Messina & Cossu, 2009). Spontaneous fate transitions of myoblasts to pericytes were observed in foetuses at E16.5 (Cappellari *et al*, 2013). Therefore, it is possible that hiMPs reflect this plastic foetal myoblast nature and therefore might respond more robustly to DLL4 and PDGF‐BB treatment.

Morphological analysis between treated and untreated hiMPs revealed that DLL4 and PDGF‐BB induced shape changes in a subset of treated cells, in keeping with perturbations of the actin cytoskeleton highlighted by RNAseq data (Fig 3A). Several studies have demonstrated that chemokines enhance myoblast migration via direct regulation of the actin cytoskeleton (Kawamura *et al*, 2004; Ishido & Kasuga, 2011). For example, hepatocyte growth factor‐mediated increase in migration is facilitated by lamellipodia formation via the PI3K/AKT and ERK/MEK signalling pathways (González *et al*, 2017). Additionally, stromal‐derived factor 1 (SDF1), via interaction with CXCR4, increases migration via upregulation of Rho GTPases, *CDC42* and *Rac1* in addition to several other migration‐associated transcripts such as actin bundling protein, *ACTN1* and calcium‐dependent protease, *CAPSN1*, necessary for cleavage of focal adhesions (Kowalski *et al*, 2017). The presence of a subset of cells displaying morphological changes in response to treatment could also indicate the existence of multiple cell states within the hiMP population with differential susceptibility to perturbations of NOTCH and PDGF signalling, also suggested by our migration analyses. Future work should consider correlating single‐cell RNAseq and motility analyses to identify responders, characterising cell states, increasing purity of the treated population and defining molecular mechanisms responsible for increased cell migration.

Any viable treatment to improve engraftment of cell therapy products should not impact negatively on their proliferation and differentiation capacity. DLL4 and PDGF‐BB treatment did not alter hiMP proliferation and the expected NOTCH‐mediated reduction in differentiation was rescued upon removal of the exogenous DLL4 and PDGF‐BB stimuli or, more rapidly by γ‐secretase inhibition of NOTCH signalling.

*In silico* analyses and *in vitro* assays indicated that treated hiMPs possess enhanced motility and trans‐endothelial migration, validating our initial hypothesis that DLL4 and PDGF‐BB modulate migration also in hiPSC derivatives. Microfluidic devices allowed us to interrogate the trans‐endothelial migration process of hiMPs under flow conditions, enabling dissection of two distinct processes that occur during extravasation: adhesion and diapedesis. In leucocytes, trans‐endothelial migration takes place in a step‐wise, sequential manner, consisting of initial adhesion of cells to the endothelial wall and subsequent migration through intercellular gaps of the blood vessel (reviewed in Choi *et al*, 2020). Our results indicate that DLL4 and PDGF‐BB do not modulate the adhesive properties of hiMPs, suggesting a possible mechanism of the treatment on more downstream processes of extravasation. These findings prompted us to explore relevant signalling pathways and molecules involved in cell extravasation. Leukocytes are the typical benchmark for extravasating cells and many genes regulating leukocyte extravasation were found to be differentially expressed upon DLL4 and PDGF‐BB treatment in hiMPs. Interestingly, molecules relevant for leukocyte extravasation such as CXCL12 and integrin β2, were downregulated upon DLL4 and PDGF‐BB treatment, suggesting that myogenic progenitor trans‐endothelial migration may not necessarily mimic all aspects of leukocyte extravasation. For example, downregulation of *JAM‐A* enhances trans‐endothelial migration of adult muscle pericyte‐derived mesoangioblasts (Giannotta *et al*, 2014). Furthermore, intra‐arterial delivery of adult mouse mesoangioblasts in *JAM‐A‐null* dystrophic mice resulted in increased engraftment, indicating that absence of endothelial JAM‐A improves trans‐endothelial migration of myogenic cells (Giannotta *et al*, 2014; Bonfanti *et al*, 2015). This is in contrast to leukocytes, in which isophilic interactions between JAM‐A of leukocytes and endothelial cells are necessary for efficient extravasation (Corada *et al*, 2005; Woodfin *et al*, 2007, 2009). Although not all mechanisms of leukocyte extravasation are mimicked by myogenic progenitors, it remains possible that conserved elements may exist (e.g., Fig 5A). Combining these features with the machinery that other non‐haematopoietic cells use to travel through endothelia (e.g., metastatic cancer cells) could provide additional tools for myogenic cells to efficiently extravasate. Notably, our work goes beyond modelling extravasation, as our newly developed *quasi vivo* migration assay on 3D bioengineered muscles enabled us to study events which follows diapedesis, such as migration within regenerating human myofibres.

Future studies should also investigate *in vitro* high‐throughput and high‐fidelity methods to evaluate cell transmigration, ideally using organotypic (i.e., skeletal muscle‐specific) endothelial and smooth muscle cells on top of a basement membrane, which could facilitate unravelling of adhesion profiles and tissue‐specific recruitment mechanisms necessary for efficient trans‐endothelial migration. This strategy may be more informative than interspecific *in vivo* experiments based upon hiMPs delivery within murine blood vessels, where the species mismatch could affect receptor recognition and downstream signalling (e.g., limited interactions between human‐mouse selectins/integrins). In summary, this study provides an important first step towards defining druggable targets to increase the migration capacity of hiMPs, ultimately contributing to the identification of a systemically deliverable and engraftable hiPSC‐derivative for muscle cell therapies.

## Materials and Methods

### Cell isolation and culture

Primary mMuSCs were isolated, purified via FACS from skeletal muscles of four distinct *Tg:Pax7‐nGFP* F1:C57BL/6:DBA2 mice expressing nuclear‐localised EGFP in Pax7‐expressing cells (Sambasivan *et al*, 2009) and cultured as previously reported (Gerli *et al*, 2019). C2C12 myoblasts (Yaffe & Saxel, 1977) were cultured in DMEM supplemented with 10% FBS, 1% glutamine (Sigma‐Aldrich) and 1% penicillin–streptomycin. Primary human myoblasts from three different donors were obtained from the MRC Neuromuscular Centre Biobank (shortened as L3, L5 and L8), FACS‐purified for CD56^+^ (Biolegend; CD56‐FITC 304604) and cultured as previously reported (Gerli *et al*, 2019); an additional polyclonal population of biopsy‐derived human myoblasts was purchased (Gibco human skeletal myoblasts A12555, shortened in the text as GI) and purified using CD56. Human immortalised myoblasts were kindly provided by the Myoline platform of the Institut de Myologie, Paris, France. Five different hiPSC lines have been used in this study; four lines have been used for most experiments: N1 (short for NCRM‐1: https://hpscreg.eu/cell‐line/CRMi003‐A), N5 (short for NCRM‐5: https://hpscreg.eu/cell‐line/CRMi001‐A); SBI (short for SBIi006‐A: https://hpscreg.eu/cell‐line/SBIi006‐A); A1 (short for: Gibco Episomal hiPSC line A13777); Genetically‐corrected DMD (DYS‐HAC) iPSCs were generated using Sendai‐virus‐delivered reprogramming factors and kindly provided by Dr. Y. Kazuki and Prof. M. Oshimura (Tottori University, Japan; Choi *et al*, 2016). hiPSCs were cultured on vitronectin XF™ (Stemcell Technologies; 07180) at 37°C, 5% CO_2_ and 3% O_2_. mTESR‐E8™ (Stemcell Technologies; 07174) was used for cell expansion and colonies were passaged using gentle dissociation media (Stemcell Technologies; 07174) according to manufacturer's instructions. Skeletal myogenic differentiation of hiPSCs was performed with a commercially available protocol (Genea Biocells, now Myocea; Caron *et al*, 2016). Briefly, hiPSCs were dissociated into single cells and plated onto Matrigel‐coated (Corning) dishes. Subsequently, cells were exposed to induction medium for 10 days to generate myogenic progenitors. The resulting hiMPs were expanded in Genea's myoblast medium, Lonza's myoblast growth and proliferation medium (SKBM‐2) or in‐house DMEM/F12 (Gibco; 11320074)‐based primary myoblast medium (Gerli *et al*, 2019), as detailed in specific sections. All myogenic cells were differentiated in DMEM containing 2% horse serum apart from 3D bioengineered muscle which were differentiated in DMEM containing 10 μg/ml insulin (Gibco; I0516‐5ML). Human cell work was conducted under the approval of the NHS Health Research Authority Research Ethics Committee reference no. 13/LO/1826; IRAS project ID no. 141100.

### DLL4, PDGF‐BB and γ‐secretase inhibitor treatment

Recombinant human DLL4 (DLL4 fused with the Fc domain of human IgG; R&D Systems; 1506‐D4) was resuspended to a final concentration of 10 μg/ml in sterile PBS containing 1% wt/vol bovine serum albumin (BSA; Sigma‐Aldrich; A9418‐10G) as a carrier protein. Standard cell culture plastic dishes were coated with the DLL4 solution and incubated at 37°C for 45 min. Cells were then seeded on the coated flasks and supplemented with 100 ng/ml of human PDGF‐BB re‐suspended in 0.1% BSA/4 mM HCl/PBS (R&D Systems; 200‐BB‐050) daily for at least 7 days. As for normal myogenic differentiation assays, DLL4 and PDGF‐BB‐treated myoblasts and untreated control were seeded at a high density on collagen‐coated dishes and, when confluent, switched to differentiation medium (DMEM supplemented with 2% HS (w/v) + 1% P/S (w/v)). To block NOTCH signalling, cells were incubated with 660 ng/ml of *γ*‐secretase inhibitor (L685458, Sigma) 24 h before the switch to differentiation medium and over the two following days.

### RNA sequencing

#### RNA library preparation

Mouse MuSC‐derived myoblasts, human myoblasts and hiMPs (detailed in previous section “Cell isolation and culture”) were seeded on dishes coated with 10 μg/ml DLL4 and medium supplemented daily with 50 ng/ml recombinant PDGF‐BB with a minimum of 1 passage throughout the 7 days reprogramming protocol to replace the DLL4 protein. An untreated control was grown in parallel on 1% BSA‐coated dishes. After 7 days, samples were collected and RNA extracted with Qiagen RNeasy kit, with on‐column DNaseI treatment. RNA concentration and integrity were assessed by Nanodrop spectrophotometer and Agilent 2100 bioanalyzer (model G2939A). An RNA Integrity Number (RIN; Schroeder *et al*, 2006), was quantified for each sample and scores between 9.8 and 10 accepted. Library preparations were performed with the UCL Genomics facility, using the KAPA mRNA HyperPrep Kit (Roche) to capture mRNA and deplete ribosomal RNA. Samples were barcoded and run together on an Illumina NextSeq 550 System to minimise batch variation.

#### Analyses

Raw sequence data were pre‐processed to remove small (> 20 bp) or poor quality reads using Trimmomatic v0.36.4 (Bolger *et al*, 2014). Reads were aligned either to the Human hg38 genome using Spliced Transcripts Alignment to a Reference (STAR) software v2.5.2b (Dobin *et al*, 2013), mapped reads de‐duplicated with Picard v2.7.1.1 (Broad Institute) and reads‐per‐transcript calculated with feature Counts v1.4.6.p5 read summarisation tool (Liao *et al*, 2014). Finally, differential expression was calculated using SARTools R package v.1.3.2.0 (Varet *et al*, 2016), based on the DESeq2 model and package (Love *et al*, 2014).

In order to perform gene set enrichment analyses, mouse gene symbols were first converted into their respective human orthologs using the BiomaRt v2.46.2 package (Smedley *et al*, 2009). Subsequently, HUGO Gene Nomenclature Committee (HGNC) symbols were converted into Entrez Gene IDs using BiomaRt. Differentially expressed genes with a fold‐change > 2 and a *P*‐value < 0.05 were then subjected to gene set enrichment analysis with ClusterProfiler v3.18.0 and ReactomePA v1.34.0 packages (Yu *et al*, 2012; Yu & He, 2016); script available as Dataset EV1.

To analyse signalling pathway changes in response to DLL4 and PDGF‐BB, differential expression data were inputted into Ingenuity Pathway Analysis (IPA, Qiagen). The Genebank gene ID, log2 fold change expression, *P*‐value and adjusted *P*‐values (*P*adj) were included, in order to account for the experimental false discovery rate. To ensure only highly likely interactions were accounted for, only experimentally observed interactions in mammalian cells were included, and cut‐offs were set at log2 fold change (−0.58, +0.58 i.e. a fold change of 1.5) with a padj of 0.05. From this, 2,259 genes (1,002 increased, 1,557 decreased) remained on the hiMP dataset. Additional expression analyses were performed using Stemformatics (www.stemformatics.org/; Choi *et al*, 2019). Functional protein association network analysis was performed using https://string‐db.org. RNAseq reads and scripts utilised for PCA and gene enrichment analyses available upon request to the corresponding author. European Nucleotide Archive (ENA) study accession number: PRJEB43338.

### Quantitative real‐time PCR

Cells were seeded on 6‐ or 12‐well plates for at least 24 h before detaching them and centrifuging at 336.47 *g* to obtain pellets for RNA extraction using RNeasy Micro kit (Quiagen, 74004) according to manufacturer's instructions. RNA purity and yield were assessed using a Nanodrop spectrophotometer. Retro‐transcription to cDNA was performed with the ImProm‐IITM Reverse Transcription System kit (Promega, A3800) following manufacturer's instructions; a minimum of 50 ng of RNA per reaction was used. qRT‐PCRs were performed with the SYBR‐Green Real Time Master Mix (Promega; A600A) according to manufacturer's instructions using a BioRad CFX96 machine. qRT‐PCRs were performed in triplicate on samples from at least three independent experiments. Ct data were normalised to GAPDH (Stern‐Straeter *et al*, 2009). Data were presented as mean ± SEM of the fold change. Significance was assessed on the delta Ct values using Student's two‐tailed *t*‐test. List of primers used are available in Table 2.

**Table 2:** List of primers used.

Gene	Forward primer	Reverse primer
*GAPDH*	TTCACCACCATGGAGAAGGC	GGCATGGACTGTGGTCATGA
*PAX7*	CAAACACAGCATCGACGG	CTTCAGTGGGAGGTCAGGTT
*MYOD*	AATAAGAGTTGCTTTGCCAG	GTACAAATTCCCTGTAGCAC
*MYOGENIN*	CCAGGGGTGCCCAGCGAATG	AGCCGTGAGCAGATGATCCCC
*PDGFRB*	AGCTGTTACCCACTCTGGGA	TGGTGTCCTTGCTGCTGATG
*TNAP*	TGTGGGGTGAAGGCCAATG	GTGGTGGTCACAATGCCCA
*CD146*	GGAAGCAGGAGATCACGCTAC	GATTCGGGGCTAATGCCTCA
*HEY1*	AGGTTACTTTGACGCGCACG	ACCAGTCGAACTCGAAGCG
*HES1*	AGAAAGATAGCTCGCGGCA	TACTTCCCCAGCACACTTGG

### Immunofluorescence

Cells were fixed in 4% paraformaldehyde (PFA) for 5 min at room temperature (RT), washed twice with phosphate buffered saline (PBS) and incubated 30 min with PBS‐1% BSA‐0.2% triton. Cells were then incubated for 30 min with 10% donkey or goat serum solution at RT to reduce non‐specific antibody binding. Primary antibodies were diluted to the appropriate concentration (Table 3) in PBS‐1% BSA‐0.2% triton and incubated either 1 h at RT or overnight at 4°C. Subsequently, cells were washed three times with PBS‐0.2% triton to eliminate unbound antibody and then incubated for 1 h with fluorescently conjugated secondary antibodies raised in goat or donkey and Hoechst 33342 to visualise nuclei (Fluka; B2261). Cells were imaged using an inverted fluorescence microscope (Leica DMI6000B). At least 5 non‐overlapping random field images were acquired and analysed using ImageJ or Adobe Photoshop software.

**Table 3:** List of antibodies used.

Antibody	Dilution	Company; Catalogue number
Anti‐MyoD	1:100	Santa Cruz; sc‐760 (M‐318)
Anti‐Pax‐6	1:100	Santa Cruz; sc‐81,649
Anti‐MAP‐2	1:100	Santa Cruz; sc‐74,421
Anti‐MyHC	1:9	DSHB; MF20
Anti‐CD31	1:40	Abcam; 28,364
Anti‐SPECTRIN	1:100	Leica Biosystems; NCL‐SPEC1
Anti‐Lamin A/C	1:250	Novocastra; NCL‐LAM
Phalloidin	1:400	Invitrogen; A30137

### Fluorescence activated cell sorting (FACS)

Cells were prepared for FACS analysis as previously published (Maffioletti *et al*, 2015). Briefly, cells were trypsinised and filtered through a 40 μm cell strainer to get a single cell suspension. At least 1.5 × 10^5^ cells were stained for each fluorochrome‐conjugated primary antibody for 1 h on ice. An additional unstained control tube was included for each cell line. Cells were then washed, fixed in 2% (w/v) paraformaldehyde for 5 min after which 3 ml of FACS buffer was added and cells centrifuged at 232 *g* for 5 min. Supernatant was discarded and cells were resuspended in 100 μl FACs buffer and sorted with a CyAn™ ADP Analyser (Beckman Coulter, Inc.) at the UCL GOSICH Flow Cytometry Core Facility. A minimum of 20,000 events per antibody were analysed. FACS data analysis was done using FCS Express 4 (*De Novo* Software). A similar procedure was followed for FACS cell purification, apart from fixation. Cells were sorted using a MoFlo XDP machine (Beckman Coulter).

### Morphometry and proliferation analyses

To compare morphology between DLL4 and PDGF‐BB‐treated and untreated hiMPs, the circularity ratio of cells was analysed using ImageJ (0 = line; 1 = perfect circle). Circularity ratios of cells were obtained via quantification of manually labelled cell contours of phase‐contrast images. Three random fields were analysed for three independent experiments with at least 300 cells analysed for each biological replicate. To identify differences in proliferation between DLL4 and PDGF‐BB‐treated and untreated hiMPs, cells were pulsed with 10 μM 5‐Ethynyl‐2'deoxyuridine (EdU) for 2 h following manufacturer's instructions (Click‐iT^®^ EdU Alexa Fluor^®^ Imaging Kit, Life Technologies). Cells were subsequently fixed and stained with Hoechst 33342. The proportion of proliferating cells was then calculated by comparing the number of EdU+ nuclei with the total number of nuclei within the field.

### Cell motility assays

For conventional motility assay, 1.5 × 10^4^ hiMPs were plated in triplicate onto 24‐well multi‐well dishes and incubated overnight. For conditions of continuous treatment, hiMPs were plated onto 24‐well dishes coated with either 1% BSA or DLL4 and supplemented with PDGF‐BB. Cells were pulsed with Hoechst 33342 (100 ng/ml) for 45 min prior to imaging to aid tracking. Imaging was performed with the ImageXpress acquiring images every 10 min for 12 h (segmented using ImageJ). Cell tracking, calculation of total distance travelled (μm), velocity (μm/min), mean straight line speed (μm/min) and total displacement (μm) was performed with Trackmate (Tinevez *et al*, 2017). Only cells remaining within the field were analysed. Statistical analysis was performed on three independent repeats with a minimum of 20 cells/condition/repeat.

For analysis of cell motility with Heteromotility, image segmentation was performed using a deep learning approach. A StarDist model was trained on manually annotated images obtained from individual frames of the tracking dataset selected to capture variation in intensity, shape and size between nuclei. Images were annotated with Caliban (https://github.com/vanvalenlab/caliban) and the StarDist model was trained from scratch using the ZeroCostDL4Mic platform (Schmidt *et al*, 2018; von Chamier *et al*, 2021). Single‐cell tracking was performed with Bayesian Tracker with modified configurations to optimise tracking for videos obtained from ImageXpress (Bove *et al*, 2017; https://github.com/quantumjot/BayesianTracker). Analysis of single‐cell tracks of lengths > 60 was subsequently performed with Heteromotility using all features except turning features (Kimmel *et al*, 2018; https://github.com/cellgeometry/heteromotility). The following parameters were utilised: “total_distance” travelled by the cell during time‐lapse; “net_distance” travelled by the cell during time‐lapse; “linearity”: linear regression analysis of the XY coordinates of a cell at each time point; “spearmanrsq”: assessment of the monotonic relationship of the distribution of XY coordinates of cells at each time point;” progressivity”: ratio between “net_distance” and “total_distance”, serving as an indicator of the directional nature of the cell track during time course. Larger values suggest directional motility; “max_speed”, “min_speed” and “avg_speed”: maximum/minimum/average speed of a cell during time‐lapse; “MSD_slope”: spatial deviation of a cell with respect to a reference position during time‐lapse. Higher values suggest directional motility whilst lower values indicate random motion; “hurst_RS”: a metric of directional persistence. Values < 0.5 suggests non‐persistent behaviour. A value of 0.5 indicates brownian motion. Values between > 0.5 and 1.0 indicate persistent behaviour; “nongauss”: the extent of the non‐Gaussian nature of the distribution of displacement of the cell within timelapse—value closer to 0 indicate Gaussian distribution; “rw_linearity”: linearity of a cell track minus linearity of a simulated random walk; “rw_netdist”: net distance travelled by a cell minus net distance of a simulated random walk; “rw_kurtosis”: kurtosis of a cell displacement minus kurtosis of a random walk for each sub‐track; “avg_moving_speed” of a cell during a specified sub‐track; “time_moving”: proportion of time spent moving by the cell during a sub‐track; “autocorr”: similarity of a cell displacement series as a function of time lag between each displacement.

### Microfluidic and transwell assays

Microfluidic assessment of cell adhesion was performed using the OrganoPlate^®^ 3‐lane 40 and the OrganoFlow^®^ S (Mimetas). 3D channels were endothelialised with HUVECs (Lonza) cultured in EGM1 medium (Lonza) at 37°, 5% CO_2_. The “OrganoPlate^®^ 3‐lane tubule seeding” protocol (www.mimetas.com/en/knowledge‐center/) was followed with minor alterations. First, the 4 mg/ml collagen‐I mixture was prepared by mixing 5 mg/ml collagen‐I (AMSbio: 3447‐020‐01), 1 M HEPES (Thermofisher: 15630130), and 37 g/l NaHCO_3_ in a 8:1:1 ratio. 2 μl of the mixture of was added to the central channel of each chip and the plate was subsequently incubated at 37°C for 15 min to facilitate collagen polymerisation. 3 × 10^5^ HUVECs were then seeded into the top medium inlet and the plate was left on a 70° angle to allow direct attachment of HUVECs against the ECM gel for 5 h. Following cell attachment, the plate was placed on an OrganoFlow^®^ S (Mimetas; 7°; 8 min) to induce flow. All tubules were used between 48 and 72 h after seeding. Tubules were fixed with 4% PFA for 10 min at RT. Culture medium was aspirated and PFA was added to wells at the following volumes: 100 μl in the top medium inlet, 50 μl in the top medium outlet, gel and bottom medium inlets and outlets. Staining of chips was performed according to manufacturer's instructions (www.mimetas.com/en/knowledge‐center/) with minor modifications. All steps, unless specified otherwise were performed with the following volumes in each well: 100 μl in the top medium inlet, 50 μl in the top medium outlet as well as gel and bottom medium inlets and outlets. First, cells were permeabilised with 1% BSA wt/vol and 0.2% Triton‐X in PBS for 30 min. A blocking step was then performed using 2% FBS and 2% BSA in PBS for 30 min. Primary antibodies were diluted to appropriate concentrations in permeabilisation buffer, 25 μl were added to the top medium inlets and outlets and 15 μl was added to the bottom medium inlets and outlets. After three washes with 0.2% Triton/PBS, fluorescently conjugated secondary antibodies, diluted in 0.2% Triton‐X/PBS, were added to chips at the same volumes as the primary antibody solution and incubated for 1 h at room temperature. Subsequently, two washes with 0.2% Triton‐X/PBS and one wash with PBS was performed prior to imaging. Barrier integrity assays were performed following manufacturer's instructions (www.mimetas.com/en/knowledge‐center/) with minor adaptations. A “wetting step” was performed prior to the assay by adding 50 μl EGM1 to the gel and bottom perfusion inlets and outlets for 5 min. A dextran working solution of 0.5 mg/ml 20 kDa FITC dextran and 0.5 mg/ml 150 kDa TRITC dextran was prepared in EGM1. All media was removed from the top medium inlet and outlet, whilst 20 μl of media was left in the gel and bottom perfusion inlets and outlets. Forty microliter and 30 μl of the dextran working solution was added to the top perfusion inlet and outlet, respectively. Imaging was initiated immediately after addition of the dextran solution every 3 min for 15 min on the LTTL system. Normalised intensity was calculated as a ratio of fluorescence between the top perfusion channel and ECM channel. Quantification of fluorescence was performed using FIJI by manual annotation of regions of interest (ROI) within the perfusion and ECM channels. The ROI was kept consistent across time points, fluorescence channels, top and central tubules. The integrated density, the sum of all pixel values within the ROI, was used as the measurement to calculate normalised intensity. To assess cell adhesion on endothelialised tubules under flow condition, cells labelled with 2.5 μM 5‐chloromethylfluorescein diacetate (CMFDA; ThermoFisher Scientific) were resuspended in a mixture of myoblast proliferation medium (MM; Gerli *et al*, 2019) and EGM1 at a 1:1 ratio (400 cells/μl). After generation of blood vessels at least 48 h prior to seeding, 50 μl of MM/EGM1 media was added in the perfusion outlets. Subsequently, 50 μl of cell suspension was added to top medium inlets. The OrganoPlate^®^ was then placed on the OrganoFlow^®^ S (7°; 4 min) for 15 min. Chips were imaged using the EVOS™ M5000. Adhesion was calculated by counting the number of in‐focus attached cells within the top perfusion channels after 15 min on the OrganoFlow^®^ S. After imaging adhesion, we attempted to use the same chips to assess transmigration; however, this was complicated by HUVECs consistently migrating in the ECM channel in response to chemoattractants, resulting into disruption of the endothelial cell architecture in the tubules. This confounding factor and background noise from HUVECs prompted us to assess trans‐endothelial migration using transwell dishes as an alternative assay.

*In vitro* trans‐endothelial migration (transwell) assay was performed using HUVECs (Lonza) grown at 37°C, 5% CO_2_ in EGM1 (Lonza) on 1% gelatin‐coated flasks (Sigma), kept below 70% confluence and used up to passage 6. Eight micrometer porous cell culture membranes (BD Biosciences; 353093) were coated with 1.5% gelatin for 1 h at 37°C, cross‐linked with 2% glutaraldehyde (Sigma) for 15 min at RT, incubated with 70% ethanol for 1 h at RT and washed three times with PBS before an overnight incubation in 2 mM glycine/PBS at 4°C. After 5 PBS washes, 2 × 10^5^ HUVECs were seeded on top and grown to confluence for at least 72 h. hiMPs were then dissociated with TrypLE Select (Thermo Fisher Scientific) and labelled with 0.7 μM 6‐carboxyfluorescin dictate (6‐CFDA; ThermoFisher Scientific) for 30 min at 37°C. Subsequently, the upper chamber was loaded with 3 × 10^4^ cells to be tested, resuspended in serum‐free medium. The lower chamber was loaded with a chemoattractant composed of 50% fresh growth medium and 50% medium previously exposed for 24 h to differentiated C2C12 myoblasts. After 8 h, membranes were washed in PBS and fixed for 5 min in 4% PFA. The upper side of the membrane was scraped with a cotton bud to remove non‐migrated cells. After an additional PBS wash, membranes were mounted on slides and cells migrated through the endothelial layer were quantified by counting the number of fluorescent cells on the lower side of the membrane using a Leica DMI6000B microscope. A minimum of 10 random 20× field/condition per experiment was quantified. Experiments were performed in duplicate on at least three separate occasions.

### Migration assay on 3D bioengineered human muscles

3D artificial muscles were generated using immortalised human myoblasts as previously described (Maffioletti *et al*, 2018; Pinton *et al*, 2022). hiMPs were stained with 2.5 μM CMFDA for 30 min at 37°C, 5% CO_2_ and 3D muscles were stained with 5 μM CMPTX (ThermoFisher Scientific; C34552) for 45 min at 37°C, 5% CO_2_. Acute injury was induced in 3D muscles by depositing 5 μl cardiotoxin (CTX; Sigma) on their surface for 15 min at RT; muscles were then washed in warm PBS. hiMPs were resuspended in 50% MM media, 50% matrigel (8,000 hiMPs/μl). Five microliter of hiMP suspension was deposited onto the surface of the bioengineered muscles and incubated without media for 10 min at 37°C, 5% CO_2_ to facilitate Matrigel polymerisation before returning them to 24‐well multi‐well plates with MM media. After 24 h, 3D muscles were subjected to time‐lapse microscopy with a CSU‐W1 spinning disk microscope over 12 h with imaging performed every 12 min with a 10× objective. A 100 μm z‐stack was taken every 5 μm.

Analysis of 3D migration was performed with Imaris (version 9.0.1). First, a 2 × 2 binning was performed and images of both hiMPs and 3D muscle were subjected to median filtering and background subtraction prior to segmentation and tracking. To correct for drift of the 3D muscle, a mean trajectory of the muscle was approximated. This involved identifying highly correlated tracks within the 3D muscle and was performed using the cosine similarity metric with a threshold of 0.95. The average position at each time point of all correlated tracks was subsequently calculated to be used as the mean trajectory to normalise hiMP trajectories. Of note, this assay can be done in a fully isogenic setting also using 3D muscles generated from the same iPSC source utilised to derive hiMPs (Pinton *et al*, 2022).

### Cell transplantation

Intramuscular transplantation of hiMPs in adult male NOD/scid/gamma (NSG) mice (*N* = 3) was done as previously described (Benedetti *et al*, 2018) with some minor modifications. Cryoinjured tibialis anterior muscles were harvested 18 days following transplantation with treated (*n* = 3) and untreated (*n* = 3) 3 × 10^5^ N5 hiMPs and immunostained for stained LAMIN A/C (human nuclei; Leica NCL‐LAM) and SPECTRIN (sarcolemma; Leica NCL‐SPEC1). Operator was blinded on the cell treatment during transplantation and muscle harvesting. Animal work was performed following under UK Home Office project licences no. 70/8566 and PP2527748.

### Statistical analysis

Number of experimental replicates is specified in figure legends and key experiments were repeated at least three times prior to any statistical testing (“*N*” refers to independent experiments or individual animals, “*n*” to data points). Sample size estimate was informed by expected results based upon previous work with similar treatment (Gerli *et al*, 2019). Where appropriate, subjective bias was minimised by blinding or automation of analysis (e.g. motility assays). Quantification, data distribution and statistical testing were performed using Microsoft Excel and GraphPad Prism 9 software. Statistical testing was based on Student's *t*‐test unless otherwise stated. Error bars (standard deviation: SD; standard error of mean: SEM) are specified in figure legends. *P* values are specified in each figure on top of individual graphs.

## Author contributions

**SungWoo Choi:** Conceptualization; resources; data curation; software; formal analysis; validation; investigation; visualization; methodology; writing – review and editing. **Giulia Ferrari:** Conceptualization; data curation; formal analysis; investigation; methodology. **Louise A Moyle:** Conceptualization; formal analysis; investigation; methodology. **Kirsty Mackinlay:** Formal analysis. **Naira Naouar:** Data curation; formal analysis; methodology. **Salma Jalal:** Formal analysis; methodology. **Sara Benedetti:** Funding acquisition; investigation; methodology. **Christine Wells:** Data curation; formal analysis; supervision; investigation; writing – review and editing. **Francesco Muntoni:** Supervision; investigation; writing – review and editing. **Francesco Saverio Tedesco:** Conceptualization; data curation; formal analysis; supervision; funding acquisition; validation; investigation; methodology; writing – original draft; project administration; writing – review and editing.

In addition to the CRediT author contributions listed above, the contributions in detail are:

Conceptualisation: FST, GF, SWC, LAM; Methodology: SWC, GF, LAM and FST; Investigation: GF, SWC, LAM, KM, SJ, SB, NN, CW, FM and FST; Writing: FST, GF and SWC; Funding acquisition and coordination: FST. SWC and GF contributed equally to this work; their names are listed in alphabetical order and their respective position is commutative.

## Disclosure and competing interests statement

FST has received speaker and consultancy honoraria from Takeda, Sanofi Genzyme and Aleph Farms (via UCL Consultants). All other authors declare that they have no conflict of interest.

**For more information**

- Lab website: www.tedescolab.org
- Heteromotility source code: https://github.com/cellgeometry/heteromotility
- Stemformatics: https://www.stemformatics.org
- Functional protein association network analysis: https://string-db.org
- Relevant patient associations (examples): https://www.musculardystrophyuk.org

## Supporting information



Appendix

Expanded View Figures PDF

Dataset EV1

Movie EV1

Movie EV2

Source Data for Expanded View

PDF+

Source Data for Figure 2

Source Data for Figure 3

Source Data for Figure 4

Source Data for Figure 5

Source Data for Figure 6

## Data Availability

RNAseq data are available in European Nucleotide Archive (ENA; www.ebi.ac.uk/ena) within study accession number: PRJEB43338 (http://www.ebi.ac.uk/ena/data/view/PRJEB43338).
