# Supplementary material for: Assessing and enhancing migration of human myogenic progenitors using directed iPS cell differentiation and advanced tissue modelling
Source: EMBO Mol Med. 2022 Sep 26;14(10):e14526. doi: 10.15252/emmm.202114526 (PMC9549733; doi:10.15252/emmm.202114526)
Supplement: Supplementary file 1 — Appendix [file EMMM-14-e14526-s003.pdf]

APPENDIX

Assessing and enhancing migration of human myogenic progenitors using directed iPS cell differentiation and advanced tissue modelling

SungWoo Choi[1,2,#], Giulia Ferrari[2,#], Louise A. Moyle[2,§], Kirsty Mackinlay[2,Ø], Naira Naouar[3], Salma Jalal[2], Sara Benedetti[4,5], Christine Wells[6], Francesco Muntoni[5,7], Francesco Saverio Tedesco[1,2,7]*

[1]The Francis Crick Institute, 1 Midland Road, London, NW11AT, UK
[2]Department of Cell and Developmental Biology, University College London, London, WC1E6DE, UK
[3]Sorbonne Université, Institut de Biologie Paris Seine FR3631, Plateforme de bioinformatique ARTbio, Paris, France.
[4]UCL Great Ormond Street Institute of Child Health, University College London, London, UK
[5]National Institute for Health Research Great Ormond Street Hospital Biomedical Research Centre, London, UK
[6]Centre for Stem Cell Systems, The University of Melbourne, Melbourne, Victoria, Australia.
[7]Dubowitz Neuromuscular Centre, UCL Great Ormond Street Institute of Child Health & Great Ormond Street Hospital for Children, London, UK
[#]Equally contributing authors
[§]Current address: Institute of Biomedical Engineering, University of Toronto, Toronto, M5S3E1, Canada
[Ø]Current address: University of Cambridge, Department of Physiology, Development and Neuroscience, Cambridge CB23DY, UK
*Corresponding author (f.s.tedesco@ucl.ac.uk)

Table of Contents

- Appendix Figure S1. Top 50 differentially regulated genes in mMuSC-derived myoblasts, human myoblasts and hiMPs.
- Appendix Figure S2. Cross-comparison of top 50 differentially regulated genes of treated mMuSC-derived myoblasts in human myoblasts and hiMPs.
- Appendix Figure S3. Assessment of differentiation of hiMPs expanded in DMEM/F12-based in-house primary myoblast medium.
- Appendix Figure S4. Assessment of spontaneous differentiation of DLL4 & PDGF-BB treated hiMPs.
- Appendix Table S1. Top 50 genes responsible for variations of PC1 and 2 in the principal component analysis shown in Fig 2A.
- Appendix Table S2. Full list of genes of heatmaps shown in Appendix Fig S1 displaying 50 genes which exhibit either the greatest up- or down-regulation upon treatment with DLL4 & PDGF-BB in mMuSC (left), human myoblasts (centre) and hiMPs (right).
- Appendix Table S3. Table of ranked genes supplementing heatmaps presented in Appendix Fig S2.
- Appendix Table S4. Table of ranked genes accompanying heatmaps “Regulation of cell morphology”, “Proliferation of stem/myogenic cells” and “Leukocyte trans-endothelial migration” (Figures 3A, 3D and 4H, respectively).

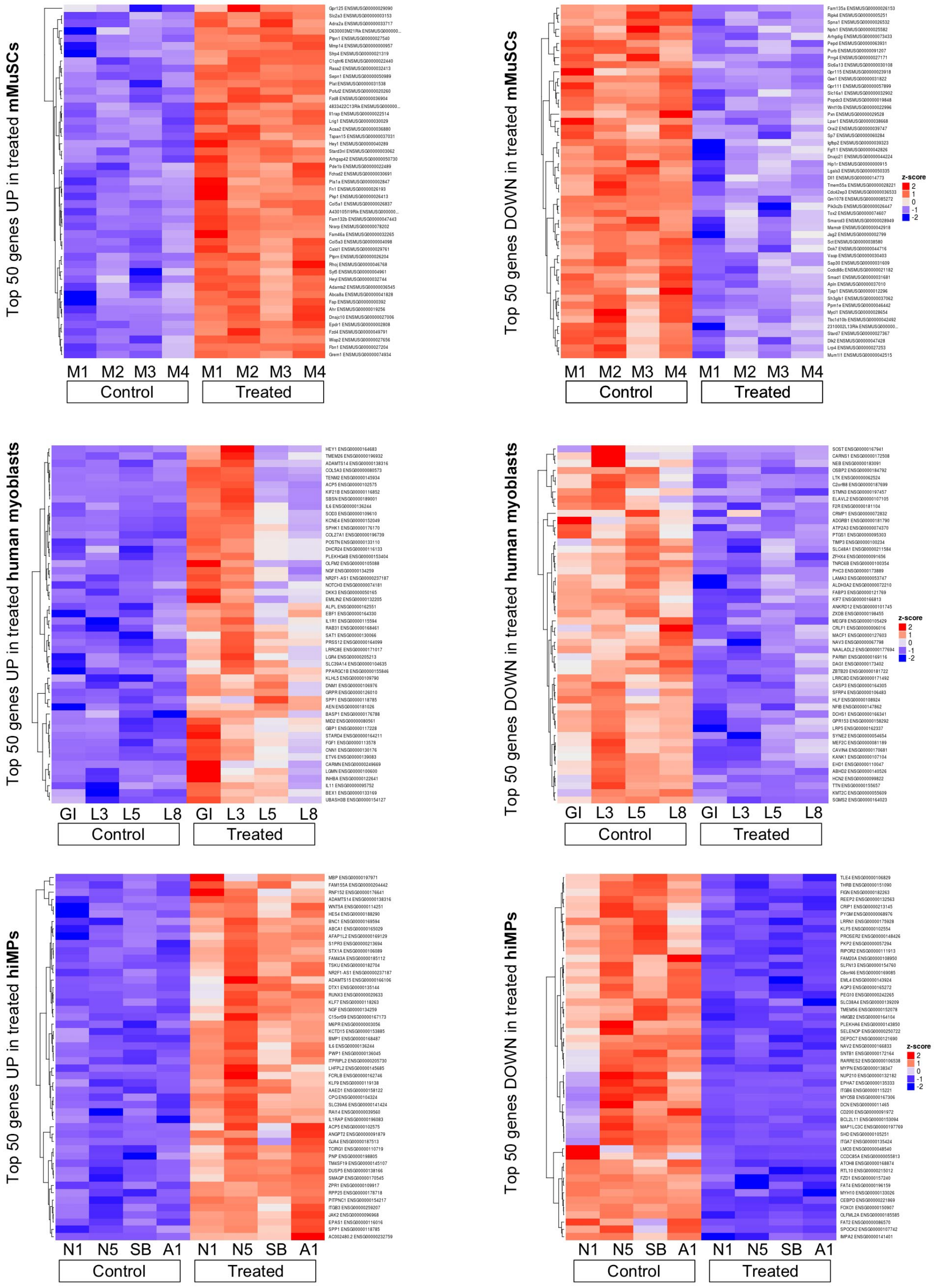

Appendix Figure S1. Top 50 differentially regulated genes in mMuSC-derived myoblasts, human myoblasts and hiMPs. Heatmaps displaying 50 genes which exhibit either the greatest up- (left) or down-regulation (right) upon treatment with DLL4 & PDGF-BB in mMuSC (top), human myoblasts (centre) and hiMPs (bottom). Additional information (details on gene list) in Appendix Table S3.

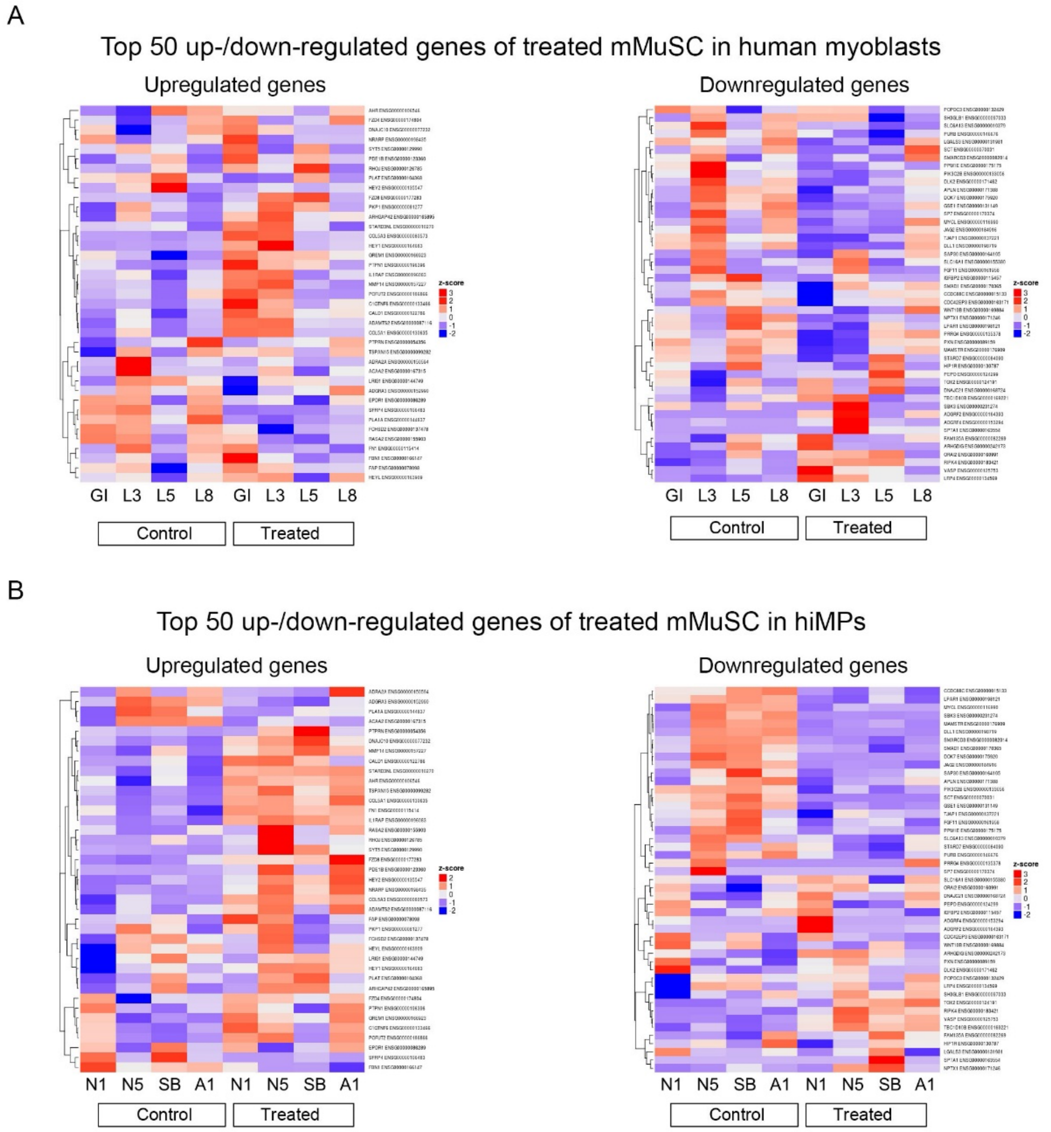

Appendix Figure S2. Cross-comparison of top 50 differentially regulated genes of treated mMuSC-derived myoblasts in human myoblasts and hiMPs. (A) Heatmaps of the top 50 up- (left) and down-regulated (right) genes of DLL4 & PDGF-BB-treated mMuSC-derived myoblasts in treated and untreated human myoblasts. **(B)** Heatmaps of the top 50 up- (left) and down-regulated (right) genes of DLL4 & PDGF-BB-treated mMuSC-derived myoblasts in treated and untreated hiMPs.

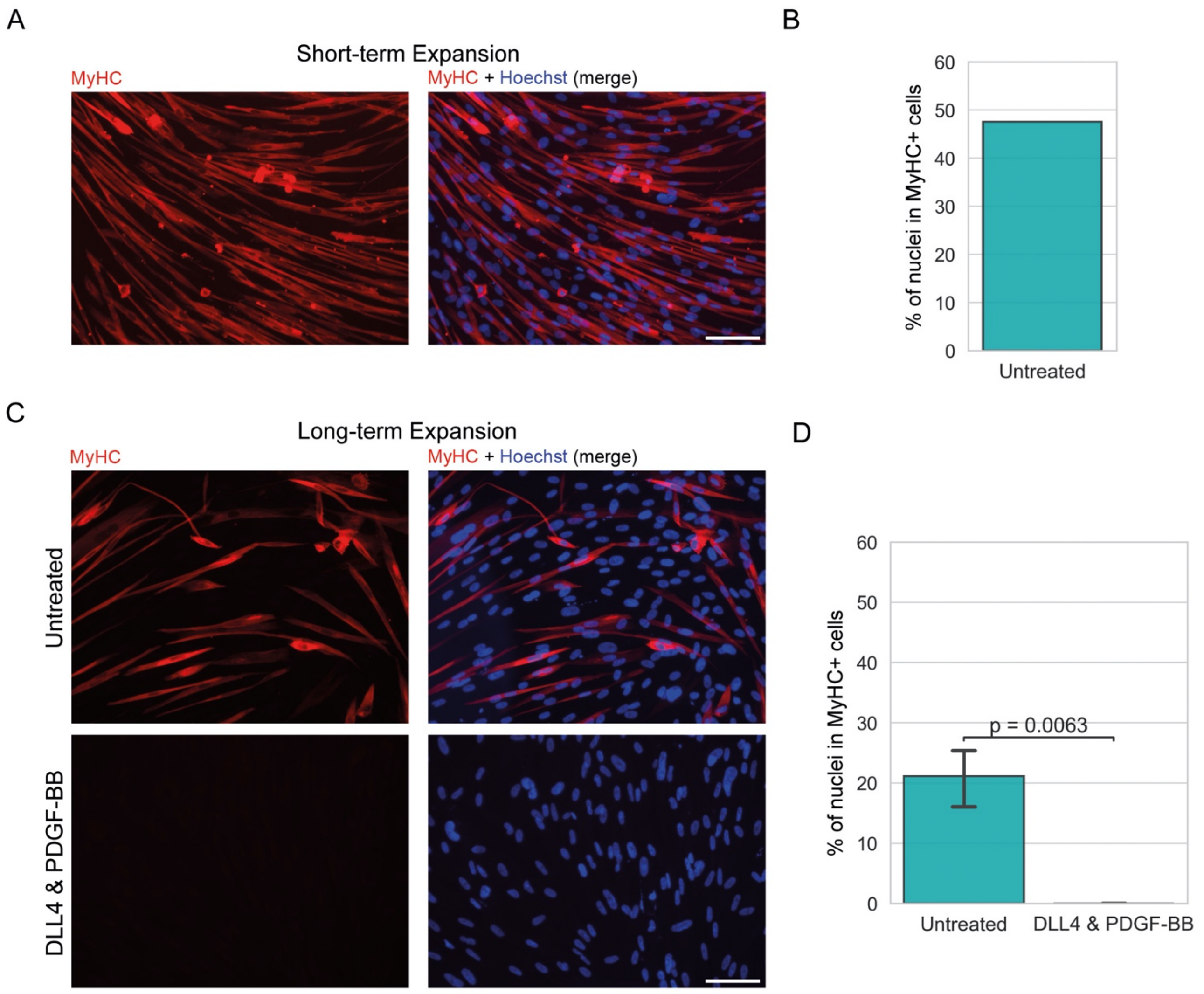

Appendix Figure S3. Assessment of differentiation of hiMPs expanded in DMEM/F12-based in-house primary myoblast medium. (A) Representative immunofluorescence images of hiMPs expanded in the in-house primary myoblast medium for 7 days (short term) (technical replicates = 2). Scale bar = 100μm. **(B)** Bar chart showing the quantification of terminal myogenic differentiation in (A). **(C)** Immunoflurescence images of hiMPs, 4 days post-differentiation into myotubes after expansion in the primary myoblast medium for longer than 7 days in either untreated or treated conditions. **(D)** Bar chart displaying the differentiation index for images in (C) (experimental replicates = 3). Scale bar = 100μm.

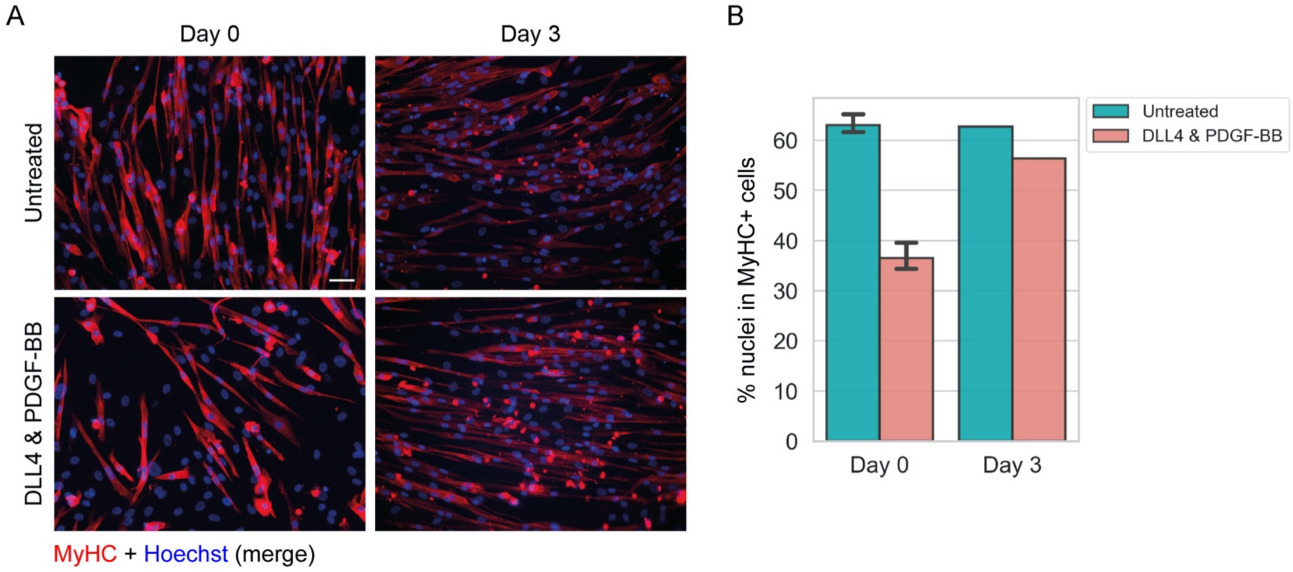

Appendix Figure S4. Assessment of spontaneous differentiation of DLL4 & PDGF-BB treated hiMPs. (A) Representative immunofluorescence images of untreated and DLL4 & PDGF-BB-treated hiMPs differentiated into myotubes for 4 days either immediately after 7 days of treatment or after proliferation of uncoated plastic dishes for 3 days. **(B)** Bar graph quantifying the differentiation of images in (A) using the differentiation index (Experimental replicates = 3 for Day 0; Experimental replicates = 1 for Day 3). Scale bar = 50µm.

	mMuSCs PC1 (45%)	mMuSCs PC2 (35%)	hMBs PC1(50%)	hMBs PC2 (25%)	hiMPs PC1 (63%)	hiMPs PC2 (17%)
1	Col15a1	Il33	COL5A3	XIST	TTN	EPGN
2	Col6a2	Actc1	TRH	RPS4Y1	MYBPH	CPA4
3	Col1a1	Flt1	TTN	DDX3Y	SYNPO2	SFRP1
4	Col6a1	Tek	SLC14A1	USP9Y	TNNI1	C3
5	Grem1	Jag1	OLFM2	NOS1	MYH8	UNC5B
6	Pdgfrb	Usp43	POSTN	KDM5D	TNNT2	SRGN
7	Heyl	Cyp2j6	ASS1	ZFY	CHRND	GDF6
8	Bgn	Myog	CRISPLD2	MYH3	CHRNA1	RGS4
9	Adamts2	Myo5b	ZNF469	LINC00261	KLHL41	OXTR
10	Sfrp4	Tnnt1	ALDH1A1	EIF1AY	SHD	IL1RL1
11	Col6a3	Tnnc2	STMN2	NLGN4Y	XIRP1	MSC
12	Col5a3	Atp2a1	PTGIS	ERAP2	ACTC1	GUCY1A2
13	Lrrc32	C1qtnf3	KLHL41	ELN	RYR1	HOXB9
14	Cdh11	Mylpf	MYH3	HLA-A	ENO3	INHBE
15	Nrarp	Grb10	IGFBP5	TXLNGY	CDH15	ARRDC4
16	Itgb3	Nefm	INA	COL11A1	UNC45B	TRIB3
17	Thy1	Sp7	NOTCH3	F13A1	FNDC5	CHRDL1
18	Igfbp7	Klhl41	SERPINE2	STMN2	TNNC1	DIO2
19	Pkp1	Smyd1	KCNE4	MMP1	MYH3	ZNF280D
20	Cyp1b1	Lepr	JAG1	EBF2	SRL	PRKG1
21	Mgp	Sema3d	LAMA3	CLGN	DES	GPRC5C
22	Serping1	Igf2	MYBPH	TNNI1	ACTA1	NLGN1
23	Ctgf	Meg3	NEB	UTY	SMYD1	NPY1R
24	Apbb1ip	Podxl	TENM2	SFRP1	MYH7	DDIT3
25	Fap	Synpo2l	NTSR1	RARRES2	VGLL2	CCDC3
26	Pcp4l1	Rian	TNFRSF1B	TNNT2	MYOZ2	MSC-AS1
27	Cldn4	Zdbf2	CD24	PLXNA4	MYOG	ADM2
28	Igfbp2	Myh3	HSPB7	KRT19	LMO7	DCC
29	Tcerg1l	Dchs1	TNNT2	IGFBP3	MYOD1	ENPP2
30	Abcb1a	Nefl	MYLPF	COLEC12	ACTN2	TSPYL5
31	Postn	Ldb3	INHBA	ZNF185	SFRP5	UNC13A
32	S1pr1	Fam84a	ADAMTS12	PRKY	F13A1	GATA6
33	Tagln	Sfrp4	NGFR	MYH7	CKM	LAMC2
34	Fn1	Sct	KIF21B	SIM2	NCAM1	TYW3
35	Trp53i11	Gpnmb	MYOD1	ANO1	MYLPF	OLFM2
36	Il6	Aqp5	SCG2	IL17RD	STAC3	SPON2
37	Itgb5	Myl1	L1CAM	CECR1	NEB	TUBB
38	Fam132b	Mstn	CKB	MYLPF	KLHL31	KLF4
39	Pde1b	H19	F3	TTTY15	ITGA7	BAALC
40	Scg2	Actn2	SFRP1	ACTN2	ERBB3	DPP4
41	Itga1	Mybpc1	COL4A1	ACTA1	GATM	LURAP1L
42	Cd248	Btc	MYH7	F2RL1	MYL4	TGM2
43	Cd28	Lmod3	ADAM12	IL13RA2	MYPN	JRK
44	Slit2	Myl4	ALDH3A1	HOXC10	B3GALT2	LGR4
45	Pappa	Ppfia4	ACTC1	KIAA1462	FGFR4	MCTP2
46	Gucy1a2	Srl	CCDC141	MYBPH	SHISA9	GJB2
47	Stc1	Mylk4	MYH8	CASQ2	NNAT	CXCL8
48	Klf9	Pdlim3	COL5A1	FLG	SORBS1	CPE
49	Tnfaip2	Nrep	ADAMTS2	ANKRD1	NPY	PCDH1
50	Mrc2	Acta1	MYOG	SLIT2	COL25A1	TBX2

Appendix Table S1. Top 50 genes responsible for variations of PC1 and 2 in the principal component analysis shown in Fig 2A.

50 top upregulated genes in mMuSCs	50 top up genes in human myoblasts	50 top upregulated genes in hiMPs
Gpr125, ENSMUSG00000029090	HEY1, ENSG00000164683	MBP, ENSG00000197971
Slc2a3, ENSMUSG00000003153	TMEM26, ENSG00000196932	FAM155A, ENSG00000204442
Adra2a, ENSMUSG00000033717	**ADAMTS14, ENSG00000138316**	RNF152, ENSG00000176641
D630003M21Rik, ENSMUSG00000037813	COL5A3, ENSG00000080573	**ADAMTS14, ENSG00000138316**
Ptpn1, ENSMUSG00000027540	TENM2, ENSG00000145934	WNT5A, ENSG00000114251
Mmp14, ENSMUSG00000000957	ACP5, ENSG00000102575	HES4, ENSG00000188290
Sfrp4, ENSMUSG00000021319	KIF21B, ENSG00000116852	BNC1, ENSG00000169594
C1qtnf6, ENSMUSG00000022440	SBSN, ENSG00000189001	ABCA1, ENSG00000165029
Rasa2, ENSMUSG00000032413	**IL6, ENSG00000136244**	AFAP1L2, ENSG00000169129
Sepn1, ENSMUSG00000050989	SOD3, ENSG00000109610	S1PR3, ENSG00000213694
Plat, ENSMUSG00000031538	KCNE4, ENSG00000152049	STX1A, ENSG00000106089
Pofut2, ENSMUSG00000020260	SPHK1, ENSG00000176170	FAM43A, ENSG00000185112
Fzd8, ENSMUSG00000036904	COL27A1, ENSG00000196739	TSKU, ENSG00000182704
4833422C13Rik, ENSMUSG00000074782	POSTN, ENSG00000133110	NR2F1-AS1, ENSG00000237187
Il1rap, ENSMUSG00000022514	DHCR24, ENSG00000116133	**ADAMTS15, ENSG00000166106**
Lrig1, ENSMUSG00000030029	PLEKHG4B, ENSG00000153404	DTX1, ENSG00000135144
Acaa2, ENSMUSG00000036880	OLFM2, ENSG00000105088	RUNX3, ENSG00000020633
Tspan15, ENSMUSG00000037031	**NGF, ENSG00000134259**	KLF7, ENSG00000118263
Hey1, ENSMUSG00000040289	**NR2F1-AS1, ENSG00000237187**	**NGF, ENSG00000134259**
Stard3nl, ENSMUSG00000003062	NOTCH3, ENSG00000074181	C15orf39, ENSG00000167173
Arhgap42, ENSMUSG00000050730	DKK3, ENSG00000050165	M6PR, ENSG00000003056
Pde1b, ENSMUSG00000022489	EMILIN2, ENSG00000132205	KCTD15, ENSG00000153885
Fchsd2, ENSMUSG00000030691	ALPL, ENSG00000162551	BMP1, ENSG00000168487
Pla1a, ENSMUSG00000002847	EBF1, ENSG00000164330	**IL6, ENSG00000136244**
Fn1, ENSMUSG00000026193	IL1R1, ENSG00000115594	PWP1, ENSG00000136045
Pkp1, ENSMUSG00000026413	RAB31, ENSG00000168461	ITPRIPL2, ENSG00000205730
Col5a1, ENSMUSG00000026837	SAT1, ENSG00000130066	LHFPL2, ENSG00000145685
A430105I19Rik, ENSMUSG00000045838	PRSS12, ENSG00000164099	FCRLB, ENSG00000162746
Fam132b, ENSMUSG00000047443	LRRC8E, ENSG00000171017	KLF9, ENSG00000119138
Nrarp, ENSMUSG00000078202	LGR4, ENSG00000205213	AAED1, ENSG00000158122
Fam46a, ENSMUSG00000032265	SLC39A14, ENSG00000104635	CPQ, ENSG00000104324
Col5a3, ENSMUSG00000004098	PPARGC1B, ENSG00000155846	SLC39A6, ENSG00000141424
Cald1, ENSMUSG00000029761	KLHL5, ENSG00000109790	RAI14, ENSG00000039560
Ptprn, ENSMUSG00000026204	DNM1, ENSG00000106976	IL1RAP, ENSG00000196083
Rhoj, ENSMUSG00000046768	GRPR, ENSG00000126010	ACP5, ENSG00000102575
Syt5, ENSMUSG00000004961	**SPP1, ENSG00000118785**	ANGPT2, ENSG00000091879
Heyl, ENSMUSG00000032744	AEN, ENSG00000181026	GJA4, ENSG00000187513
Adamts2, ENSMUSG00000036545	BASP1, ENSG00000176788	TCIRG1, ENSG00000110719
Abca8a, ENSMUSG00000041828	MID2, ENSG00000080561	PNP, ENSG00000198805
Fap, ENSMUSG00000000392	GBP1, ENSG00000117228	TM4SF19, ENSG00000145107
Ahr, ENSMUSG00000019256	STARD4, ENSG00000164211	DUSP5, ENSG00000138166
Dnajc10, ENSMUSG00000027006	FGF1, ENSG00000113578	SMAGP, ENSG00000170545
Epdr1, ENSMUSG00000002808	CNN1, ENSG00000130176	ZPR1, ENSG00000109917
Fzd4, ENSMUSG00000049791	ETV6, ENSG00000139083	RPP25, ENSG00000178718
Wisp2, ENSMUSG00000027656	CARMN, ENSG00000249669	PITPNC1, ENSG00000154217
Fbn1, ENSMUSG00000027204	LGMN, ENSG00000100600	ITGB3, ENSG00000259207
Grem1, ENSMUSG00000074934	INHBA, ENSG00000122641	JAK2, ENSG00000096968
*Cfh ENSMUSG00000026365	IL11, ENSG00000095752	EPAS1, ENSG00000116016
*AW011738, ENSMUSG00000078349	BEX1, ENSG00000133169	**SPP1, ENSG00000118785**
*Hey2, ENSMUSG00000019789	UBASH3B, ENSG00000154127	AC002480.2, ENSG00000232759
**50 top downregulated genes in mMuSCs**	**50 top downregulated genes in human myoblasts**	**50 top downregulated genes in hiMPs**
Fam135a, ENSMUSG00000026153	SOST, ENSG00000167941	TLE4, ENSG00000106829,,
Ripk4, ENSMUSG00000005251	CARNS1, ENSG00000172508	THRB, ENSG00000151090
Spna1, ENSMUSG00000026532	NEB, ENSG00000183091	FIGN, ENSG00000182263
Nptx1, ENSMUSG00000025582	OSBP2, ENSG00000184792	REEP2, ENSG00000132563
Arhgdig, ENSMUSG00000073433	LTK, ENSG00000062524	CRIP1, ENSG00000213145
Pepd, ENSMUSG00000063931	C2orf88, ENSG00000187699	PYGM, ENSG00000068976
Purb, ENSMUSG00000091207	STMN3, ENSG00000197457	LRRN1, ENSG00000175928
Prrg4, ENSMUSG00000027171	ELAVL2, ENSG00000107105	KLF5, ENSG00000102554
Slc6a13, ENSMUSG00000030108	F2R, ENSG00000181104	PROSER2, ENSG00000148426
Gpr115, ENSMUSG00000023918	CRMP1, ENSG00000072832	PKP2, ENSG00000057294
Gse1, ENSMUSG00000031822	ADGRB1, ENSG00000181790	RIPOR2, ENSG00000111913
Gpr111, ENSMUSG00000057899	ATP2A3, ENSG00000074370	FAM20A, ENSG00000108950
Slc16a1, ENSMUSG00000032902	PTGS1, ENSG00000095303	SLFN13, ENSG00000154760
Popdc3, ENSMUSG00000019848	TIMP3, ENSG00000100234	C8orf46, ENSG00000169085
Wnt10b, ENSMUSG00000022996	SLC48A1, ENSG00000211584	EML4, ENSG00000143924
Pxn, ENSMUSG00000029528	ZFHX4, ENSG00000091656	AQP3, ENSG00000165272
Lpar1, ENSMUSG00000038668	TNRC6B, ENSG00000100354	PEG10, ENSG00000242265
Orai2, ENSMUSG00000039747	PHC3, ENSG00000173889	SLC38A4, ENSG00000139209
Sp7, ENSMUSG00000060284	LAMA3, ENSG00000053747	TMEM56, ENSG00000152078
Igfbp2, ENSMUSG00000039323	ALDH3A2, ENSG00000072210	HMGB2, ENSG00000164104
Fgf11, ENSMUSG00000042826	FABP3, ENSG00000121769	PLEKHA6, ENSG00000143850
Dnajc21, ENSMUSG00000044224	KIF7, ENSG00000166813	SELENOP, ENSG00000250722
Hip1r, ENSMUSG00000000915	ANKRD12, ENSG00000101745	DEPDC7, ENSG00000121690
Lgals3, ENSMUSG00000050335	ZXDB, ENSG00000198455	NAV2, ENSG00000166833
Dll1, ENSMUSG00000014773	MEGF8, ENSG00000105429	SNTB1, ENSG00000172164
Tmem55a, ENSMUSG00000028221	CRLF1, ENSG00000006016	RARRES2, ENSG00000106538
Cdc42ep3, ENSMUSG00000036533	MACF1, ENSG00000127603	MYPN, ENSG00000138347
Gm1078, ENSMUSG00000085272	NAV3, ENSG00000067798	NUP210, ENSG00000132182
Pik3c2b, ENSMUSG00000026447	NAALADL2, ENSG00000177694	EPHA7, ENSG00000135333
Tox2, ENSMUSG00000074607	PARM1, ENSG00000169116	ITGB6, ENSG00000115221
Smarcd3, ENSMUSG00000028949	DAG1, ENSG00000173402	MYO5B, ENSG00000167306
Mamstr, ENSMUSG00000042918	ZBTB20, ENSG00000181722	DCN, ENSG00000011465
Jag2, ENSMUSG00000002799	LRRC8D, ENSG00000171492	CD200, ENSG00000091972
Sct, ENSMUSG00000038580	CASP3, ENSG00000164305	BCL2L11, ENSG00000153094
Dok7, ENSMUSG00000044716	SFRP4, ENSG00000106483	MAP1LC3C, ENSG00000197769
Vasp, ENSMUSG00000030403	HLF, ENSG00000108924	SHD, ENSG00000105251
Sap30, ENSMUSG00000031609	NFIB, ENSG00000147862	ITGA7, ENSG00000135424
Ccdc88c, ENSMUSG00000021182	DCHS1, ENSG00000166341	LMO3, ENSG00000048540
Smad1, ENSMUSG00000031681	GPR153, ENSG00000158292	CCDC85A, ENSG00000055813
Apln, ENSMUSG00000037010	LRP5, ENSG00000162337	ATOH8, ENSG00000168874
Tjap1, ENSMUSG00000012296	SYNE2, ENSG00000054654	RTL10, ENSG00000215012
Sh3glb1, ENSMUSG00000037062	MEF2C, ENSG00000081189	FZD1, ENSG00000157240
Ppm1e, ENSMUSG00000046442	CAVIN4, ENSG00000170681	FAT4, ENSG00000196159
Mycl1, ENSMUSG00000028654	KANK1, ENSG00000107104	MYH10, ENSG00000133026
Tbc1d10b, ENSMUSG00000042492	EHD1, ENSG00000110047	CEBPD, ENSG00000221869
2310002L13Rik, ENSMUSG00000024512	ABHD2, ENSG00000140526	FOXO1, ENSG00000150907
Stard7, ENSMUSG00000027367	HCN2, ENSG00000099822	OLFML2A, ENSG00000185585
Dlk2, ENSMUSG00000047428	TTN, ENSG00000155657	FAT2, ENSG00000086570
Lrp4, ENSMUSG00000027253	KMT2C, ENSG00000055609	SPOCK2, ENSG00000107742
Mum1l1, ENSMUSG00000042515	SGMS2, ENSG00000164023	IMPA2, ENSG00000141401

Appendix Table S2. Full list of genes of heatmaps shown in Appendix Fig S1 displaying 50 genes which exhibit either the greatest up- or down-regulation upon treatment with DLL4 & PDGF-BB in mMuSC (left), human myoblasts (centre) and hiMPs (right). *Genes not shown in heatmap due to N/A rows resulting from Stemformatics analysis. Bold font: common genes in human lists.

Ranked genes							
**Top 50 upregulated genes of treated mMuSC in human myoblasts**		**Top 50 downregulated genes of treated mMuSC in human myoblasts**		**Top 50 upregulated genes of treated mMuSC in hiMPs**		**Top 50 downregulated genes of treated mMuSC in hiMPs**	
Gene	Probe	Gene	Probe	Gene	Probe	Gene	Probe
AHR	ENSG00000106546	POPDC3	ENSG00000132429	ADRA2A	ENSG00000150594	CCDC88C	ENSG00000015133
FZD4	ENSG00000174804	SH3GLB1	ENSG00000097033	ADGRA3	ENSG00000152990	LPAR1	ENSG00000198121
DNAJC10	ENSG00000077232	SLC6A13	ENSG00000010379	PLA1A	ENSG00000144837	MYCL	ENSG00000116990
NRARP	ENSG00000198435	PURB	ENSG00000146676	ACAA2	ENSG00000167315	SBK3	ENSG00000231274
SYT5	ENSG00000129990	LGALS3	ENSG00000131981	PTPRN	ENSG00000054356	MAMSTR	ENSG00000176909
PDE1B	ENSG00000123360	SCT	ENSG00000070031	DNAJC10	ENSG00000077232	DLL1	ENSG00000198719
RHOJ	ENSG00000126785	SMARCD3	ENSG00000082014	MMP14	ENSG00000157227	SMARCD3	ENSG00000082014
PLAT	ENSG00000104368	PPM1E	ENSG00000175175	CALD1	ENSG00000122786	SMAD1	ENSG00000170365
HEY2	ENSG00000135547	PIK3C2B	ENSG00000133056	STARD3NL	ENSG00000010270	DOK7	ENSG00000175920
FZD8	ENSG00000177283	DLK2	ENSG00000171462	AHR	ENSG00000106546	JAG2	ENSG00000184916
PKP1	ENSG00000081277	APLN	ENSG00000171388	TSPAN15	ENSG00000099282	SAP30	ENSG00000164105
ARHGAP42	ENSG00000165895	DOK7	ENSG00000175920	COL5A1	ENSG00000130635	APLN	ENSG00000171388
STARD3NL	ENSG00000010270	GSE1	ENSG00000131149	FN1	ENSG00000115414	PIK3C2B	ENSG00000133056
COL5A3	ENSG00000080573	SP7	ENSG00000170374	IL1RAP	ENSG00000196083	SCT	ENSG00000070031
HEY1	ENSG00000164683	MYCL	ENSG00000116990	RASA2	ENSG00000155903	GSE1	ENSG00000131149
GREM1	ENSG00000166923	JAG2	ENSG00000184916	RHOJ	ENSG00000126785	TJAP1	ENSG00000137221
PTPN1	ENSG00000196396	TJAP1	ENSG00000137221	SYT5	ENSG00000129990	FGF11	ENSG00000161958
IL1RAP	ENSG00000196083	DLL1	ENSG00000198719	FZD8	ENSG00000177283	PPM1E	ENSG00000175175
MMP14	ENSG00000157227	SAP30	ENSG00000164105	PDE1B	ENSG00000123360	SLC6A13	ENSG00000010379
POFUT2	ENSG00000186866	SLC16A1	ENSG00000155380	HEY2	ENSG00000135547	STARD7	ENSG00000084090
C1QTNF6	ENSG00000133466	FGF11	ENSG00000161958	NRARP	ENSG00000198435	PURB	ENSG00000146676
CALD1	ENSG00000122786	IGFBP2	ENSG00000115457	COL5A3	ENSG00000080573	PRRG4	ENSG00000135378
ADAMTS2	ENSG00000087116	SMAD1	ENSG00000170365	ADAMTS2	ENSG00000087116	SP7	ENSG00000170374
COL5A1	ENSG00000130635	CCDC88C	ENSG00000015133	FAP	ENSG00000078098	SLC16A1	ENSG00000155380
PTPRN	ENSG00000054356	CDC42EP3	ENSG00000163171	PKP1	ENSG00000081277	ORAI2	ENSG00000160991
TSPAN15	ENSG00000099282	WNT10B	ENSG00000169884	FCHSD2	ENSG00000137478	DNAJC21	ENSG00000168724
ADRA2A	ENSG00000150594	NPTX1	ENSG00000171246	HEYL	ENSG00000163909	PEPD	ENSG00000124299
ACAA2	ENSG00000167315	LPAR1	ENSG00000198121	LRIG1	ENSG00000144749	IGFBP2	ENSG00000115457
LRIG1	ENSG00000144749	PRRG4	ENSG00000135378	HEY1	ENSG00000164683	ADGRF4	ENSG00000153294
ADGRA3	ENSG00000152990	PXN	ENSG00000089159	PLAT	ENSG00000104368	ADGRF2	ENSG00000164393
EPDR1	ENSG00000086289	MAMSTR	ENSG00000176909	ARHGAP42	ENSG00000165895	CDC42EP3	ENSG00000163171
SFRP4	ENSG00000106483	STARD7	ENSG00000084090	FZD4	ENSG00000174804	WNT10B	ENSG00000169884
PLA1A	ENSG00000144837	HIP1R	ENSG00000130787	PTPN1	ENSG00000196396	ARHGDIG	ENSG00000242173
FCHSD2	ENSG00000137478	PEPD	ENSG00000124299	GREM1	ENSG00000166923	PXN	ENSG00000089159
RASA2	ENSG00000155903	TOX2	ENSG00000124191	C1QTNF6	ENSG00000133466	DLK2	ENSG00000171462
FN1	ENSG00000115414	DNAJC21	ENSG00000168724	POFUT2	ENSG00000186866	POPDC3	ENSG00000132429
FBN1	ENSG00000166147	TBC1D10B	ENSG00000169221	EPDR1	ENSG00000086289	LRP4	ENSG00000134569
FAP	ENSG00000078098	SBK3	ENSG00000231274	SFRP4	ENSG00000106483	SH3GLB1	ENSG00000097033
HEYL	ENSG00000163909	ADGRF2	ENSG00000164393	FBN1	ENSG00000166147	TOX2	ENSG00000124191
Fam132b	*	ADGRF4	ENSG00000153294	Fam132b	*	RIPK4	ENSG00000183421
4833422C13Rik	*	SPTA1	ENSG00000163554	4833422C13Rik	*	VASP	ENSG00000125753
Wisp2	*	FAM135A	ENSG00000082269	Wisp2	*	TBC1D10B	ENSG00000169221
A430105I19Rik	*	ARHGDIG	ENSG00000242173	A430105I19Rik	*	FAM135A	ENSG00000082269
Slc2a3	*	ORAI2	ENSG00000160991	Slc2a3	*	HIP1R	ENSG00000130787
Abca8a	*	RIPK4	ENSG00000183421	Abca8a	*	LGALS3	ENSG00000131981
Cfh	*	VASP	ENSG00000125753	Cfh	*	SPTA1	ENSG00000163554
Fam46a	*	LRP4	ENSG00000134569	Fam46a	*	NPTX1	ENSG00000171246
Sepn1	*	Tmem55a	*	Sepn1	*	Tmem55a	*
AW011738	*	Mum1l1	*	AW011738	*	Mum1l1	*
D630003M21Rik	*	Dynap	*	D630003M21Rik	*	Dynap	*

Appendix Table S3. Table of ranked genes supplementing heatmaps presented in Appendix Fig S2: "Top 50 upregulated genes of treated mMuSC-derived myoblasts in human myoblasts" (Appendix Fig S2A) (left); "Top 50 downregulated genes of treated mMuSC-derived myoblasts in human myoblasts" (Appendix Fig S2A) (right); "Top 50 upregulated genes of treated mMuSC-derived myoblasts in hiMPs" (Appendix Fig S2B) (left); "Top 50 downregulated genes of treated mMuSC-derived myoblasts in hiMPs" (Appendix Fig S2B) (right). *no human orthologue found.

Ranked genes					
**Regulation of cell morphology**		**Proliferation of stem/myogenic Cells**		**Leukocyte trans-endothelial migration**	
Gene	Probe	Gene	Probe	Gene	Probe
MYO10	ENSG00000145555	MMP9	ENSG00000100985	TXK	ENSG00000074966
VEGFA	ENSG00000112715	PTGIR	ENSG00000160013	ACTN3	ENSG00000248746
RHOQ	ENSG00000119729	VEGFA	ENSG00000112715	VAV3	ENSG00000134215
FN1	ENSG00000115414	PDGFRB	ENSG00000113721	CLDN5	ENSG00000184113
KIT	ENSG00000157404	NGF	ENSG00000134259	ITGB2	ENSG00000160255
RAC3	ENSG00000169750	IRAK1	ENSG00000184216	RASSF5	ENSG00000266094
WIPF1	ENSG00000115935	TGFB1	ENSG00000105329	ACTN2	ENSG00000077522
SH3KBP1	ENSG00000147010	C3AR1	ENSG00000171860	JAM2	ENSG00000154721
RHOC	ENSG00000155366	KITLG	ENSG00000049130	PIK3R1	ENSG00000145675
HEXB	ENSG00000049860	GNAI3	ENSG00000065135	JAM3	ENSG00000166086
CDC42EP4	ENSG00000179604	SIRT6	ENSG00000077463	MYLPF	ENSG00000180209
RAC1	ENSG00000136238	JAK2	ENSG00000096968	CTNNA3	ENSG00000183230
MSN	ENSG00000147065	ITGB3	ENSG00000259207	GNAI1	ENSG00000127955
PLXND1	ENSG00000004399	SNAI2	ENSG00000019549	CXCL12	ENSG00000107562
IL6	ENSG00000136244	IL6	ENSG00000136244	MYL2	ENSG00000111245
FMNL3	ENSG00000161791	NOTCH3	ENSG00000074181	MMP9	ENSG00000100985
MYH9	ENSG00000100345	MYC	ENSG00000136997	PLCG2	ENSG00000197943
KIF3A	ENSG00000131437	NOS3	ENSG00000164867	CLDN7	ENSG00000181885
FBLIM1	ENSG00000162458	ILK	ENSG00000166333	ESAM	ENSG00000149564
CDC42EP1	ENSG00000128283	TRIB1	ENSG00000173334	ARHGAP35	ENSG00000160007
DLC1	ENSG00000164741	IL12A	ENSG00000168811	RHOH	ENSG00000168421
ARHGAP35	ENSG00000160007	HBEGF	ENSG00000113070	ACTN1	ENSG00000072110
ARAP3	ENSG00000120318	CAV1	ENSG00000105974	ITGB1	ENSG00000150093
RHOG	ENSG00000177105	CNN1	ENSG00000130176	VASP	ENSG00000125753
RHOD	ENSG00000173156	FGF9	ENSG00000102678	THY1	ENSG00000154096
SEMA4D	ENSG00000187764	BMPR1A	ENSG00000107779	RAP1B	ENSG00000127314
LPAR1	ENSG00000198121	HMGB2	ENSG00000164104	RAC1	ENSG00000136238
MYH10	ENSG00000133026	BMP4	ENSG00000125378	GNAI3	ENSG00000065135
RHOBTB3	ENSG00000164292	RBPJ	ENSG00000168214	MSN	ENSG00000147065
PHIP	ENSG00000146247	CTNNBIP1	ENSG00000178585		
S100A13	ENSG00000189171	SOX15	ENSG00000129194		
PALMD	ENSG00000099260	MYOD1	ENSG00000129152		
ITGA7	ENSG00000135424	MAP3K5	ENSG00000197442		
PALM2	ENSG00000243444	PIK3R1	ENSG00000145675		
EPB41L3	ENSG00000082397	MYOG	ENSG00000122180		
WIPF3	ENSG00000122574	KLHL41	ENSG00000239474		
SEMA3E	ENSG00000170381	SMARCD3	ENSG00000082014		
KDR	ENSG00000128052	MAGI1	ENSG00000151276		
PLXNB1	ENSG00000164050	CAMK2D	ENSG00000145349		
		MEF2C	ENSG00000081189		
		MEGF10	ENSG00000145794		
		PPARGC1A	ENSG00000109819		
		PDE1A	ENSG00000115252		
		ANGPT1	ENSG00000154188		
		MMP2	ENSG00000087245		
		RGS5	ENSG00000143248		
		IL18	ENSG00000150782		
		PDGFD	ENSG00000170962		
		AKR1B1	ENSG00000085662		
		MNAT1	ENSG00000020426		
		SKP2	ENSG00000145604		
		EGR1	ENSG00000120738		
		TGM2	ENSG00000198959		
		DNMT1	ENSG00000130816		
		ASPM	ENSG00000066279		
		ORC1	ENSG00000085840		

Appendix Table S4. Table of ranked genes accompanying heatmaps “Regulation of cell morphology”, “Proliferation of stem/myogenic cells” and “Leukocyte trans-endothelial migration” (Fig 3A, 3D and 4H, respectively).
