# Supplementary material for: Assessing and enhancing migration of human myogenic progenitors using directed iPS cell differentiation and advanced tissue modelling
Source: EMBO Mol Med. 2022 Sep 26;14(10):e14526. doi: 10.15252/emmm.202114526 (PMC9549733; doi:10.15252/emmm.202114526)
Supplement: Supplementary file 2 — Expanded View Figures PDF [file EMMM-14-e14526-s012.pdf]

Expanded View Figures

Figure EV1. Assessment of purity of hiMP populations.

A Representative immunofluorescence analysis of MYOD (skeletal myogenic lineage marker, green) and PAX6 (early neuroectodermal lineage marker, red) immunoreactivity in three of the four different hiMP lines used in this study.

B Immunofluorescence analysis of MYOD and MAP2 (late neuroectodermal/neuronal marker, red) in the same hiMPs shown in (A).

C Positive controls for the PAX6 and MAP2 staining shown in (A, B); top panel: spontaneously differentiating hiPSCs; bottom panel: hiPSC-derived neurons.

D Bar graph quantifying the percentages of MYOD-positive nuclei within three populations of hiMPs (experimental replicates = 3; error bars: SD). Data information: Scale bars: (A, B) 75 µm; (C) top 100 µm; bottom 20 µm.

Source data are available online for this figure.

 © 2022 The Authors

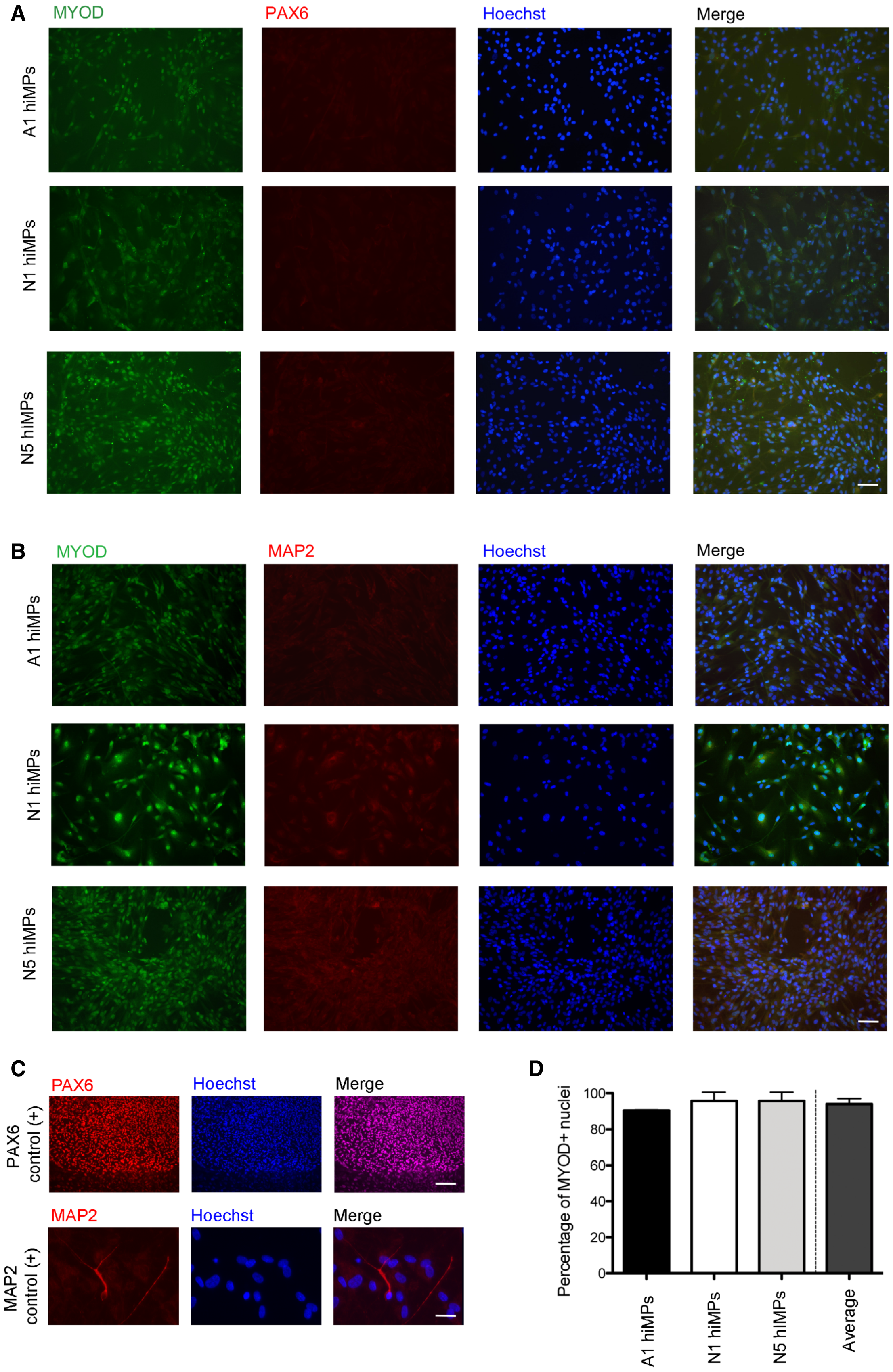
A
MYOD
PAX6
Hoechst
Merge
A1 hiMPs
N1 hiMPs
N5 hiMPs
B
MYOD
MAP2
Hoechst
Merge
A1 hiMPs
N1 hiMPs
N5 hiMPs
C
PAX6
Hoechst
Merge
PAX6 control (+)
MAP2
Hoechst
Merge
MAP2 control (+)
D
Percentage of MYOD+ nuclei
100
80
60
40
20
0
A1 hiMPs
N1 hiMPs
N5 hiMPs
Average

Figure EV1.

Figure EV2. Additional *in vitro* motility and migration analyses of treated and untreated hiMPs.

A Trajectory plots for visualisation of the migratory paths of untreated and treated cells that were exposed to either 1% BSA or DLL4 and PDGF-BB, respectively, over the course of the motility assay. Each line depicts the path of an individual cell.

B Visualisation of the motility state space of untreated and treated hiMPs using t-SNE (perplexity = 35).

C Hierarchical clustering of the first 30 principal components visualised with a t-SNE plot showing two clusters (Silhouette S_i = 0.19).

D Bar charts displaying the normalised motility feature values for comparison between conditions: untreated and DLL4 and PDGF-BB (left), cluster 1 and cluster 2 (right) (experimental replicates = 3; total 412 cells).

E Bar graph showing proportions of untreated and treated hiMPs within the two clusters. Hypothesis testing was performed with a chi-squared (χ^2) test.

F Trajectory plots for visualisation of hiMP migration after 24 h of treatment (top row), or 72 h of treatment (bottom row).

G, H t-SNE plots (perplexity = 35) for visualisation of the motility state space of hiMPs in two-dimensions (left). Cluster assignments after hierarchical clustering (S_i = 0.13 (24 h); S_i = 0.18 (72 h)). (H) Bar plots showing normalised motility features for both 24 h (top row) and 72 h (bottom row) conditions (experimental replicates = 3; total 876 cells and total 478 cells analysed for 24 and 72 h conditions, respectively.).

I Bar graph displaying proportions of untreated and DLL4 and PDGF-BB-treated hiMPs treated for 24 and 72 h. Hypothesis testing was performed with a chi-squared (χ^2) test.

J Bar graphs depict quantification of parameters obtained from single cell tracking analysed using TrackMate. Motility statistics were calculated for untreated (grey bars) and treated (white bars) hiMPs (experimental replicates = 3; error bars: SD). *P* values within figure: *t*-test.

Source data are available online for this figure.

© 2022 The Authors

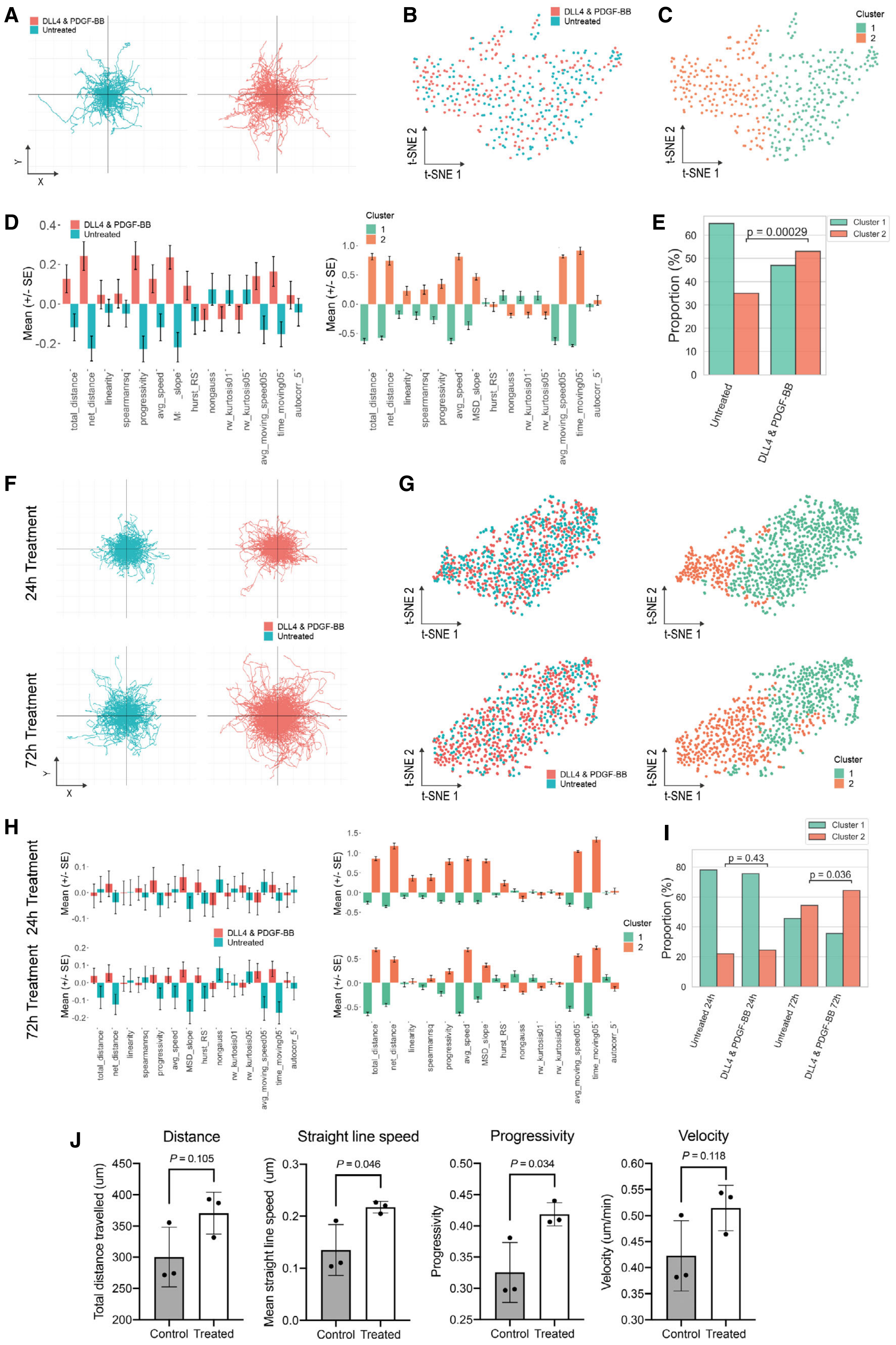
A
DLL4 & PDGF-BB
Untreated
B
C
Cluster
t-SNE 1
t-SNE 2
D
E
Mean (+/- SE)
Proportion (%)
Cluster 1
Cluster 2
p = 0.00029
Untreated
DLL4 & PDGF-BB
F
24h Treatment
72h Treatment
G
H
I
p = 0.43
p = 0.036
Untreated 24h
DLL4 & PDGF-BB 24h
Untreated 72h
DLL4 & PDGF-BB 72h
total_distance
net_distance
linearity
spearmanrsq
progressivity
avg_speed
MSD_slope
hurst_RS
nongauss
rw_kurtosis01
rw_kurtosis05
avg_moving_speed05
time_moving05
autocorr_5
J
Distance
Straight line speed
Progressivity
Velocity
P = 0.105
P = 0.046
P = 0.034
P = 0.118
Total distance travelled (um)
Mean straight line speed (um)
Velocity (um/min)
Control
Treated

Figure EV2.

Figure EV3. Additional *in silico* analyses and assessment of permeability of endothelialised 3D blood vessel-like microfluidic channels.

- A Heatmaps displaying genes that are involved in negative regulation of cellular extravasation (left; GO: 002692) and positive regulation of cellular extravasation (right; GO: 002693). $*P < 0.05$.
- B *P* value-adjusted hierarchical clustering heatmap showing a manually curated list of genes involved in enhanced trans-endothelial migration of cancer cells (*P* set al 0.05).
- C Maximum intensity projection of a microfluidic channel immunostained for CD31 showing cobblestone-like morphology of HUVECs lining the top perfusion channel. Scale bar = 30 μm.
- D Representative fluorescence images of 20 kDa FITC-conjugated dextran (top two rows) and 150 kDa TRITC-conjugated dextran (bottom two rows) added to the top perfusion channel of OrganoPlate® chips with and without 3D endothelial monolayers generated by HUVECs. Chips were imaged every 3 min for 15 min. Scale bar = 100 μm.
- E Bar chart quantifying images shown in (D) using the normalised intensity calculated as the ratio of fluorescence between the ECM channel and top perfusion channel at each time point for cell-free and HUVEC chips containing 20 and 150 kDa fluorescent dextrans (technical replicates = 3; error bars; SD).

Source data are available online for this figure.

© 2022 The Authors

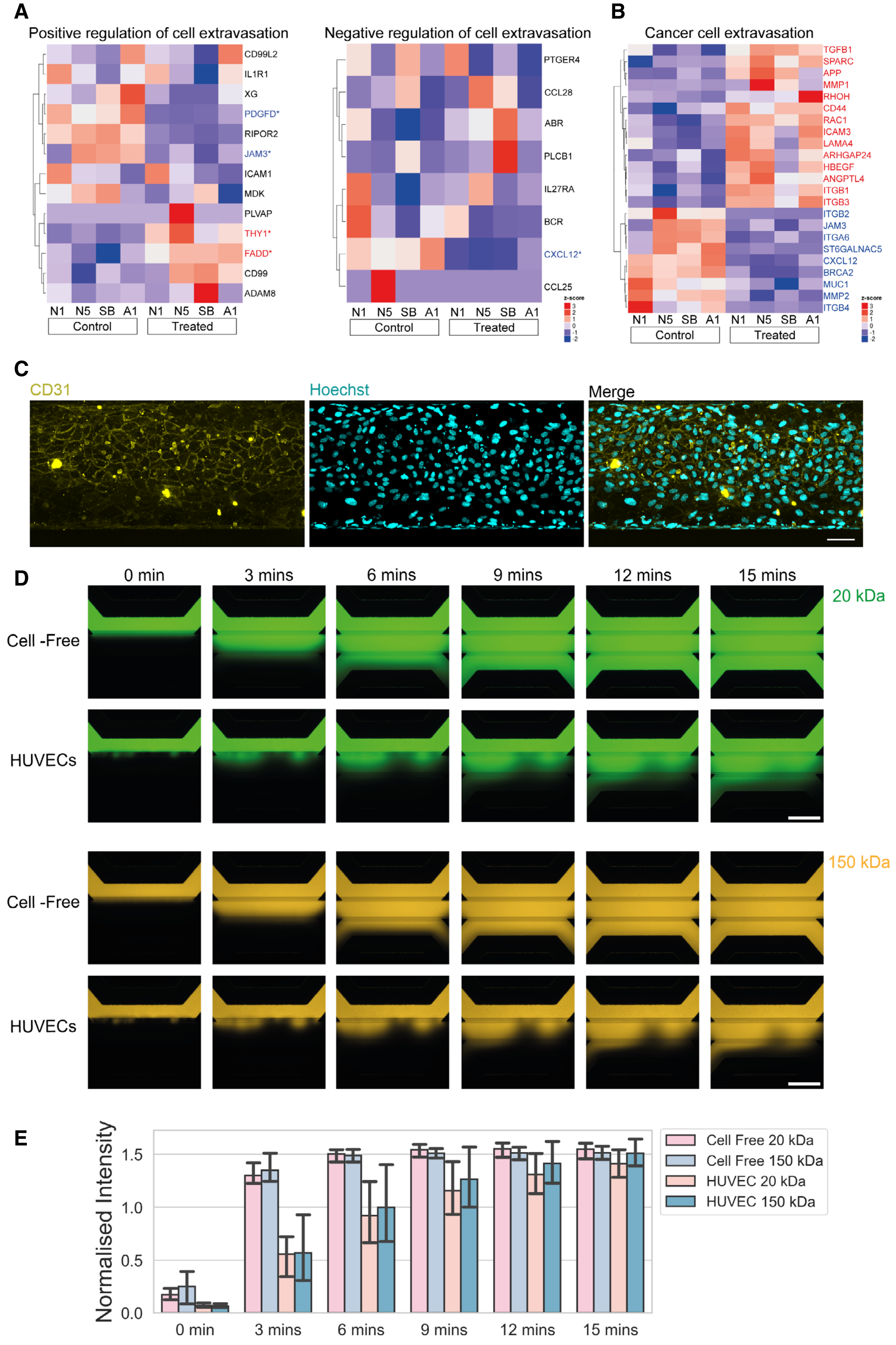
A
Positive regulation of cell extravasation
CD99L2
IL1R1
XG
PDGFD*
RIPOR2
JAM3*
ICAM1
MDK
PLVAP
THY1*
FADD*
CD99
ADAM8
N1 N5 SB A1 N1 N5 SB A1
Control
Treated
Negative regulation of cell extravasation
PTGER4
CCL28
ABR
PLCB1
IL27RA
BCR
CXCL12*
CCL25
N1 N5 SB A1 N1 N5 SB A1
Control
Treated
z-score
B
Cancer cell extravasation
TGFB1
SPARC
APP
MMP1
RHOH
CD44
RAC1
ICAM3
LAMA4
ARHGAP24
HBEGF
ANGPTL4
ITGB1
ITGB3
ITGB2
JAM3
ITGA6
ST6GALNAC5
CXCL12
BRCA2
MUC1
MMP2
ITGB4
N1 N5 SB A1 N1 N5 SB A1
Control
Treated
z-score
C
CD31
Hoechst
Merge
D
0 min
3 mins
6 mins
9 mins
12 mins
15 mins
20 kDa
Cell -Free
HUVECs
150 kDa
Cell -Free
HUVECs
E
Normalised Intensity
0.0
0.5
1.0
1.5
0 min
3 mins
6 mins
9 mins
12 mins
15 mins
Cell Free 20 kDa
Cell Free 150 kDa
HUVEC 20 kDa
HUVEC 150 kDa

Figure EV3.
