# Supplementary material for: Assessing and enhancing migration of human myogenic progenitors using directed iPS cell differentiation and advanced tissue modelling
Source: EMBO Mol Med. 2022 Sep 26;14(10):e14526. doi: 10.15252/emmm.202114526 (PMC9549733; doi:10.15252/emmm.202114526)
Supplement: Supplementary file 4 — Movie EV1 [file EMMM-14-e14526-s005.zip › Movie EV1/Movie EV1 Legend.docx]

**Movie EV1.** Time-lapse imaging of CMFDA-stained untreated hiMPs deposited into the top perfusion channel of the OrganoPlate® chip. hiMPs were imaged at 20 frames/second for 1 minute. Scale bar = 100 μm.
