# Supplementary material for: Assessing and enhancing migration of human myogenic progenitors using directed iPS cell differentiation and advanced tissue modelling
Source: EMBO Mol Med. 2022 Sep 26;14(10):e14526. doi: 10.15252/emmm.202114526 (PMC9549733; doi:10.15252/emmm.202114526)
Supplement: Supplementary file 5 — Movie EV2 [file EMMM-14-e14526-s002.zip › Movie EV2/Movie EV2 Legend.docx]

**Movie EV2.** Live imaging of migrating hiMPs (green) within bioengineered human muscles (gray) over 8 hours. Both hiMP and 3D muscle channels were subject to background subtraction. Scale bar = 100 μm.
