# Supplementary material for: Assessing and enhancing migration of human myogenic progenitors using directed iPS cell differentiation and advanced tissue modelling
Source: EMBO Mol Med. 2022 Sep 26;14(10):e14526. doi: 10.15252/emmm.202114526 (PMC9549733; doi:10.15252/emmm.202114526)
Supplement: Supplementary file 7 — PDF+ [file EMMM-14-e14526-s006.pdf]

Article

SOURCE DATA | TRANSPARENT PROCESS | OPEN ACCESS

EMBO Molecular Medicine

Assessing and enhancing migration of human myogenic progenitors using directed iPS cell differentiation and advanced tissue modelling

SungWoo Choi[1,2,†], Giulia Ferrari[2,†], Louise A Moyle[2], Kirsty Mackinlay[2], Naira Naouar[3], Salma Jalal[1,2], Sara Benedetti[4,5], Christine Wells[6], Francesco Muntoni[5,7] & Francesco Saverio Tedesco[1,2,7,*]

Abstract

Muscle satellite stem cells (MuSCs) are responsible for skeletal muscle growth and regeneration. Despite their differentiation potential, human MuSCs have limited *in vitro* expansion and *in vivo* migration capacity, limiting their use in cell therapies for diseases affecting multiple skeletal muscles. Several protocols have been developed to derive MuSC-like progenitors from human induced pluripotent stem (iPS) cells (hiPSCs) to establish a source of myogenic cells with controllable proliferation and differentiation. However, current hiPSC myogenic derivatives also suffer from limitations of cell migration, ultimately delaying their clinical translation. Here we use a multidisciplinary approach including bioinformatics and tissue engineering to show that DLL4 and PDGF-BB improve migration of hiPSC-derived myogenic progenitors. Transcriptomic analyses demonstrate that this property is conserved across species and multiple hiPSC lines, consistent with results from single cell motility profiling. Treated cells showed enhanced trans-endothelial migration in transwell assays. Finally, increased motility was detected in a novel humanised assay to study cell migration using 3D artificial muscles, harnessing advanced tissue modelling to move hiPSCs closer to future muscle gene and cell therapies.

Keywords cell migration; cell therapy; iPS cells; muscular dystrophy; tissue engineering

Subject Categories Musculoskeletal System; Stem Cells & Regenerative Medicine

DOI 10.15252/emmm.202114526 | Received 8 May 2021 | Revised 19 August 2022 | Accepted 19 August 2022

EMBO Mol Med (2022) 14: e14526

Introduction

Muscle satellite stem cells (MuSCs) reside between the basal lamina and sarcolemma of muscle fibres and are responsible for growth and regeneration of skeletal myofibres. Upon activation, MuSCs give rise to an activated progeny named myoblasts, which then repair and regenerate myofibres (reviewed in Benedetti *et al*, 2013). Myoblasts have been tested in numerous clinical trials for Duchenne muscular dystrophy (DMD), the most common muscular dystrophy of childhood, which severely affects most skeletal muscles and remains incurable (reviewed in Tedesco *et al*, 2010). However, despite promising pre-clinical results in animal models, to date, only myogenic cell therapies of localised muscular dystrophies such as oculopharyngeal muscular dystrophy (OPMD) have reported functional improvements upon myoblast transplantations in patients (Périé *et al*, 2014).

Skeletal myogenic cells have been delivered via the intramuscular or the intravascular route (Tedesco *et al*, 2010). However, the efficacy of both transplantation modalities is impeded by insufficient migration, leading to poor muscle biodistribution of donor cells. Intramuscular injections frequently result in generation of chimeric myofibres often limited to areas adjacent to the needle trajectory, necessitating multiple injections and making this strategy challenging for generalised myopathies such as DMD (Skuk, 2004). On the other hand, intravascular delivery of donor cells via major arteries may facilitate simultaneous targeting of multiple muscle groups. Intra-arterial injections of mesoangioblasts, myogenic cells derived from a subset of muscle perivascular cells, ameliorated muscle pathology and function in pre-clinical models of muscular dystrophy and was also translated into a phase I/IIa clinical trial in five DMD

1 The Francis Crick Institute, London, UK
2 Department of Cell and Developmental Biology, University College London, London, UK
3 Institut de Biologie Paris Seine FR3631, Plateforme de Bioinformatique ARTbio, Sorbonne Université, Paris, France
4 UCL Great Ormond Street Institute of Child Health, University College London, London, UK
5 National Institute for Health Research Great Ormond Street Hospital Biomedical Research Centre, London, UK
6 Centre for Stem Cell Systems, The University of Melbourne, Melbourne, VIC, Australia
7 Dubowitz Neuromuscular Centre, UCL Great Ormond Street Institute of Child Health & Great Ormond Street Hospital for Children, London, UK
*Corresponding author. Tel: +44 02031082383; E-mail: f.s.tedesco@ucl.ac.uk
†These authors contributed equally to this work
‡Present address: Institute of Biomedical Engineering, University of Toronto, Toronto, ON, Canada
§Present address: Department of Physiology, Development and Neuroscience, University of Cambridge, Cambridge, UK

© 2022 The Authors. Published under the terms of the CC BY 4.0 license.

boys (Cossu *et al*, 2016). Although mesoangioblasts are still considered promising candidate cells for systemic delivery owing to their trans-endothelial migration (also known as extravasation) capacity, they possess lower skeletal myogenic and self-renewal capacity than MuSCs, which limits their long-term translational potential. Therefore, an ideal cell type for myogenic cell therapies should possess the migratory capacity of perivascular cells as well as the differentiation and self-renewing potential of MuSCs.

NOTCH signalling plays a pivotal role in cell fate specification during embryonic myogenesis, as well as in post-natal MuSC self-renewal and differentiation (Conboy & Rando, 2002; Schuster-Gossler *et al*, 2007; Bjornson *et al*, 2012; Mourikis & Tajbakhsh, 2014; Baghdadi *et al*, 2018; Verma *et al*, 2018). Canonical NOTCH signalling involves interactions between NOTCH ligands (e.g., Delta-like (DLL) 1, 3, 4 and Jagged (JAG) 1, 2) and receptors (NOTCH 1–4). Perturbation of NOTCH signalling in donor cells has shown context-dependent effects on myogenic cell transplants. Treatment of mouse and human myoblasts with DLL1 and DLL4 did not enhance engraftment in *mdx* mice, a DMD mouse model (Sakai *et al*, 2017). However, DLL1 treatment of canine MuSCs maintained their engraftment potential during *in vitro* expansion (Parker *et al*, 2012). Furthermore, modulation of the DLL1-NOTCH1 axis in both mouse and human mesoangioblasts supported improvement of the dystrophic phenotype after intra-arterial delivery in mice (Quattrocelli *et al*, 2014). Platelet-derived growth factor (PDGF) signalling is another regulator of myogenic cell behaviour. PDGF receptor-β (PDGFR-β) is expressed by cells derived from the mesenchyme (Dellavalle *et al*, 2007; Trojanowska, 2009). PDGF-BB, the putative ligand of PDGFR-β, is expressed by endothelial cells and dystrophic muscle fibres for recruitment of pericytes and MuSCs, respectively (Betsholtz, 2004; Piñol-Jurado *et al*, 2017).

Previous work showed that mouse embryonic myoblasts in close proximity to blood vessels undergo a spontaneous fate shift into pericyte-like cells *in vivo*; this phenomenon was mimicked *in vitro* by treating embryonic myoblasts with DLL4 and PDGF-BB (Cappellari *et al*, 2013). More recently, we showed that modulation of NOTCH and PDGF pathways induces perivascular cell features while enhancing self-renewal and migration in adult mouse and human MuSC-derived myoblasts (Gerli *et al*, 2019). However, the translational potential of primary, tissue-derived MuSCs is hindered by the need to obtain them invasively (i.e. via muscle biopsies), as well as by their limited expansion capacity and premature differentiation *in vitro*, which pose major hurdles to reach the cell number required to treat patients with disorders involving multiple muscles such as DMD (Cossu *et al*, 2016). Induced pluripotent stem cells (iPSCs) offer a solution to bypass these limitations.

Human iPSCs (hiPSCs) are becoming a key source of skeletal myogenic progenitor cells for disease modelling and transplantation studies, owing to their controllable proliferation and differentiation capacity, lack of significant ethical concerns and non-invasive sampling of the starting primary cell population (Loperfido *et al*, 2015). Several protocols are currently available to generate skeletal myogenic derivatives from hiPSCs (reviewed in Selvaraj *et al*, 2019a). Starting from the pioneering studies based upon controlled expression of myogenic regulators to obtain transplantable skeletal myogenic cells from hiPSCs (e.g., Darabi *et al*, 2012; Goudenege *et al*, 2012; Tedesco *et al*, 2012), the field has refined transgene-based protocols to direct hiPSC differentiation into skeletal muscle (e.g., Albini *et al*, 2013; Maffioletti *et al*, 2015; Shoji *et al*, 2016; Selvaraj *et al*, 2019b), whilst also developing genomic-integration-free, small molecule-based methods to derive myogenic cells mimicking embryonic development (e.g., Borchin *et al*, 2013; Caron *et al*, 2016; Chal *et al*, 2016; Hicks *et al*, 2018). However, the focus on perfecting methods to obtain myogenic progenitors resembling self-renewing MuSCs has neglected the critical need to enhance their migration capacity, which is essential to deliver cells to large or multiple muscle districts. Although some attempts have previously been made to deliver iPSC-derived myogenic cells systemically (Tedesco *et al*, 2012; Matthias *et al*, 2015; Incitti *et al*, 2019), no specific methods are currently available to differentiate hiPSCs into myogenic progenitors with enhanced migratory and/or extravasation capacity.

Here we exploited directed hiPSC-differentiation, bioinformatics and advanced tissue modelling (Jalal *et al*, 2021) to engineer a developmentally-inspired, small-molecule-based, genomic-integration-free strategy to increase motility and trans-endothelial migration of human myogenic progenitor cells via modulation of NOTCH and PDGF signalling. This study (summarised in Fig 1) provides a framework to model, test and enhance migration of human myogenic cells for future cell therapies of muscle diseases.

Results

Combined activation of NOTCH and PDGF signalling pathways induces conserved transcriptional changes in mouse and human tissue- and iPSC-derived myogenic progenitors

We aimed to identify targetable pathways to improve migration of human iPSC-derived myogenic progenitors (hiMPs), and focused on NOTCH and PDGF (Hellström *et al*, 1999; Armulik *et al*, 2011) which have been shown to improve migration in tissue-resident MuSCs (Gerli *et al*, 2019). To identify whether hiMPs respond to activation of the aforementioned pathways, we performed an unbiased assessment of global transcriptomic changes induced by DLL4 and PDGF-BB in wild-type mouse and human primary MuSC-derived myoblasts, alongside hiMPs. Before performing bulk RNA-sequencing (RNAseq) of these cell populations, we first assessed their purity. Four distinct mouse and four distinct human MuSC-derived myoblast populations were isolated and FACS-purified from skeletal muscles of Pax7-nGFP mice and from healthy human muscle biopsies using green fluorescent protein (GFP) and CD56 (NCAM), respectively (Materials and Methods). hiMPs were derived from four distinct, fully-characterised hiPSC lines generated with genomic-integration-free technologies using a validated transgene-free, small molecule-based protocol recapitulating skeletal muscle developmental specification and differentiation *in vitro* (Caron *et al*, 2016; Materials and Methods). Purity of the hiPSC derivatives was assessed by immunostaining for myogenic and other non-myogenic markers. hiMPs were homogeneously positive for the skeletal myogenic determination factor MYOD and lacked contamination from neuroectodermal derivatives (PAX6 and MAP2; Fig EV1). After assessing their purity, the three groups of cells were treated for 7 days with DLL4 and PDGF-BB (Materials and Methods) and then mRNA was extracted from treated and untreated samples for RNAseq. Principal component analysis (PCA) revealed distinct

 © 2022 The Authors

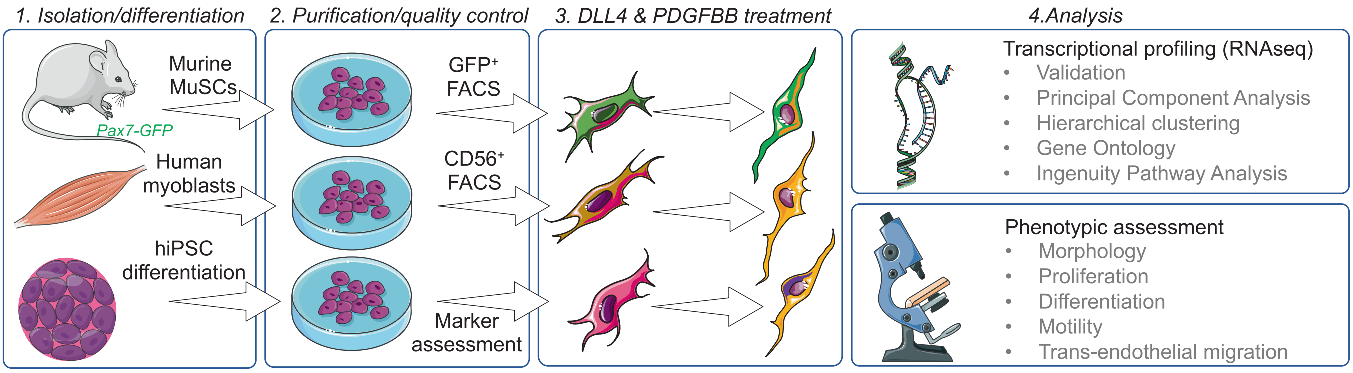

Figure 1. Schematic representation of the cell isolation, culture, treatment, differentiation and analysis pipeline underpinning this study.

Figure created using Servier Medical Art (https://smart.servier.com) in accordance with a Creative Commons Attribution 3.0 Unported Licence (https://creativecommons.org/licenses/by/3.0/).

segregation between DLL4 and PDGF-BB-treated and untreated populations of the 3 cell types (Fig 2A, and Appendix Tables S1 and S2). Additionally, RNAseq analysis provided a total of 1,405, 337 and 2,990 differentially expressed genes between treated mouse MuSC-derived myoblasts, human myoblasts and hiMPs and their untreated controls respectively (Fig 2B). Hierarchical clustering of top 50 differentially regulated genes in mouse and human samples showed overall consistency of transcriptional dynamics in those transcripts across all four lines analysed (Appendix Fig S1 and Table S2).

We then tested whether the observed global transcriptional changes were a consequence of NOTCH and PDGF signalling activation. To address this question, we looked at specific downstream targets of NOTCH and PDGF pathways, as well as key myogenic and perivascular markers known to be modulated by this treatment in murine myoblasts (Cappellari *et al*, 2013; Gerli *et al*, 2019). As shown in Fig 2C, treated mouse MuSCs (mMuSCs), human myoblasts and hiMPs shared similar dynamics of NOTCH targets and perivascular transcripts upregulation, coupled with downregulation of myogenic transcripts such as *Myogenin* and *MyoD* (also a downstream NOTCH signalling target (Kopan *et al*, 1994)). This was further validated via qRT-PCR analysis in two representative hiMP lines (Fig 2D). We subsequently wanted to identify inter-species similarities in transcriptional response to DLL4 and PDGF-BB treatment. For this purpose, we selected the top 50 differentially regulated genes of treated mMuSC-derived myoblasts, found the relative human orthologues and then performed hierarchical clustering on human myoblast and hiMP datasets. The resulting heatmaps show that the majority of transcripts in the treated human cells display a similar regulation in comparison to their murine counterparts (Appendix Fig S2 and Table S3), further indicating an overall conservation of the cellular response to DLL4 and PDGF-BB in skeletal myogenic cells (albeit with some expected variability in human, non-syngeneic cells). Finally, Gene Ontology (GO), Kegg and Reactome enrichment analyses showed shared gene functions amongst the cell groups, including pathways involved in extracellular matrix remodelling, integrin-cell surface interactions, focal adhesion generation, in addition to the expected NOTCH and PDGF pathways (Fig 2E).Together these data demonstrate that DLL4 and PDGF-BB induce transcriptional changes across skeletal myogenic progenitors from different species and developmental origins, with hiMPs showing the greatest transcriptional response.

Analysis of morphology, proliferation and differentiation of DLL4 and PDGFBB-treated hiMPS

To identify whether the transcriptional response of DLL4 and PDGFBB-treated hiMPs results in detectable, cellular phenotypic changes, we assessed specific transcriptional signatures alongside functional readouts such as morphology, proliferation and skeletal myogenic differentiation capacity. Hierarchical clustering analysis highlighted modulation of several regulators of cell morphology such as upregulation of *MYH9*, *MYO10*, *RAC1/3* and *RHOC*, alongside downregulation of *RHOD*, *MYH10*, *ITGA7* and *SEMA3* (Fig 3A; Appendix Table S4). After 1 week of treatment, hiMPs appeared more elongated than their untreated counterpart, in accordance with what was observed in mMuSCs (Gerli *et al*, 2019). Morphometric analysis confirmed this finding, revealing a higher number of cells falling within the first quartile (0–0.25) of cell circularity ratio (i.e., cells with marked protrusions; Fig 3B and C; mean ± SD: treated 45.33 ± 10.26, untreated 12.67 ± 10.60; $P = 0.027$, paired t-test). We next assessed the impact of DLL4 and PDGF-BB treatment on hiMP proliferation. A decrease in the proliferative capacity of myogenic cells could be detrimental for cell therapy, limiting the translational potential of donor cells. Hierarchical clustering analysis highlighted modulation of several regulators of cell proliferation and lineage commitment in at least 3 out of 4 hiMP lines, including upregulation of *PDGFRB*, *NOTCH3*, *VEGFA* and *TGFB1*, alongside downregulation of *CTNNBIP1*, *HMGB2* and the myogenic factors *MEF2C* and *MYOG* (Fig 3D). These transcriptional changes did not impact on the proliferative ability of hiMPs, with treated and untreated cells displaying a comparable cell cycle, as shown by functional EdU incorporation assay (Fig 3E and F; Appendix Table S4).

NOTCH activation inhibits myogenesis *in vitro* in embryonic and adult myoblasts (Kopan *et al*, 1994; Conboy & Rando, 2002; Mourikis & Tajbakhsh, 2014; Gerli *et al*, 2019). Although RNAseq analysis of the NOTCH pathway shows modulation of several effectors

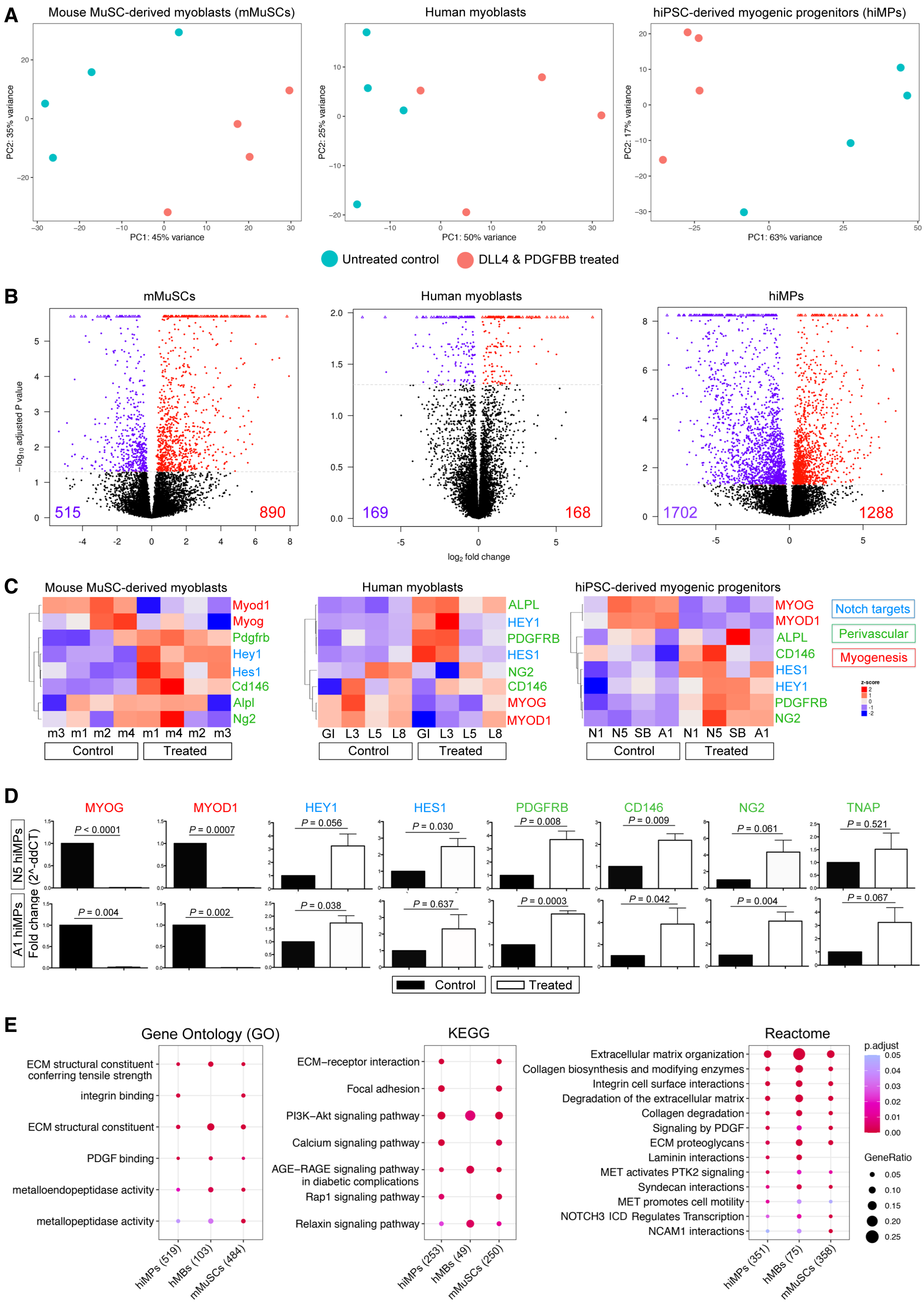

Figure 2.

© 2022 The Authors

Figure 2. RNAseq-based transcriptional profiling of mouse and human myogenic progenitors upon activation of NOTCH and PDGF signalling pathways.

A Principal Component Analysis (PCA) showing mMuSC-derived myoblasts (left), human myoblasts (centre) and hiMPs (right). Four cell lines were analysed by RNAseq in treated and untreated conditions for each cell population. Each point on the PCA represents a cell population. Additional information in Appendix Tables S1 and S2.

B Volcano plots showing differentially expressed genes between untreated and DLL4 and PDGFBB-treated mMuSCs, human myoblasts and hiMPs. Red dots represent genes which display a positive fold-change in expression upon treatment with DLL4 and PDGF-BB whilst violet dots represent genes which are significantly downregulated. Differentially expressed genes required a *P* value of $\leq$ 0.05 to be considered significant.

C Heatmaps showing changes in expression of key myogenic (*MYOGENIN, MYOD1*), perivascular (*PDGFRB*, *CD146*, *NG2*, *ALPL*) and NOTCH target (*HEY1*, *HES1*) genes upon treatment with DLL4 and PDGF-BB in mMuSC-derived myoblasts (left), human myoblasts (middle) and hiMPs (right). Clustering was performed by genes/probes with Pearson correlation. Colour scale based on z-scores: red regions indicate high expression whilst blue regions indicate low expression. Dendrograms indicate the similarity of clusters as well as the orders in which clusters were assembled.

D Validation of RNAseq data of panel (C) by real-time PCR analysis of the same myogenic, perivascular and NOTCH target transcripts in treated and untreated hiMPs (experimental replicates = 3; error bars; SEM). Statistical analysis (paired *t*-test) was performed on ΔCt values whilst graphs show fold change relative to untreated controls.

E Curated dot plot Gene Ontology (GO; left), Kyoto Encyclopaedia of Genes and Genomes (KEGG; centre) and Reactome (right) enrichment analyses showing shared gene functions amongst the cell groups; numbers in brackets: genes analysed with a *P* value threshold set at 0.05; full lists in a dedicated spreadsheet available in Dataset EV1.

Source data are available online for this figure.

(Fig 3G), we wanted to functionally verify the conservation of this phenomenon in hiMPs. To achieve this aim, we induced myogenic differentiation of DLL4 and PDGF-BB-treated cells and observed a significant reduction in the percentage of nuclei within MyHC-positive fibres, from 70.00 ± 0.29 to 43.06 ± 1.20% (Fig 3H and I; $P < 0.0001$; $N = 3$; mean ± SD). A similar reduction in myogenic differentiation was observed when hiMPs were expanded in an alternative medium, albeit with lower pre-treatment differentiation capacity upon long-term expansion (details in Appendix Fig S3). To further validate the NOTCH-dependency of this finding, we blocked NOTCH pathway with the γ-secretase inhibitor L685458, which selectively inhibits γ-secretase-dependent nuclear translocation of the NOTCH Intra-Cellular Domain (NICD). Upon treatment with L685458, the impairment of differentiation was reverted from 43.06 ± 1.20 to 65.59 ± 5.11 (*P* 0.028; Fig 3H and I), thus confirming that hiMP myogenic differentiation potential is NOTCH-dependent and could be restored to pre-treatment levels. Moreover, reversion of differentiation impairment was also noted to take place spontaneously upon removal of DLL4 and PDGF-BB, with increasing myogenic differentiation noticeable from day 3 onwards of removal of the stimuli (Appendix Fig S4). Finally, treated and untreated cells were intramuscularly transplanted in regenerating muscles of immunodeficient mice ($N = 3$) and no statistically significant differences were noted between the two groups, indicating that DLL4 and PDGF-BB treatment does not negatively impact on the myogenic capacity of cells upon transplantation (Fig 3J and K).

Combined DLL4 and PDGF-BB treatment enhances motility of hiMPs

We and others have shown that NOTCH and PDGF pathways play a critical role in regulating developmental fate, regenerative potential and migration of primary, native myogenic cells (Betsholtz, 2004; Cappellari *et al*, 2013; Piñol-Jurado *et al*, 2017; Camps *et al*, 2019; Gerli *et al*, 2019). Of the aforementioned properties, cell migration is of key relevance for cell therapy. To investigate whether DLL4 and PDGF-BB had an effect on cell migration of hiMPs, we analysed the differentially expressed genes in our RNAseq dataset using Ingenuity Pathway Analysis (IPA). Amongst the most significantly modulated cellular functions upon DLL4 and PDGF-BB treatment there was "Cellular Movement", with a total of 578 differentially expressed genes (Table 1). To correlate these transcriptional changes to a phenotypic response, automated single cell tracking of cells exposed to DLL4 and PDGF-BB was performed and track features were extracted using Heteromotility (Kimmel *et al*, 2018; Fig 4B–F; Materials and Methods). Motility assays were performed under conditions of continuous treatment, in which hiMPs were exposed to either 1% BSA (untreated) or DLL4 and PDGF-BB treatment during the duration of the assay (Fig EV2), as well as in conditions of discontinued treatment, where hiMPs were plated on uncoated surfaces without addition of PDGF-BB (Fig 4). For both conditions, single-cell trajectories indicated an increase in motility mediated by the treatment (Figs 4A and EV2A). Visualisation of single-cell motility features with t-SNE plots revealed that both untreated and DLL4 and PDGF-BB-treated hiMPs shared the same motility state space (Figs 4B and EV2B). To identify heterogenous motility phenotypes, unsupervised hierarchical clustering (Ward's method) was performed with the first 30 principal components which captured > 95% variation to obtain two clusters (Fig 4C and EV2C). Cluster 1 was comprised of a less motile population of cells as indicated by lower total distance travelled, average speed and average time spent moving (Figs 4D and E and EV2D). Cluster 2 represents the motile population of cells, demonstrating higher distances travelled, average speeds and proportion of time spent moving. Additionally, cells within cluster 2 performed directed migration as shown by higher progressivity, linearity and mean squared displacement (MSD; Figs 4D and E, and EV2). In both conditions of treatment, a significant increase in the proportion of cells in cluster 2 was observed indicating that states of high motility are maintained for at least 24 h after the treatment was discontinued (Figs 4F and EV2E). Increased proportion of migratory cells were also detected after a shorter course of treatment of 72 h (Fig EV2F–I). Furthermore, analyses performed with Trackmate (Materials and Methods) validated these findings, showing increased trends in distance, straight line speed, progressivity and velocity in treated hiMPs (Fig EV2J).

© 2022 The Authors

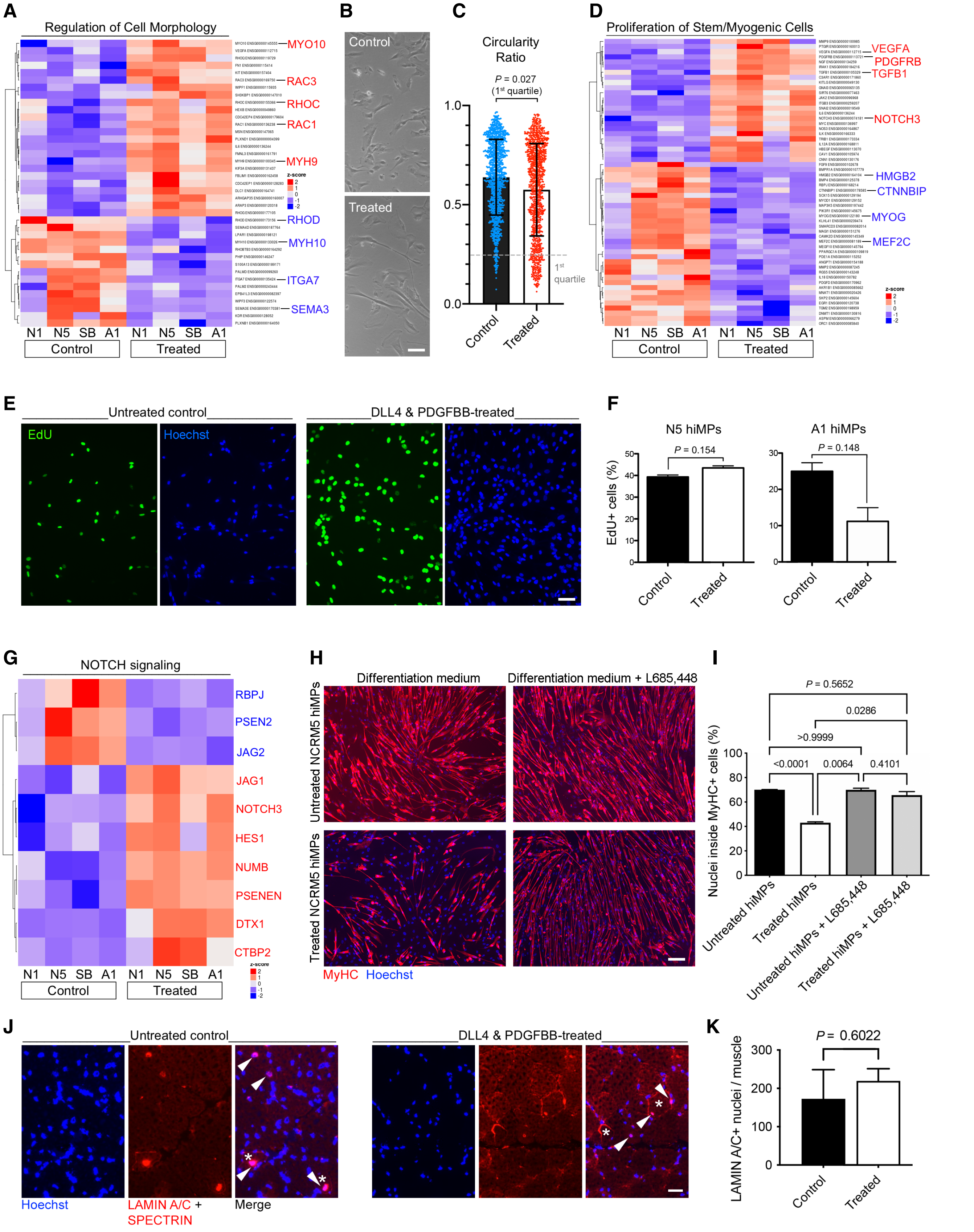

Figure 3.

© 2022 The Authors

◀ **Figure 3. Analysis of morphology, proliferation and differentiation of DLL4 and PDGFBB-treated hiMPs.**

A *P* value-adjusted hierarchical clustering heatmap generated from a gene ontology list of genes involved in regulation of cell morphology (GO 0008360; *P* set at 0.05).
B Phase contrast images displaying morphology of untreated and treated hiMPs. Scale bar: 25 μm.
C Scatter plot showing morphometric analysis of treated and untreated hiMPs. Morphology was quantified using the circularity ratio, where 1 = perfect circle and 0 = line (experimental replicates = 3). Statistical analysis (paired *t*-test) was performed on the first quartile to enhance detection of morphological changes (error bars: SD).
D *P* value-adjusted hierarchical clustering heatmap generated from a gene ontology list of genes involved in proliferation of stem and myogenic cell types (GO 2000291; 0048660; 0014857; 0072091; *P* set at 0.05).
E Immunofluorescence images of untreated and treated hiMPs incubated with EdU for 2 h. Scale bar: 75 μm.
F Bar graphs quantifying EdU experiment shown in (E) (experimental replicates = 3; error bars: SEM). Statistical analysis based on an unpaired *t*-test.
G *P* value-adjusted hierarchical clustering heatmap of NOTCH signalling genes (Kegg pathway 04330).
H Immunofluorescence images of hiMPs expanded in control or treated conditions for 1 week, induced to differentiate for 4 days in the presence or absence of γ-secretase inhibitor L685458 and immunostained for myosin heavy chain (MyHC). Scale bar: 75 μm.
I Bar graph quantifying the average percentage of nuclei within MyHC positive myotubes (experimental replicates = 3; error bars: SEM). Statistical significance based on one-way ANOVA with Tukey's multiple comparison. Scale bar: 75 μm.
J Immunofluorescence panels showing human specific LAMIN A/C+ (nuclei) and SPECTRIN (sarcolemma) staining in tibialis anterior muscles of immunodeficient mice (*N* = 3) transplanted with treated (*n* = 3 muscles) and untreated (*n* = 3 muscles) N5 hiMPs.
K Quantification of LAMIN A/C+ grafted human cells across each muscle. Data information: full gene list for heatmaps in (A) and (D) available in Appendix Table S4.

Source data are available online for this figure.

Table 1. Top cellular and molecular functions associated with DLL4 and PDGF-BB modulation generated via ingenuity pathway analysis (IPA).

IPA cellular and molecular function	*P*-value range	Number of molecules
Cellular assembly and organisation	2.66E-08–4.79E-33	600
Cellular function and maintenance	3.69E-08–4.79E-33	710
Cellular movement	3.38E-08–4.16E-31	578
Cell death and survival	3.77E-08–2.55E-28	814
Cellular development	2.23E-08–7.60E-23	726

Genes upregulated in the DLL4 and PDGF-BB-treated hiMPs relative to the untreated control were subjected to IPA to reveal the predicted most significant associated functions.

To gain further insights on possible protein–protein interaction networks that might positively regulate cell migration, we analysed our RNAseq dataset with the STRING platform (https://string-db.org; Szklarczyk *et al*, 2019; Fig 4G). Functional enrichment analysis highlighted that a number of candidate proteins with relevance in cell migration, which could be associated with the observed migratory phenotype, were upregulated in our datasets, such as TGFB1, ADAMTS2/12/14 and THY1 (Fig 4G; Barker *et al*, 2004; Sciorati *et al*, 2006; Li *et al*, 2020).

Assessment of the effect of DLL4 and PDGF-BB on trans-endothelial migration of hiMPs

Although encouraging, enhanced cellular motility may not be directly relevant in the context of cell therapies requiring intravascular cell delivery to target multiple large muscles. Therefore, we assessed the effect of DLL4 and PDGF-BB treatment on trans-endothelial migration which is essential for systemically delivered cells. Interestingly, RNAseq analysis showed positive modulation of several transcripts involved in cell adhesion and extravasation in treated hiMPs, such as *ESAM*, *ICAM3*, *JAM2*, *MMP9*, *PDGFD* and *THY1*, although some other mediators of extravasation such as *ITGB2* and *CXCL12* were downregulated (Figs 5A, and EV3A and B; Appendix Table S4). Trans-endothelial migration is a multi-step process which starts with cell adhesion to the endothelium under perfusion and then ends with diapedesis in target tissues. To recapitulate the complexity of this process and to functionally assess if DLL4 and PDGF-BB have a role on hiMPs extravasation we first utilised an organ-on-chip system with artificial flow to assess cell–cell adhesion to endothelial cells. Each chip consists of three channels: a top perfusion channel, central extracellular matrix (ECM) channel and bottom perfusion channel (Fig 5B). Flow within channels is introduced using a rocker platform. Within the top perfusion channels, $CD31^+$ 3D blood vessel-like tubules were generated using human-umbilical vein endothelial cells (HUVECs; Figs 5C and D, and EV3C). We first validated that the endothelial channels were functional using a barrier integrity assay, in which fluorescent dextrans of different molecular weights (20 and 150 kDa) were introduced in the top perfusion channel. Quantification of dextran diffusion into the ECM channel indicated that the layer of HUVECs reduced the channel's permeability to dextran molecules (Figs 5E and EV3D). To investigate the adhesive capacity of treated and untreated cells under conditions of flow, hiMPs were delivered through the top perfusion channel and after 15 min the number of fluorescent cells adhering to the endothelial cells was counted, revealing that DLL4 and PDGF-BB had no effect on adhesion efficiency of hiMPs (Figs 5F and G; Movie EV1). Nonetheless, this finding does not rule out an effect of the treatment on trans-endothelial migration which is independent from cell adhesion; however, this hypothesis was difficult to test in the same microfluidic platform, as HUVECs migrated in response to chemoattractants, biasing outcomes of the assay. To overcome this limitation, we assessed trans-endothelial capacity of treated hiMPs using a trans-well migration assay. After 7 days of treatment with DLL4 and PDGF-BB, hiMPs were incubated with a transient fluorescent dye (CFDA, Materials and Methods) and plated onto a monolayer of HUVECs. After 8 h, membranes were fixed and CFDA-positive, trans-migrated cells were quantified. As shown in Fig 5H and I, treatment with DLL4 and

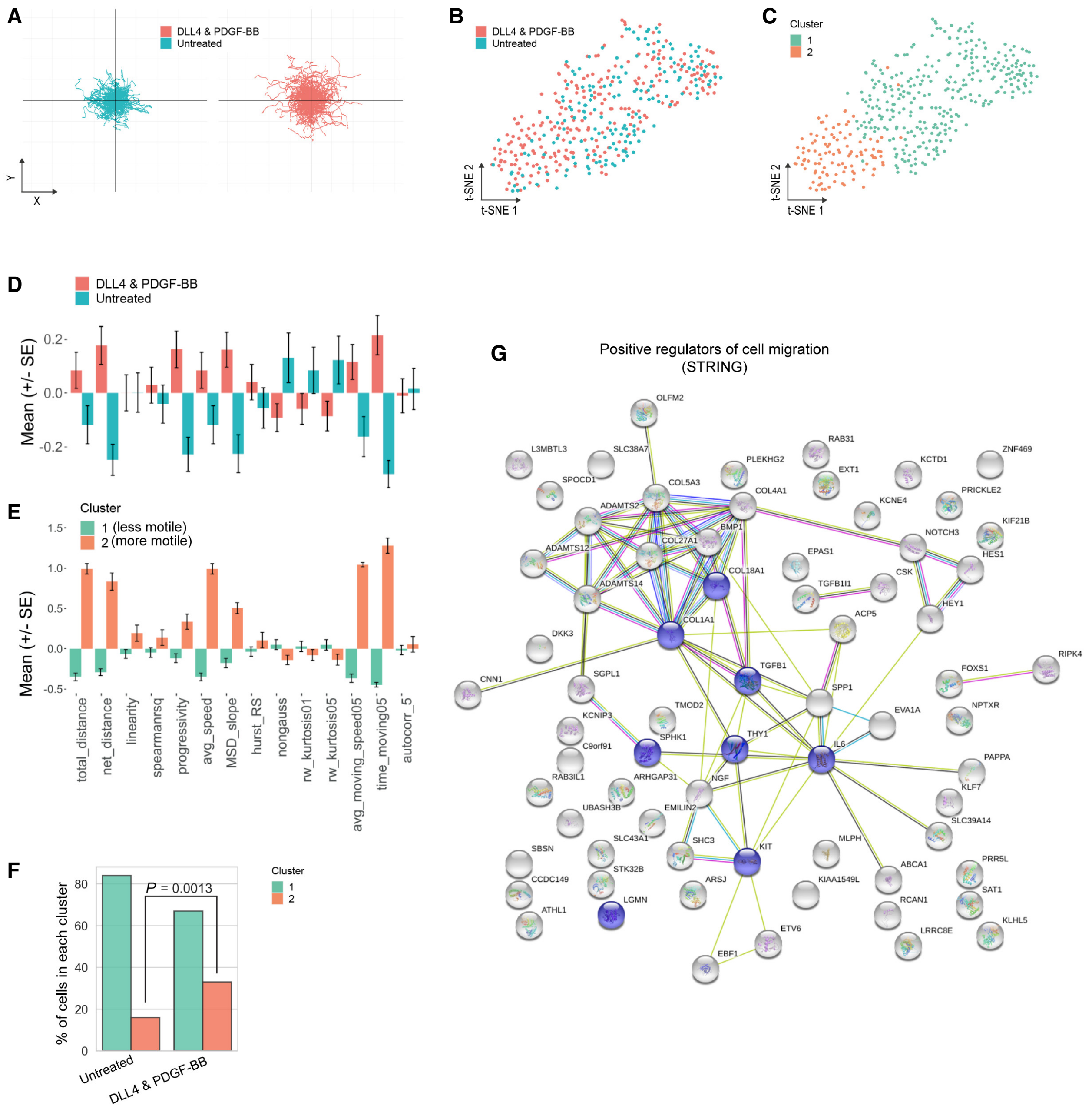

Figure 4. Analysis motile and migratory properties of DLL4 and PDGF-BB treated hiMPs.

A Trajectory plots for visualisation of the migratory paths of treated and untreated cells over the duration of the motility assay. Each line represents the path of a single cell. DLL4 and PDGF-BB treatment was completed prior to motility assessment and all cells were imaged on plastic dishes in absence of the two inducing factors.

B Visualisation of the motility state space of untreated and DLL4 and PDGF-BB-treated hiMPs using t-SNE plots (perplexity = 35).

C Unsupervised hierarchical clustering (Ward's method) visualised with a t-SNE plot showing two clusters (Silhouette S_i = 0.22).

D, E Bar charts demonstrating normalised values for comparison of motility features between conditions (D; untreated and DLL4 and PDGF-BB) and clusters (E) (experimental replicates = 3; total 408 cells; error bars: SEM).

F Bar graph demonstrating proportions of control and DLL4 and PDGF-BB-treated cells within each cluster. Hypothesis testing was performed using the chi-squared (χ^2) test.

G Functional protein association network analysis (https://string-db.org). The network view summarises predicted associations for proteins positively regulating cell migration common to all three datasets. The nodes are proteins and the edges represent the predicted functional associations. Red line: fusion evidence; Green line: neighbourhood evidence; Blue line: co-occurrence evidence; Purple line: experimental evidence; Yellow line: text mining evidence; Light blue line: database evidence; Black line: co-expression evidence. Blue nodes: GO:0030335 positive regulation of cell migration, Count in gene set: 8 of 452, false discovery rate: 0.0156.

Source data are available online for this figure.

 © 2022 The Authors

PDGF-BB significantly enhanced the ability of hiMPs to migrate through an endothelial monolayer (from 0.59 to 3.19 cells/mm^2 (P = 0.0180) and from 0.50 to 20.68 cells/mm^2 (P = 0.0464), in healthy donor-derived hiMPs, respectively). Similar results were obtained with hiMPs derived from a DMD patient and genetically-corrected with a human artificial chromosome containing the entire 2.5 Mb *DYSTROPHIN* genetic locus (DYS-HAC, Materials and Methods; Choi *et al*, 2016), demonstrating that even after genetic correction, hiMPs remain responsive to the DLL4 and PDGF-BB treatment (Fig 5H and I). Overall, these findings suggest that DLL4 and PDGF-

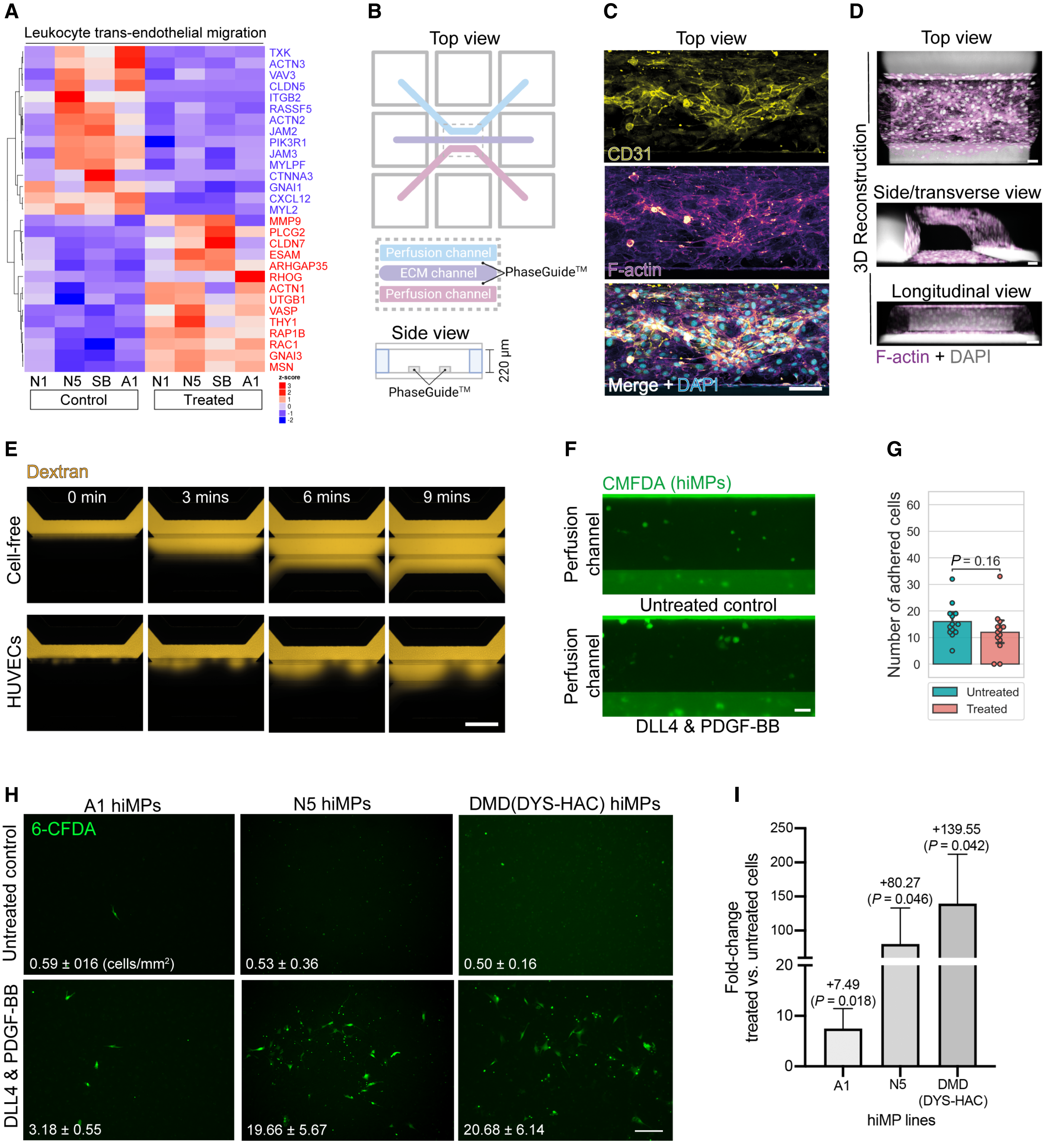

Figure 5.

◀ **Figure 5. Modelling and assessing trans-endothelial migration of hiMPs.**

A *P* value-adjusted hierarchical clustering heatmap displaying hierarchical clustering of genes associated with leukocyte trans-endothelial migration (KEGG pathway: hsa04670; *P* set at 0.05).

B Graphical representation of an individual chip of the OrganoPlate® (produced with BioRender, www.biorender.com). Each chip consists of a top perfusion channel, central ECM channel and bottom perfusion channel. Phase guides between channels allows for generation of surface tension after deposition of collagen-I within the ECM channel so that there is no physical barrier between the collagen gel and perfusion channels. This facilitates generation of a 3D blood vessel that is in direct contact with the ECM channel.

C Maximum intensity projections of the top perfusion channel, 48 h after seeding HUVECs, immunostained for CD31 and F-actin. Scale bar: 100 μm.

D 3D projections of blood vessel-like tubules of the top perfusion channel stained for F-actin. Scale bar: 50 μm.

E Representative fluorescence images of 150 kDa TRITC-conjugated dextran added to the top perfusion channel of OrganoPlate® chips with and without 3D endothelial monolayers generated by HUVECs. Chips were imaged every 3 min. See ~~Appendix~~ Fig EV3 for extended panel and quantification. Scale bar: 100 μm.

F Representative fluorescence images of CMFDA-stained untreated and DLL4 and PDGF-BB-treated hiMPs within the top perfusion channel, 15 min after delivery and kept on the OrganoFlow®. Scale bar: 50 μm.

G Bar graph quantifying adhesion images in (E). Statistical significance was calculated based on a paired *t*-test (experimental replicates = 3). Each point on the plot represents the number of adhered cells after 15 min within a single chip.

H Assessment of DLL4 and PDGF-BB-treated WT and genetically corrected DMD hiMP migration through a layer of endothelial cells. Representative images showing the lower side of the trans-well membrane on which treated and untreated hiMPs (stained with the transient dye CFDA, in green) are simultaneously seeded on HUVECs for 8 h. Bar graphs quantifying the average number of CFDA-positive cells/ mm^2, that have migrated through the endothelial layer in each considered condition. (experimental replicates = 3). A minimum of 10 (1.5 mm^2) fields per condition was quantified (error bars: SEM). Scale bar: 250 μm.

I Bar graph showing fold-change in trans-endothelial migration (error bars: SEM). Statistical significance based on one-way ANOVA with Bonferroni's multiple comparison.

Source data are available online for this figure.

BB treatment likely mediates an increase in trans-endothelial migration via modulation of the latter stages of extravasation, namely, crawling and/or diapedesis but not rolling or adhesion.

Advanced modelling of 3D tissue migration of hiMPs treated with DLL4 and PDGF-BB using bioengineered muscles

Irrespective of the delivery method (i.e. intramuscular or intravascular), transplanted cells eventually will have to migrate through a network of ECM to reach and fuse with degenerating-regenerating myofibres. To recapitulate the complexity of this process, we developed a novel humanised *quasi vivo* assay by depositing untreated and treated hiMPs on 3D human artificial muscles (Maffioletti *et al*, 2018), and performed time-lapse imaging and single-cell tracking (Fig 6A). Additionally, to mimic the microenvironment of dystrophic, degenerating muscles, artificial muscles were acutely injured with the myonecrotic agent cardiotoxin (Fig 6B). Twenty-four hours after cell deposition, artificial muscles were live-imaged for 8 h and hiMPs tracked at the single-cell level (Fig 6C and D; Movie EV2). This revealed a significant increase in the total distance travelled by hiMPs treated with DLL4 and PDGF-BB in comparison to untreated hiMPs (Fig 6E). Furthermore, hierarchical clustering was performed to identify two clusters based on total distances travelled. Cells of cluster 1 travelled an average of 47.44 μm over 8 h, whilst cells of cluster 2 travelled 74.73 μm in the same timeframe (Fig 6F). After treatment with DLL4 and PDGF-BB, there was a significant increase in the proportion of cells in cluster 2, from 4.17% in the untreated condition to 45.83% in the treated condition ($P < 0.001$; Fig 6G). Collectively, these results show that modelling intravascular delivery and intramuscular migration in complex humanised platforms validates the observation that modulation of NOTCH and PDGF signalling pathways improves migration of hiMPs across endothelial monolayers and in within regenerating myofibres. These findings lay the foundation for future studies aimed at elucidating and further enhancing the molecular mechanism underpinning this phenomenon.

Discussion

In this work, we exploited directed iPSC-differentiation, transcriptomics, single-cell profiling, microfluidics and 3D tissue engineering to develop a strategy to induce and enhance hiMP migration properties. We show that DLL4 and PDGF-BB treatment induces a transcriptional profile comparable to those detected in MuSCs from mouse and human primary samples, including key markers of myogenic commitment, downstream NOTCH signalling targets and perivascular markers. This transcriptional response is most likely caused by the role of DLL4 and PDGF-BB as developmental determinants of skeletal muscle pericytes (Cappellari *et al*, 2013; Moyle *et al*, 2019). Notably, hiMPs responded to treatment more consistently than adult mouse and human myoblasts, possibly due to their relatively immature state compared with their adult counterpart. Interestingly a recent study showed that hiMPs are transcriptionally comparable to late embryonic and early foetal myoblasts (Xi *et al*, 2020). Secondary myogenesis occurs between E14.5–17.5 of mouse development during which foetal myoblasts either contribute to existing primary myofibres or fuse with each other to give rise to secondary muscle fibres (Messina & Cossu, 2009). Spontaneous fate transitions of myoblasts to pericytes were observed in foetuses at E16.5 (Cappellari *et al*, 2013). Therefore, it is possible that hiMPs reflect this plastic foetal myoblast nature and therefore might respond more robustly to DLL4 and PDGF-BB treatment.

Morphological analysis between treated and untreated hiMPs revealed that DLL4 and PDGF-BB induced shape changes in a subset of treated cells, in keeping with perturbations of the actin cytoskeleton highlighted by RNAseq data (Fig 3A). Several studies have demonstrated that chemokines enhance myoblast migration via direct regulation of the actin cytoskeleton (Kawamura *et al*, 2004; Ishido & Kasuga, 2011). For example, hepatocyte growth factor-mediated increase in migration is facilitated by lamellipodia formation via the PI3K/AKT and ERK/MEK signalling pathways (González *et al*, 2017). Additionally, stromal-derived factor 1 (SDF1), via interaction with CXCR4, increases migration via upregulation of Rho

© 2022 The Authors

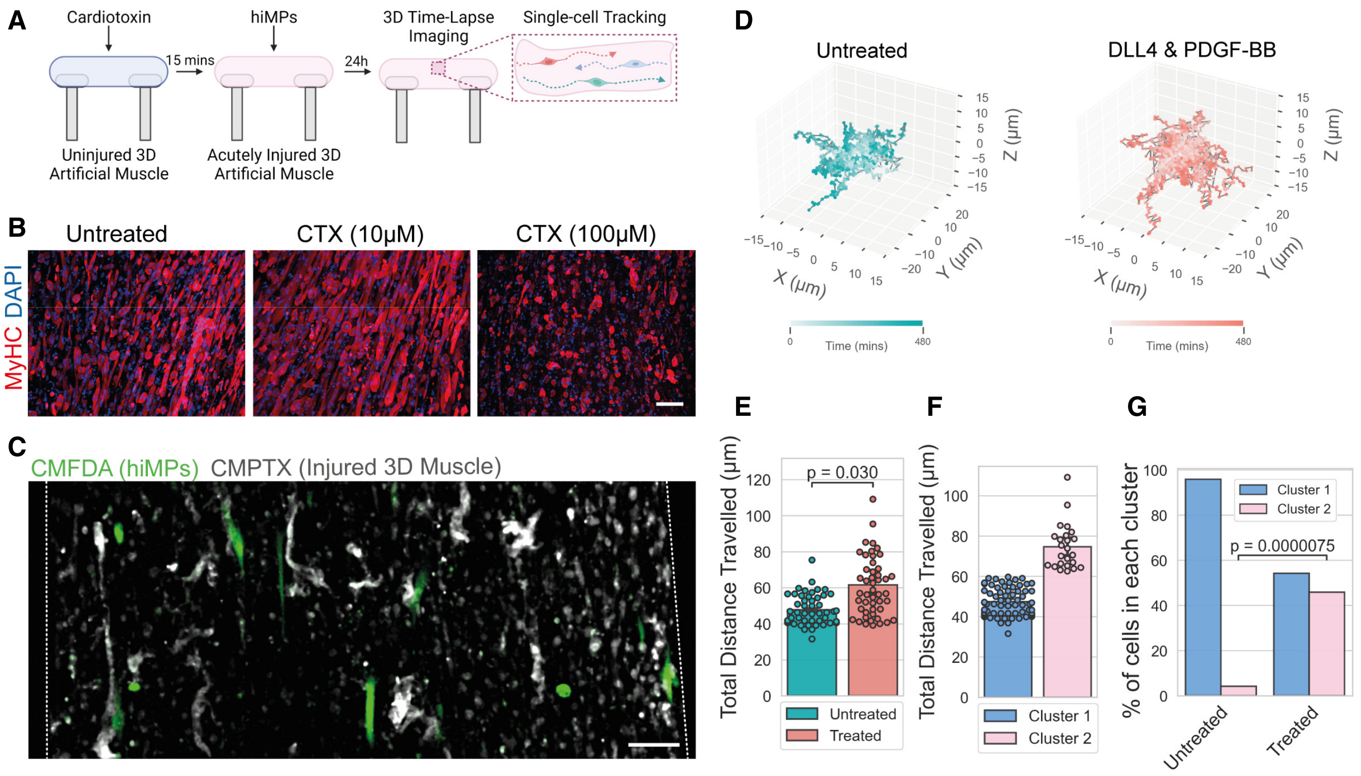

Figure 6. *Quasi vivo* modelling of hiMP tissue migration using 3D human bioengineered muscles.

A Schematic representation of the experimental setup (produced with BioRender, www.biorender.com).

B Immunofluorescence images of 3D artificial muscles stained for myosin heavy chain (MyHC), after 15 min exposure to PBS (uninjured) or 10/100 μM cardiotoxin. Scale bar: 50 μm.

C Maximum intensity projections of fluorescence images of CMPTX-labelled human 3D muscles after background subtraction, ~~stained with CMPTX deposited with~~ containing “transplanted” CMFDA-labelled (green) hiMPs. Dotted lines demarcate the outline of the 3D construct. Scale bar: 100 μm. Time lapse video availabe in Movie EV2.

D 3D trajectory plots for visualisation of single-cell tracks of hiMPs on 3D muscles for 8 h for both untreated (left) and DLL4 and PDGF-BB-treated (right) conditions.

E Bar chart representing the total distances travelled of single-cells tracked for DLL4 and PDGF-BB and untreated hiMPs. Statistical testing was performed with an independent *t*-test with each experimental replicate as data points (experimental replicates = 3). Velocities of individual cells are displayed as single points to visualise the distribution of data.

F Bar chart displaying the velocity of cells within clusters generated using hierarchical clustering of cells using total distance travelled as a feature (S_i = 0.67). Each point represents a single cell.

G Bar plots showing the proportions of untreated and DLL4 and PDGF-BB-treated cells within the two clusters shown in (F). Statistical test performed with a Chi-squared (χ^2) test.

Source data are available online for this figure.

GTPases, *CDC42* and *Rac1* in addition to several other migration-associated transcripts such as actin bundling protein, *ACTN1* and calcium-dependent protease, *CAPSN1*, necessary for cleavage of focal adhesions (Kowalski *et al*, 2017). The presence of a subset of cells displaying morphological changes in response to treatment could also indicate the existence of multiple cell states within the hiMP population with differential susceptibility to perturbations of NOTCH and PDGF signalling, also suggested by our migration analyses. Future work should consider correlating single-cell RNAseq and motility analyses to identify responders, characterising cell states, increasing purity of the treated population and defining molecular mechanisms responsible for increased cell migration.

Any viable treatment to improve engraftment of cell therapy products should not impact negatively on their proliferation and differentiation capacity. DLL4 and PDGF-BB treatment did not alter hiMP proliferation and the expected NOTCH-mediated reduction in differentiation was rescued upon removal of the exogenous DLL4 and PDGF-BB stimuli or, more rapidly by γ-secretase inhibition of NOTCH signalling.

In silico analyses and *in vitro* assays indicated that treated hiMPs possess enhanced motility and trans-endothelial migration, validating our initial hypothesis that DLL4 and PDGF-BB modulate migration also in hiPSC derivatives. Microfluidic devices allowed us to interrogate the trans-endothelial migration process of hiMPs under flow conditions, enabling dissection of two distinct processes that occur during extravasation: adhesion and diapedesis. In leucocytes, trans-endothelial migration takes place in a step-wise, sequential manner, consisting of initial adhesion of cells to the endothelial wall and subsequent migration through intercellular gaps of the blood vessel (reviewed in Choi *et al*, 2020). Our results indicate that DLL4 and PDGF-BB do not modulate the adhesive properties of hiMPs, suggesting a possible mechanism of the treatment on more

© 2022 The Authors

downstream processes of extravasation. These findings prompted us to explore relevant signalling pathways and molecules involved in cell extravasation. Leukocytes are the typical benchmark for extravasating cells and many genes regulating leukocyte extravasation were found to be differentially expressed upon DLL4 and PDGF-BB treatment in hiMPs. Interestingly, molecules relevant for leukocyte extravasation such as CXCL12 and integrin β2, were downregulated upon DLL4 and PDGF-BB treatment, suggesting that myogenic progenitor trans-endothelial migration may not necessarily mimic all aspects of leukocyte extravasation. For example, downregulation of *JAM-A* enhances trans-endothelial migration of adult muscle pericyte-derived mesoangioblasts (Giannotta *et al*, 2014). Furthermore, intra-arterial delivery of adult mouse mesoangioblasts in *JAM-A-null* dystrophic mice resulted in increased engraftment, indicating that absence of endothelial JAM-A improves trans-endothelial migration of myogenic cells (Giannotta *et al*, 2014; Bonfanti *et al*, 2015). This is in contrast to leukocytes, in which isophilic interactions between JAM-A of leukocytes and endothelial cells are necessary for efficient extravasation (Corada *et al*, 2005; Woodfin *et al*, 2007, 2009). Although not all mechanisms of leukocyte extravasation are mimicked by myogenic progenitors, it remains possible that conserved elements may exist (e.g., Fig 5A). Combining these features with the machinery that other non-haematopoietic cells use to travel through endothelia (e.g., metastatic cancer cells) could provide additional tools for myogenic cells to efficiently extravasate. Notably, our work goes beyond modelling extravasation, as our newly developed *quasi vivo* migration assay on 3D bioengineered muscles enabled us to study events which follows diapedesis, such as migration within regenerating human myofibres.

Future studies should also investigate *in vitro* high-throughput and high-fidelity methods to evaluate cell transmigration, ideally using organotypic (i.e., skeletal muscle-specific) endothelial and smooth muscle cells on top of a basement membrane, which could facilitate unravelling of adhesion profiles and tissue-specific recruitment mechanisms necessary for efficient trans-endothelial migration. This strategy may be more informative than interspecific *in vivo* experiments based upon hiMPs delivery within murine blood vessels, where the species mismatch could affect receptor recognition and downstream signalling (e.g., limited interactions between human-mouse selectins/integrins). In summary, this study provides an important first step towards defining druggable targets to increase the migration capacity of hiMPs, ultimately contributing to the identification of a systemically deliverable and engraftable hiPSC-derivative for muscle cell therapies.

Materials and Methods

Cell isolation and culture

Primary mMuSCs were isolated, purified via FACS from skeletal muscles of four distinct *Tg:Pax7-nGFP* F1:C57BL/6:DBA2 mice expressing nuclear-localised EGFP in Pax7-expressing cells (Sambasivan *et al*, 2009) and cultured as previously reported (Gerli *et al*, 2019). C2C12 myoblasts (Yaffe & Saxel, 1977) were cultured in DMEM supplemented with 10% FBS, 1% glutamine (Sigma-Aldrich) and 1% penicillin–streptomycin. Primary human myoblasts from three different donors were obtained from the MRC Neuromuscular Centre Biobank (shortened as L3, L5 and L8), FACS-purified for $CD56^+$ (Biolegend; CD56-FITC 304604) and cultured as previously reported (Gerli *et al*, 2019); an additional polyclonal population of biopsy-derived human myoblasts was purchased (Gibco human skeletal myoblasts A12555, shortened in the text as GI) and purified using CD56. Human immortalised myoblasts were kindly provided by the Myoline platform of the Institut de Myologie, Paris, France. Five different hiPSC lines have been used in this study; four lines have been used for most experiments: N1 (short for NCRM-1: https://hpscreg.eu/cell-line/CRMi003-A), N5 (short for NCRM-5: https://hpscreg.eu/cell-line/CRMi001-A); SBI (short for SBIi006-A: https://hpscreg.eu/cell-line/SBIi006-A); A1 (short for: Gibco Episomal hiPSC line A13777); Genetically-corrected DMD (DYS-HAC) iPSCs were generated using Sendai-virus-delivered reprogramming factors and kindly provided by Dr. Y. Kazuki and Prof. M. Oshimura (Tottori University, Japan; Choi *et al*, 2016). hiPSCs were cultured on vitronectin XF™ (Stemcell Technologies; 07180) at 37°C, 5% CO_2 and 3% O_2. mTESR-E8™ (Stemcell Technologies; 07174) was used for cell expansion and colonies were passaged using gentle dissociation media (Stemcell Technologies; 07174) according to manufacturer's instructions. Skeletal myogenic differentiation of hiPSCs was performed with a commercially available protocol (Genea Biocells, now Myocea; Caron *et al*, 2016). Briefly, hiPSCs were dissociated into single cells and plated onto Matrigel-coated (Corning) dishes. Subsequently, cells were exposed to induction medium for 10 days to generate myogenic progenitors. The resulting hiMPs were expanded in Genea's myoblast medium, Lonza's myoblast growth and proliferation medium (SKBM-2) or in-house DMEM/F12 (Gibco; 11320074)-based primary myoblast medium (Gerli *et al*, 2019), as detailed in specific sections. All myogenic cells were differentiated in DMEM containing 2% horse serum apart from 3D bioengineered muscle which were differentiated in DMEM containing 10 μg/ml insulin (Gibco; I0516-5ML). Human cell work was conducted under the approval of the NHS Health Research Authority Research Ethics Committee reference no. 13/LO/1826; IRAS project ID no. 141100.

DLL4, PDGF-BB and γ-secretase inhibitor treatment

Recombinant human DLL4 (DLL4 fused with the Fc domain of human IgG; R&D Systems; 1506-D4) was resuspended to a final concentration of 10 μg/ml in sterile PBS containing 1% wt/vol bovine serum albumin (BSA; Sigma-Aldrich; A9418-10G) as a carrier protein. Standard cell culture plastic dishes were coated with the DLL4 solution and incubated at 37°C for 45 min. Cells were then seeded on the coated flasks and supplemented with 100 ng/ml of human PDGF-BB re-suspended in 0.1% BSA/4 mM HCl/PBS (R&D Systems; 200-BB-050) daily for at least 7 days. As for normal myogenic differentiation assays, DLL4 and PDGF-BB-treated myoblasts and untreated control were seeded at a high density on collagen-coated dishes and, when confluent, switched to differentiation medium (DMEM supplemented with 2% HS (w/v) + 1% P/S (w/v)). To block NOTCH signalling, cells were incubated with 660 ng/ml of γ-secretase inhibitor (L685458, Sigma) 24 h before the switch to differentiation medium and over the two following days.

 © 2022 The Authors

RNA sequencing

RNA library preparation

Mouse MuSC-derived myoblasts, human myoblasts and hiMPs (detailed in previous section "Cell isolation and culture") were seeded on dishes coated with 10 μg/ml DLL4 and medium supplemented daily with 50 ng/ml recombinant PDGF-BB with a minimum of 1 passage throughout the 7 days reprogramming protocol to replace the DLL4 protein. An untreated control was grown in parallel on 1% BSA-coated dishes. After 7 days, samples were collected and RNA extracted with Qiagen RNeasy kit, with on-column DNaseI treatment. RNA concentration and integrity were assessed by Nanodrop spectrophotometer and Agilent 2100 bioanalyzer (model G2939A). An RNA Integrity Number (RIN; Schroeder *et al*, 2006), was quantified for each sample and scores between 9.8 and 10 accepted. Library preparations were performed with the UCL Genomics facility, using the KAPA mRNA HyperPrep Kit (Roche) to capture mRNA and deplete ribosomal RNA. Samples were barcoded and run together on an Illumina NextSeq 550 System to minimise batch variation.

Analyses

Raw sequence data were pre-processed to remove small (> 20 bp) or poor quality reads using Trimmomatic v0.36.4 (Bolger *et al*, 2014). Reads were aligned either to the Human hg38 genome using Spliced Transcripts Alignment to a Reference (STAR) software v2.5.2b (Dobin *et al*, 2013), mapped reads de-duplicated with Picard v2.7.1.1 (Broad Institute) and reads-per-transcript calculated with feature Counts v1.4.6.p5 read summarisation tool (Liao *et al*, 2014). Finally, differential expression was calculated using SARTools R package v.1.3.2.0 (Varet *et al*, 2016), based on the DESeq2 model and package (Love *et al*, 2014).

In order to perform gene set enrichment analyses, mouse gene symbols were first converted into their respective human orthologs using the BiomaRt v2.46.2 package (Smedley *et al*, 2009). Subsequently, HUGO Gene Nomenclature Committee (HGNC) symbols were converted into Entrez Gene IDs using BiomaRt. Differentially expressed genes with a fold-change > 2 and a P-value < 0.05 were then subjected to gene set enrichment analysis with ClusterProfiler v3.18.0 and ReactomePA v1.34.0 packages (Yu *et al*, 2012; Yu & He, 2016); script available as Dataset EV1.

To analyse signalling pathway changes in response to DLL4 and PDGF-BB, differential expression data were inputted into Ingenuity Pathway Analysis (IPA, Qiagen). The Genebank gene ID, log2 fold change expression, P-value and adjusted P-values (Padj) were included, in order to account for the experimental false discovery rate. To ensure only highly likely interactions were accounted for, only experimentally observed interactions in mammalian cells were included, and cut-offs were set at log2 fold change (−0.58, +0.58 i.e. a fold change of 1.5) with a padj of 0.05. From this, 2,259 genes (1,002 increased, 1,557 decreased) remained on the hiMP dataset. Additional expression analyses were performed using Stemformatics (www.stemformatics.org/; Choi *et al*, 2019). Functional protein association network analysis was performed using https://string-db.org. RNAseq reads and scripts utilised for PCA and gene enrichment analyses available upon request to the corresponding author. European Nucleotide Archive (ENA) study accession number: PRJEB43338.

Quantitative real-time PCR

Cells were seeded on 6- or 12-well plates for at least 24 h before detaching them and centrifuging at 336.47 *g* to obtain pellets for RNA extraction using RNeasy Micro kit (Quiagen, 74004) according to manufacturer's instructions. RNA purity and yield were assessed using a Nanodrop spectrophotometer. Retro-transcription to cDNA was performed with the ImProm-IITM Reverse Transcription System kit (Promega, A3800) following manufacturer's instructions; a minimum of 50 ng of RNA per reaction was used. qRT-PCRs were performed with the SYBR-Green Real Time Master Mix (Promega; A600A) according to manufacturer's instructions using a BioRad CFX96 machine. qRT-PCRs were performed in triplicate on samples from at least three independent experiments. Ct data were normalised to GAPDH (Stern-Straeter *et al*, 2009). Data were presented as mean ± SEM of the fold change. Significance was assessed on the delta Ct values using Student's two-tailed t-test. List of primers used are available in Table 2.

Immunofluorescence

Cells were fixed in 4% paraformaldehyde (PFA) for 5 min at room temperature (RT), washed twice with phosphate buffered saline (PBS) and incubated 30 min with PBS-1% BSA-0.2% triton. Cells were then incubated for 30 min with 10% donkey or goat serum solution at RT to reduce non-specific antibody binding. Primary antibodies were diluted to the appropriate concentration (Table 3) in PBS-1% BSA-0.2% triton and incubated either 1 h at RT or overnight at 4°C. Subsequently, cells were washed three times with PBS-0.2% triton to eliminate unbound antibody and then incubated for 1 h with fluorescently conjugated secondary antibodies raised in goat or donkey and Hoechst 33342 to visualise nuclei (Fluka; B2261). Cells were imaged using an inverted fluorescence microscope (Leica DMI6000B). At least 5 non-overlapping random field images were acquired and analysed using ImageJ or Adobe Photoshop software.

Fluorescence activated cell sorting (FACS)

Cells were prepared for FACS analysis as previously published (Maffioletti *et al*, 2015). Briefly, cells were trypsinised and filtered through a 40 μm cell strainer to get a single cell suspension. At

Table 2. List of primers used.

Gene	Forward primer	Reverse primer
GAPDH	TTCACCACCATGGAGAAGGC	GGCATGGACTGTGGTCATGA
PAX7	CAAACACAGCATCGACGG	CTTCAGTGGGAGGTCAGGTT
MYOD	AATAAGAGTTGCTTTGCCAG	GTACAAATTCCCTGTAGCAC
MYOGENIN	CCAGGGGTGCCCAGCGAATG	AGCCGTGAGCAGATGATCCCC
PDGFRB	AGCTGTTACCCACTCTGGGA	TGGTGTCCTTGCTGCTGATG
TNAP	TGTGGGGTGAAGGCCAATG	GTGGTGGTCACAATGCCCA
CD146	GGAAGCAGGAGATCACGCTAC	GATTCGGGGCTAATGCCTCA
HEY1	AGGTTACTTTGACGCGCACG	ACCAGTCGAACTCGAAGCG
HES1	AGAAAGATAGCTCGCGGCA	TACTTCCCCAGCACACTTGG

Table 3. List of antibodies used.

Antibody	Dilution	Company; Catalogue number
Anti-MyoD	1:100	Santa Cruz; sc-760 (M-318)
Anti-Pax-6	1:100	Santa Cruz; sc-81,649
Anti-MAP-2	1:100	Santa Cruz; sc-74,421
Anti-MyHC	1:9	DSHB; MF20
Anti-CD31	1:40	Abcam; 28,364
Anti-SPECTRIN	1:100	Leica Biosystems; NCL-SPEC1
Anti-Lamin A/C	1:250	Novocastra; NCL-LAM
Phalloidin	1:400	Invitrogen; A30137

least 1.5×10^5 cells were stained for each fluorochrome-conjugated primary antibody for 1 h on ice. An additional unstained control tube was included for each cell line. Cells were then washed, fixed in 2% (w/v) paraformaldehyde for 5 min after which 3 ml of FACS buffer was added and cells centrifuged at 232 *g* for 5 min. Supernatant was discarded and cells were resuspended in 100 µl FACs buffer and sorted with a CyAn™ ADP Analyser (Beckman Coulter, Inc.) at the UCL GOSICH Flow Cytometry Core Facility. A minimum of 20,000 events per antibody were analysed. FACS data analysis was done using FCS Express 4 (*De Novo* Software). A similar procedure was followed for FACS cell purification, apart from fixation. Cells were sorted using a MoFlo XDP machine (Beckman Coulter).

Morphometry and proliferation analyses

To compare morphology between DLL4 and PDGF-BB-treated and untreated hiMPs, the circularity ratio of cells was analysed using ImageJ (0 = line; 1 = perfect circle). Circularity ratios of cells were obtained via quantification of manually labelled cell contours of phase-contrast images. Three random fields were analysed for three independent experiments with at least 300 cells analysed for each biological replicate. To identify differences in proliferation between DLL4 and PDGF-BB-treated and untreated hiMPs, cells were pulsed with 10 µM 5-Ethynyl-2'deoxyuridine (EdU) for 2 h following manufacturer's instructions (Click-iT® EdU Alexa Fluor® Imaging Kit, Life Technologies). Cells were subsequently fixed and stained with Hoechst 33342. The proportion of proliferating cells was then calculated by comparing the number of EdU+ nuclei with the total number of nuclei within the field.

Cell motility assays

For conventional motility assay, 1.5×10^4 hiMPs were plated in triplicate onto 24-well multi-well dishes and incubated overnight. For conditions of continuous treatment, hiMPs were plated onto 24-well dishes coated with either 1% BSA or DLL4 and supplemented with PDGF-BB. Cells were pulsed with Hoechst 33342 (100 ng/ml) for 45 min prior to imaging to aid tracking. Imaging was performed with the ImageXpress acquiring images every 10 min for 12 h (segmented using ImageJ). Cell tracking, calculation of total distance travelled (µm), velocity (µm/min), mean straight line speed (µm/min) and total displacement (µm) was performed with Trackmate (Tinevez *et al*, 2017). Only cells remaining within the field were analysed. Statistical analysis was performed on three independent repeats with a minimum of 20 cells/condition/repeat.

For analysis of cell motility with Heteromotility, image segmentation was performed using a deep learning approach. A StarDist model was trained on manually annotated images obtained from individual frames of the tracking dataset selected to capture variation in intensity, shape and size between nuclei. Images were annotated with Caliban (https://github.com/vanvalenlab/caliban) and the StarDist model was trained from scratch using the ZeroCostDL4Mic platform (Schmidt *et al*, 2018; von Chamier *et al*, 2021). Single-cell tracking was performed with Bayesian Tracker with modified configurations to optimise tracking for videos obtained from ImageXpress (Bove *et al*, 2017; https://github.com/quantumjot/BayesianTracker). Analysis of single-cell tracks of lengths > 60 was subsequently performed with Heteromotility using all features except turning features (Kimmel *et al*, 2018; https://github.com/cellgeometry/heteromotility). The following parameters were utilised: "total_distance" travelled by the cell during time-lapse; "net_distance" travelled by the cell during time-lapse; "linearity": linear regression analysis of the XY coordinates of a cell at each time point; "spearmanrsq": assessment of the monotonic relationship of the distribution of XY coordinates of cells at each time point;" progressivity": ratio between "net_distance" and "total_distance", serving as an indicator of the directional nature of the cell track during time course. Larger values suggest directional motility; "max_speed", "min_speed" and "avg_speed": maximum/minimum/average speed of a cell during time-lapse; "MSD_slope": spatial deviation of a cell with respect to a reference position during time-lapse. Higher values suggest directional motility whilst lower values indicate random motion; "hurst_RS": a metric of directional persistence. Values < 0.5 suggests non-persistent behaviour. A value of 0.5 indicates brownian motion. Values between > 0.5 and 1.0 indicate persistent behaviour; "nongauss": the extent of the non-Gaussian nature of the distribution of displacement of the cell within timelapse—value closer to 0 indicate Gaussian distribution; "rw_linearity": linearity of a cell track minus linearity of a simulated random walk; "rw_netdist": net distance travelled by a cell minus net distance of a simulated random walk; "rw_kurtosis": kurtosis of a cell displacement minus kurtosis of a random walk for each sub-track; "avg_moving_speed" of a cell during a specified sub-track; "time_moving": proportion of time spent moving by the cell during a sub-track; "autocorr": similarity of a cell displacement series as a function of time lag between each displacement.

Microfluidic and transwell assays

Microfluidic assessment of cell adhesion was performed using the OrganoPlate® 3-lane 40 and the OrganoFlow® S (Mimetas). 3D channels were endothelialised with HUVECs (Lonza) cultured in EGM1 medium (Lonza) at 37°, 5% CO_2. The "OrganoPlate® 3-lane tubule seeding" protocol (www.mimetas.com/en/knowledge-center/) was followed with minor alterations. First, the 4 mg/ml collagen-I mixture was prepared by mixing 5 mg/ml collagen-I (AMSbio: 3447-020-01), 1 M HEPES (Thermofisher: 15630130), and 37 g/l $NaHCO_3$ in a 8:1:1 ratio. 2 µl of the mixture of was added to the central channel of each chip and the plate was subsequently incubated at 37°C for 15 min to facilitate collagen polymerisation. 3×10^5 HUVECs were then seeded into the top medium inlet and

© 2022 The Authors

the plate was left on a 70° angle to allow direct attachment of HUVECs against the ECM gel for 5 h. Following cell attachment, the plate was placed on an OrganoFlow® S (Mimetas; 7°; 8 min) to induce flow. All tubules were used between 48 and 72 h after seeding. Tubules were fixed with 4% PFA for 10 min at RT. Culture medium was aspirated and PFA was added to wells at the following volumes: 100 µl in the top medium inlet, 50 µl in the top medium outlet, gel and bottom medium inlets and outlets. Staining of chips was performed according to manufacturer's instructions (www.mimetas.com/en/knowledge-center/) with minor modifications. All steps, unless specified otherwise were performed with the following volumes in each well: 100 µl in the top medium inlet, 50 µl in the top medium outlet as well as gel and bottom medium inlets and outlets. First, cells were permeabilised with 1% BSA wt/vol and 0.2% Triton-X in PBS for 30 min. A blocking step was then performed using 2% FBS and 2% BSA in PBS for 30 min. Primary antibodies were diluted to appropriate concentrations in permeabilisation buffer, 25 µl were added to the top medium inlets and outlets and 15 µl was added to the bottom medium inlets and outlets. After three washes with 0.2% Triton/PBS, fluorescently conjugated secondary antibodies, diluted in 0.2% Triton-X/PBS, were added to chips at the same volumes as the primary antibody solution and incubated for 1 h at room temperature. Subsequently, two washes with 0.2% Triton-X/PBS and one wash with PBS was performed prior to imaging. Barrier integrity assays were performed following manufacturer's instructions (www.mimetas.com/en/knowledge-center/) with minor adaptations. A "wetting step" was performed prior to the assay by adding 50 µl EGM1 to the gel and bottom perfusion inlets and outlets for 5 min. A dextran working solution of 0.5 mg/ml 20 kDa FITC dextran and 0.5 mg/ml 150 kDa TRITC dextran was prepared in EGM1. All media was removed from the top medium inlet and outlet, whilst 20 µl of media was left in the gel and bottom perfusion inlets and outlets. Forty microliter and 30 µl of the dextran working solution was added to the top perfusion inlet and outlet, respectively. Imaging was initiated immediately after addition of the dextran solution every 3 min for 15 min on the LTTL system. Normalised intensity was calculated as a ratio of fluorescence between the top perfusion channel and ECM channel. Quantification of fluorescence was performed using FIJI by manual annotation of regions of interest (ROI) within the perfusion and ECM channels. The ROI was kept consistent across time points, fluorescence channels, top and central tubules. The integrated density, the sum of all pixel values within the ROI, was used as the measurement to calculate normalised intensity. To assess cell adhesion on endothelialised tubules under flow condition, cells labelled with 2.5 µM 5-chloromethylfluorescein diacetate (CMFDA; ThermoFisher Scientific) were resuspended in a mixture of myoblast proliferation medium (MM; Gerli *et al*, 2019) and EGM1 at a 1:1 ratio (400 cells/µl). After generation of blood vessels at least 48 h prior to seeding, 50 µl of MM/EGM1 media was added in the perfusion outlets. Subsequently, 50 µl of cell suspension was added to top medium inlets. The OrganoPlate® was then placed on the OrganoFlow® S (7°; 4 min) for 15 min. Chips were imaged using the EVOS™ M5000. Adhesion was calculated by counting the number of in-focus attached cells within the top perfusion channels after 15 min on the OrganoFlow® S. After imaging adhesion, we attempted to use the same chips to assess transmigration; however, this was complicated by HUVECs consistently migrating in the ECM channel in response to chemoattractants, resulting into disruption of the endothelial cell architecture in the tubules. This confounding factor and background noise from HUVECs prompted us to assess trans-endothelial migration using transwell dishes as an alternative assay.

In vitro trans-endothelial migration (transwell) assay was performed using HUVECs (Lonza) grown at 37°C, 5% CO_2 in EGM1 (Lonza) on 1% gelatin-coated flasks (Sigma), kept below 70% confluence and used up to passage 6. Eight micrometer porous cell culture membranes (BD Biosciences; 353093) were coated with 1.5% gelatin for 1 h at 37°C, cross-linked with 2% glutaraldehyde (Sigma) for 15 min at RT, incubated with 70% ethanol for 1 h at RT and washed three times with PBS before an overnight incubation in 2 mM glycine/PBS at 4°C. After 5 PBS washes, 2×10^5 HUVECs were seeded on top and grown to confluence for at least 72 h. hiMPs were then dissociated with TrypLE Select (Thermo Fisher Scientific) and labelled with 0.7 µM 6-carboxyfluorescin dictate (6-CFDA; ThermoFisher Scientific) for 30 min at 37°C. Subsequently, the upper chamber was loaded with 3×10^4 cells to be tested, resuspended in serum-free medium. The lower chamber was loaded with a chemoattractant composed of 50% fresh growth medium and 50% medium previously exposed for 24 h to differentiated C2C12 myoblasts. After 8 h, membranes were washed in PBS and fixed for 5 min in 4% PFA. The upper side of the membrane was scraped with a cotton bud to remove non-migrated cells. After an additional PBS wash, membranes were mounted on slides and cells migrated through the endothelial layer were quantified by counting the number of fluorescent cells on the lower side of the membrane using a Leica

The paper explained

Problem

Cell therapies to treat severe muscular dystrophies remain inefficient to date. Major hurdles to clinical efficacy of such therapies include the limited ability to expand mature myogenic cells *in vitro*, as well as the minimal migration capacity of myogenic cells upon transplantation which reduces their ability to disperse into the affected tissues where they are needed.

Results

We used a developmentally-inspired treatment (via DLL4 and PDGF-BB) activating the NOTCH and PDGF pathways to impart higher migratory capacity to human myogenic cells derived from induced pluripotent stem cells (iPSCs; a source providing a potentially limitless supply of cells). We showed the efficacy of this treatment using a range of advanced and emerging technologies such as directed iPSC differentiation, single-cell profiling, microfluidics and 3D tissue engineering.

Impact

In this work, we developed tools to assess and a strategy to induce and enhance migration of iPSC-derived myogenic cells, with translational relevance for both intramuscular and intra-vascular cell delivery. Enhancing extravasation is a key milestone to develop future efficacious muscle cell therapies. Moreover, the technological platforms we have used for the validation of this treatment can be applied further to wider drug and therapy screening purposes.

© 2022 The Authors

DMI6000B microscope. A minimum of 10 random 20× field/condition per experiment was quantified. Experiments were performed in duplicate on at least three separate occasions.

Migration assay on 3D bioengineered human muscles

3D artificial muscles were generated using immortalised human myoblasts as previously described (Maffioletti *et al*, 2018; Pinton *et al*, 2022). hiMPs were stained with 2.5 μM CMFDA for 30 min at 37°C, 5% CO_2 and 3D muscles were stained with 5 μM CMPTX (ThermoFisher Scientific; C34552) for 45 min at 37°C, 5% CO_2. Acute injury was induced in 3D muscles by depositing 5 μl cardiotoxin (CTX; Sigma) on their surface for 15 min at RT; muscles were then washed in warm PBS. hiMPs were resuspended in 50% MM media, 50% matrigel (8,000 hiMPs/μl). Five microliter of hiMP suspension was deposited onto the surface of the bioengineered muscles and incubated without media for 10 min at 37°C, 5% CO_2 to facilitate Matrigel polymerisation before returning them to 24-well multi-well plates with MM media. After 24 h, 3D muscles were subjected to time-lapse microscopy with a CSU-W1 spinning disk microscope over 12 h with imaging performed every 12 min with a 10× objective. A 100 μm z-stack was taken every 5 μm.

Analysis of 3D migration was performed with Imaris (version 9.0.1). First, a 2 × 2 binning was performed and images of both hiMPs and 3D muscle were subjected to median filtering and background subtraction prior to segmentation and tracking. To correct for drift of the 3D muscle, a mean trajectory of the muscle was approximated. This involved identifying highly correlated tracks within the 3D muscle and was performed using the cosine similarity metric with a threshold of 0.95. The average position at each time point of all correlated tracks was subsequently calculated to be used as the mean trajectory to normalise hiMP trajectories. Of note, this assay can be done in a fully isogenic setting also using 3D muscles generated from the same iPSC source utilised to derive hiMPs (Pinton *et al*, 2022).

Cell transplantation

Intramuscular transplantation of hiMPs in adult male NOD/scid/gamma (NSG) mice ($N = 3$) was done as previously described (Benedetti *et al*, 2018) with some minor modifications. Cryoinjured tibialis anterior muscles were harvested 18 days following transplantation with treated ($n = 3$) and untreated ($n = 3$) 3×10^5 N5 hiMPs and immunostained for stained LAMIN A/C (human nuclei; Leica NCL-LAM) and SPECTRIN (sarcolemma; Leica NCL-SPEC1). Operator was blinded on the cell treatment during transplantation and muscle harvesting. Animal work was performed following under UK Home Office project licences no. 70/8566 and PP2527748.

Statistical analysis

Number of experimental replicates is specified in figure legends and key experiments were repeated at least three times prior to any statistical testing ("*N*" refers to independent experiments or individual animals, "*n*" to data points). Sample size estimate was informed by expected results based upon previous work with similar treatment (Gerli *et al*, 2019). Where appropriate, subjective bias was minimised by blinding or automation of analysis (e.g. motility assays). Quantification, data distribution and statistical testing were performed using Microsoft Excel and GraphPad Prism 9 software. Statistical testing was based on Student's *t*-test unless otherwise stated. Error bars (standard deviation: SD; standard error of mean: SEM) are specified in figure legends. *P* values are specified in each figure on top of individual graphs.

Data availability

RNAseq data are available in European Nucleotide Archive (ENA; www.ebi.ac.uk/ena) within study accession number: PRJEB43338 (http://www.ebi.ac.uk/ena/data/view/PRJEB43338).

Expanded View for this article is available online.

Acknowledgements
The authors thank all members of the Tedesco laboratory and Y. Fadaili for helpful feedback, UCL Genomics for support with RNAseq analyses, O. Taso for experimental support, R. D'Antuono and the advanced light microscopy team at The Francis Crick Institute for support with imaging, S. Tajbakhsh and H. Sakai (Institut Pasteur, Paris, France), Y. Kazuki and M. Oshimura (Tottori University, Japan), K. Bentley (The Francis Crick Institute, London, UK), the Myoline platform of the Institut de Myologie (Paris, France), and the MRC Centre for Neuromuscular Diseases Biobank, supported by the National Institute for Health and Care Research (NIHR) Biomedical Research Centres at Great Ormond Street Hospital for Children NHS Foundation Trust and at University College London Hospitals NHS Foundation Trust and University College London, for providing mouse and human cells. For the purpose of Open Access, the author has applied a CC BY public copyright licence to any Author Accepted Manuscript version arising from this submission. This work was supported by: the European Research Council (759108 – HISTOID); the Francis Crick Institute which receives its core funding from Cancer Research UK, the UK Medical Research Council, and the Wellcome Trust (FC001002); Muscular Dystrophy UK (RA4/3023/1, with Duchenne Children's Trust/Duchenne UK and Duchenne Research Fund; 19GRO-PS48-0188; 17GRO-PS48-0093-1); the EU 7th Framework Program project no. 602423 (PluriMes) and the NIHR (the views expressed are those of the authors and not necessarily those of the National Health Service, the NIHR, or the Department of Health). Work in the Tedesco laboratory received also funding by the BBSRC (BB/M009513/1), AFM-Telethon (21687) and Duchenne Parent Project.

Author contributions
SungWoo Choi: Conceptualization; resources; data curation; software; formal analysis; validation; investigation; visualization; methodology; writing – review and editing. **Giulia Ferrari:** Conceptualization; data curation; formal analysis; investigation; methodology. **Louise A Moyle:** Conceptualization; formal analysis; investigation; methodology. **Kirsty Mackinlay:** Formal analysis. **Naira Naouar:** Data curation; formal analysis; methodology. **Salma Jalal:** Formal analysis; methodology. **Sara Benedetti:** Funding acquisition; investigation; methodology. **Christine Wells:** Data curation; formal analysis; supervision; investigation; writing – review and editing. **Francesco Muntoni:** Supervision; investigation; writing – review and editing. **Francesco Saverio Tedesco:** Conceptualization; data curation; formal analysis; supervision; funding acquisition; validation; investigation; methodology; writing – original draft; project administration; writing – review and editing.

In addition to the CRediT author contributions listed above, the contributions in detail are:

© 2022 The Authors

Conceptualisation: FST, GF, SWC, LAM; Methodology: SWC, GF, LAM and FST; Investigation: GF, SWC, LAM, KM, SJ, SB, NN, CW, FM and FST; Writing: FST, GF and SWC; Funding acquisition and coordination: FST. SWC and GF contributed equally to this work; their names are listed in alphabetical order and their respective position is commutative.

Disclosure and competing interests statement

FST has received speaker and consultancy honoraria from Takeda, Sanofi Genzyme and Aleph Farms (via UCL Consultants). All other authors declare that they have no conflict of interest.

For more information

i Lab website: www.tedescolab.org
ii Heteromotility source code: https://github.com/cellgeometry/heteromotility
iii Stemformatics: https://www.stemformatics.org
iv Functional protein association network analysis: https://string-db.org
v Relevant patient associations (examples): https://www.musculardystrophyuk.org

References

Albini S, Coutinho P, Malecova B, Giordani L, Savchenko A, Forcales SV, Puri PL (2013) Epigenetic reprogramming of human embryonic stem cells into skeletal muscle cells and generation of contractile myospheres. *Cell Rep* 3: 661 – 670

Armulik A, Genové G, Betsholtz C (2011) Pericytes: developmental, physiological, and pathological perspectives, problems, and promises. *Dev Cell* 21: 193 – 215

Baghdadi MB, Firmino J, Soni K, Evano B, Di Girolamo D, Mourikis P, Castel D, Tajbakhsh S (2018) Notch-induced miR-708 antagonizes satellite cell migration and maintains quiescence. *Cell Stem Cell* 23: 859 – 868

Barker TH, Grenett HE, MacEwen MW, Tilden SG, Fuller GM, Settleman J, Woods A, Murphy-Ullrich J, Hagood JS (2004) Thy-1 regulates fibroblast focal adhesions, cytoskeletal organization and migration through modulation of p190 RhoGAP and Rho GTPase activity. *Exp Cell Res* 295: 488 – 496

Benedetti S, Hoshiya H, Tedesco FS (2013) Repair or replace? Exploiting novel gene and cell therapy strategies for muscular dystrophies. *FEBS J* 280: 4263 – 4280

Benedetti S, Uno N, Hoshiya H, Ragazzi M, Ferrari G, Kazuki Y, Moyle LA, Tonlorenzi R, Lombardo A, Chaouch S *et al* (2018) Reversible immortalisation enables genetic correction of human muscle progenitors and engineering of next-generation human artificial chromosomes for Duchenne muscular dystrophy. *EMBO Mol Med* 10: 254 – 275

Betsholtz C (2004) Insight into the physiological functions of PDGF through genetic studies in mice. *Cytokine Growth Factor Rev* 15: 215 – 228

Bjornson CRR, Cheung TH, Liu L, Tripathi PV, Steeper KM, Rando TA (2012) Notch signaling is necessary to maintain quiescence in adult muscle stem cells. *Stem Cells* 30: 232 – 242

Bolger AM, Lohse M, Usadel B (2014) Trimmomatic: a flexible trimmer for Illumina sequence data. *Bioinformatics* 30: 2114 – 2120

Bonfanti C, Rossi G, Tedesco FS, Giannotta M, Benedetti S, Tonlorenzi R, Antonini S, Marazzi G, Dejana E, Sassoon D *et al* (2015) PW1/Peg3 expression regulates key properties that determine mesoangioblast stem cell competence. *Nat Commun* 6: 6364

Borchin B, Chen J, Barberi T (2013) Derivation and FACS-mediated purification of PAX3+/PAX7+skeletal muscle precursors from human pluripotent stem cells. *Stem Cell Rep* 1: 620 – 631

Bove A, Gradeci D, Fujita Y, Banerjee S, Charras G, Lowe AR (2017) Local cellular neighborhood controls proliferation in cell competition. *Mol Biol Cell* 28: 3215 – 3228

Camps J, Grosemans H, Gijsbers R, Maes C, Sampaolesi M (2019) Growth factor screening in dystrophic muscles reveals PDGFB/PDGFRB-mediated migration of interstitial stem cells. *Int J Mol Sci* 20: 1118

Cappellari O, Benedetti S, Innocenzi A, Tedesco FS, Moreno-Fortuny A, Ugarte G, Lampugnani MG, Messina G, Cossu G (2013) Dll4 and PDGF-BB convert committed skeletal myoblasts to pericytes without erasing their myogenic memory. *Dev Cell* 24: 586 – 599

Caron L, Kher D, Lee KL, McKernan R, Dumevska B, Hidalgo A, Li J, Yang H, Main H, Ferri G *et al* (2016) A human pluripotent stem cell model of facioscapulohumeral muscular dystrophy-affected skeletal muscles. *Stem Cells Transl Med* 5: 1145 – 1161

Chal J, Al Tanoury Z, Hestin M, Gobert B, Aivio S, Hick A, Cherrier T, Nesmith AP, Parker KK, Pourquié O (2016) Generation of human muscle fibers and satellite-like cells from human pluripotent stem cells in vitro. *Nat Protoc* 11: 1833 – 1850

von Chamier L, Laine RF, Jukkala J, Spahn C, Krentzel D, Nehme E, Lerche M, Hernández-Pérez S, Mattila PK, Karinou E *et al* (2021) Democratising deep learning for microscopy with ZeroCostDL4Mic. *Nat Commun* 12: 1 – 18

Choi IY, Lim HT, Estrellas K, Mula J, Cohen TV, Zhang Y, Donnelly CJ, Richard JP, Kim YJ, Kim H *et al* (2016) Concordant but varied phenotypes among Duchenne muscular dystrophy patient-specific myoblasts derived using a human iPSC-based model. *Cell Rep* 15: 2301 – 2312

Choi J, Pacheco CM, Mosbergen R, Korn O, Chen T, Nagpal I, Englart S, Angel PW, Wells CA (2019) Stemformatics: visualize and download curated stem cell data. *Nucleic Acids Res* 47: D841 – D846

Choi S, Ferrari G, Tedesco FS (2020) Cellular dynamics of myogenic cell migration: molecular mechanisms and implications for skeletal muscle cell therapies. *EMBO Mol Med* 12: e12357

Conboy IM, Rando TA (2002) The regulation of Notch signaling controls satellite cell activation and cell fate determination in postnatal myogenesis. *Dev Cell* 3: 397 – 409

Corada M, Chimenti S, Cera MR, Vinci M, Salio M, Fiordaliso F, De Angelis N, Villa A, Bossi M, Staszewsky LI *et al* (2005) Junctional adhesion molecule-A-deficient polymorphonuclear cells show reduced diapedesis in peritonitis and heart ischemia-reperfusion injury. *Proc Natl Acad Sci USA* 102: 10634 – 10639

Cossu G, Previtali SC, Napolitano S, Cicalese MP, Tedesco FS, Nicastro F, Noviello M, Roostalu U, Natali Sora MG, Scarlato M *et al* (2016) Intra-arterial transplantation of HLA-matched donor mesoangioblasts in Duchenne muscular dystrophy. *EMBO Mol Med* 8: 1470 – 1471

Darabi R, Arpke RW, Irion S, Dimos JT, Grskovic M, Kyba M, Perlingeiro RCR (2012) Human ES- and iPS-derived myogenic progenitors restore DYSTROPHIN and improve contractility upon transplantation in dystrophic mice. *Cell Stem Cell* 10: 610 – 619

Dellavalle A, Sampaolesi M, Tonlorenzi R, Tagliafico E, Sacchetti B, Perani L, Innocenzi A, Galvez BG, Messina G, Morosetti R *et al* (2007) Pericytes of human skeletal muscle are myogenic precursors distinct from satellite cells. *Nat Cell Biol* 9: 255 – 267

Dobin A, Davis CA, Schlesinger F, Drenkow J, Zaleski C, Jha S, Batut P, Chaisson M, Gingeras TR (2013) STAR: ultrafast universal RNA-seq aligner. *Bioinformatics* 29: 15 – 21

Gerli MFM, Moyle LA, Benedetti S, Ferrari G, Ucuncu E, Ragazzi M, Constantinou C, Louca I, Sakai H, Ala P *et al* (2019) Combined Notch and PDGF signaling enhances migration and expression of stem cell markers

© 2022 The Authors

while inducing perivascular cell features in muscle satellite cells. *Stem Cell Reports* 12: 461 – 473

Giannotta M, Benedetti S, Tedesco FS, Corada M, Trani M, D'Antuono R, Millet Q, Orsenigo F, Gálvez BG, Cossu G *et al* (2014) Targeting endothelial junctional adhesion molecule-A/ EPAC/ Rap-1 axis as a novel strategy to increase stem cell engraftment in dystrophic muscles. *EMBO Mol Med* 6: 239 – 258

González MN, de Mello W, Butler-Browne GS, Silva-Barbosa SD, Mouly V, Savino W, Riederer I (2017) HGF potentiates extracellular matrix-driven migration of human myoblasts: involvement of matrix metalloproteinases and MAPK/ERK pathway. *Skelet Muscle* 7: 20

Goudenege S, Lebel C, Huot NB, Dufour C, Fujii I, Gekas J, Rousseau J, Tremblay JP (2012) Myoblasts derived from normal hESCs and dystrophic hiPSCs efficiently fuse with existing muscle fibers following transplantation. *Mol Ther* 20: 2153 – 2167

Hellström M, Kalén M, Lindahl P, Abramsson A, Betsholtz C (1999) Role of PDGF-B and PDGFR-β in recruitment of vascular smooth muscle cells and pericytes during embryonic blood vessel formation in the mouse. *Development* 126: 3047 – 3055

Hicks MR, Hiserodt J, Paras K, Fujiwara W, Eskin A, Jan M, Xi H, Young CS, Evseenko D, Nelson SF *et al* (2018) ERBB3 and NGFR mark a distinct skeletal muscle progenitor cell in human development and hPSCs. *Nat Cell Biol* 20: 46 – 57

Incitti T, Magli A, Darabi R, Yuan C, Lin K, Arpke RW, Azzag K, Yamamoto A, Stewart R, Thomson JA *et al* (2019) Pluripotent stem cell-derived myogenic progenitors remodel their molecular signature upon in vivo engraftment. *Proc Natl Acad Sci USA* 116: 4346 – 4351

Ishido M, Kasuga N (2011) In situ real-time imaging of the satellite cells in rat intact and injured soleus muscles using quantum dots. *Histochem Cell Biol* 135: 21 – 26

Jalal S, Dastidar S, Tedesco FS (2021) Advanced models of human skeletal muscle differentiation, development and disease: Three-dimensional cultures, organoids and beyond. *Curr Opin Cell Biol* 73: 92 – 104

Kawamura K, Takano K, Suetsugu S, Kurisu S, Yamazaki D, Miki H, Takenawa T, Endo T (2004) N-WASP and WAVE2 acting downstream of phosphatidylinositol 3-kinase are required for myogenic cell migration induced by hepatocyte growth factor. *J Biol Chem* 279: 54862 – 54871

Kimmel JC, Chang AY, Brack AS, Marshall WF (2018) Inferring cell state by quantitative motility analysis reveals a dynamic state system and broken detailed balance. *PLoS Comput Biol* 14: e1005927

Kopan R, Nye JS, Weintraub H (1994) The intracellular domain of mouse Notch: a constitutively activated repressor of myogenesis directed at the basic helix-loop-helix region of MyoD. *Development* 120: 2385 – 2396

Kowalski K, Kołodziejczyk A, Sikorska M, Płaczkiewicz J, Cichosz P, Kowalewska M, Stremińska W, Jańczyk-Ilach K, Koblowska M, Fogtman A *et al* (2017) Stem cells migration during skeletal muscle regeneration – the role of Sdf-1/Cxcr4 and Sdf-1/Cxcr7 axis. *Cell Adhes Migr* 11: 384 – 398

Li C, Luo X, Huang B, Wang X, Deng Y, Zhong Z (2020) ADAMTS12 acts as a cancer promoter in colorectal cancer via activating the Wnt/β-catenin signaling pathway in vitro. *Ann Transl Med* 8: 301

Liao Y, Smyth GK, Shi W (2014) FeatureCounts: an efficient general purpose program for assigning sequence reads to genomic features. *Bioinformatics* 30: 923 – 930

Loperfido M, Steele-Stallard H, Tedesco F, VandenDriessche T (2015) Pluripotent stem cells for gene therapy of degenerative muscle diseases. *Curr Gene Ther* 15: 364 – 380

Love MI, Huber W, Anders S (2014) Moderated estimation of fold change and dispersion for RNA-seq data with DESeq2. *Genome Biol* 15: 550

Maffioletti SM, Gerli MFM, Ragazzi M, Dastidar S, Benedetti S, Loperfido M, Vandendriessche T, Chuah MK, Tedesco FS (2015) Efficient derivation and inducible differentiation of expandable skeletal myogenic cells from human ES and patient-specific iPS cells. *Nat Protoc* 10: 941 – 958

Maffioletti SM, Sarcar S, Henderson ABH, Mannhardt I, Pinton L, Moyle LA, Steele-Stallard H, Cappellari O, Wells KE, Ferrari G *et al* (2018) Three-dimensional human iPSC-derived artificial skeletal muscles model muscular dystrophies and enable multilineage tissue engineering. *Cell Rep* 23: 899 – 908

Matthias N, Hunt SD, Wu J, Darabi R (2015) Skeletal muscle perfusion and stem cell delivery in muscle disorders using intra-femoral artery canulation in mice. *Exp Cell Res* 339: 103 – 111

Messina G, Cossu G (2009) The origin of embryonic and fetal myoblasts: a role of Pax3 and Pax7. *Genes Dev* 23: 902 – 905

Mourikis P, Tajbakhsh S (2014) Distinct contextual roles for Notch signalling in skeletal muscle stem cells. *BMC Dev Biol* 14: 2

Moyle LA, Tedesco FS, Benedetti S (2019) Pericytes in muscular dystrophies. *Adv Exp Med Biol* 1147: 319 – 344

Parker MH, Loretz C, Tyler AE, Duddy WJ, Hall JK, Olwin BB, Bernstein ID, Storb R, Tapscott SJ (2012) Activation of notch signaling during ex vivo expansion maintains donor muscle cell engraftment. *Stem Cells* 30: 2212 – 2220

Périé S, Trollet C, Mouly V, Vanneaux V, Mamchaoui K, Bouazza B, Marolleau JP, Laforêt P, Chapon F, Eymard B *et al* (2014) Autologous myoblast transplantation for oculopharyngeal muscular dystrophy: a phase I/IIa clinical study. *Mol Ther* 22: 219 – 225

Piñol-Jurado P, Gallardo E, de Luna N, Suárez-Calvet X, Sánchez-Riera C, Fernández-Simón E, Gomis C, Illa I, Díaz-Manera J (2017) Platelet-derived growth factor BB influences muscle regeneration in Duchenne muscle dystrophy. *Am J Pathol* 187: 1814 – 1827

Pinton L, Khedr M, Lionello VM, Sarcar S, Maffioletti SM, Dastidar S, Negroni E, Choi S, Khokhar N, Bigot A *et al* (2022) 3D human induced pluripotent stem cell-derived bioengineered skeletal muscles for tissue, disease and therapy modelling. *Protoc Exch* https://doi.org/10.21203/rs.3.pex-2034/v1

Quattrocelli M, Costamagna D, Giacomazzi G, Camps J, Sampaolesi M (2014) Notch signaling regulates myogenic regenerative capacity of murine and human mesoangioblasts. *Cell Death Dis* 5: e1448

Sakai H, Fukuda S, Nakamura M, Uezumi A, Noguchi YT, Sato T, Morita M, Yamada H, Tsuchida K, Tajbakhsh S *et al* (2017) Notch ligands regulate the muscle stem-like state ex vivo but are not sufficient for retaining regenerative capacity. *PLoS ONE* 12: e0177516

Sambasivan R, Gayraud-Morel B, Dumas G, Cimper C, Paisant S, Kelly R, Tajbakhsh S (2009) Distinct regulatory cascades govern extraocular and pharyngeal arch muscle progenitor cell fates. *Dev Cell* 16: 810 – 821

Schmidt U, Weigert M, Broaddus C, Myers G (2018) Cell detection with star-convex polygons. *Lect Notes Comput Sci (including Subser Lect Notes Artif Intell Lect Notes Bioinformatics)* 11071 LNCS: 265 – 273

Schroeder A, Mueller O, Stocker S, Salowsky R, Leiber M, Gassmann M, Lightfoot S, Menzel W, Granzow M, Ragg T (2006) The RIN: an RNA integrity number for assigning integrity values to RNA measurements. *BMC Mol Biol* 7: 3

Schuster-Gossler K, Cordes R, Gossler A (2007) Premature myogenic differentiation and depletion of progenitor cells cause severe muscle hypotrophy in Delta1 mutants. *Proc Natl Acad Sci USA* 104: 537 – 542

Sciorati C, Galvez BG, Brunelli S, Tagliafico E, Ferrari S, Cossu G, Clementi E (2006) Ex vivo treatment with nitric oxide increases mesoangioblast therapeutic efficacy in muscular dystrophy. *J Cell Sci* 119: 5114 – 5123

© 2022 The Authors

Selvaraj S, Kyba M, Perlingeiro RCR (2019a) Pluripotent stem cell-based therapeutics for muscular dystrophies. *Trends Mol Med* 25: 803 – 816

Selvaraj S, Mondragon-Gonzalez R, Xu B, Magli A, Kim H, Lainé J, Kiley J, Mckee H, Rinaldi F, Aho J *et al* (2019b) Screening identifies small molecules that enhance the maturation of human pluripotent stem cell-derived myotubes. *Elife* 8: e47970

Shoji E, Woltjen K, Sakurai H (2016) Directed myogenic differentiation of human induced pluripotent stem cells. *Methods Mol Biol* 1353: 89 – 99

Skuk D (2004) Myoblast transplantion for inherited myopathies: a clinical approach. *Expert Opin Biol Ther* 4: 1871 – 1885

Smedley D, Haider S, Ballester B, Holland R, London D, Thorisson G, Kasprzyk A (2009) BioMart – biological queries made easy. *BMC Genomics* 10: 22

Stern-Straeter J, Bonaterra GA, Hörmann K, Kinscherf R, Goessler UR (2009) Identification of valid reference genes during the differentiation of human myoblasts. *BMC Mol Biol* 10: 66

Szklarczyk D, Gable AL, Lyon D, Junge A, Wyder S, Huerta-Cepas J, Simonovic M, Doncheva NT, Morris JH, Bork P *et al* (2019) STRING v11: protein-protein association networks with increased coverage, supporting functional discovery in genome-wide experimental datasets. *Nucleic Acids Res* 47: D607 – D613

Tedesco FS, Dellavalle A, Diaz-Manera J, Messina G, Cossu G (2010) Repairing skeletal muscle: regenerative potential of skeletal muscle stem cells. *J Clin Invest* 120: 11 – 19

Tedesco FS, Gerli MFM, Perani L, Benedetti S, Ungaro F, Cassano M, Antonini S, Tagliafico E, Artusi V, Longa E *et al* (2012) Transplantation of genetically corrected human iPSC-derived progenitors in mice with limb-girdle muscular dystrophy. *Sci Transl Med* 4: 140ra189

Tinevez JY, Perry N, Schindelin J, Hoopes GM, Reynolds GD, Laplantine E, Bednarek SY, Shorte SL, Eliceiri KW (2017) TrackMate: an open and extensible platform for single-particle tracking. *Methods* 115: 80 – 90

Trojanowska M (2009) Role of PDGF in fibrotic diseases and systemic sclerosis. *Rheumatology* 47: v2 – v4

Varet H, Brillet-Guéguen L, Coppée JY, Dillies MA (2016) SARTools: a DESeq2- and edgeR-based R pipeline for comprehensive differential analysis of RNA-Seq data. *PLoS ONE* 11: e0157022

Verma M, Asakura Y, Murakonda BSR, Pengo T, Latroche C, Chazaud B, McLoon LK, Asakura A (2018) Muscle satellite cell cross-talk with a vascular niche maintains Quiescence via VEGF and notch signaling. *Cell Stem Cell* 23: 530 – 543

Woodfin A, Reichel CA, Khandoga A, Corada M, Voisin MB, Scheiermann C, Haskard DO, Dejana E, Krombach F, Nourshargh S (2007) JAM-A mediates neutrophil transmigration in a stimulus-specific manner in vivo: evidence for sequential roles for JAM-A and PECAM-1 in neutrophil transmigration. *Blood* 110: 1848 – 1856

Woodfin A, Voisin MB, Imhof BA, Dejana E, Engelhardt B, Nourshargh S (2009) Endothelial cell activation leads to neutrophil transmigration as supported by the sequential roles of ICAM-2, JAM-A, and PECAM-1. *Blood* 113: 6246 – 6257

Xi H, Langerman J, Sabri S, Chien P, Young CS, Younesi S, Hicks M, Gonzalez K, Fujiwara W, Marzi J *et al* (2020) A human skeletal muscle atlas identifies the trajectories of stem and progenitor cells across development and from human pluripotent stem cells. *Cell Stem Cell* 27: 158 – 176

Yaffe D, Saxel O (1977) Serial passaging and differentiation of myogenic cells isolated from dystrophic mouse muscle. *Nature* 270: 725 – 727

Yu G, He QY (2016) ReactomePA: an R/Bioconductor package for reactome pathway analysis and visualization. *Mol Biosyst* 12: 477 – 479

Yu G, Wang LG, Han Y, He QY (2012) ClusterProfiler: an R package for comparing biological themes among gene clusters. *OMICS* 16: 284 – 287

License: This is an open access article under the terms of the Creative Commons Attribution License, which permits use, distribution and reproduction in any medium, provided the original work is properly cited.

Expanded View Figures

Figure EV1. Assessment of purity of hiMP populations.

A Representative immunofluorescence analysis of MYOD (skeletal myogenic lineage marker, green) and PAX6 (early neuroectodermal lineage marker, red) immunoreactivity in three of the four different hiMP lines used in this study.
B Immunofluorescence analysis of MYOD and MAP2 (late neuroectodermal/neuronal marker, red) in the same hiMPs shown in (A).
C Positive controls for the PAX6 and MAP2 staining shown in (A, B); top panel: spontaneously differentiating hiPSCs; bottom panel: hiPSC-derived neurons.
D Bar graph quantifying the percentages of MYOD-positive nuclei within three populations of hiMPs (experimental replicates = 3; error bars: SD). Data information: Scale bars: (A, B) 75 μm; (C) top 100 μm; bottom 20 μm.

Source data are available online for this figure.

 © 2022 The Authors

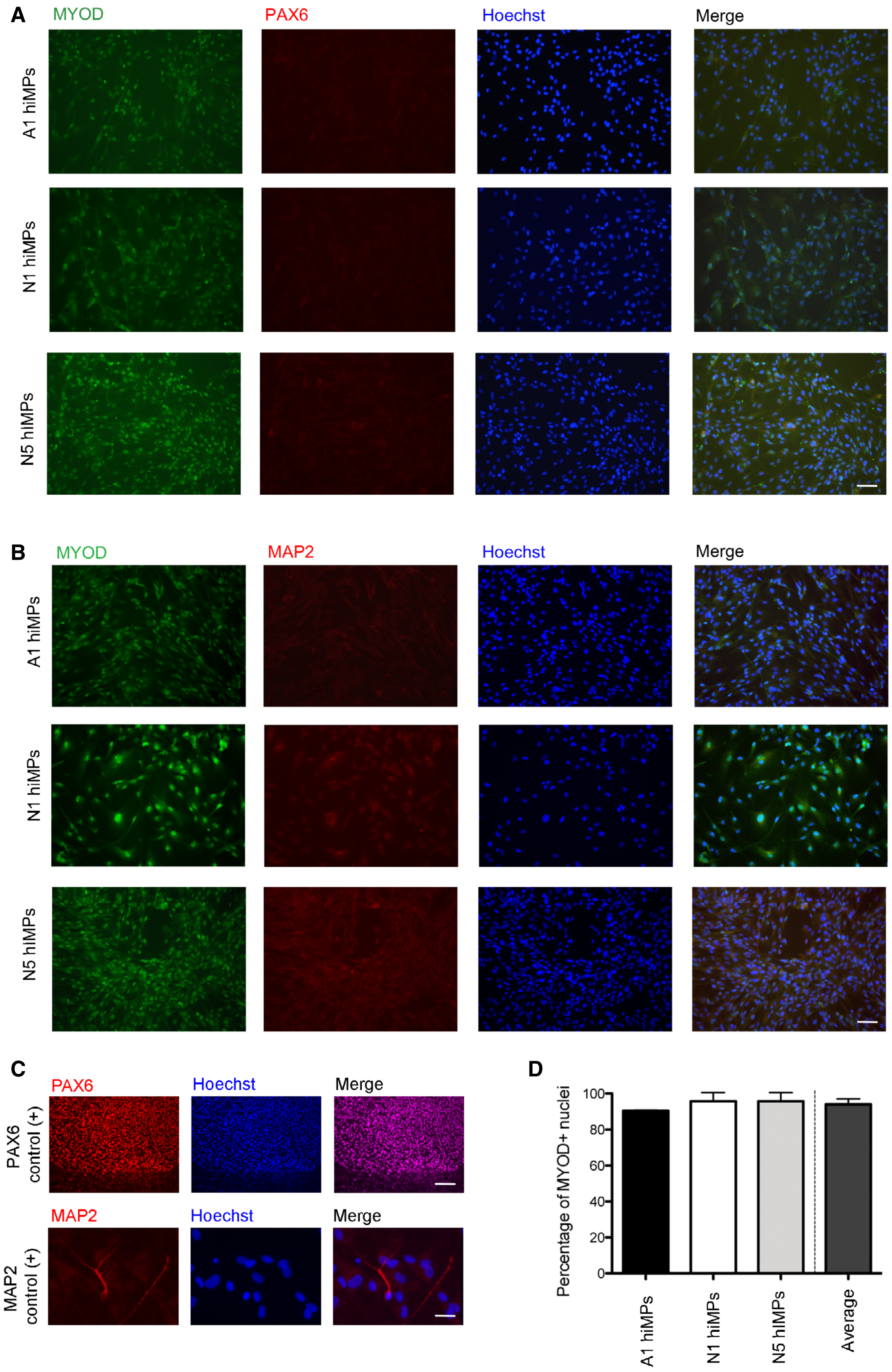
A
MYOD
PAX6
Hoechst
Merge
A1 hiMPs
N1 hiMPs
N5 hiMPs
B
MYOD
MAP2
Hoechst
Merge
A1 hiMPs
N1 hiMPs
N5 hiMPs
C
PAX6
Hoechst
Merge
PAX6 control (+)
MAP2
Hoechst
Merge
MAP2 control (+)
D
Percentage of MYOD+ nuclei
100
80
60
40
20
0
A1 hiMPs
N1 hiMPs
N5 hiMPs
Average

Figure EV1.

Figure EV2. Additional *in vitro* motility and migration analyses of treated and untreated hiMPs.

A Trajectory plots for visualisation of the migratory paths of untreated and treated cells that were exposed to either 1% BSA or DLL4 and PDGF-BB, respectively, over the course of the motility assay. Each line depicts the path of an individual cell.

B Visualisation of the motility state space of untreated and treated hiMPs using t-SNE (perplexity = 35).

C Hierarchical clustering of the first 30 principal components visualised with a t-SNE plot showing two clusters (Silhouette S_i = 0.19).

D Bar charts displaying the normalised motility feature values for comparison between conditions: untreated and DLL4 and PDGF-BB (left), cluster 1 and cluster 2 (right) (experimental replicates = 3; total 412 cells).

E Bar graph showing proportions of untreated and treated hiMPs within the two clusters. Hypothesis testing was performed with a chi-squared (χ^2) test.

F Trajectory plots for visualisation of hiMP migration after 24 h of treatment (top row), or 72 h of treatment (bottom row).

G, H t-SNE plots (perplexity = 35) for visualisation of the motility state space of hiMPs in two-dimensions (left). Cluster assignments after hierarchical clustering (S_i = 0.13 (24 h); S_i = 0.18 (72 h)). (H) Bar plots showing normalised motility features for both 24 h (top row) and 72 h (bottom row) conditions (experimental replicates = 3; total 876 cells and total 478 cells analysed for 24 and 72 h conditions, respectively.).

I Bar graph displaying proportions of untreated and DLL4 and PDGF-BB-treated hiMPs treated for 24 and 72 h. Hypothesis testing was performed with a chi-squared (χ^2) test.

J Bar graphs depict quantification of parameters obtained from single cell tracking analysed using TrackMate. Motility statistics were calculated for untreated (grey bars) and treated (white bars) hiMPs (experimental replicates = 3; error bars: SD). *P* values within figure: *t*-test.

Source data are available online for this figure.

© 2022 The Authors

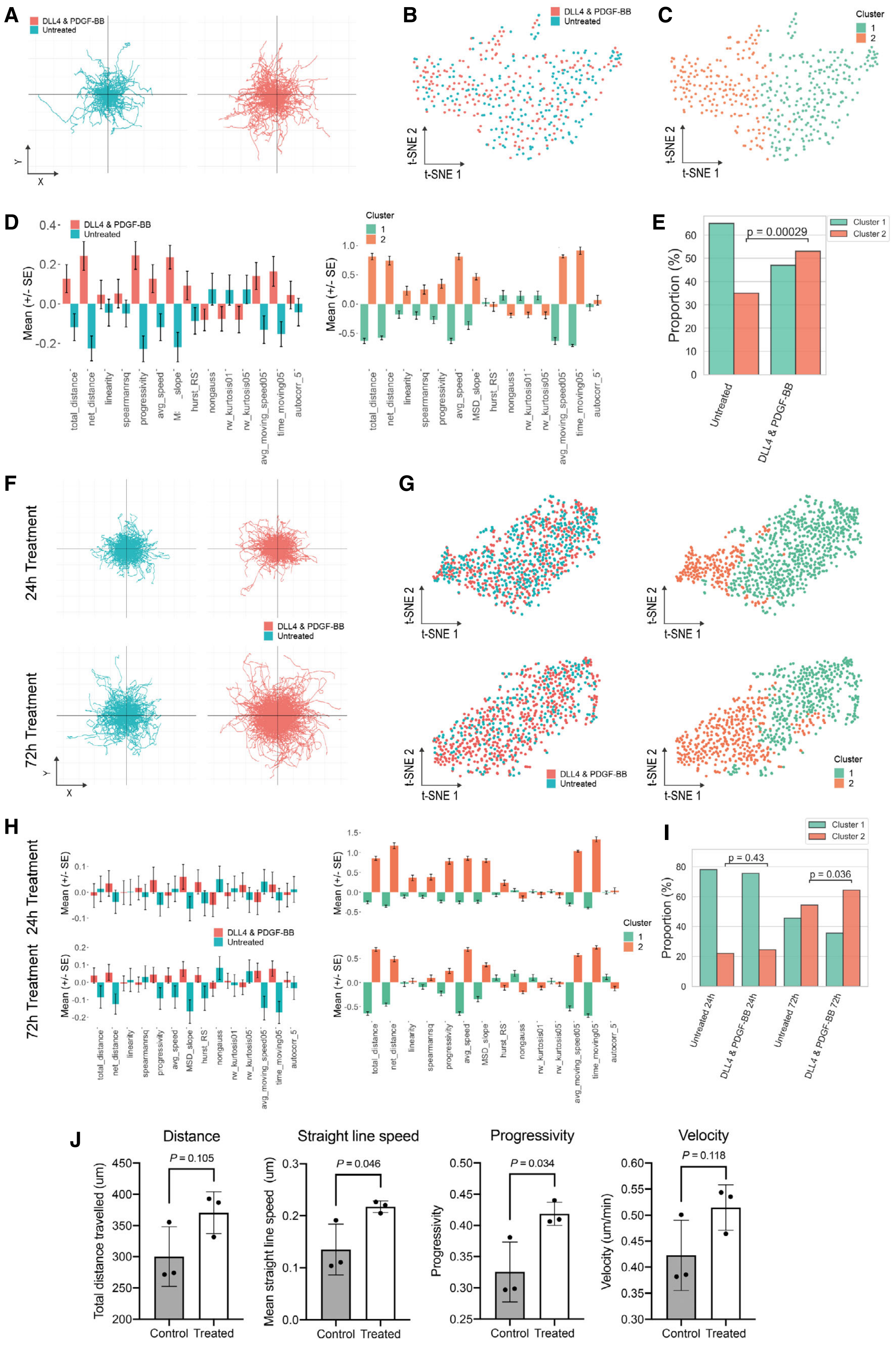
A
DLL4 & PDGF-BB
Untreated
B
C
Cluster
t-SNE 1
t-SNE 2
D
E
Proportion (%)
p = 0.00029
Cluster 1
Cluster 2
Untreated
DLL4 & PDGF-BB
F
24h Treatment
72h Treatment
G
H
I
p = 0.43
p = 0.036
Untreated 24h
DLL4 & PDGF-BB 24h
Untreated 72h
DLL4 & PDGF-BB 72h
Mean (+/- SE)
total_distance
net_distance
linearity
spearmanrsq
progressivity
avg_speed
MSD_slope
hurst_RS
nongauss
rw_kurtosis01
rw_kurtosis05
avg_moving_speed05
time_moving05
autocorr_5
J
Distance
P = 0.105
Total distance travelled (um)
Straight line speed
P = 0.046
Mean straight line speed (um)
Progressivity
P = 0.034
Velocity
P = 0.118
Velocity (um/min)
Control
Treated

Figure EV2.

Figure EV3. Additional *in silico* analyses and assessment of permeability of endothelialised 3D blood vessel-like microfluidic channels.

A Heatmaps displaying genes that are involved in negative regulation of cellular extravasation (left; GO: 002692) and positive regulation of cellular extravasation (right; GO: 002693). $*P < 0.05$.

B *P* value-adjusted hierarchical clustering heatmap showing a manually curated list of genes involved in enhanced trans-endothelial migration of cancer cells (*P* set al 0.05).

C Maximum intensity projection of a microfluidic channel immunostained for CD31 showing cobblestone-like morphology of HUVECs lining the top perfusion channel. Scale bar = 30 μm.

D Representative fluorescence images of 20 kDa FITC-conjugated dextran (top two rows) and 150 kDa TRITC-conjugated dextran (bottom two rows) added to the top perfusion channel of OrganoPlate® chips with and without 3D endothelial monolayers generated by HUVECs. Chips were imaged every 3 min for 15 min. Scale bar = 100 μm.

E Bar chart quantifying images shown in (D) using the normalised intensity calculated as the ratio of fluorescence between the ECM channel and top perfusion channel at each time point for cell-free and HUVEC chips containing 20 and 150 kDa fluorescent dextrans (technical replicates = 3; error bars; SD).

Source data are available online for this figure.

 © 2022 The Authors

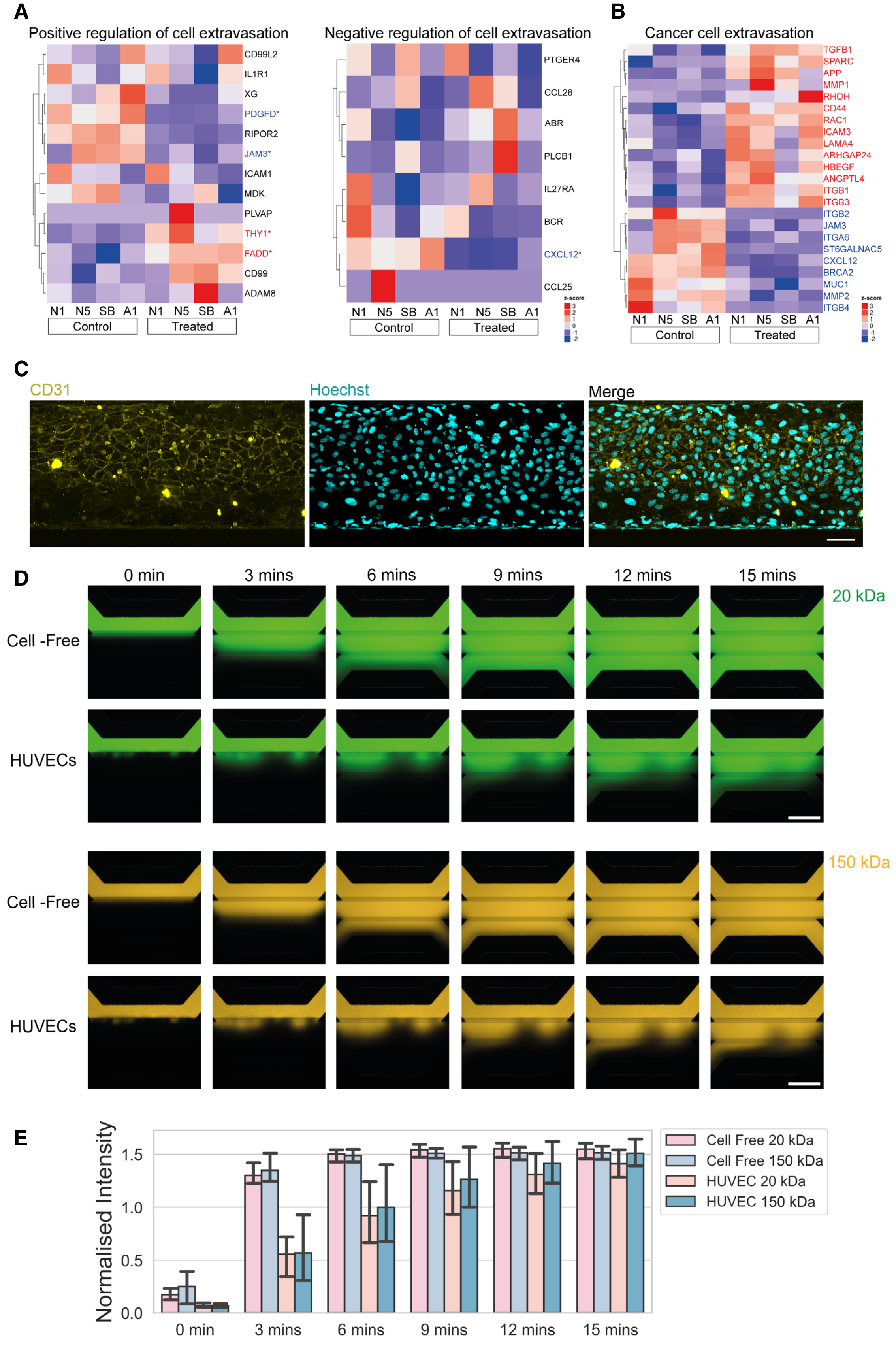
A
Positive regulation of cell extravasation
CD99L2
IL1R1
XG
PDGFD*
RIPOR2
JAM3*
ICAM1
MDK
PLVAP
THY1*
FADD*
CD99
ADAM8
N1 N5 SB A1 N1 N5 SB A1
Control
Treated
Negative regulation of cell extravasation
PTGER4
CCL28
ABR
PLCB1
IL27RA
BCR
CXCL12*
CCL25
N1 N5 SB A1 N1 N5 SB A1
Control
Treated
B
Cancer cell extravasation
TGFB1
SPARC
APP
MMP1
RHOH
CD44
RAC1
ICAM3
LAMA4
ARHGAP24
HBEGF
ANGPTL4
ITGB1
ITGB3
ITGB2
JAM3
ITGA6
ST6GALNAC5
CXCL12
BRCA2
MUC1
MMP2
ITGB4
N1 N5 SB A1 N1 N5 SB A1
Control
Treated
C
CD31
Hoechst
Merge
D
0 min
3 mins
6 mins
9 mins
12 mins
15 mins
20 kDa
Cell -Free
HUVECs
150 kDa
Cell -Free
HUVECs
E
Normalised Intensity
0.0
0.5
1.0
1.5
0 min
3 mins
6 mins
9 mins
12 mins
15 mins
Cell Free 20 kDa
Cell Free 150 kDa
HUVEC 20 kDa
HUVEC 150 kDa

Figure EV3.

APPENDIX

Assessing and enhancing migration of human myogenic progenitors using directed iPS cell differentiation and advanced tissue modelling

SungWoo Choi[1,2,#], Giulia Ferrari[2,#], Louise A. Moyle[2,§], Kirsty Mackinlay[2,Ø], Naira Naouar[3], Salma Jalal[2], Sara Benedetti[4,5], Christine Wells[6], Francesco Muntoni[5,7], Francesco Saverio Tedesco[1,2,7]*

[1]The Francis Crick Institute, 1 Midland Road, London, NW11AT, UK
[2]Department of Cell and Developmental Biology, University College London, London, WC1E6DE, UK
[3]Sorbonne Université, Institut de Biologie Paris Seine FR3631, Plateforme de bioinformatique ARTbio, Paris, France.
[4]UCL Great Ormond Street Institute of Child Health, University College London, London, UK
[5]National Institute for Health Research Great Ormond Street Hospital Biomedical Research Centre, London, UK
[6]Centre for Stem Cell Systems, The University of Melbourne, Melbourne, Victoria, Australia.
[7]Dubowitz Neuromuscular Centre, UCL Great Ormond Street Institute of Child Health & Great Ormond Street Hospital for Children, London, UK
[#]Equally contributing authors
[§]Current address: Institute of Biomedical Engineering, University of Toronto, Toronto, M5S3E1, Canada
[Ø]Current address: University of Cambridge, Department of Physiology, Development and Neuroscience, Cambridge CB23DY, UK
*Corresponding author (f.s.tedesco@ucl.ac.uk)

Table of Contents

- Appendix Figure S1. Top 50 differentially regulated genes in mMuSC-derived myoblasts, human myoblasts and hiMPs.
- Appendix Figure S2. Cross-comparison of top 50 differentially regulated genes of treated mMuSC-derived myoblasts in human myoblasts and hiMPs.
- Appendix Figure S3. Assessment of differentiation of hiMPs expanded in DMEM/F12-based in-house primary myoblast medium.
- Appendix Figure S4. Assessment of spontaneous differentiation of DLL4 & PDGF-BB treated hiMPs.
- Appendix Table S1. Top 50 genes responsible for variations of PC1 and 2 in the principal component analysis shown in Fig 2A.
- Appendix Table S2. Full list of genes of heatmaps shown in Appendix Fig S1 displaying 50 genes which exhibit either the greatest up- or down-regulation upon treatment with DLL4 & PDGF-BB in mMuSC (left), human myoblasts (centre) and hiMPs (right).
- Appendix Table S3. Table of ranked genes supplementing heatmaps presented in Appendix Fig S2.
- Appendix Table S4. Table of ranked genes accompanying heatmaps “Regulation of cell morphology”, “Proliferation of stem/myogenic cells” and “Leukocyte trans-endothelial migration” (Figures 3A, 3D and 4H, respectively).

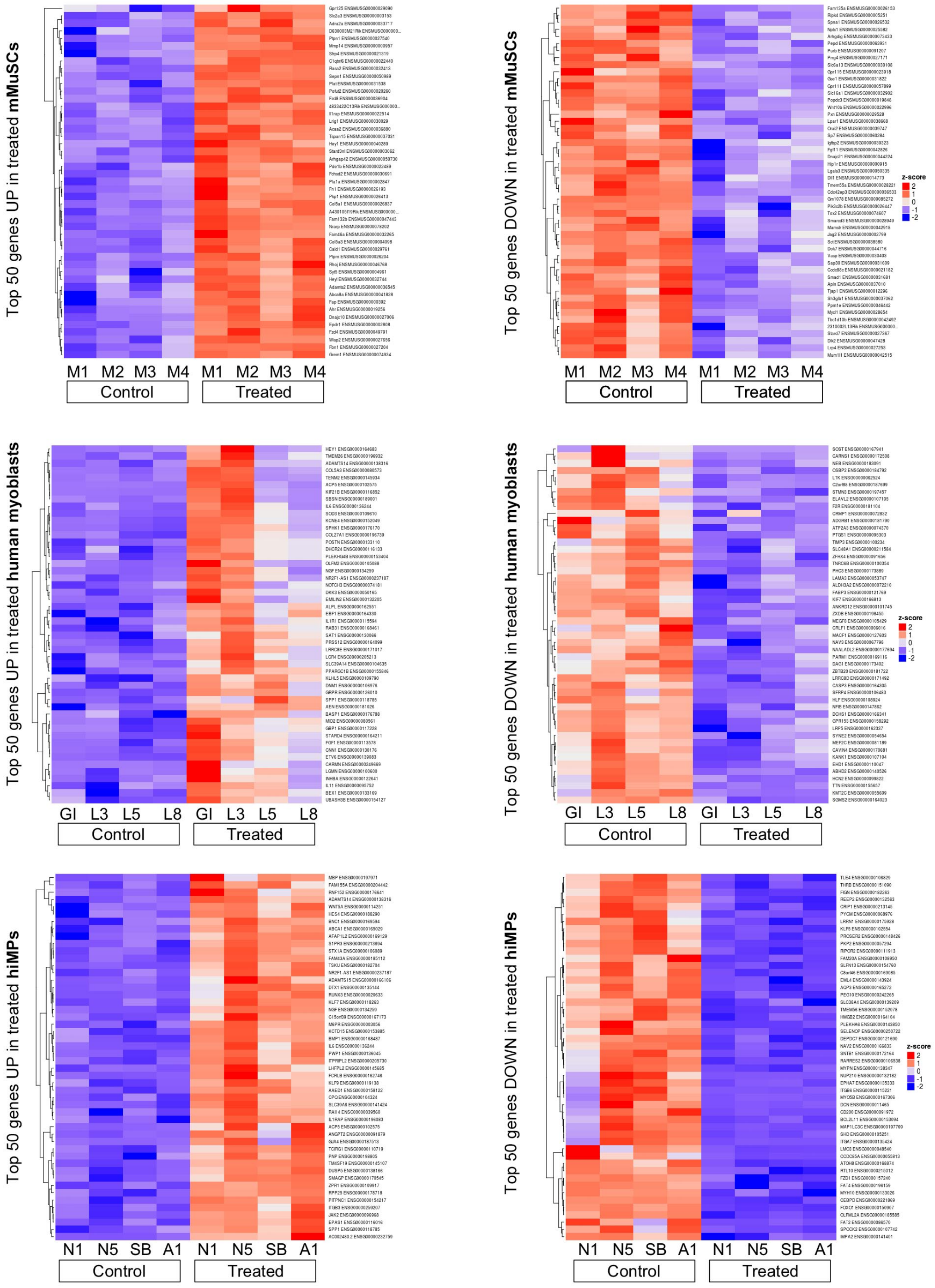

Appendix Figure S1. Top 50 differentially regulated genes in mMuSC-derived myoblasts, human myoblasts and hiMPs. Heatmaps displaying 50 genes which exhibit either the greatest up- (left) or down-regulation (right) upon treatment with DLL4 & PDGF-BB in mMuSC (top), human myoblasts (centre) and hiMPs (bottom). Additional information (details on gene list) in Appendix Table S3.

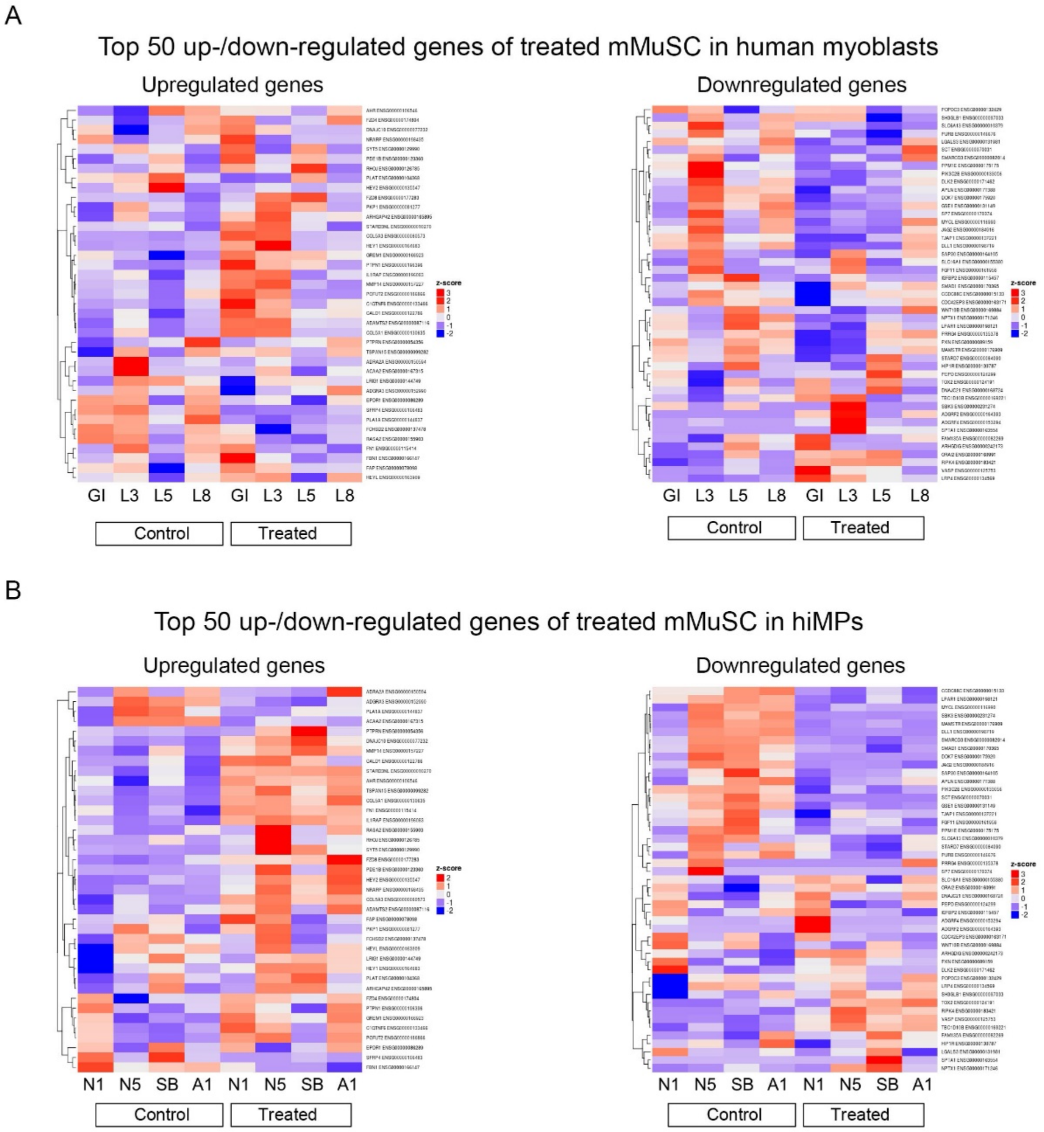

Appendix Figure S2. Cross-comparison of top 50 differentially regulated genes of treated mMuSC-derived myoblasts in human myoblasts and hiMPs. (A) Heatmaps of the top 50 up- (left) and down-regulated (right) genes of DLL4 & PDGF-BB-treated mMuSC-derived myoblasts in treated and untreated human myoblasts. **(B)** Heatmaps of the top 50 up- (left) and down-regulated (right) genes of DLL4 & PDGF-BB-treated mMuSC-derived myoblasts in treated and untreated hiMPs.

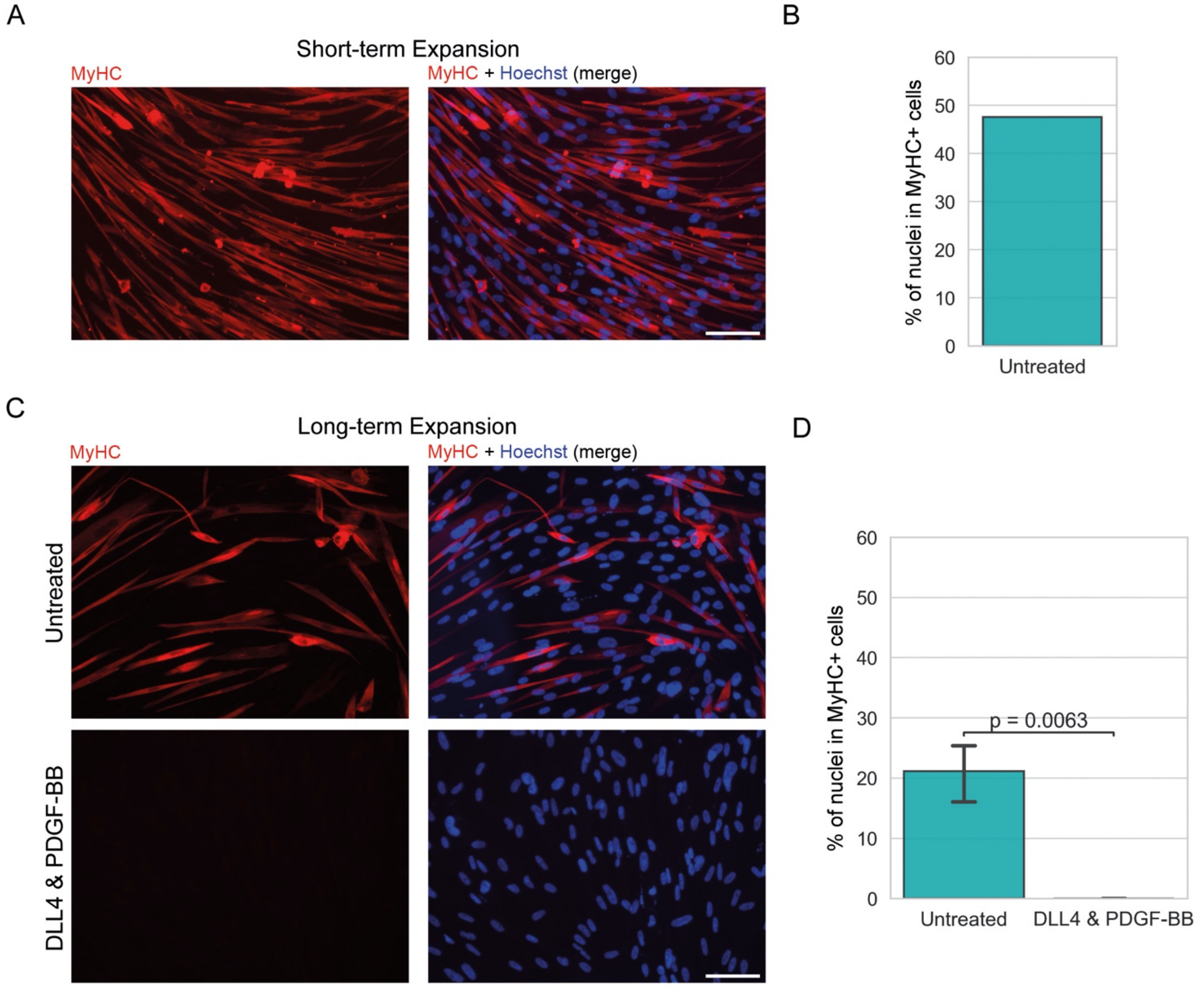

Appendix Figure S3. Assessment of differentiation of hiMPs expanded in DMEM/F12-based in-house primary myoblast medium. (A) Representative immunofluorescence images of hiMPs expanded in the in-house primary myoblast medium for 7 days (short term) (technical replicates = 2). Scale bar = 100µm. **(B)** Bar chart showing the quantification of terminal myogenic differentiation in (A). **(C)** Immunoflurescence images of hiMPs, 4 days post-differentiation into myotubes after expansion in the primary myoblast medium for longer than 7 days in either untreated or treated conditions. **(D)** Bar chart displaying the differentiation index for images in (C) (experimental replicates = 3). Scale bar = 100µm.

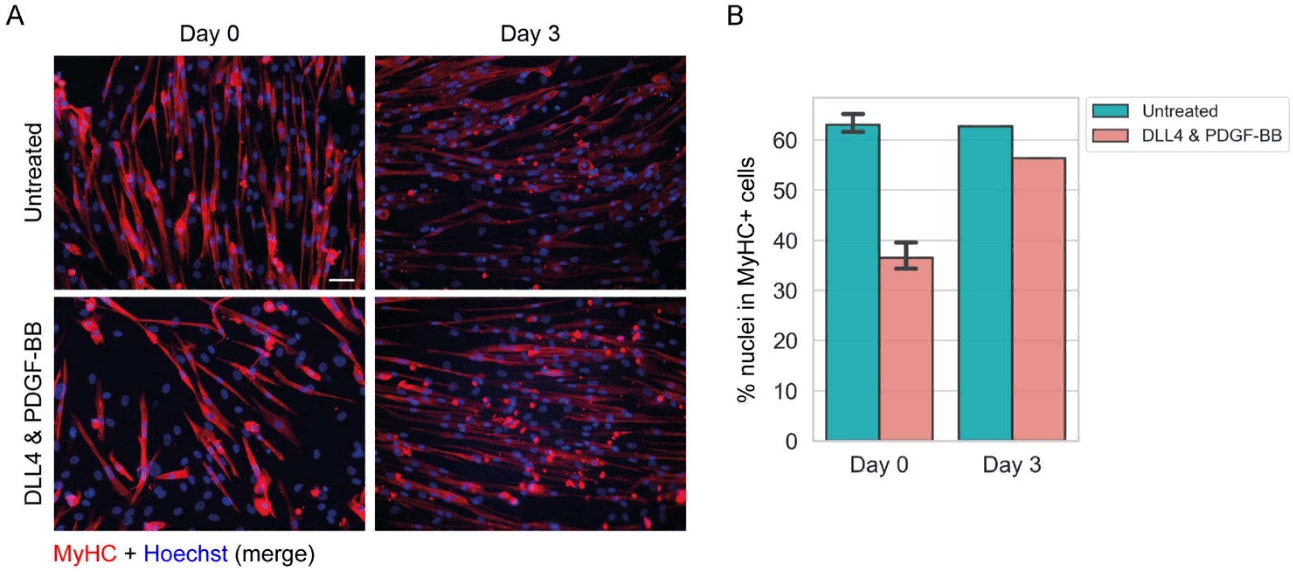

Appendix Figure S4. Assessment of spontaneous differentiation of DLL4 & PDGF-BB treated hiMPs. (A) Representative immunofluorescence images of untreated and DLL4 & PDGF-BB-treated hiMPs differentiated into myotubes for 4 days either immediately after 7 days of treatment or after proliferation of uncoated plastic dishes for 3 days. **(B)** Bar graph quantifying the differentiation of images in (A) using the differentiation index (Experimental replicates = 3 for Day 0; Experimental replicates = 1 for Day 3). Scale bar = 50µm.

	mMuSCs PC1 (45%)	mMuSCs PC2 (35%)	hMBs PC1(50%)	hMBs PC2 (25%)	hiMPs PC1 (63%)	hiMPs PC2 (17%)
1	Col15a1	Il33	COL5A3	XIST	TTN	EPGN
2	Col6a2	Actc1	TRH	RPS4Y1	MYBPH	CPA4
3	Col1a1	Flt1	TTN	DDX3Y	SYNPO2	SFRP1
4	Col6a1	Tek	SLC14A1	USP9Y	TNNI1	C3
5	Grem1	Jag1	OLFM2	NOS1	MYH8	UNC5B
6	Pdgfrb	Usp43	POSTN	KDM5D	TNNT2	SRGN
7	Heyl	Cyp2j6	ASS1	ZFY	CHRND	GDF6
8	Bgn	Myog	CRISPLD2	MYH3	CHRNA1	RGS4
9	Adamts2	Myo5b	ZNF469	LINC00261	KLHL41	OXTR
10	Sfrp4	Tnnt1	ALDH1A1	EIF1AY	SHD	IL1RL1
11	Col6a3	Tnnc2	STMN2	NLGN4Y	XIRP1	MSC
12	Col5a3	Atp2a1	PTGIS	ERAP2	ACTC1	GUCY1A2
13	Lrrc32	C1qtnf3	KLHL41	ELN	RYR1	HOXB9
14	Cdh11	Mylpf	MYH3	HLA-A	ENO3	INHBE
15	Nrarp	Grb10	IGFBP5	TXLNGY	CDH15	ARRDC4
16	Itgb3	Nefm	INA	COL11A1	UNC45B	TRIB3
17	Thy1	Sp7	NOTCH3	F13A1	FNDC5	CHRDL1
18	Igfbp7	Klhl41	SERPINE2	STMN2	TNNC1	DIO2
19	Pkp1	Smyd1	KCNE4	MMP1	MYH3	ZNF280D
20	Cyp1b1	Lepr	JAG1	EBF2	SRL	PRKG1
21	Mgp	Sema3d	LAMA3	CLGN	DES	GPRC5C
22	Serping1	Igf2	MYBPH	TNNI1	ACTA1	NLGN1
23	Ctgf	Meg3	NEB	UTY	SMYD1	NPY1R
24	Apbb1ip	Podxl	TENM2	SFRP1	MYH7	DDIT3
25	Fap	Synpo2l	NTSR1	RARRES2	VGLL2	CCDC3
26	Pcp4l1	Rian	TNFRSF1B	TNNT2	MYOZ2	MSC-AS1
27	Cldn4	Zdbf2	CD24	PLXNA4	MYOG	ADM2
28	Igfbp2	Myh3	HSPB7	KRT19	LMO7	DCC
29	Tcerg1l	Dchs1	TNNT2	IGFBP3	MYOD1	ENPP2
30	Abcb1a	Nefl	MYLPF	COLEC12	ACTN2	TSPYL5
31	Postn	Ldb3	INHBA	ZNF185	SFRP5	UNC13A
32	S1pr1	Fam84a	ADAMTS12	PRKY	F13A1	GATA6
33	Tagln	Sfrp4	NGFR	MYH7	CKM	LAMC2
34	Fn1	Sct	KIF21B	SIM2	NCAM1	TYW3
35	Trp53i11	Gpnmb	MYOD1	ANO1	MYLPF	OLFM2
36	Il6	Aqp5	SCG2	IL17RD	STAC3	SPON2
37	Itgb5	Myl1	L1CAM	CECR1	NEB	TUBB
38	Fam132b	Mstn	CKB	MYLPF	KLHL31	KLF4
39	Pde1b	H19	F3	TTTY15	ITGA7	BAALC
40	Scg2	Actn2	SFRP1	ACTN2	ERBB3	DPP4
41	Itga1	Mybpc1	COL4A1	ACTA1	GATM	LURAP1L
42	Cd248	Btc	MYH7	F2RL1	MYL4	TGM2
43	Cd28	Lmod3	ADAM12	IL13RA2	MYPN	JRK
44	Slit2	Myl4	ALDH3A1	HOXC10	B3GALT2	LGR4
45	Pappa	Ppfia4	ACTC1	KIAA1462	FGFR4	MCTP2
46	Gucy1a2	Srl	CCDC141	MYBPH	SHISA9	GJB2
47	Stc1	Mylk4	MYH8	CASQ2	NNAT	CXCL8
48	Klf9	Pdlim3	COL5A1	FLG	SORBS1	CPE
49	Tnfaip2	Nrep	ADAMTS2	ANKRD1	NPY	PCDH1
50	Mrc2	Acta1	MYOG	SLIT2	COL25A1	TBX2

Appendix Table S1. Top 50 genes responsible for variations of PC1 and 2 in the principal component analysis shown in Fig 2A.

50 top upregulated genes in mMuSCs	50 top up genes in human myoblasts	50 top upregulated genes in hiMPs
Gpr125, ENSMUSG00000029090	HEY1, ENSG00000164683	MBP, ENSG00000197971
Slc2a3, ENSMUSG00000003153	TMEM26, ENSG00000196932	FAM155A, ENSG00000204442
Adra2a, ENSMUSG00000033717	**ADAMTS14, ENSG00000138316**	RNF152, ENSG00000176641
D630003M21Rik, ENSMUSG00000037813	COL5A3, ENSG00000080573	**ADAMTS14, ENSG00000138316**
Ptpn1, ENSMUSG00000027540	TENM2, ENSG00000145934	WNT5A, ENSG00000114251
Mmp14, ENSMUSG00000000957	ACP5, ENSG00000102575	HES4, ENSG00000188290
Sfrp4, ENSMUSG00000021319	KIF21B, ENSG00000116852	BNC1, ENSG00000169594
C1qtnf6, ENSMUSG00000022440	SBSN, ENSG00000189001	ABCA1, ENSG00000165029
Rasa2, ENSMUSG00000032413	**IL6, ENSG00000136244**	AFAP1L2, ENSG00000169129
Sepn1, ENSMUSG00000050989	SOD3, ENSG00000109610	S1PR3, ENSG00000213694
Plat, ENSMUSG00000031538	KCNE4, ENSG00000152049	STX1A, ENSG00000106089
Pofut2, ENSMUSG00000020260	SPHK1, ENSG00000176170	FAM43A, ENSG00000185112
Fzd8, ENSMUSG00000036904	COL27A1, ENSG00000196739	TSKU, ENSG00000182704
4833422C13Rik, ENSMUSG00000074782	POSTN, ENSG00000133110	NR2F1-AS1, ENSG00000237187
Il1rap, ENSMUSG00000022514	DHCR24, ENSG00000116133	**ADAMTS15, ENSG00000166106**
Lrig1, ENSMUSG00000030029	PLEKHG4B, ENSG00000153404	DTX1, ENSG00000135144
Acaa2, ENSMUSG00000036880	OLFM2, ENSG00000105088	RUNX3, ENSG00000020633
Tspan15, ENSMUSG00000037031	**NGF, ENSG00000134259**	KLF7, ENSG00000118263
Hey1, ENSMUSG00000040289	**NR2F1-AS1, ENSG00000237187**	**NGF, ENSG00000134259**
Stard3nl, ENSMUSG00000003062	NOTCH3, ENSG00000074181	C15orf39, ENSG00000167173
Arhgap42, ENSMUSG00000050730	DKK3, ENSG00000050165	M6PR, ENSG00000003056
Pde1b, ENSMUSG00000022489	EMILIN2, ENSG00000132205	KCTD15, ENSG00000153885
Fchsd2, ENSMUSG00000030691	ALPL, ENSG00000162551	BMP1, ENSG00000168487
Pla1a, ENSMUSG00000002847	EBF1, ENSG00000164330	**IL6, ENSG00000136244**
Fn1, ENSMUSG00000026193	IL1R1, ENSG00000115594	PWP1, ENSG00000136045
Pkp1, ENSMUSG00000026413	RAB31, ENSG00000168461	ITPRIPL2, ENSG00000205730
Col5a1, ENSMUSG00000026837	SAT1, ENSG00000130066	LHFPL2, ENSG00000145685
A430105I19Rik, ENSMUSG00000045838	PRSS12, ENSG00000164099	FCRLB, ENSG00000162746
Fam132b, ENSMUSG00000047443	LRRC8E, ENSG00000171017	KLF9, ENSG00000119138
Nrarp, ENSMUSG00000078202	LGR4, ENSG00000205213	AAED1, ENSG00000158122
Fam46a, ENSMUSG00000032265	SLC39A14, ENSG00000104635	CPQ, ENSG00000104324
Col5a3, ENSMUSG00000004098	PPARGC1B, ENSG00000155846	SLC39A6, ENSG00000141424
Cald1, ENSMUSG00000029761	KLHL5, ENSG00000109790	RAI14, ENSG00000039560
Ptprn, ENSMUSG00000026204	DNM1, ENSG00000106976	IL1RAP, ENSG00000196083
Rhoj, ENSMUSG00000046768	GRPR, ENSG00000126010	ACP5, ENSG00000102575
Syt5, ENSMUSG00000004961	**SPP1, ENSG00000118785**	ANGPT2, ENSG00000091879
Heyl, ENSMUSG00000032744	AEN, ENSG00000181026	GJA4, ENSG00000187513
Adamts2, ENSMUSG00000036545	BASP1, ENSG00000176788	TCIRG1, ENSG00000110719
Abca8a, ENSMUSG00000041828	MID2, ENSG00000080561	PNP, ENSG00000198805
Fap, ENSMUSG00000000392	GBP1, ENSG00000117228	TM4SF19, ENSG00000145107
Ahr, ENSMUSG00000019256	STARD4, ENSG00000164211	DUSP5, ENSG00000138166
Dnajc10, ENSMUSG00000027006	FGF1, ENSG00000113578	SMAGP, ENSG00000170545
Epdr1, ENSMUSG00000002808	CNN1, ENSG00000130176	ZPR1, ENSG00000109917
Fzd4, ENSMUSG00000049791	ETV6, ENSG00000139083	RPP25, ENSG00000178718
Wisp2, ENSMUSG00000027656	CARMN, ENSG00000249669	PITPNC1, ENSG00000154217
Fbn1, ENSMUSG00000027204	LGMN, ENSG00000100600	ITGB3, ENSG00000259207
Grem1, ENSMUSG00000074934	INHBA, ENSG00000122641	JAK2, ENSG00000096968
*Cfh ENSMUSG00000026365	IL11, ENSG00000095752	EPAS1, ENSG00000116016
*AW011738, ENSMUSG00000078349	BEX1, ENSG00000133169	**SPP1, ENSG00000118785**
*Hey2, ENSMUSG00000019789	UBASH3B, ENSG00000154127	AC002480.2, ENSG00000232759
**50 top downregulated genes in mMuSCs**	**50 top downregulated genes in human myoblasts**	**50 top downregulated genes in hiMPs**
Fam135a, ENSMUSG00000026153	SOST, ENSG00000167941	TLE4, ENSG00000106829,,
Ripk4, ENSMUSG00000005251	CARNS1, ENSG00000172508	THRB, ENSG00000151090
Spna1, ENSMUSG00000026532	NEB, ENSG00000183091	FIGN, ENSG00000182263
Nptx1, ENSMUSG00000025582	OSBP2, ENSG00000184792	REEP2, ENSG00000132563
Arhgdig, ENSMUSG00000073433	LTK, ENSG00000062524	CRIP1, ENSG00000213145
Pepd, ENSMUSG00000063931	C2orf88, ENSG00000187699	PYGM, ENSG00000068976
Purb, ENSMUSG00000091207	STMN3, ENSG00000197457	LRRN1, ENSG00000175928
Prrg4, ENSMUSG00000027171	ELAVL2, ENSG00000107105	KLF5, ENSG00000102554
Slc6a13, ENSMUSG00000030108	F2R, ENSG00000181104	PROSER2, ENSG00000148426
Gpr115, ENSMUSG00000023918	CRMP1, ENSG00000072832	PKP2, ENSG00000057294
Gse1, ENSMUSG00000031822	ADGRB1, ENSG00000181790	RIPOR2, ENSG00000111913
Gpr111, ENSMUSG00000057899	ATP2A3, ENSG00000074370	FAM20A, ENSG00000108950
Slc16a1, ENSMUSG00000032902	PTGS1, ENSG00000095303	SLFN13, ENSG00000154760
Popdc3, ENSMUSG00000019848	TIMP3, ENSG00000100234	C8orf46, ENSG00000169085
Wnt10b, ENSMUSG00000022996	SLC48A1, ENSG00000211584	EML4, ENSG00000143924
Pxn, ENSMUSG00000029528	ZFHX4, ENSG00000091656	AQP3, ENSG00000165272
Lpar1, ENSMUSG00000038668	TNRC6B, ENSG00000100354	PEG10, ENSG00000242265
Orai2, ENSMUSG00000039747	PHC3, ENSG00000173889	SLC38A4, ENSG00000139209
Sp7, ENSMUSG00000060284	LAMA3, ENSG00000053747	TMEM56, ENSG00000152078
Igfbp2, ENSMUSG00000039323	ALDH3A2, ENSG00000072210	HMGB2, ENSG00000164104
Fgf11, ENSMUSG00000042826	FABP3, ENSG00000121769	PLEKHA6, ENSG00000143850
Dnajc21, ENSMUSG00000044224	KIF7, ENSG00000166813	SELENOP, ENSG00000250722
Hip1r, ENSMUSG00000000915	ANKRD12, ENSG00000101745	DEPDC7, ENSG00000121690
Lgals3, ENSMUSG00000050335	ZXDB, ENSG00000198455	NAV2, ENSG00000166833
Dll1, ENSMUSG00000014773	MEGF8, ENSG00000105429	SNTB1, ENSG00000172164
Tmem55a, ENSMUSG00000028221	CRLF1, ENSG00000006016	RARRES2, ENSG00000106538
Cdc42ep3, ENSMUSG00000036533	MACF1, ENSG00000127603	MYPN, ENSG00000138347
Gm1078, ENSMUSG00000085272	NAV3, ENSG00000067798	NUP210, ENSG00000132182
Pik3c2b, ENSMUSG00000026447	NAALADL2, ENSG00000177694	EPHA7, ENSG00000135333
Tox2, ENSMUSG00000074607	PARM1, ENSG00000169116	ITGB6, ENSG00000115221
Smarcd3, ENSMUSG00000028949	DAG1, ENSG00000173402	MYO5B, ENSG00000167306
Mamstr, ENSMUSG00000042918	ZBTB20, ENSG00000181722	DCN, ENSG00000011465
Jag2, ENSMUSG00000002799	LRRC8D, ENSG00000171492	CD200, ENSG00000091972
Sct, ENSMUSG00000038580	CASP3, ENSG00000164305	BCL2L11, ENSG00000153094
Dok7, ENSMUSG00000044716	SFRP4, ENSG00000106483	MAP1LC3C, ENSG00000197769
Vasp, ENSMUSG00000030403	HLF, ENSG00000108924	SHD, ENSG00000105251
Sap30, ENSMUSG00000031609	NFIB, ENSG00000147862	ITGA7, ENSG00000135424
Ccdc88c, ENSMUSG00000021182	DCHS1, ENSG00000166341	LMO3, ENSG00000048540
Smad1, ENSMUSG00000031681	GPR153, ENSG00000158292	CCDC85A, ENSG00000055813
Apln, ENSMUSG00000037010	LRP5, ENSG00000162337	ATOH8, ENSG00000168874
Tjap1, ENSMUSG00000012296	SYNE2, ENSG00000054654	RTL10, ENSG00000215012
Sh3glb1, ENSMUSG00000037062	MEF2C, ENSG00000081189	FZD1, ENSG00000157240
Ppm1e, ENSMUSG00000046442	CAVIN4, ENSG00000170681	FAT4, ENSG00000196159
Mycl1, ENSMUSG00000028654	KANK1, ENSG00000107104	MYH10, ENSG00000133026
Tbc1d10b, ENSMUSG00000042492	EHD1, ENSG00000110047	CEBPD, ENSG00000221869
2310002L13Rik, ENSMUSG00000024512	ABHD2, ENSG00000140526	FOXO1, ENSG00000150907
Stard7, ENSMUSG00000027367	HCN2, ENSG00000099822	OLFML2A, ENSG00000185585
Dlk2, ENSMUSG00000047428	TTN, ENSG00000155657	FAT2, ENSG00000086570
Lrp4, ENSMUSG00000027253	KMT2C, ENSG00000055609	SPOCK2, ENSG00000107742
Mum1l1, ENSMUSG00000042515	SGMS2, ENSG00000164023	IMPA2, ENSG00000141401

Appendix Table S2. Full list of genes of heatmaps shown in Appendix Fig S1 displaying 50 genes which exhibit either the greatest up- or down-regulation upon treatment with DLL4 & PDGF-BB in mMuSC (left), human myoblasts (centre) and hiMPs (right). *Genes not shown in heatmap due to N/A rows resulting from Stemformatics analysis. Bold font: common genes in human lists.

Ranked genes							
**Top 50 upregulated genes of treated mMuSC in human myoblasts**		**Top 50 downregulated genes of treated mMuSC in human myoblasts**		**Top 50 upregulated genes of treated mMuSC in hiMPs**		**Top 50 downregulated genes of treated mMuSC in hiMPs**	
Gene	Probe	Gene	Probe	Gene	Probe	Gene	Probe
AHR	ENSG00000106546	POPDC3	ENSG00000132429	ADRA2A	ENSG00000150594	CCDC88C	ENSG00000015133
FZD4	ENSG00000174804	SH3GLB1	ENSG00000097033	ADGRA3	ENSG00000152990	LPAR1	ENSG00000198121
DNAJC10	ENSG00000077232	SLC6A13	ENSG00000010379	PLA1A	ENSG00000144837	MYCL	ENSG00000116990
NRARP	ENSG00000198435	PURB	ENSG00000146676	ACAA2	ENSG00000167315	SBK3	ENSG00000231274
SYT5	ENSG00000129990	LGALS3	ENSG00000131981	PTPRN	ENSG00000054356	MAMSTR	ENSG00000176909
PDE1B	ENSG00000123360	SCT	ENSG00000070031	DNAJC10	ENSG00000077232	DLL1	ENSG00000198719
RHOJ	ENSG00000126785	SMARCD3	ENSG00000082014	MMP14	ENSG00000157227	SMARCD3	ENSG00000082014
PLAT	ENSG00000104368	PPM1E	ENSG00000175175	CALD1	ENSG00000122786	SMAD1	ENSG00000170365
HEY2	ENSG00000135547	PIK3C2B	ENSG00000133056	STARD3NL	ENSG00000010270	DOK7	ENSG00000175920
FZD8	ENSG00000177283	DLK2	ENSG00000171462	AHR	ENSG00000106546	JAG2	ENSG00000184916
PKP1	ENSG00000081277	APLN	ENSG00000171388	TSPAN15	ENSG00000099282	SAP30	ENSG00000164105
ARHGAP42	ENSG00000165895	DOK7	ENSG00000175920	COL5A1	ENSG00000130635	APLN	ENSG00000171388
STARD3NL	ENSG00000010270	GSE1	ENSG00000131149	FN1	ENSG00000115414	PIK3C2B	ENSG00000133056
COL5A3	ENSG00000080573	SP7	ENSG00000170374	IL1RAP	ENSG00000196083	SCT	ENSG00000070031
HEY1	ENSG00000164683	MYCL	ENSG00000116990	RASA2	ENSG00000155903	GSE1	ENSG00000131149
GREM1	ENSG00000166923	JAG2	ENSG00000184916	RHOJ	ENSG00000126785	TJAP1	ENSG00000137221
PTPN1	ENSG00000196396	TJAP1	ENSG00000137221	SYT5	ENSG00000129990	FGF11	ENSG00000161958
IL1RAP	ENSG00000196083	DLL1	ENSG00000198719	FZD8	ENSG00000177283	PPM1E	ENSG00000175175
MMP14	ENSG00000157227	SAP30	ENSG00000164105	PDE1B	ENSG00000123360	SLC6A13	ENSG00000010379
POFUT2	ENSG00000186866	SLC16A1	ENSG00000155380	HEY2	ENSG00000135547	STARD7	ENSG00000084090
C1QTNF6	ENSG00000133466	FGF11	ENSG00000161958	NRARP	ENSG00000198435	PURB	ENSG00000146676
CALD1	ENSG00000122786	IGFBP2	ENSG00000115457	COL5A3	ENSG00000080573	PRRG4	ENSG00000135378
ADAMTS2	ENSG00000087116	SMAD1	ENSG00000170365	ADAMTS2	ENSG00000087116	SP7	ENSG00000170374
COL5A1	ENSG00000130635	CCDC88C	ENSG00000015133	FAP	ENSG00000078098	SLC16A1	ENSG00000155380
PTPRN	ENSG00000054356	CDC42EP3	ENSG00000163171	PKP1	ENSG00000081277	ORAI2	ENSG00000160991
TSPAN15	ENSG00000099282	WNT10B	ENSG00000169884	FCHSD2	ENSG00000137478	DNAJC21	ENSG00000168724
ADRA2A	ENSG00000150594	NPTX1	ENSG00000171246	HEYL	ENSG00000163909	PEPD	ENSG00000124299
ACAA2	ENSG00000167315	LPAR1	ENSG00000198121	LRIG1	ENSG00000144749	IGFBP2	ENSG00000115457
LRIG1	ENSG00000144749	PRRG4	ENSG00000135378	HEY1	ENSG00000164683	ADGRF4	ENSG00000153294
ADGRA3	ENSG00000152990	PXN	ENSG00000089159	PLAT	ENSG00000104368	ADGRF2	ENSG00000164393
EPDR1	ENSG00000086289	MAMSTR	ENSG00000176909	ARHGAP42	ENSG00000165895	CDC42EP3	ENSG00000163171
SFRP4	ENSG00000106483	STARD7	ENSG00000084090	FZD4	ENSG00000174804	WNT10B	ENSG00000169884
PLA1A	ENSG00000144837	HIP1R	ENSG00000130787	PTPN1	ENSG00000196396	ARHGDIG	ENSG00000242173
FCHSD2	ENSG00000137478	PEPD	ENSG00000124299	GREM1	ENSG00000166923	PXN	ENSG00000089159
RASA2	ENSG00000155903	TOX2	ENSG00000124191	C1QTNF6	ENSG00000133466	DLK2	ENSG00000171462
FN1	ENSG00000115414	DNAJC21	ENSG00000168724	POFUT2	ENSG00000186866	POPDC3	ENSG00000132429
FBN1	ENSG00000166147	TBC1D10B	ENSG00000169221	EPDR1	ENSG00000086289	LRP4	ENSG00000134569
FAP	ENSG00000078098	SBK3	ENSG00000231274	SFRP4	ENSG00000106483	SH3GLB1	ENSG00000097033
HEYL	ENSG00000163909	ADGRF2	ENSG00000164393	FBN1	ENSG00000166147	TOX2	ENSG00000124191
Fam132b	*	ADGRF4	ENSG00000153294	Fam132b	*	RIPK4	ENSG00000183421
4833422C13Rik	*	SPTA1	ENSG00000163554	4833422C13Rik	*	VASP	ENSG00000125753
Wisp2	*	FAM135A	ENSG00000082269	Wisp2	*	TBC1D10B	ENSG00000169221
A430105I19Rik	*	ARHGDIG	ENSG00000242173	A430105I19Rik	*	FAM135A	ENSG00000082269
Slc2a3	*	ORAI2	ENSG00000160991	Slc2a3	*	HIP1R	ENSG00000130787
Abca8a	*	RIPK4	ENSG00000183421	Abca8a	*	LGALS3	ENSG00000131981
Cfh	*	VASP	ENSG00000125753	Cfh	*	SPTA1	ENSG00000163554
Fam46a	*	LRP4	ENSG00000134569	Fam46a	*	NPTX1	ENSG00000171246
Sepn1	*	Tmem55a	*	Sepn1	*	Tmem55a	*
AW011738	*	Mum1l1	*	AW011738	*	Mum1l1	*
D630003M21Rik	*	Dynap	*	D630003M21Rik	*	Dynap	*

Appendix Table S3. Table of ranked genes supplementing heatmaps presented in Appendix Fig S2: “Top 50 upregulated genes of treated mMuSC-derived myoblasts in human myoblasts” (Appendix Fig S2A) (left); “Top 50 downregulated genes of treated mMuSC-derived myoblasts in human myoblasts” (Appendix Fig S2A) (right); “Top 50 upregulated genes of treated mMuSC-derived myoblasts in hiMPs” (Appendix Fig S2B) (left); “Top 50 downregulated genes of treated mMuSC-derived myoblasts in hiMPs” (Appendix Fig S2B) (right). *no human orthologue found.

Ranked genes					
Regulation of cell morphology		Proliferation of stem/myogenic Cells		Leukocyte trans-endothelial migration	
Gene	Probe	Gene	Probe	Gene	Probe
MYO10	ENSG00000145555	MMP9	ENSG00000100985	TXK	ENSG00000074966
VEGFA	ENSG00000112715	PTGIR	ENSG00000160013	ACTN3	ENSG00000248746
RHOQ	ENSG00000119729	VEGFA	ENSG00000112715	VAV3	ENSG00000134215
FN1	ENSG00000115414	PDGFRB	ENSG00000113721	CLDN5	ENSG00000184113
KIT	ENSG00000157404	NGF	ENSG00000134259	ITGB2	ENSG00000160255
RAC3	ENSG00000169750	IRAK1	ENSG00000184216	RASSF5	ENSG00000266094
WIPF1	ENSG00000115935	TGFB1	ENSG00000105329	ACTN2	ENSG00000077522
SH3KBP1	ENSG00000147010	C3AR1	ENSG00000171860	JAM2	ENSG00000154721
RHOC	ENSG00000155366	KITLG	ENSG00000049130	PIK3R1	ENSG00000145675
HEXB	ENSG00000049860	GNAI3	ENSG00000065135	JAM3	ENSG00000166086
CDC42EP4	ENSG00000179604	SIRT6	ENSG00000077463	MYLPF	ENSG00000180209
RAC1	ENSG00000136238	JAK2	ENSG00000096968	CTNNA3	ENSG00000183230
MSN	ENSG00000147065	ITGB3	ENSG00000259207	GNAI1	ENSG00000127955
PLXND1	ENSG00000004399	SNAI2	ENSG00000019549	CXCL12	ENSG00000107562
IL6	ENSG00000136244	IL6	ENSG00000136244	MYL2	ENSG00000111245
FMNL3	ENSG00000161791	NOTCH3	ENSG00000074181	MMP9	ENSG00000100985
MYH9	ENSG00000100345	MYC	ENSG00000136997	PLCG2	ENSG00000197943
KIF3A	ENSG00000131437	NOS3	ENSG00000164867	CLDN7	ENSG00000181885
FBLIM1	ENSG00000162458	ILK	ENSG00000166333	ESAM	ENSG00000149564
CDC42EP1	ENSG00000128283	TRIB1	ENSG00000173334	ARHGAP35	ENSG00000160007
DLC1	ENSG00000164741	IL12A	ENSG00000168811	RHOH	ENSG00000168421
ARHGAP35	ENSG00000160007	HBEGF	ENSG00000113070	ACTN1	ENSG00000072110
ARAP3	ENSG00000120318	CAV1	ENSG00000105974	ITGB1	ENSG00000150093
RHOG	ENSG00000177105	CNN1	ENSG00000130176	VASP	ENSG00000125753
RHOD	ENSG00000173156	FGF9	ENSG00000102678	THY1	ENSG00000154096
SEMA4D	ENSG00000187764	BMPR1A	ENSG00000107779	RAP1B	ENSG00000127314
LPAR1	ENSG00000198121	HMGB2	ENSG00000164104	RAC1	ENSG00000136238
MYH10	ENSG00000133026	BMP4	ENSG00000125378	GNAI3	ENSG00000065135
RHOBTB3	ENSG00000164292	RBPJ	ENSG00000168214	MSN	ENSG00000147065
PHIP	ENSG00000146247	CTNNBIP1	ENSG00000178585		
S100A13	ENSG00000189171	SOX15	ENSG00000129194		
PALMD	ENSG00000099260	MYOD1	ENSG00000129152		
ITGA7	ENSG00000135424	MAP3K5	ENSG00000197442		
PALM2	ENSG00000243444	PIK3R1	ENSG00000145675		
EPB41L3	ENSG00000082397	MYOG	ENSG00000122180		
WIPF3	ENSG00000122574	KLHL41	ENSG00000239474		
SEMA3E	ENSG00000170381	SMARCD3	ENSG00000082014		
KDR	ENSG00000128052	MAGI1	ENSG00000151276		
PLXNB1	ENSG00000164050	CAMK2D	ENSG00000145349		
		MEF2C	ENSG00000081189		
		MEGF10	ENSG00000145794		
		PPARGC1A	ENSG00000109819		
		PDE1A	ENSG00000115252		
		ANGPT1	ENSG00000154188		
		MMP2	ENSG00000087245		
		RGS5	ENSG00000143248		
		IL18	ENSG00000150782		
		PDGFD	ENSG00000170962		
		AKR1B1	ENSG00000085662		
		MNAT1	ENSG00000020426		
		SKP2	ENSG00000145604		
		EGR1	ENSG00000120738		
		TGM2	ENSG00000198959		
		DNMT1	ENSG00000130816		
		ASPM	ENSG00000066279		
		ORC1	ENSG00000085840		

Appendix Table S4. Table of ranked genes accompanying heatmaps “Regulation of cell morphology”, “Proliferation of stem/myogenic cells” and “Leukocyte trans-endothelial migration” (Fig 3A, 3D and 4H, respectively).
