# Supplementary figures and images for: Assessing and enhancing migration of human myogenic progenitors using directed iPS cell differentiation and advanced tissue modelling
Source: EMBO Mol Med. 2022 Sep 26;14(10):e14526. doi: 10.15252/emmm.202114526 (PMC9549733; doi:10.15252/emmm.202114526)

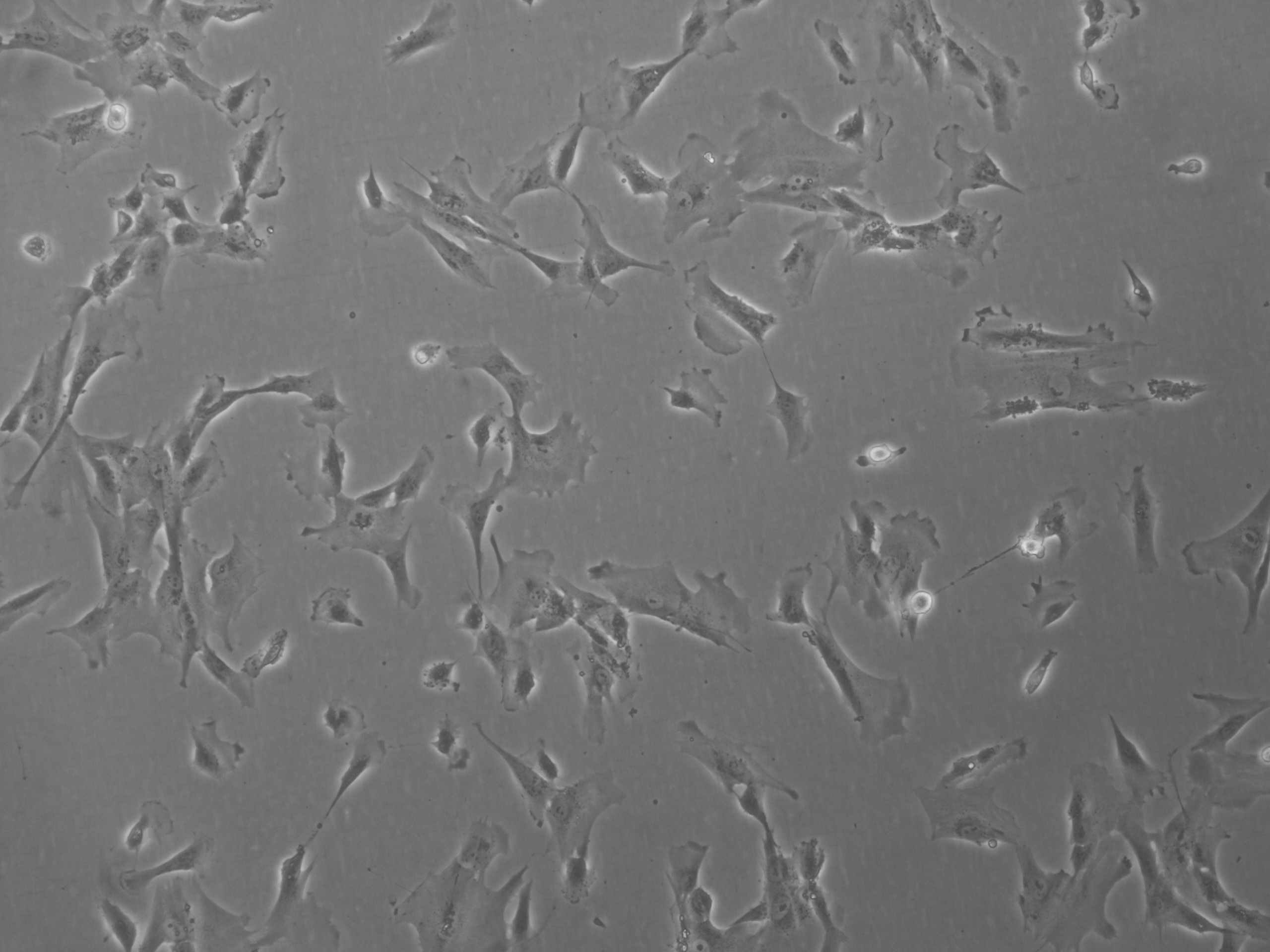

Supplement: Supplementary file 9 — Source Data for Figure 3 [file EMMM-14-e14526-s011.zip › Figure 3/Figure 3 Images/Figure 3B/Untreated 2.tif]

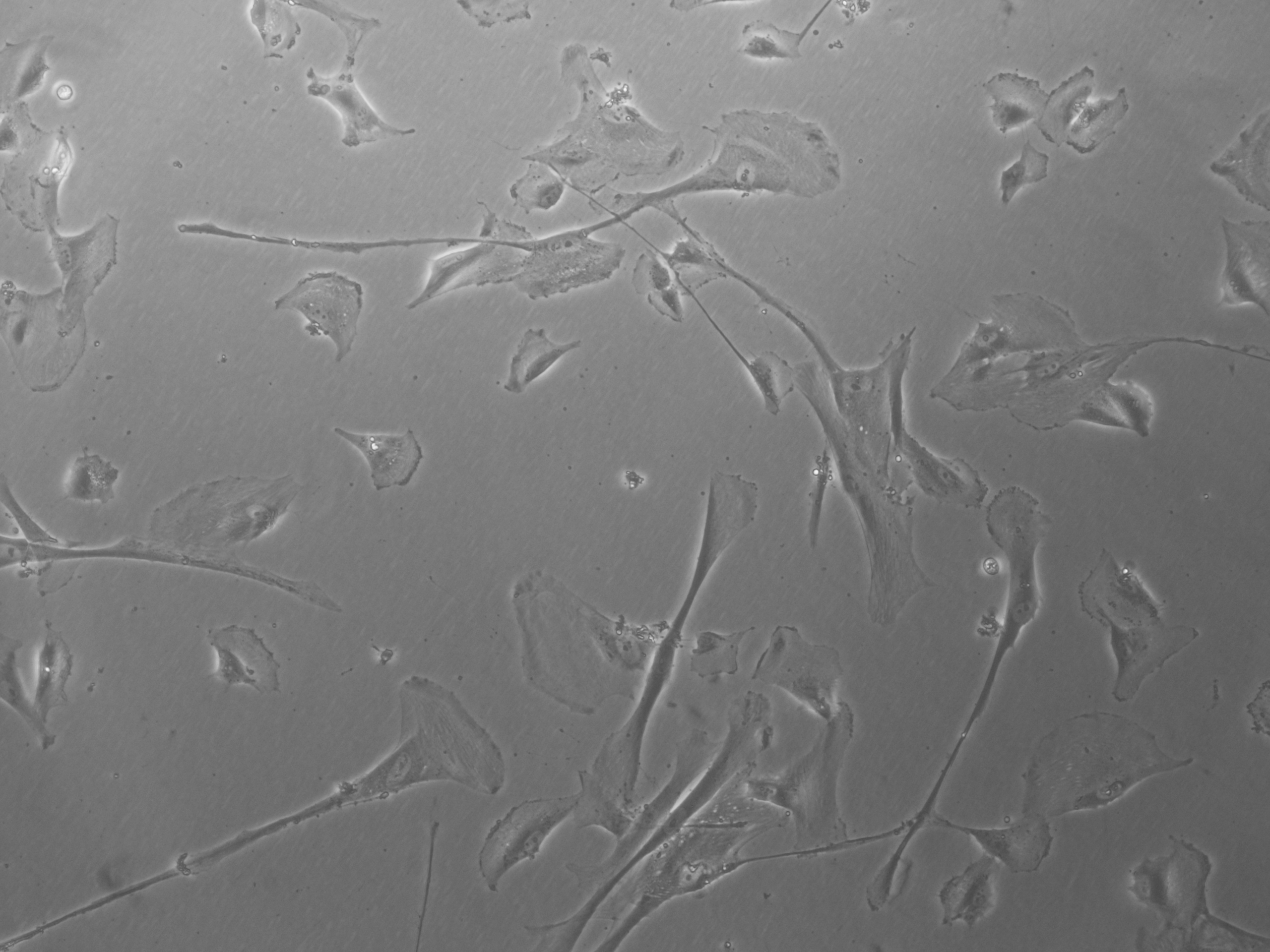

Supplement: Supplementary file 9 — Source Data for Figure 3 [file EMMM-14-e14526-s011.zip › Figure 3/Figure 3 Images/Figure 3B/Treated 2.tif]

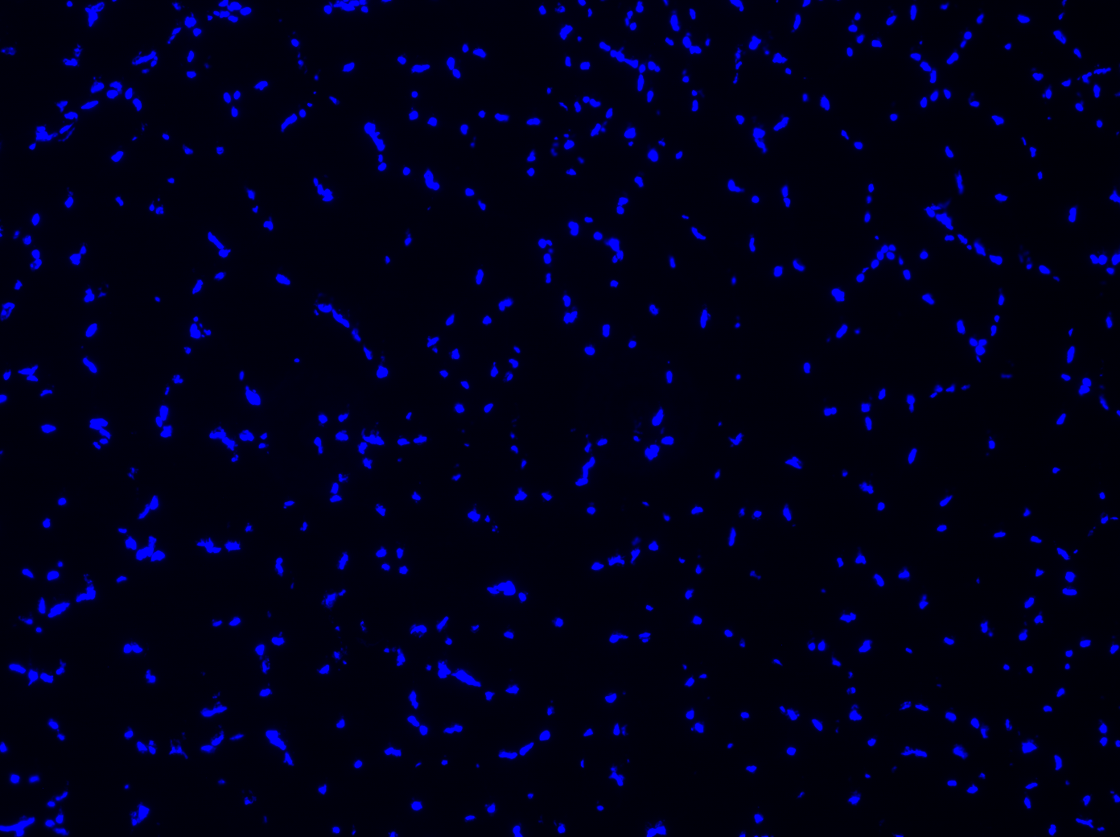

Supplement: Supplementary file 9 — Source Data for Figure 3 [file EMMM-14-e14526-s011.zip › Figure 3/Figure 3 Images/Figure 3J/treated_dapi.tif]

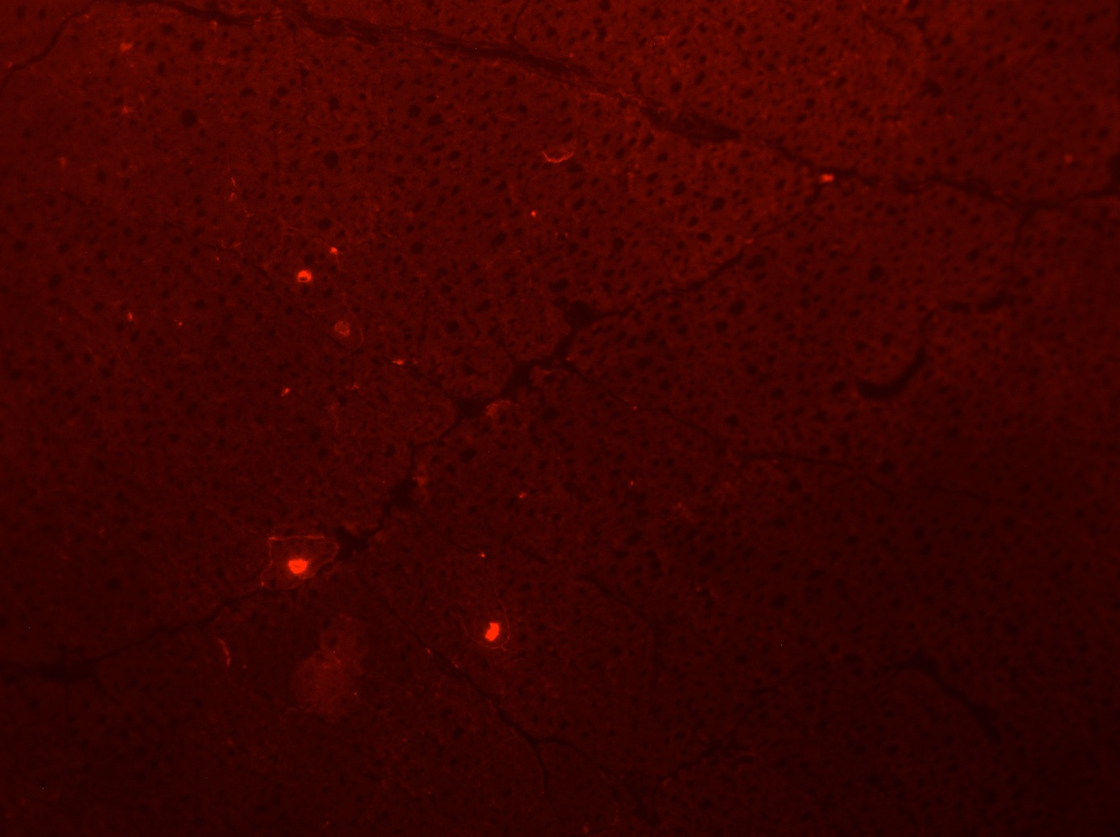

Supplement: Supplementary file 9 — Source Data for Figure 3 [file EMMM-14-e14526-s011.zip › Figure 3/Figure 3 Images/Figure 3J/untreated_laminac_spectrin.tif]

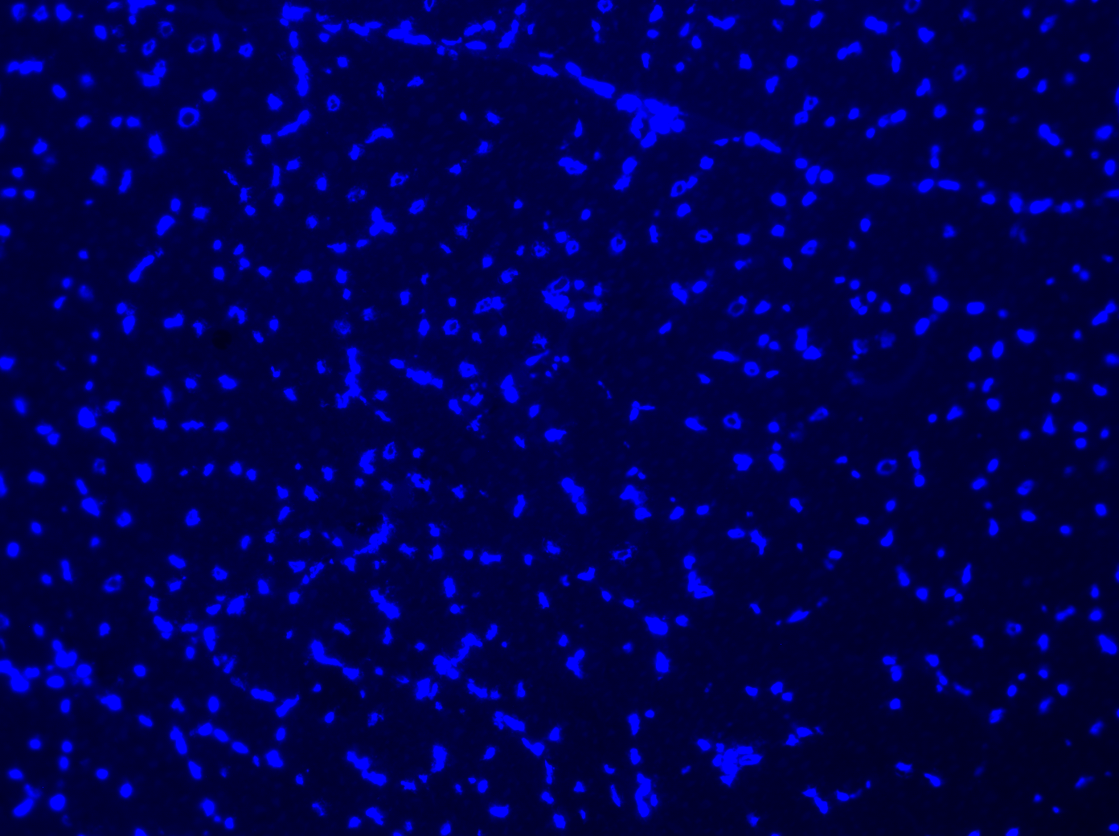

Supplement: Supplementary file 9 — Source Data for Figure 3 [file EMMM-14-e14526-s011.zip › Figure 3/Figure 3 Images/Figure 3J/untreated_dapi.tif]

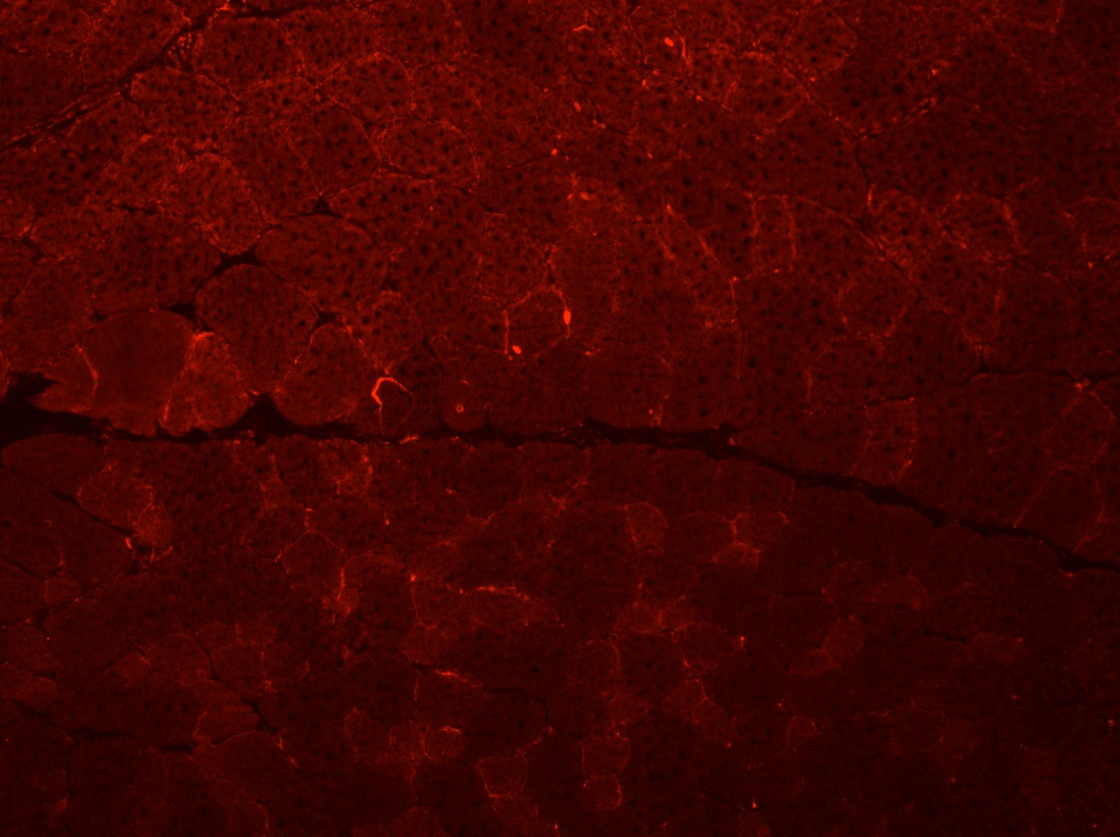

Supplement: Supplementary file 9 — Source Data for Figure 3 [file EMMM-14-e14526-s011.zip › Figure 3/Figure 3 Images/Figure 3J/treated_laminac_spectrin.tif]

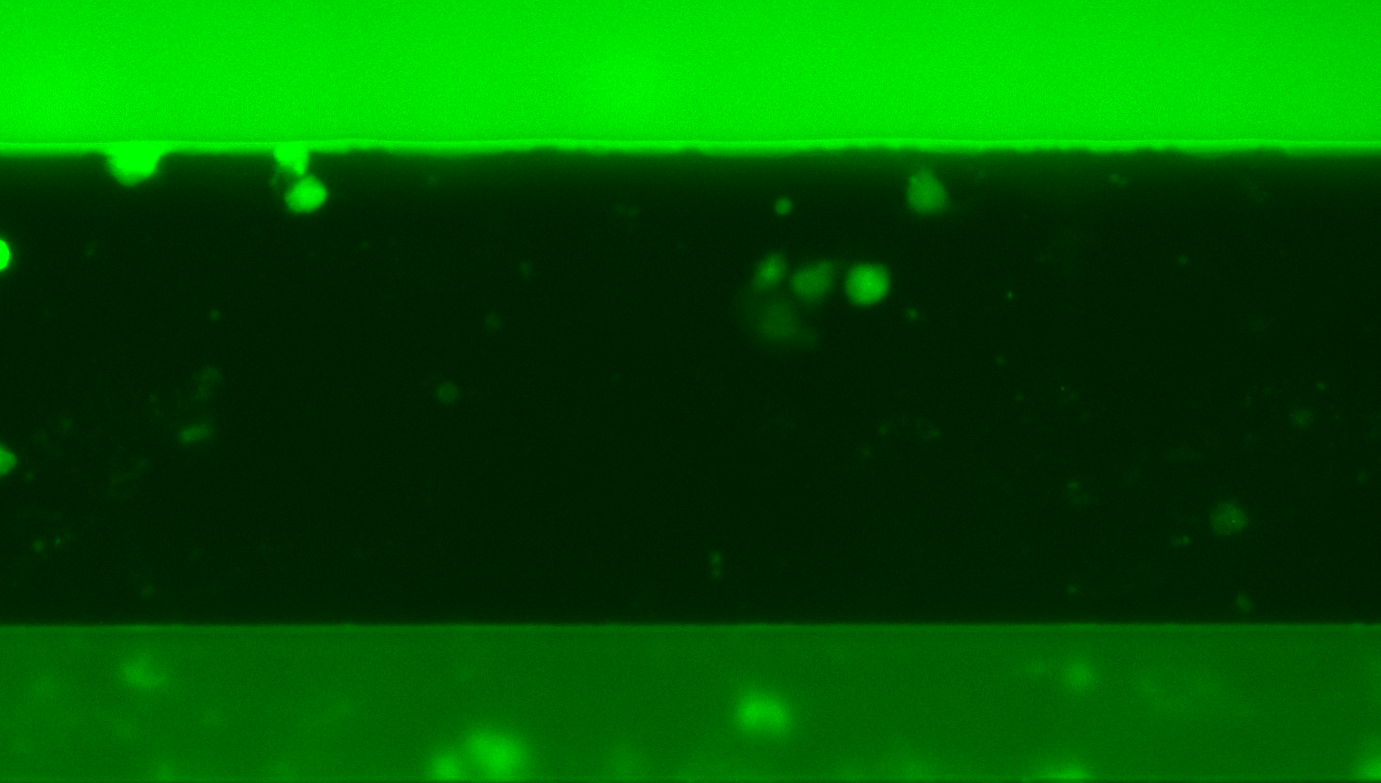

Supplement: Supplementary file 11 — Source Data for Figure 5 [file EMMM-14-e14526-s001.zip › Figure 5/Figure 5 Images/5F/treated_adhesion_upper.png]

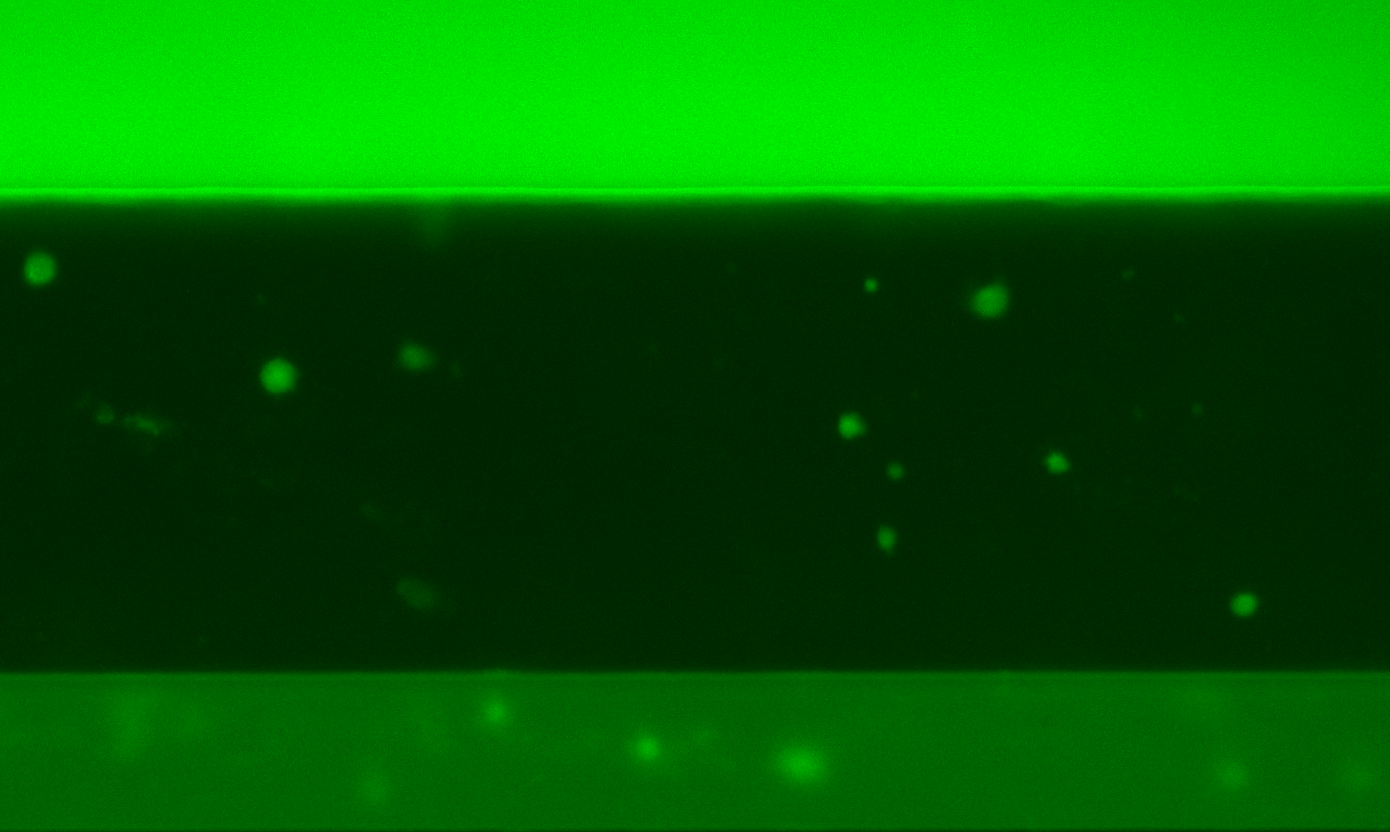

Supplement: Supplementary file 11 — Source Data for Figure 5 [file EMMM-14-e14526-s001.zip › Figure 5/Figure 5 Images/5F/untreated_adhesion_upper.png]

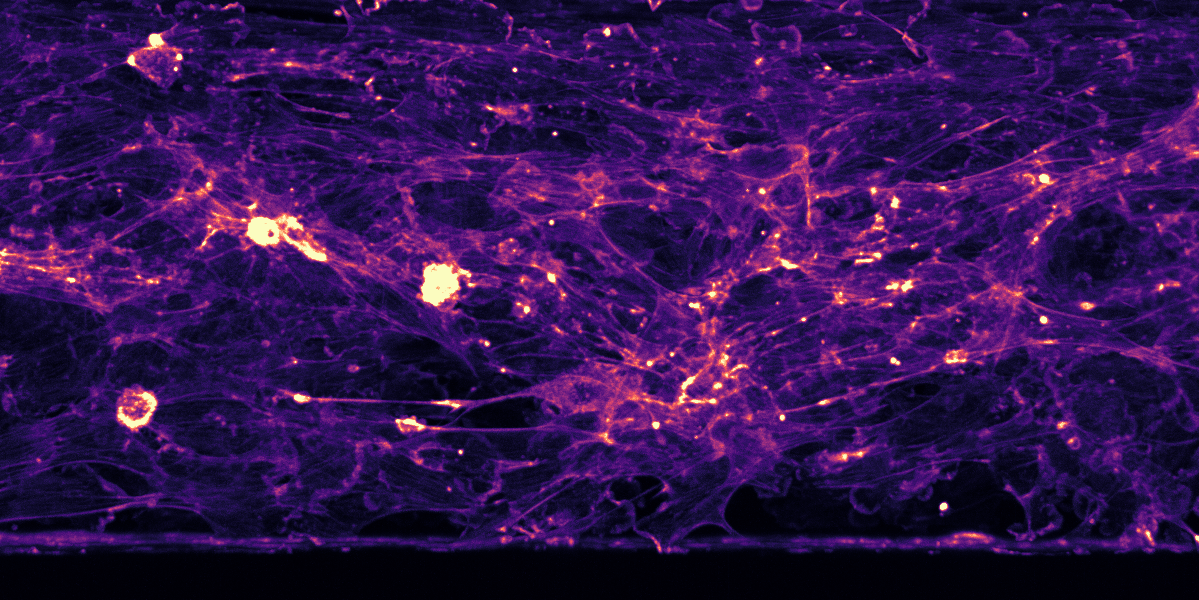

Supplement: Supplementary file 11 — Source Data for Figure 5 [file EMMM-14-e14526-s001.zip › Figure 5/Figure 5 Images/5C/series_3_phalloidin.png]

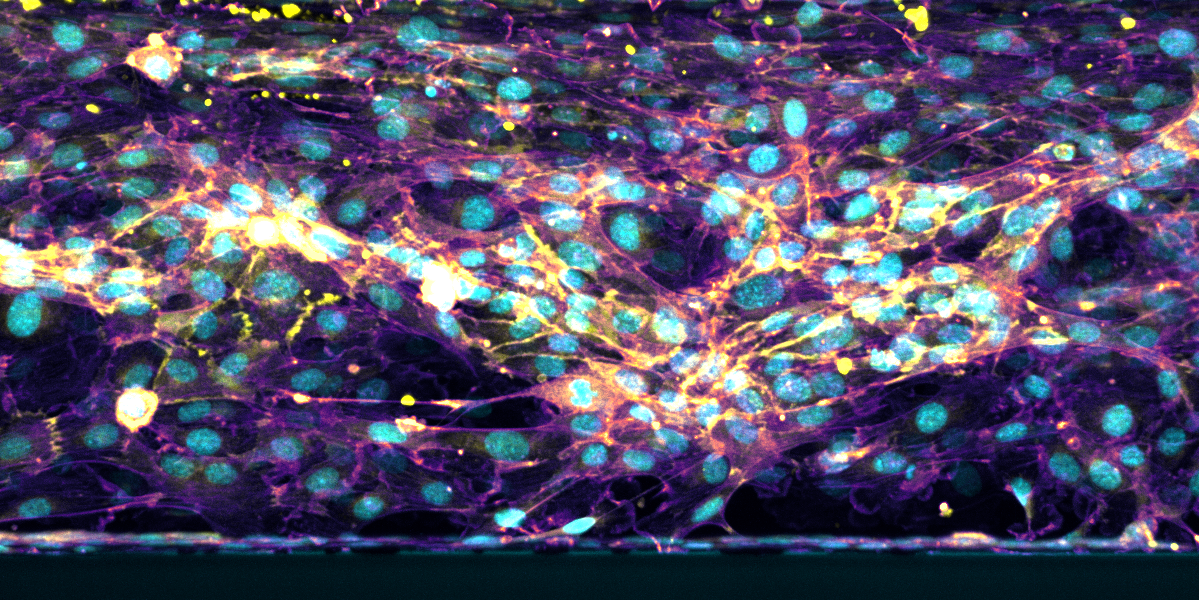

Supplement: Supplementary file 11 — Source Data for Figure 5 [file EMMM-14-e14526-s001.zip › Figure 5/Figure 5 Images/5C/series_3_merge.png]

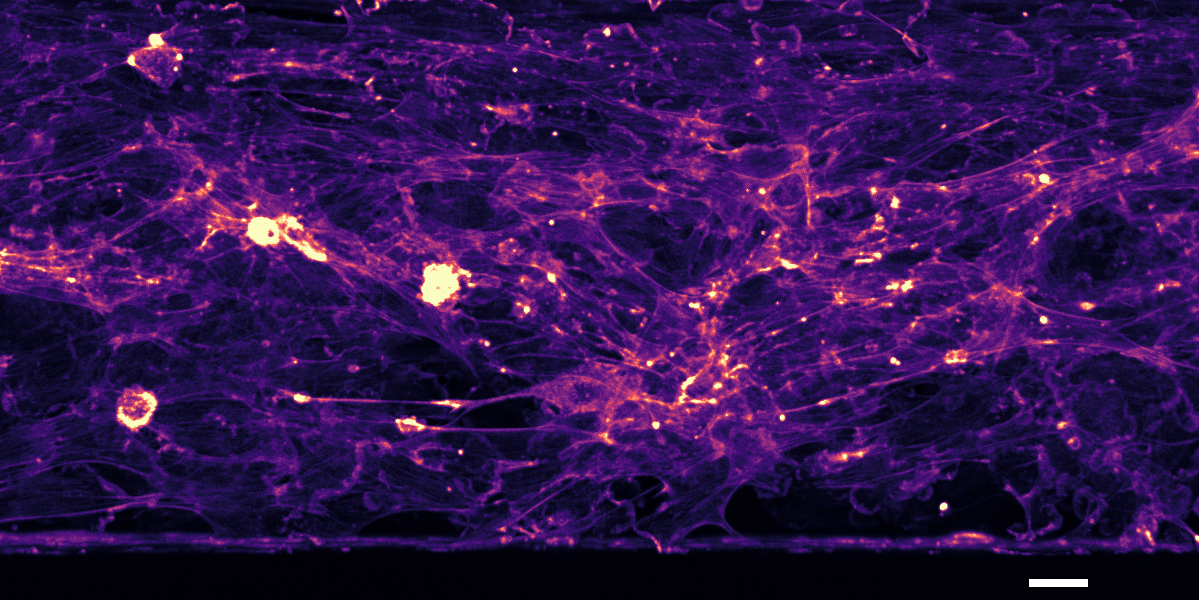

Supplement: Supplementary file 11 — Source Data for Figure 5 [file EMMM-14-e14526-s001.zip › Figure 5/Figure 5 Images/5C/series_3_phalloidin_scale_bar.png]

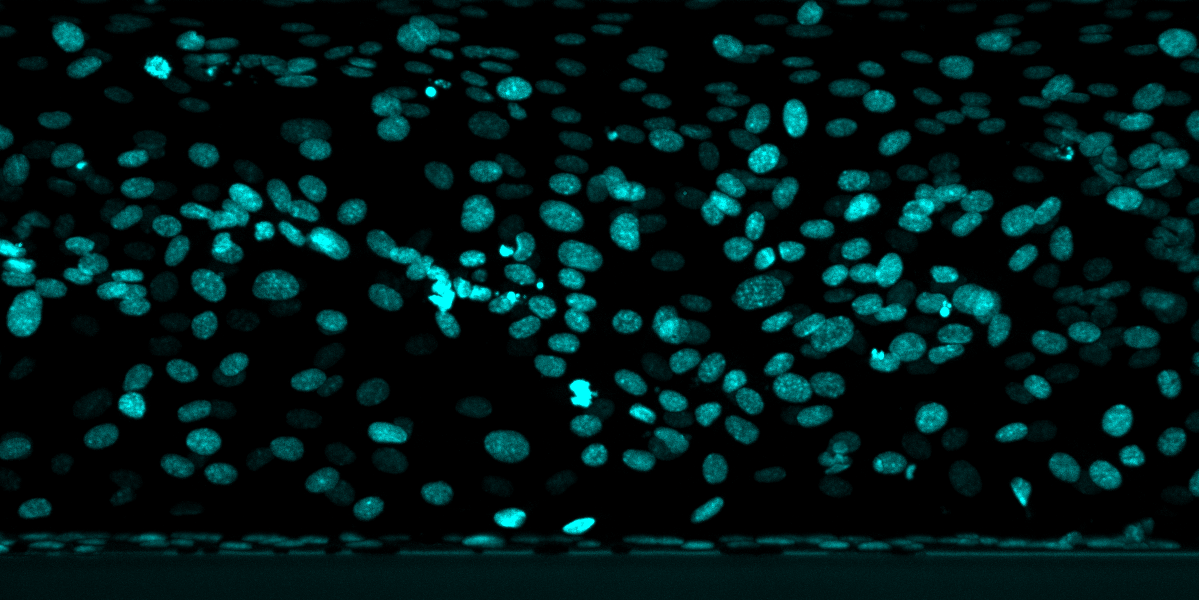

Supplement: Supplementary file 11 — Source Data for Figure 5 [file EMMM-14-e14526-s001.zip › Figure 5/Figure 5 Images/5C/series_3_DAPI.png]

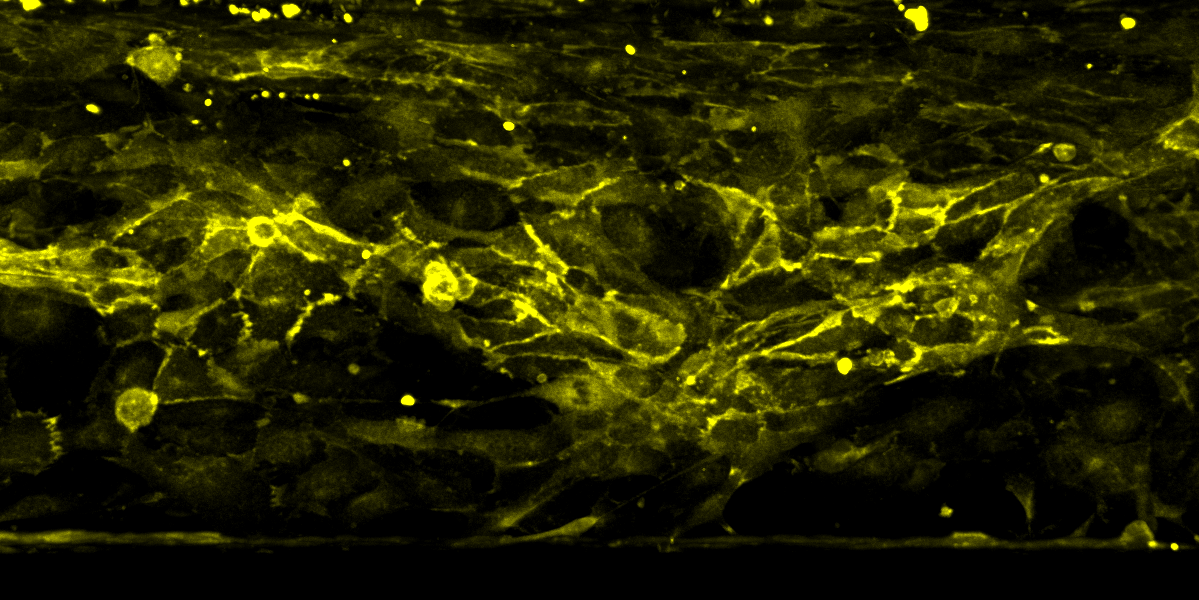

Supplement: Supplementary file 11 — Source Data for Figure 5 [file EMMM-14-e14526-s001.zip › Figure 5/Figure 5 Images/5C/series_3_CD31.png]

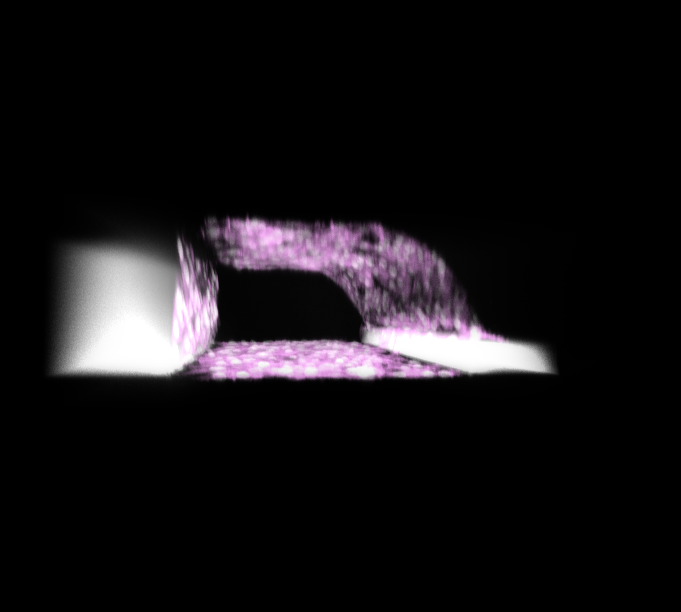

Supplement: Supplementary file 11 — Source Data for Figure 5 [file EMMM-14-e14526-s001.zip › Figure 5/Figure 5 Images/5D/huvec_tunnel.png]

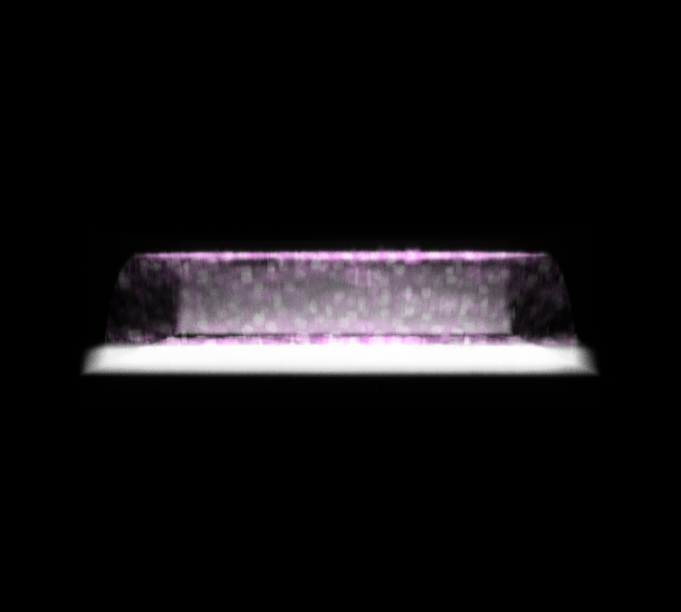

Supplement: Supplementary file 11 — Source Data for Figure 5 [file EMMM-14-e14526-s001.zip › Figure 5/Figure 5 Images/5D/huvec_side.png]

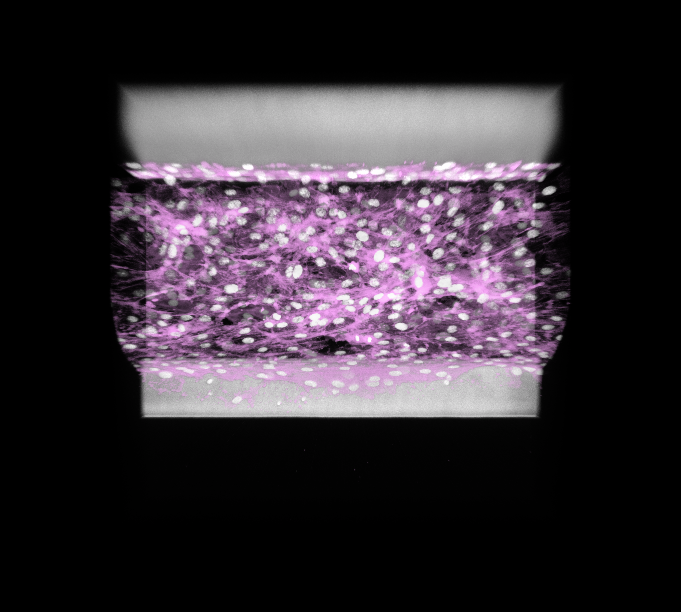

Supplement: Supplementary file 11 — Source Data for Figure 5 [file EMMM-14-e14526-s001.zip › Figure 5/Figure 5 Images/5D/huvec_top.png]

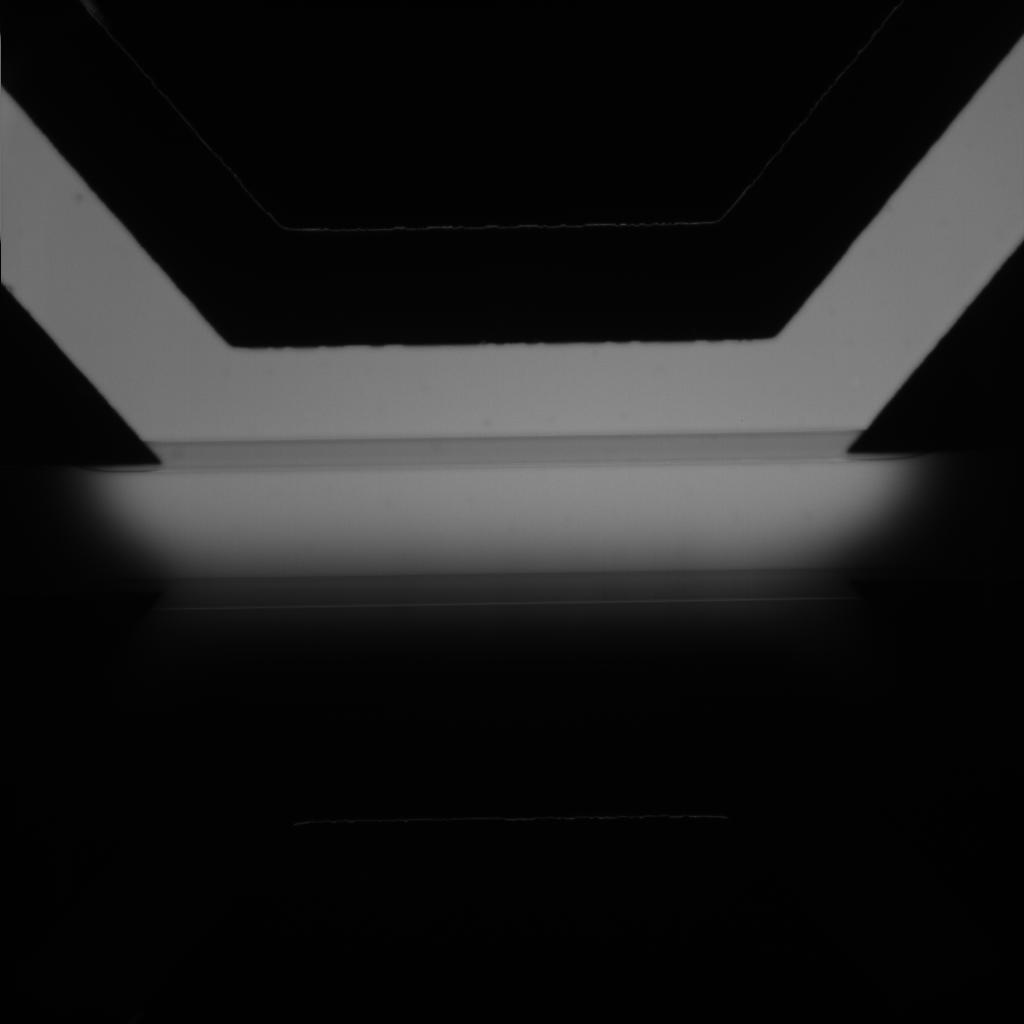

Supplement: Supplementary file 11 — Source Data for Figure 5 [file EMMM-14-e14526-s001.zip › Figure 5/Figure 5 Images/5E/cell-free/tritc/barrier_integrity_cf_tritc_1.tif]

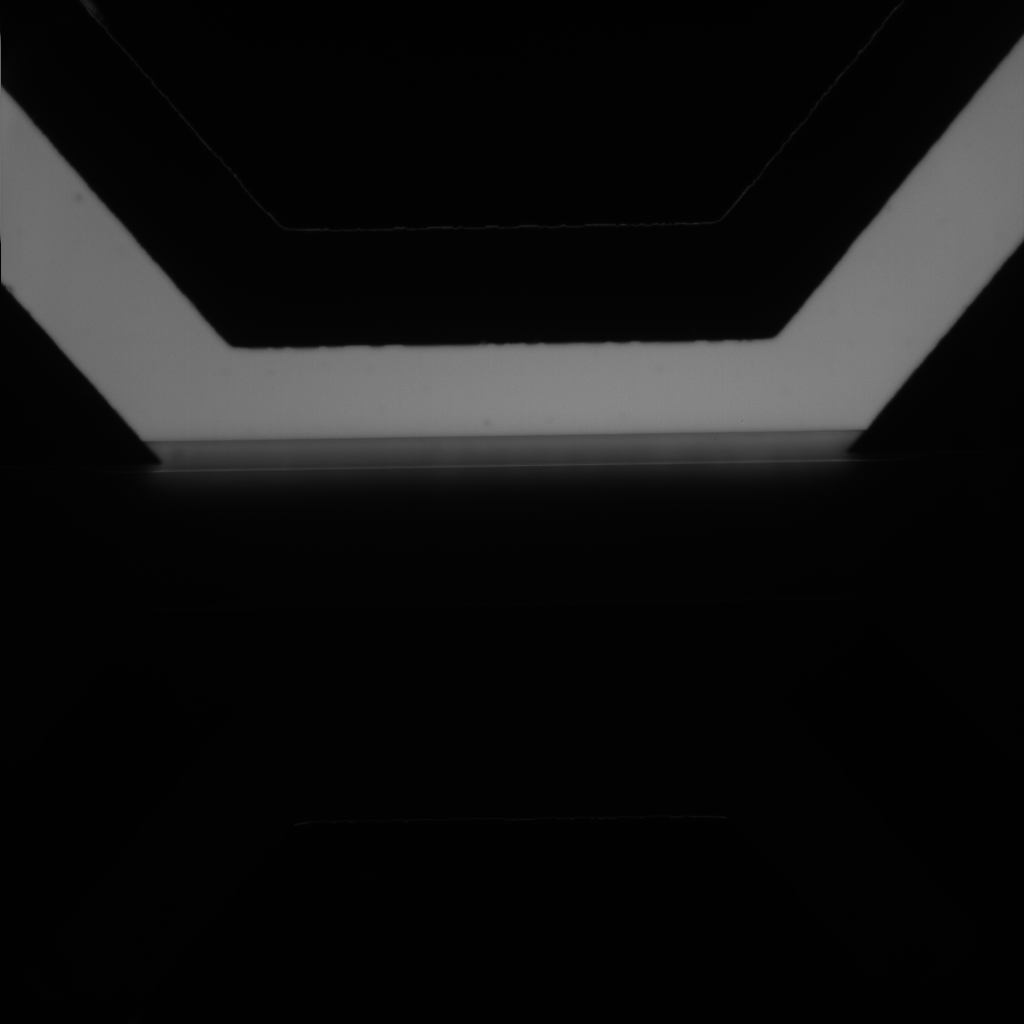

Supplement: Supplementary file 11 — Source Data for Figure 5 [file EMMM-14-e14526-s001.zip › Figure 5/Figure 5 Images/5E/cell-free/tritc/barrier_integrity_cf_tritc_0.tif]

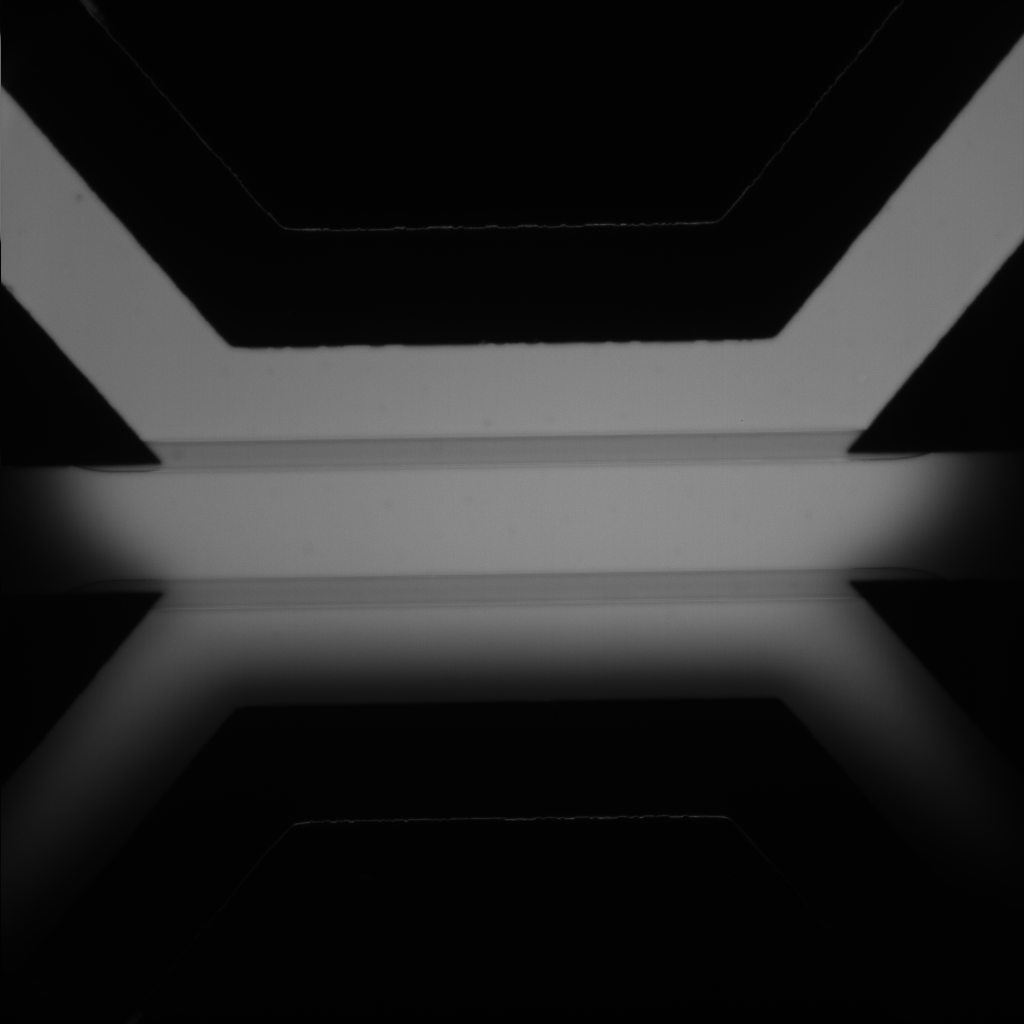

Supplement: Supplementary file 11 — Source Data for Figure 5 [file EMMM-14-e14526-s001.zip › Figure 5/Figure 5 Images/5E/cell-free/tritc/barrier_integrity_cf_tritc_2.tif]

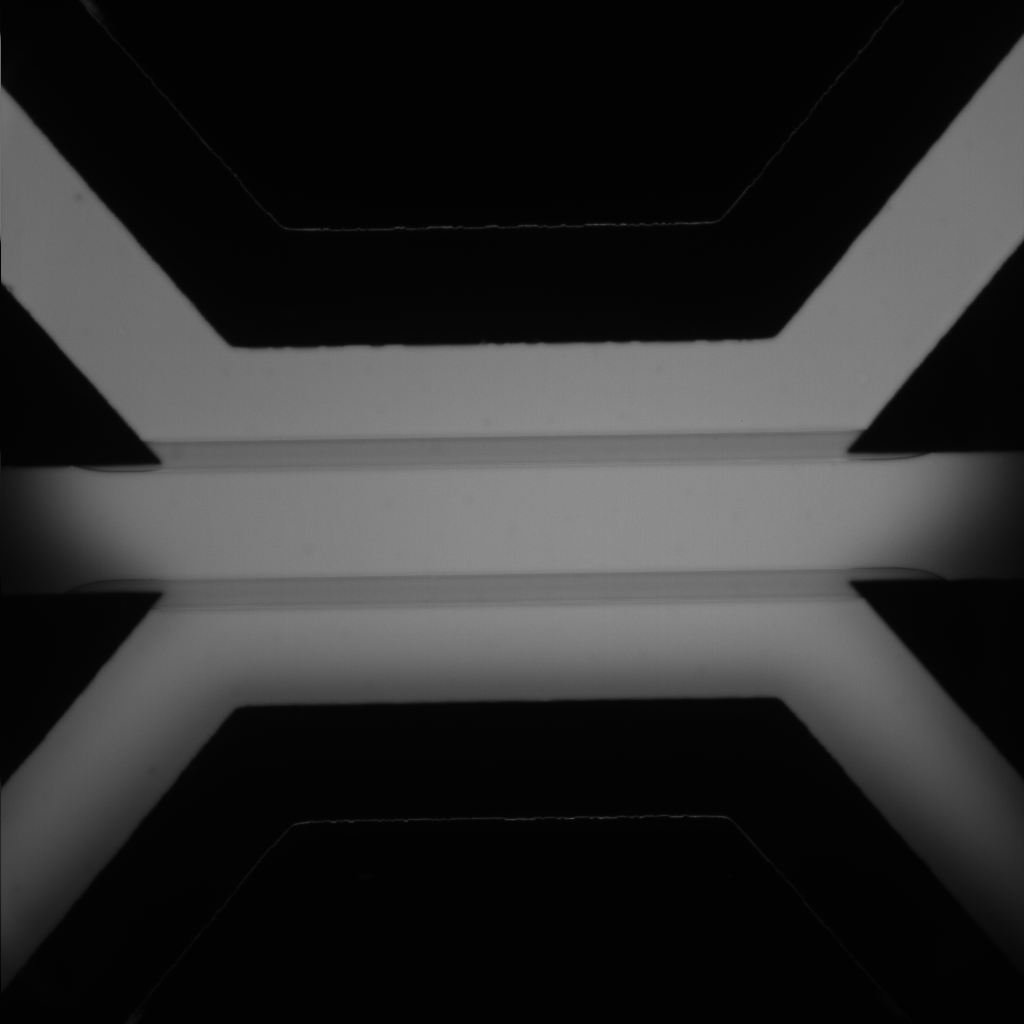

Supplement: Supplementary file 11 — Source Data for Figure 5 [file EMMM-14-e14526-s001.zip › Figure 5/Figure 5 Images/5E/cell-free/tritc/barrier_integrity_cf_tritc_3.tif]

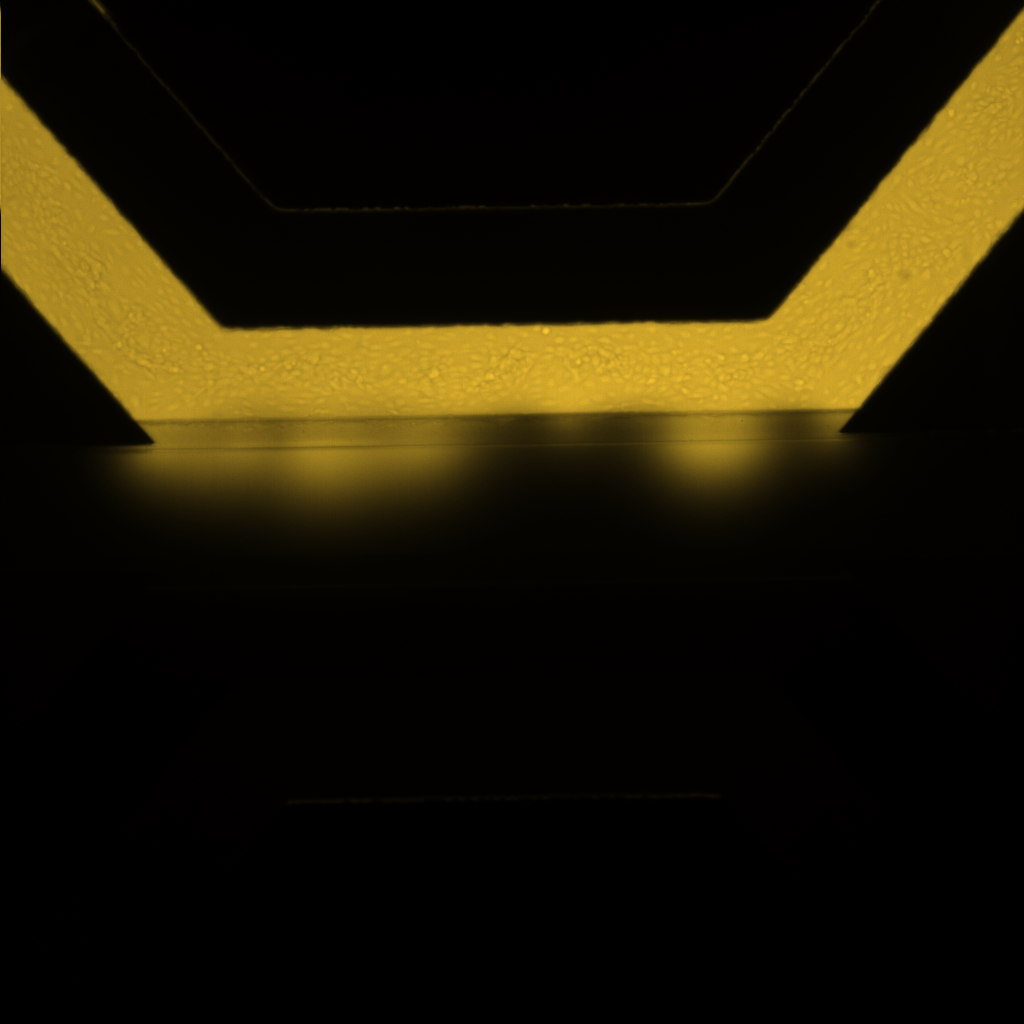

Supplement: Supplementary file 11 — Source Data for Figure 5 [file EMMM-14-e14526-s001.zip › Figure 5/Figure 5 Images/5E/huvecs/tritc/huvec_tritc_0001.png]

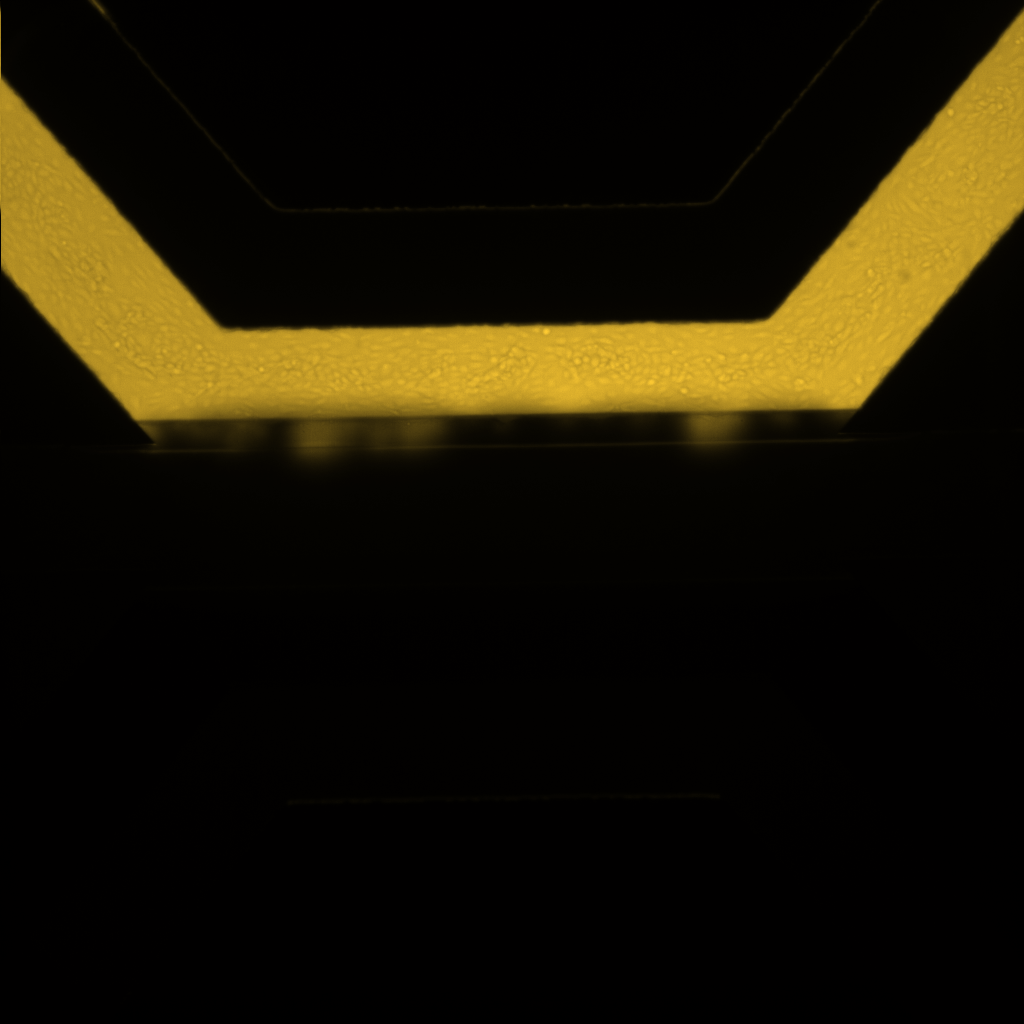

Supplement: Supplementary file 11 — Source Data for Figure 5 [file EMMM-14-e14526-s001.zip › Figure 5/Figure 5 Images/5E/huvecs/tritc/huvec_tritc_0000.png]

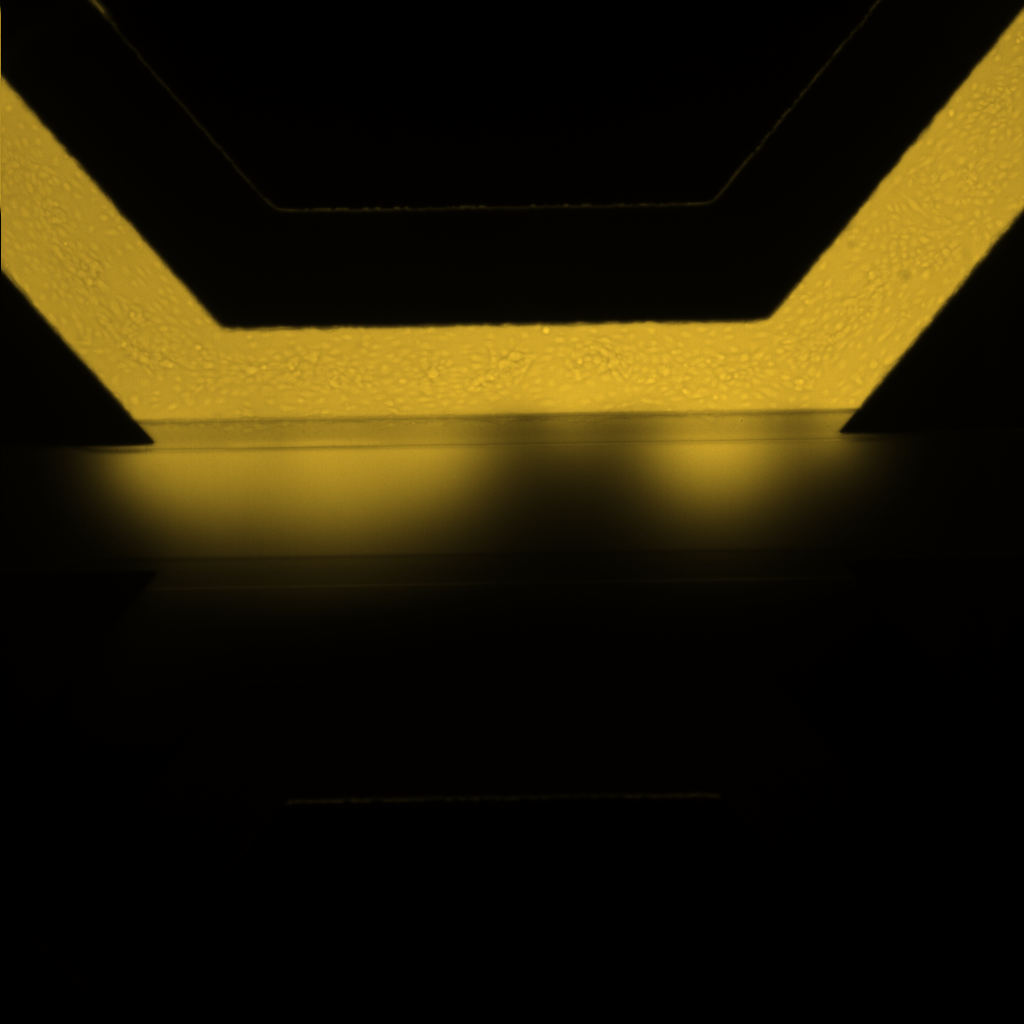

Supplement: Supplementary file 11 — Source Data for Figure 5 [file EMMM-14-e14526-s001.zip › Figure 5/Figure 5 Images/5E/huvecs/tritc/huvec_tritc_0002.png]

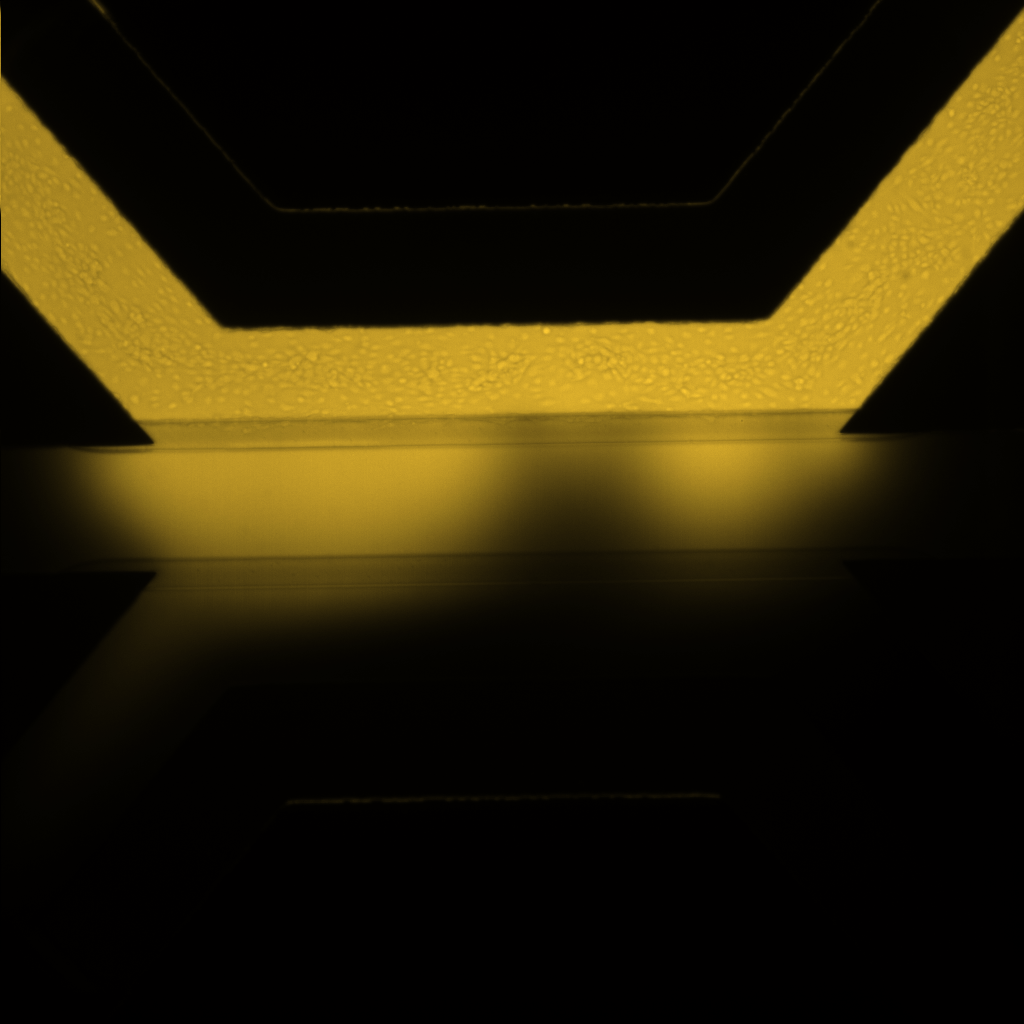

Supplement: Supplementary file 11 — Source Data for Figure 5 [file EMMM-14-e14526-s001.zip › Figure 5/Figure 5 Images/5E/huvecs/tritc/huvec_tritc_0003.png]

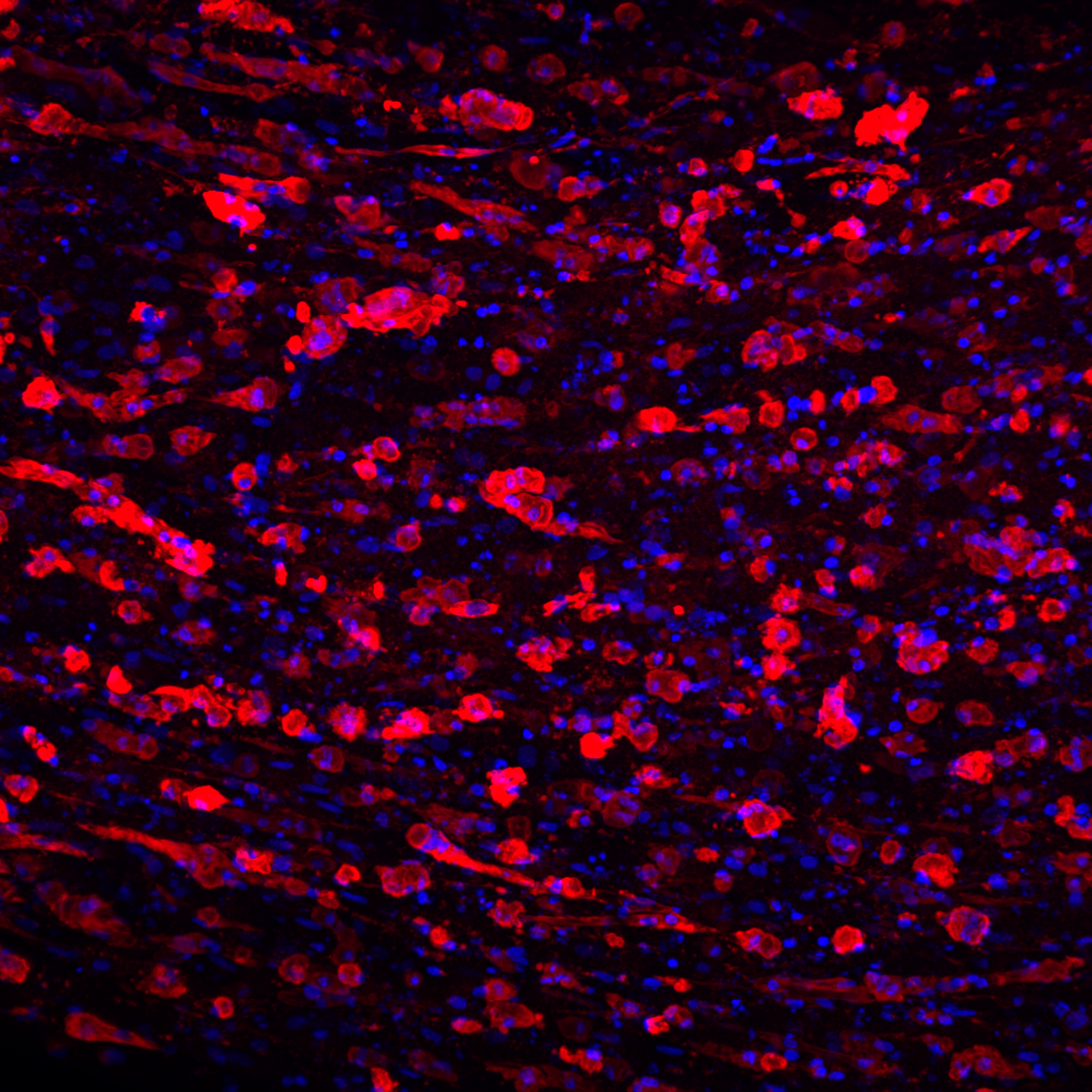

Supplement: Supplementary file 12 — Source Data for Figure 6 [file EMMM-14-e14526-s010.zip › Figure 6/Figure 6 Images/6B/MAX_3D_Injury_CTX100_2_002.png]

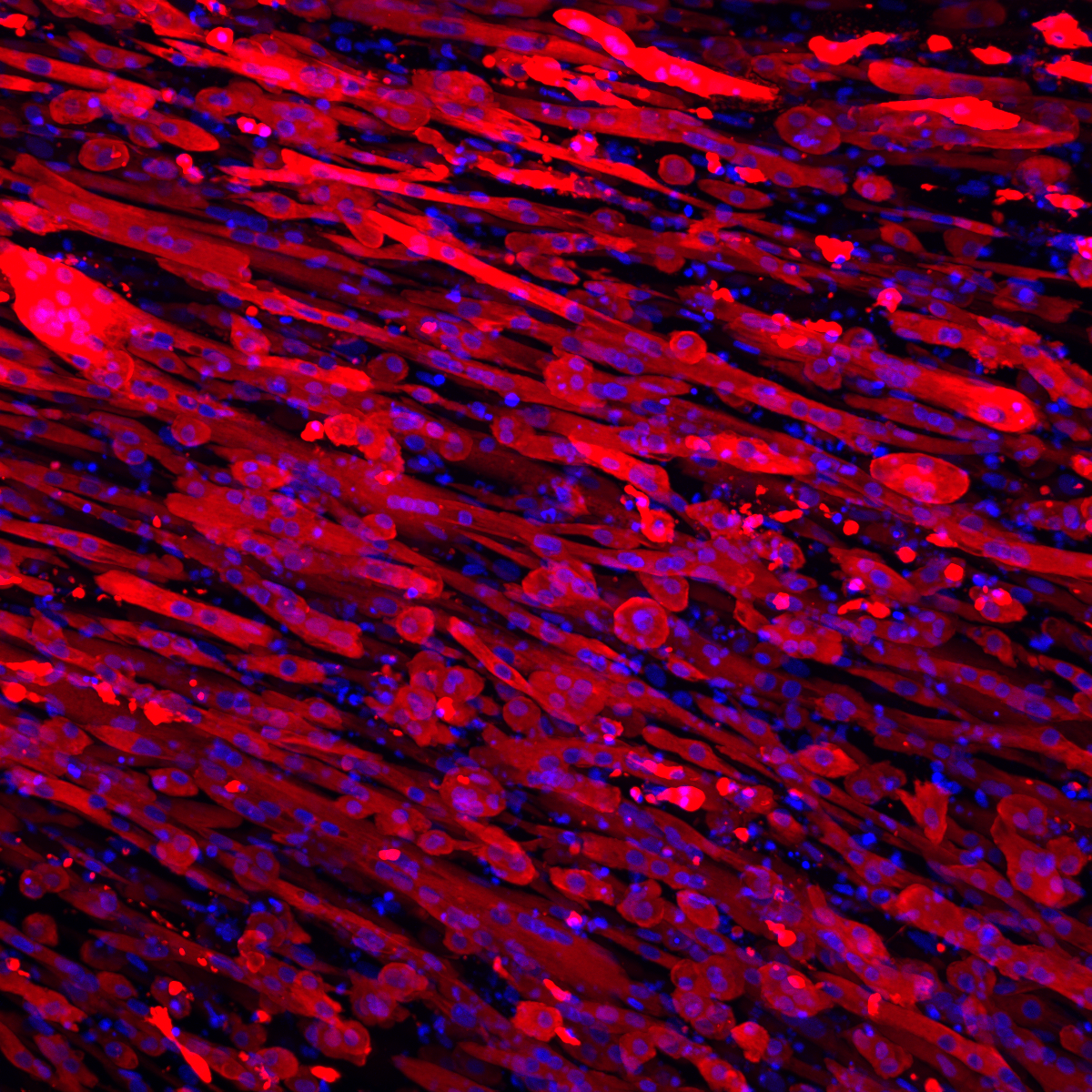

Supplement: Supplementary file 12 — Source Data for Figure 6 [file EMMM-14-e14526-s010.zip › Figure 6/Figure 6 Images/6B/MAX_3D_Injury_CTX10_2_001.png]

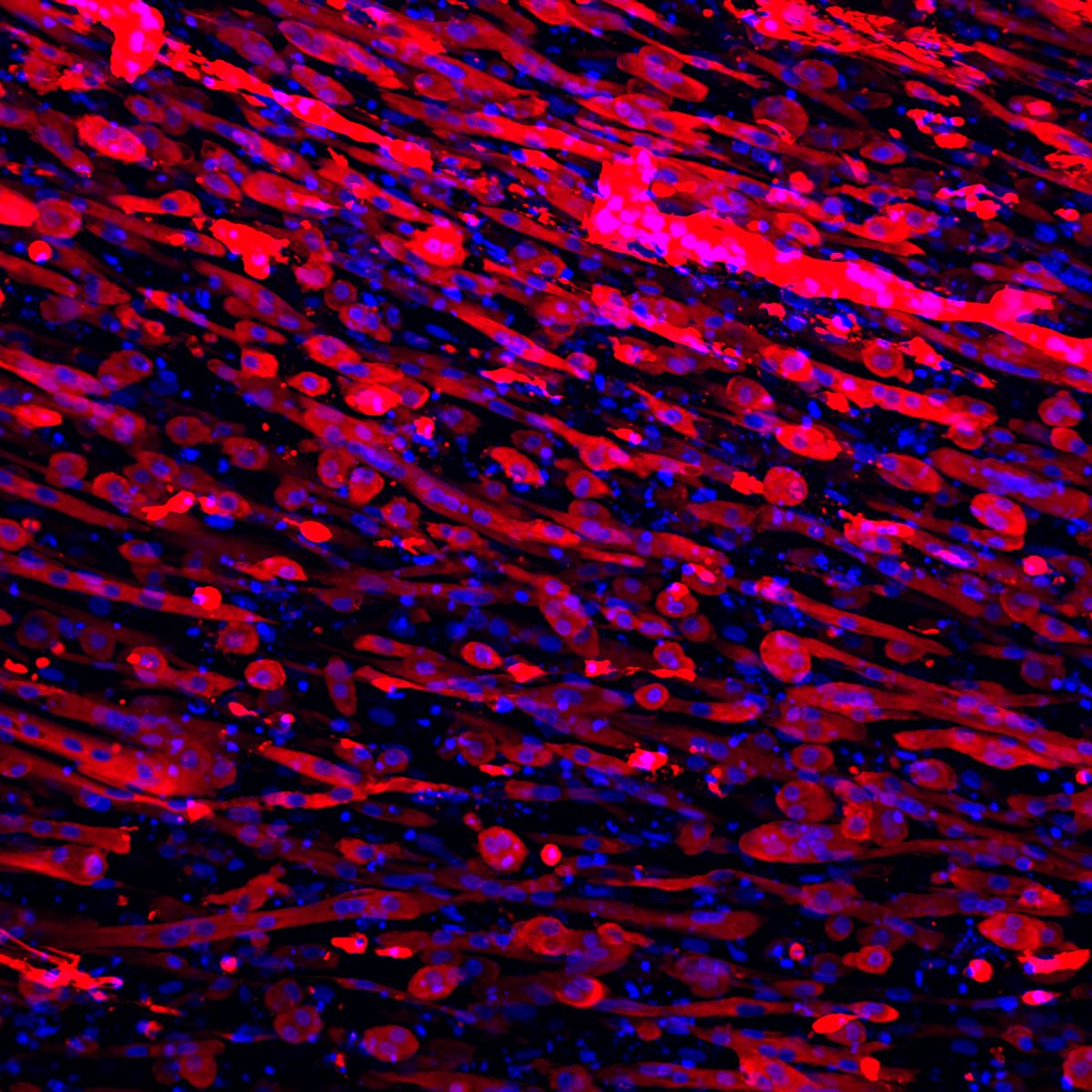

Supplement: Supplementary file 12 — Source Data for Figure 6 [file EMMM-14-e14526-s010.zip › Figure 6/Figure 6 Images/6B/MAX_3D_Injury_CTRL_.png]

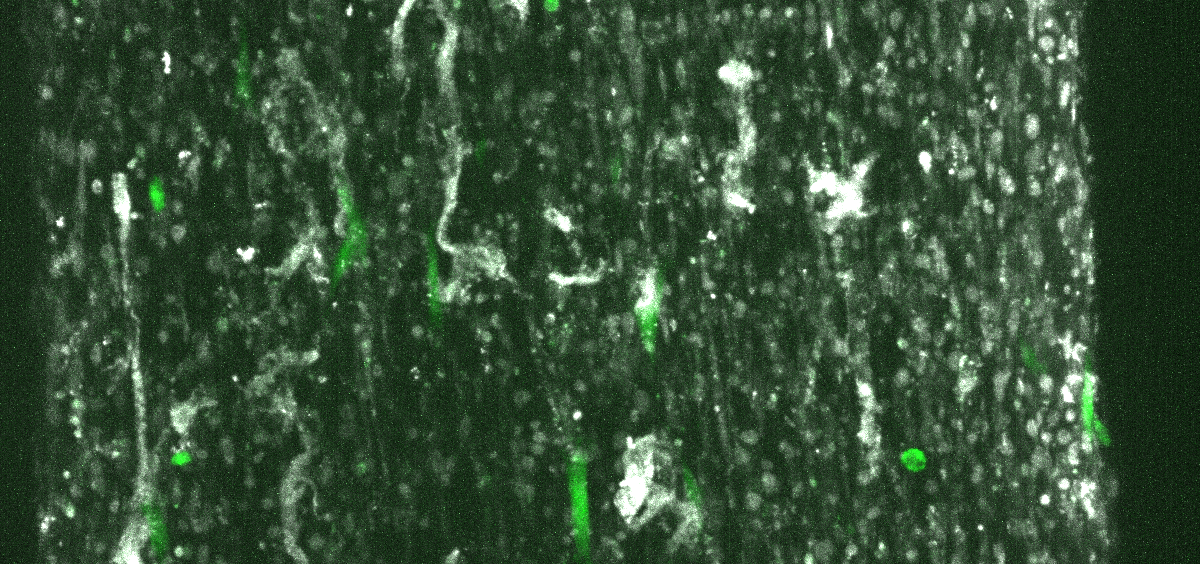

Supplement: Supplementary file 12 — Source Data for Figure 6 [file EMMM-14-e14526-s010.zip › Figure 6/Figure 6 Images/6C/sample_image_no_filter.png]

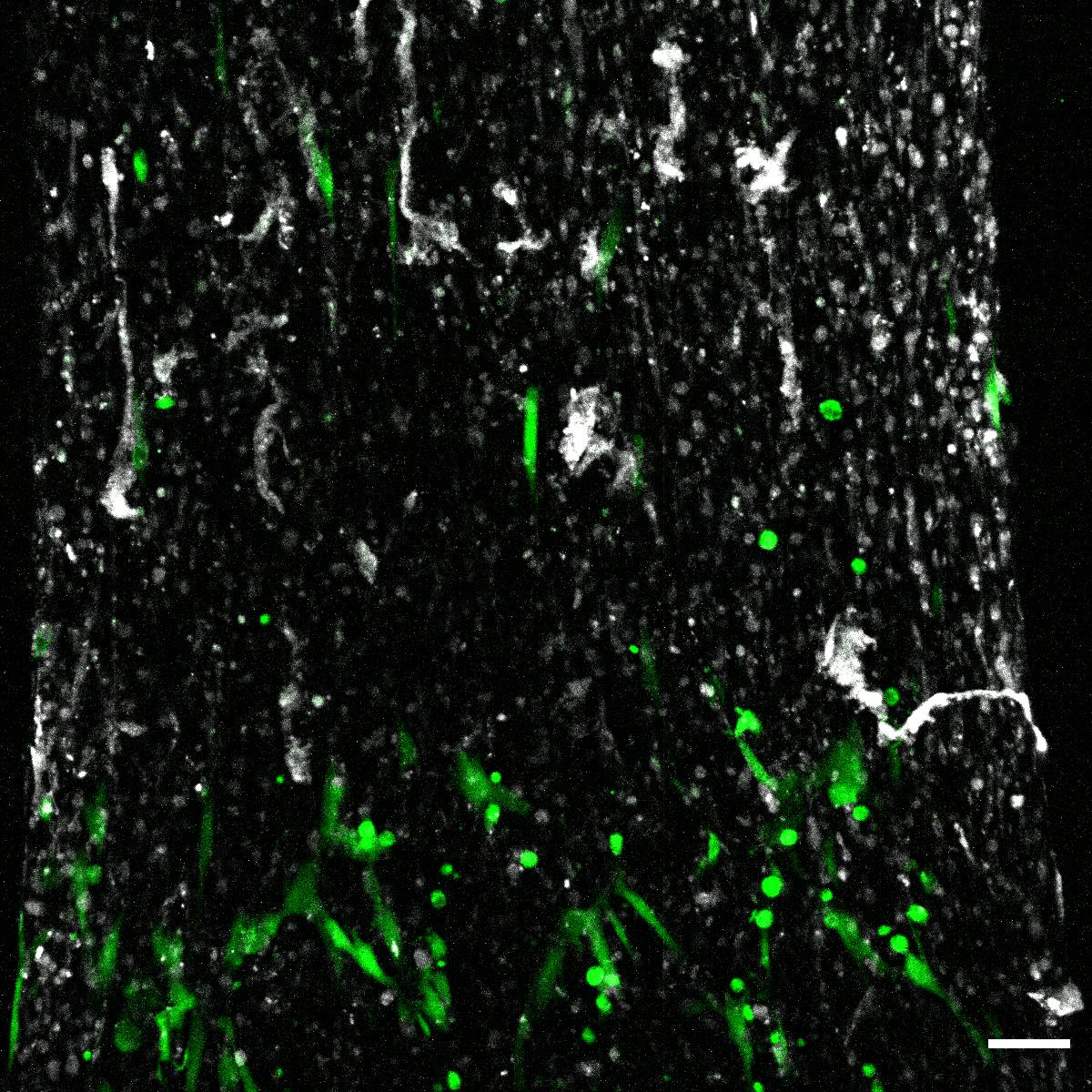

Supplement: Supplementary file 12 — Source Data for Figure 6 [file EMMM-14-e14526-s010.zip › Figure 6/Figure 6 Images/6C/sample_image_uncropped.tif]
