# Supplementary material for: Assessing and enhancing migration of human myogenic progenitors using directed iPS cell differentiation and advanced tissue modelling
Source: EMBO Mol Med. 2022 Sep 26;14(10):e14526. doi: 10.15252/emmm.202114526 (PMC9549733; doi:10.15252/emmm.202114526)
Supplement: Supplementary file 13 [file EMMM-14-e14526-s009.pdf]

EMBO Molecular Medicine

Assessing and enhancing migration of human myogenic progenitors using directed iPS cell differentiation and advanced tissue modelling

SungWoo Choi, Giulia Ferrari, Louise Moyle, Kirsty Mackinlay, Naira Naouar, Salma Jalal, Sara Benedetti, Christine Wells, Francesco Muntoni, and Francesco Saverio Tedesco
DOI: 10.15252/emmm.202114526

Corresponding author: Francesco Saverio Tedesco (f.s.tedesco@ucl.ac.uk)

Review Timeline:

Transfer from Review Commons:	8th May 21
Editorial Decision:	11th May 21
Revision Received:	1st Mar 22
Editorial Decision:	5th Apr 22
Revision Received:	19th Aug 22
Accepted:	19th Aug 22

Editor: Zeljko Durdevic

Transaction Report:

This manuscript was transferred to EMBO Molecular Medicine following peer review at Review Commons.

(Note: With the exception of the correction of typographical or spelling errors that could be a source of ambiguity, letters and reports are not edited. Depending on transfer agreements, referee reports obtained elsewhere may or may not be included in this compilation. Referee reports are anonymous unless the Referee chooses to sign their reports.)

Review #1

1. How much time do you estimate the authors will need to complete the suggested revisions:

Estimated time to Complete Revisions (Required)

(Decision Recommendation)

Between 1 and 3 months

2. Evidence, reproducibility and clarity:

Evidence, reproducibility and clarity (Required)

Summary:

The paper presented by Ferrari et al., aims to improve the migration capacity of hiPSC-derived myogenic progenitors. For this purpose, the authors used a previously published well characterized hiMPs model and focussed on the modulation of NOTCH and PDGF signaling pathways.
The rational to target these pathways was based on muscle cells migrations molecular events observed during developmental described in the literature.

Major comments:

- Are the key conclusions convincing?
This is a very interesting paper. Few clarifications as suggested below need to be done before being fully convincing.
Enrichment test and heat maps and the network analysis are not well explained in terms of which genes were selected and why, and in terms of which gene set were selected and why. In some cases, the information may be given in the paper, but it is not easy for the reader to find it. It should be stated more clearly. For example, in Fig2C why these eight were chosen for the heat maps and why not other genes known to be involved in myogenesis, cell migration etc. Similar comment for figure 3 A, D and G. Another example, in Fig 2E, on what basis are some gene sets chosen to be shown in this figure when there are many more significant in the supplementary table. Figure 4F is impossible to interpret without a clear description of how the subnetwork is extracted, was a list of gene list submitted to string, if so which genes and why? Secondly, why are there many nodes with no edges? Is it all of the nodes that are in that GO-Term, if so it needs to be clarified? Was this the most strongly deregulated go-Term according to string analysis?
Figure 4 B, C, D and E:

(1) The authors should clarify what figure 4B is? Is 1,2,3,4 different time point? Treated or untreated cells?

(2) Figure C: Is the graph showing the cell distribution of both treated and untreated cells? If

yes is it possible to give a different shape for the control cells and see if indeed more control green shape would be observed in this plot? (In the supplementary data there is the distribution showing the treated v untreated, but the clusters are not visible)

(3) Would it be possible to take some of the parameters in Figure 4D and show the distribution in treated vs untreated and perform the statistical analysis? (eg is there a significant difference for the parameter total distance between control and treated?). Or, may be just show some of the results in figure S4C and E in the main text.

(4) Why pooling the 3 independent experiment together? Looking at the data in Figure S4, it seems that one treated sample is very similar to the control, thus weakening the conclusion. The replicates in this figure are biological replicates. Yet the papers present 4/5 different cell lines, so why only 3 of them are used here? Is there some explanation regarding the outsider (cell line age, number of division etc). Might be worth adding data from the other cell lines (1 or 2 more).

(5) Figure 4 H and I: What are the statistic actually comparing: treated v untreated for each cell lines or different cell lines against each other? If the former, then how is it possible to have a 139 fold change with such a weak p value of 0.042? If the latter, then why is a p-value given for each of the 3 cell lines? Also, the number and source of replicates is unclear - N=3 is stated, so was each cell line done in triplicate? If so, how many fields per replicate?

- Would additional experiments be essential to support the claims of the paper? Request additional experiments only where necessary for the paper as it is, and do not ask authors to open new lines of experimentation.
It would important to also show the migratory capacity of these cells in vivo.
-Are the suggested experiments realistic in terms of time and resources? It would help if you could add an estimated cost and time investment for substantial experiments.
Human muscle cells engraftment and tracking in immunodeficient mice could be easily done. Engrafted muscle can be harvested 2-3 weeks after engraftment, and measurement of the distance from the engraftment point could be done (Site of injection could be labelled with tattoo die). This would be a month/month and half of work. Immunodeficient mice would cost around £1500 (n=6 mice per group => total of 12 mice) plus the cost of housing.
- Are the data and the methods presented in such a way that they can be reproduced? Are the experiments adequately replicated and statistical analysis adequate?

See comments in first paragraph. The authors should probably be able to answer easily to the different concerns raised above.

Minor comments:

typo "Onthology" should be "Ontology" in figure 2E.
Some of the data in Figure S4E should be moved to the main text.

3. Significance:

Significance (Required)

Describe the nature and significance of the advance, existing literature, audience: Generating iPSC cell lines with an improved capacity to migrate will be of high interest for the neuromuscular field, and could be a potential therapeutic strategy applicable for many neuromuscular disorders.

Muscle cell engraftment is quite challenging as the capacity of these cells to populate different muscles is very poor. Improving the cell migration, survival and proliferation may thus help to improve the muscle cell engraftment strategy.

Expertise:

I have an expertise in neuromuscular disorders, muscle stem cells (human and murine, in vitro and in vivo), as well as an expertise in omics analysis.

Review #2

1. How much time do you estimate the authors will need to complete the suggested revisions:

Estimated time to Complete Revisions (Required)

(Decision Recommendation)

Between 3 and 6 months

2. Evidence, reproducibility and clarity:

Evidence, reproducibility and clarity (Required)

In this manuscript, Ferrari and colleagues provide solid data indicating that the Notch ligand DLL4 and PDGF-BB regulate the migration of myogenic progenitors derived from human pluripotent stem cells (PSC). These studies built from recent work by the same group (Gerli et al, Stem Cell Reports, 12:461, 2019), in which the authors documented that Notch and PDGF-BB signaling enhances migration and expression of stem cell markers while inducing perivascular cell features in muscle satellite cells. Here the authors perform similar in vitro studies in PSC-derived myogenic progenitors and conclude that the same effect is observed in this population of cells. The results are clear and well presented.

Throughout the manuscript, the authors emphasize the importance of such findings for the future therapeutic application of a PSC-based therapy to treat patients with muscular dystrophy since multiples skeletal muscles need to be targeted in this group of diseases. Unfortunately, the authors do not provide transplantation data. The results would be highly meaningful if they show that observed in vitro changes (transcriptomes and chamber assay) result in meaningful migration in vivo using the systemic delivery, but as it is, the data do not support the claims and conclusions.

3. Significance:

Significance (Required)

Significance is limited if only in vitro data are provided. However if the authors are able to show enhanced engraftment upon systemic transplantation of human PSC-derived myogenic progenitors upon DLL4 and PDGF-BB treatment, the significance would be high.

In terms of existing literature, there are publications reporting systemic delivery of murine PSC-derived myogenic progenitors as well as transcriptome and in vitro migration studies. It would probably be appropriate to cite them.

If systemic engraftment is observed, the manuscript would be of interest to the skeletal muscle and stem cell biology/regenerative medicine community.

Review #3

1. How much time do you estimate the authors will need to complete the suggested revisions:

Estimated time to Complete Revisions (Required)

(Decision Recommendation)

Between 3 and 6 months

2. Evidence, reproducibility and clarity:

Evidence, reproducibility and clarity (Required)

In this manuscript, the authors exploited the signal-mediated activation of NOTCH and PDGF pathways, by one week-long delivery of DLL4 and PDGF-BB to cultures of hiPSC-derived myogenic progenitors in vitro, to improve their migration ability. They performed transcriptomic and functional analyses across human and mouse primary muscle stem cells and human hiPSC-derived myoblasts, including genetically corrected hiPSC derivatives, to show that DLL4 and PDGF-BB treatment modulates pathways involved in cell migration, including enhanced trans-endothelial migration in transwell assays.
The increased migratory ability, and in particular enhancing extravasation, is a fundamental property required for optimal performance of hiPSC myogenic derivatives, upon their intravascular delivery; hence, the finding reported here are of extremely high potential interest in term of solution of one of the major bottle-neck of cell therapy.
However, there are important issues that need to be resolved by the authors with additional experimentation, that I recommend performimg, in order to improve this manuscript.

1) The most critical issue here is that the authors fail to provide evidence that DLL4/PDGF-

BB-treated cultures of hiPSC-derived myogenic progenitors do not lose their myogenic potential and are able to form myotubes, upon interruption of treatment. It would be also important to determine when (how many days after withdrawal of DLL4/PDGF-BB) the full myogenic properties of these cells are recovered. From the RNAseq datasets shown by the authors, it appears that DLL4/PDGF-BB-treated hiPSC-derived myoblasts do not express the key genes of myogenic identity (MyoD) and early differentiation (myogenin), while expressing genes of mesenchymal/vessel-derived lineages. It is imperative that the authors show that these changes are reversible, upon withdrawal of DLL4/PDGF-BB. This should be show by an unbiased transcriptomic analysis (RNAseq) of hiPSC-derived myoblasts after withdrawal of DLL4/PDGF-BB, that should be integrated with functional evidence showing that these cells can resume their ability to form differentiated myotubes, upon exposure to myogenic culture cues in vitro.

2) A parallel evidence in vivo should be also provided, showing that DLL4/PDGF-BB-treated hiPSC-derived myoblasts do not express MyoD and myogenin when delivered intravascularly, but regain their expression after they have crossed the vessel endothelium and have entered the skeletal muscles.
If these experiments could firmly demonstrate that DLL4/PDGF-BB-treatment reversibly promotes migratory properties of hiPSC-derived myoblasts (as predicted, but not demonstrated in previous works from the same group, using mouse or human primary muscle stem cells - Cappellari et al. 2013; Gerli et al. 2019), then this work could be a great interest in term of basic and translational biology and clearly suitable for publication in a top journal.

Other points:

- Fig. 2A. it looks like there are some outlier RNAseq sample replicates that might negatively impact at the statistical level on the subsequent analysis. This issue is likely due to the heterogeneity of the samples (both untreated and treated) and could be resolved by replacing outlier samples with new replicates.
- Along the same line as above, sample heterogeneity following treatment might be resolved by a better understanding of optimal doses of DLL4/PDGF-BB and time of exposure, which I recommend the authors to define by additional experiments.

3. Significance:

Significance (Required)

If these experiments could firmly demonstrate that DLL4/PDGF-BB-treatment reversibly promotes migratory properties of hiPSC-derived myoblasts (as predicted, but not demonstrated in previous works from the same group, using mouse or human primary muscle stem cells - Cappellari et al. 2013; Gerli et al. 2019), then this work could be a great interest in term of basic and translational biology and clearly suitable for publication in a top journal and could be interesting for a wide audience in regenerative medicine.

Expertise of this reviewer:

Muscle regeneration; Muscular Dystrophies; Signaling and Epigenetics

Review Commons Reviews for Refereed Preprint RC-2021-00693

Ferrari G. *et al*., DLL4 and PDGF-BB regulate migration of human iPSC-derived skeletal myogenic progenitors.

Response to Reviewers in blue font.

Reviewer #1 (Evidence, reproducibility and clarity (Required)):

Summary:

The paper presented by Ferrari et al., aims to improve the migration capacity of hiPSC-derived myogenic progenitors. For this purpose, the authors used a previously published well characterized hiMPs model and focussed on the modulation of NOTCH and PDGF signaling pathways.
The rational to target these pathways was based on muscle cells migrations molecular events observed during developmental described in the literature.

Major comments:

Are the key conclusions convincing?

This is a very interesting paper. Few clarifications as suggested below need to be done before being fully convincing.

Enrichment test and heat maps and the network analysis are not well explained in terms of which genes were selected and why, and in terms of which gene set were selected and why. In some cases, the information may be given in the paper, but it is not easy for the reader to find it. It should be stated more clearly. For example, in Fig2C why these eight were chosen for the heat maps and why not other genes known to be involved in myogenesis, cell migration etc. Similar comment for figure 3 A, D and G. Another example, in Fig 2E, on what basis are some gene sets chosen to be shown in this figure when there are many more significant in the supplementary table.

We thank the Reviewer for their positive feedback and for this comment. Although some answers to the queries could be found within the figure legends, we agree that figures could have been more self-explanatory, and we will amend them accordingly. We will also add additional information into the main text to clarify those specific points.
In response to the specific queries:

- All enrichment heat maps were generated from GO lists or KEGG pathways.
- 2C: these were chosen instead of other myogenic or cell migration markers for consistency with our previous study (Figure 2C in Gerli et al Stem Cell Reports 2019).
- 3A, D, G: details of the GO lists used to generate heat maps were available in the relative figure legend.
- 2E: enrichment pathways – we listed pathways shared between at least 2 of the three groups and with relevance to cellular migration.

Figure 4F is impossible to interpret without a clear description of how the subnetwork is extracted, was a list of gene list submitted to string, if so which genes and why? Secondly, why are there many nodes with no edges? Is it all of the nodes that are in that GO-Term, if so it needs to be clarified? Was this the most strongly deregulated go-Term according to string analysis?

We thank the Reviewer for this comment. This specific GO list was selected for its highly relevant title/topic, i.e.: "positive regulation of cell migration". Details on this point could also be found in the specific figure legend, where we specified how the network is extracted and

constructed. There are several nodes with no edges as the edges represent predicted functional association and therefore, a lack of edges suggests a lack of interaction.

Figure 4 B, C, D and E:
(1) The authors should clarify what figure 4B is? Is 1,2,3,4 different time point? Treated or untreated cells?

We apologise with the Reviewer for not having provided enough information on this point. 1,2,3 and 4 are four sequential time points of untreated cells. We will amend the figure to make this clearer.

(2) Figure C: Is the graph showing the cell distribution of both treated and untreated cells? If yes is it possible to give a different shape for the control cells and see if indeed more control green shape would be observed in this plot? (In the supplementary data there is the distribution showing the treated v untreated, but the clusters are not visible)

We thank the Reviewer for this helpful comment. We agree that this will increase the quality of the figure. We will distinguish treated and control cells within figure 4C by replacing dots with different shapes for treated and untreated samples.

(3) Would it be possible to take some of the parameters in Figure 4D and show the distribution in treated vs untreated and perform the statistical analysis? (eg is there a significant difference for the parameter total distance between control and treated?). Or, may be just show some of the results in figure S4C and E in the main text.

We thank the Reviewer for this comment. We agree that it will be better to move S4C into the main figure and we will action this point in the revised version of the manuscript.

(4) Why pooling the 3 independent experiment together? Looking at the data in Figure S4, it seems that one treated sample is very similar to the control, thus weakening the conclusion. The replicates in this figure are biological replicates. Yet the papers present 4/5 different cell lines, so why only 3 of them are used here? Is there some explanation regarding the outsider (cell line age, number of division etc). Might be worth adding data from the other cell lines (1 or 2 more).

We thank the Reviewer for this point. The experiment shown in figure S4E has been performed with one cell line (N5) and independent experimental replicates were assessed for the statistical analysis. We are not sure why there appears to be an outlier in some cases, and this is why it was important to replicate this experiment three times. However, we will also repeat this experiment with another cell line applying more stringent conditions to strengthen this point.

(5) Figure 4 H and I: What are the statistic actually comparing: treated v untreated for each cell lines or different cell lines against each other? If the former, then how is it possible to have a 139 fold change with such a weak p value of 0.042? If the latter, then why is a p-value given for each of the 3 cell lines? Also, the number and source of replicates is unclear - N=3 is stated, so was each cell line done in triplicate? If so, how many fields per replicate?

We are happy to clarify this point for the Reviewer. The statistical analysis compares treated vs. untreated samples within the same genotype. The high fold change observed is likely

due to the large standard deviation of the dataset, which was also highlighted as raw data in the figure panel (bottom part of each picture in white colour font). For this reason, we have repeated this experiment multiple times and validated it across three independent cell lines.

Would additional experiments be essential to support the claims of the paper? Request additional experiments only where necessary for the paper as it is, and do not ask authors to open new lines of experimentation.
It would important to also show the migratory capacity of these cells in vivo.
-Are the suggested experiments realistic in terms of time and resources? It would help if you could add an estimated cost and time investment for substantial experiments.
Human muscle cells engraftment and tracking in immunodeficient mice could be easily done. Engrafted muscle can be harvested 2-3 weeks after engraftment, and measurement of the distance from the engraftment point could be done (Site of injection could be labelled with tattoo die). This would be a month/month and half of work. Immunodeficient mice would cost around £1500 (n=6 mice per group => total of 12 mice) plus the cost of housing.

We thank the Reviewer for this comment. We agree that the suggested in vivo experiment might strengthen our work and we are currently sourcing all required materials to perform it. Additionally, we will perform a similar, quasi-vivo, experiment to study migration in a species-specific setting by delivering cells in 3D models in vitro (e.g. Maffioletti SM et al., Cell Reports 2018). This strategy will provide a solid alternative to the in vivo assay, in the eventuality that the xenogeneic setting will limit the resolution of the proposed transplantation experiment.

Are the data and the methods presented in such a way that they can be reproduced? Are the experiments adequately replicated and statistical analysis adequate?

See comments in first paragraph. The authors should probably be able to answer easily to the different concerns raised above.

Minor comments:

typo "Onthology" should be "Ontology" in figure 2E.
Some of the data in Figure S4E should be moved to the main text.

Thanks for highlighting these minor comments. We will correct the typo and move data from figure S4 into the main figure 4.

Reviewer #2 (Evidence, reproducibility and clarity (Required)):

In this manuscript, Ferrari and colleagues provide solid data indicating that the Notch ligand DLL4 and PDGF-BB regulate the migration of myogenic progenitors derived from human pluripotent stem cells (PSC). These studies built from recent work by the same group (Gerli et al, Stem Cell Reports, 12:461, 2019), in which the authors documented that Notch and PDGF-BB signaling enhances migration and expression of stem cell markers while inducing perivascular cell features in muscle satellite cells. Here the authors perform similar in vitro studies in PSC-derived myogenic progenitors and conclude that the same effect is observed in this population of cells. The results are clear and well presented.

Throughout the manuscript, the authors emphasize the importance of such findings for the future therapeutic application of a PSC-based therapy to treat patients with muscular dystrophy since multiples skeletal muscles need to be targeted in this group of diseases. Unfortunately, the authors do not provide transplantation data. The results would be highly meaningful if they show that observed in vitro changes (transcriptomes and chamber assay) result in meaningful migration in vivo using the systemic delivery, but as it is, the data do not support the claims and conclusions.

We thank Reviewer 2 for their comments. We were pleased to read that they found our study and data solid, clear and well presented. Although we agree with the Reviewer that in vivo evidence would strengthen our findings, we would like to highlight that our study did not aim to be a translational investigation of the therapeutic potential of treated hPSC derivatives for muscle cell therapy (we believe our manuscript's title reflects this). We see this work more as a foundational study to establish the required evidence for future, follow up transplantation studies focused on the therapeutic potential of this approach (something requiring a dedicated project, funding and months/years of work).

Moreover, we believe that xenogeneic transplants are of limited use to investigate a complex species-specific phenomenon such as transendothelial cell migration. For this very reason we moved back to intraspecific transplants in past studies (e.g.. Tedesco et al Sci Transl Med 2012). However, as a key aim of our study is to obtain data specific to human cells and given that we already performed mouse-in-mouse *in vivo* intra-arterial delivery experiments using DLL4 and PDGFBB treated primary cells in Gerli *et al.* Stem Cell Reports 2018, we are therefore proposing and planning to:

1) Test transendothelial migration with another *quasi-vivo* microfluidic assay orthogonal to the reported transwell experiments. This will model intraspecific (i.e., human-in-human) transendothelial migration under flow conditions.
2) Assess evidence of migration in human 3D muscles setting up a novel invasion assay in our in vitro 3D muscle models.
3) Perform intramuscular delivery of treated vs. untreated cells as per Reviewer 1 request to assess migration in skeletal muscle *in vivo*.

This approach will optimise in vivo experiments in a 3Rs compliant fashion, avoiding invasive procedures in animals to study intravascular delivery.

Reviewer #2 (Significance (Required)):

Significance is limited if only in vitro data are provided. However if the authors are able to show enhanced engraftment upon systemic transplantation of human PSC-derived myogenic progenitors upon DLL4 and PDGF-BB treatment, the significance would be high.

Please see our reply to the previous point.

In terms of existing literature, there are publications reporting systemic delivery of murine PSC-derived myogenic progenitors as well as transcriptome and in vitro migration studies. It would probably be appropriate to cite them.

We apologies to the Reviewer for this oversight. We will add the following papers which include systemic delivery of murine PSC-derived myogenic progenitors as well as transcriptome and migration studies: Matthias N *et al.*, Exp Cell Res 2015; Incitti T *et al.*, PNAS 2019.

If systemic engraftment is observed, the manuscript would be of interest to the skeletal muscle and stem cell biology/regenerative medicine community.
Please see our reply to the initial point.

Reviewer #3 (Evidence, reproducibility and clarity (Required)):

In this manuscript, the authors exploited the signal-mediated activation of NOTCH and PDGF pathways, by one week-long delivery of DLL4 and PDGF-BB to cultures of hiPSC-derived myogenic progenitors in vitro, to improve their migration ability. They performed transcriptomic and functional analyses across human and mouse primary muscle stem cells and human hiPSC-derived myoblasts, including genetically corrected hiPSC derivatives, to show that DLL4 and PDGF-BB treatment modulates pathways involved in cell migration, including enhanced trans-endothelial migration in transwell assays.
The increased migratory ability, and in particular enhancing extravasation, is a fundamental property required for optimal performance of hiPSC myogenic derivatives, upon their intravascular delivery; hence, the finding reported here are of extremely high potential interest in term of solution of one of the major bottle-neck of cell therapy.
However, there are important issues that need to be resolved by the authors with additional experimentation, that I recommend performing, in order to improve this manuscript.

We sincerely thank the Reviewer for acknowledging the extremely high relevance and potential of our paper for muscle gene and cell therapies and for providing constructive feedback to improve our manuscript.

1) The most critical issue here is that the authors fail to provide evidence that DLL4/PDGF-BB-treated cultures of hiPSC-derived myogenic progenitors do not lose their myogenic potential and are able to form myotubes, upon interruption of treatment. It would be also important to determine when (how many days after withdrawal of DLL4/PDGF-BB) the full myogenic properties of these cells are recovered. From the RNAseq datasets shown by the authors, it appears that DLL4/PDGF-BB-treated hiPSC-derived myoblasts do not express the key genes of myogenic identity (MyoD) and early differentiation (myogenin), while expressing genes of mesenchymal/vessel-derived lineages. It is imperative that the authors show that these changes are reversible, upon withdrawal of DLL4/PDGF-BB. This should be show by an unbiased transcriptomic analysis (RNAseq) of hiPSC-derived myoblasts after withdrawal of DLL4/PDGF-BB, that should be integrated with functional evidence showing that these cells can resume their ability to form differentiated myotubes, upon exposure to myogenic culture cues in vitro.

We thank the Reviewer for this comment. We agree that this is an important and feasible experiment which will add important information to our work. We performed similar work in our previous study and already observed phenotype reversion of treated cells upon release of the stimuli within a few passages in cultures. However, we agree that this requires systematic assessment and quantification. To this aim, we will assess the reversibility of the DLL4 & PDGF-BB effect by stopping treatment at day 7 and then assessing skeletal myogenic differentiation capacity of target cells at sequential passages and time points post-treatment. Analysis of the differentiation index at different time points will provide functional evidence on the myogenic potential of hiPSC-derived myogenic progenitors post-withdrawal of DLL4 & PDGF-BB. We believe that the Reviewer's suggestion for transcriptomic analysis via RNA-seq might be overly costly for the purpose of identifying the myogenic potential of

treated cells post-withdrawal of treatment, and that qPCR panels alongside immunofluorescence staining may be sufficient.

2) A parallel evidence in vivo should be also provided, showing that DLL4/PDGF-BB-treated hiPSC-derived myoblasts do not express MyoD and myogenin when delivered intravascularly, but regain their expression after they have crossed the vessel endothelium and have entered the skeletal muscles.

We thank the Reviewer for suggesting this experiment. We agree that this would be a very interesting point to address; however, it might be very challenging to address this question with the proposed *in vivo* experiment. Nonetheless, we believe that with a combination of *in vitro* and *in vivo* assays we will be able to satisfactorily answer the question: Do DLL4 and PDGF-BB-treated myogenic progenitors re-gain myogenic potential upon entering skeletal muscle tissue? To this aim, we aim to analyse muscles following intramuscular transplantation of treated and untreated cells. Moreover, to model intra-vascular delivery and have high resolution imaging, we aim to adapt a microfluidic platform to perform trans-endothelial assays and selectively differentiate cells that successfully cross the blood vessel layer. Although likely to be very challenging, we will attempt to capture or stain those very cells in order to assess the expression of myogenic markers as requested by the Reviewer.

If these experiments could firmly demonstrate that DLL4/PDGF-BB-treatment reversibly promotes migratory properties of hiPSC-derived myoblasts (as predicted, but not demonstrated in previous works from the same group, using mouse or human primary muscle stem cells - Cappellari et al. 2013; Gerli et al. 2019), then this work could be a great interest in term of basic and translational biology and clearly suitable for publication in a top journal.

We thank the Reviewer for this constructive feedback and for seeing the great potential of our work in terms of basic and translational biology. We assume there was a typo in the sentence in brackets with a missing "as" ("..not demonstrated as in previous work..."): we indeed demonstrated the effect of DLL4 and PDGFBB *in vivo* extensively in our previous work.

Other points:

- Fig. 2A. it looks like there are some outlier RNAseq sample replicates that might negatively impact at the statistical level on the subsequent analysis. This issue is likely due to the heterogeneity of the samples (both untreated and treated) and could be resolved by replacing outlier samples with new replicates.

We thank the Reviewer for this comment. Although we agree that replacing those samples with new replicates might improve our statistical analyses, this will be financially challenging at this stage and perhaps also not completely reflecting the real variability of the experimental setup.

- Along the same line as above, sample heterogeneity following treatment might be resolved by a better understanding of optimal doses of DLL4/PDGF-BB and time of exposure, which I recommend the authors to define by additional experiments.

We thank the Reviewer for this comment. This is a potentially interesting experiment, which we have not performed as we took advantage of previous knowledge and dose-response on primary mouse and human myoblasts. Overall, we believe that this experiment might not be

strictly required at this stage, given that we have already solid evidence of response in hiMPs with a defined concentration and exposure time of DLL4 and PDGFBB.

Reviewer #3 (Significance (Required)):

If these experiments could firmly demonstrate that DLL4/PDGF-BB-treatment reversibly promotes migratory properties of hiPSC-derived myoblasts (as predicted, but not demonstrated in previous works from the same group, using mouse or human primary muscle stem cells - Cappellari et al. 2013; Gerli et al. 2019), then this work could be a great interest in term of basic and translational biology and clearly suitable for publication in a top journal and could be interesting for a wide audience in regenerative medicine.

We thank the Reviewer once again for this constructive feedback and for seeing the great potential of our work in terms of basic and translational biology, as well as for regenerative medicine.

Expertise of this reviewer:
Muscle regeneration; Muscular Dystrophies; Signaling and Epigenetics

Sincerely,

Prof. Francesco Saverio Tedesco
University College London and The Francis Crick Institute,
London, UK

11th May 2021

Dear Prof. Tedesco,

Thank you for the submission of your manuscript to EMBO Molecular Medicine. I have now had a chance to carefully read your point-by-point response. I also discussed your work and your response to the referees' comments with other members of our editorial team. All three reviewers are generally supportive of your study; however, they raise important critique regarding the lack of in vivo evidence to support the main conclusions of the study. We find your plan to address these points satisfactory. I would like to stress at this point that providing in vivo data on migratory capacity and myogenic potential of treated cell is essential for further considering the manuscript in EMBO Molecular Medicine.

Further consideration of a revision that addresses reviewers' concerns in full will entail a second round of review. EMBO Molecular Medicine encourages a single round of revision only and therefore, acceptance or rejection of the manuscript will depend on the completeness of your responses included in the next, final version of the manuscript. For this reason, and to save you from any frustrations in the end, I would strongly advise against returning an incomplete revision.

We would welcome the submission of a revised version within six months for further consideration. However, we realize that the current situation is exceptional on the account of the COVID-19/SARS-CoV-2 pandemic. Please let us know if you require longer to complete the revision.

I look forward to receiving your revised manuscript.

Yours sincerely,

Zeljko Durdevic

Zeljko Durdevic
Editor
EMBO Molecular Medicine

Response to Reviewers for EMM-2021-14526 | [RC-2021-00693] [REV]
Choi & Ferrari *et al*., Modelling and enhancing migration of human myogenic progenitors using iPS cells, microfluidics and bioengineered muscles (previously: Ferrari et al., DLL4 and PDGF-BB regulate migration of human iPSC-derived skeletal myogenic progenitors.

Reviewer #1 (Evidence, reproducibility and clarity (Required)):

Summary:
The paper presented by Ferrari et al., aims to improve the migration capacity of hiPSC-derived myogenic progenitors. For this purpose, the authors used a previously published well characterized hiMPs model and focussed on the modulation of NOTCH and PDGF signaling pathways.
The rational to target these pathways was based on muscle cells migrations molecular events observed during developmental described in the literature.

Major comments:

Are the key conclusions convincing?
This is a very interesting paper. Few clarifications as suggested below need to be done before being fully convincing.

Enrichment test and heat maps and the network analysis are not well explained in terms of which genes were selected and why, and in terms of which gene set were selected and why. In some cases, the information may be given in the paper, but it is not easy for the reader to find it. It should be stated more clearly. For example, in Fig2C why these eight were chosen for the heat maps and why not other genes known to be involved in myogenesis, cell migration etc. Similar comment for figure 3 A, D and G. Another example, in Fig 2E, on what basis are some gene sets chosen to be shown in this figure when there are many more significant in the supplementary table.

We thank the Reviewer for their positive feedback and for these comments. Although some answers to the queries could be found within the figure legends, we agree that figures could have been more self-explanatory, and we amended them accordingly. We have also added information into the main text to clarify those specific points.
In response to the specific queries:
- All enrichment heat maps were generated from GO lists or KEGG pathways.
- 2C: these were chosen instead of other myogenic or cell migration markers for consistency with our previous study (Figure 2C in Gerli et al Stem Cell Reports 2019).
- 3A, D, G: details of the GO lists used to generate heat maps were available in the relative figure legend.
- 2E: enrichment pathways – we listed pathways shared between at least 2 of the three groups and with relevance to cellular migration.

Figure 4F is impossible to interpret without a clear description of how the subnetwork is extracted, was a list of gene list submitted to string, if so which genes and why? Secondly, why are there many nodes with no edges? Is it all of the nodes that are in that GO-Term, if so it needs to be clarified? Was this the most strongly deregulated go-Term according to string analysis?

We thank the Reviewer for this comment. This specific GO list was selected for its highly relevant title/topic, i.e.: “positive regulation of cell migration”. Details on this point could also be found in the specific figure legend, where we specified how the network is extracted and

constructed. There are several nodes with no edges as the edges represent predicted functional association and therefore, a lack of edges suggests a lack of interaction.

Figure 4 B, C, D and E:
(1) The authors should clarify what figure 4B is? Is 1,2,3,4 different time point? Treated or untreated cells?

We apologise with the Reviewer for not having provided enough information on this point. 1,2,3 and 4 were four sequential time points of untreated cells. We have removed those images from the updated figure and inserted more informative trajectory plots.

(2) Figure C: Is the graph showing the cell distribution of both treated and untreated cells? If yes is it possible to give a different shape for the control cells and see if indeed more control green shape would be observed in this plot? (In the supplementary data there is the distribution showing the treated v untreated, but the clusters are not visible)

We thank the Reviewer for this helpful comment. We have attempted to visualise control and treated cells as different shapes but as both conditions occupy the same motility state space and as differential localisation of the state space between conditions is not readily visible, we felt that visualising control and treated cells with different shapes did not aid interpretation of the data. Instead, we have moved the control and treated t-SNE plots adjacent to the t-SNE plots of clusters as figure 4B.

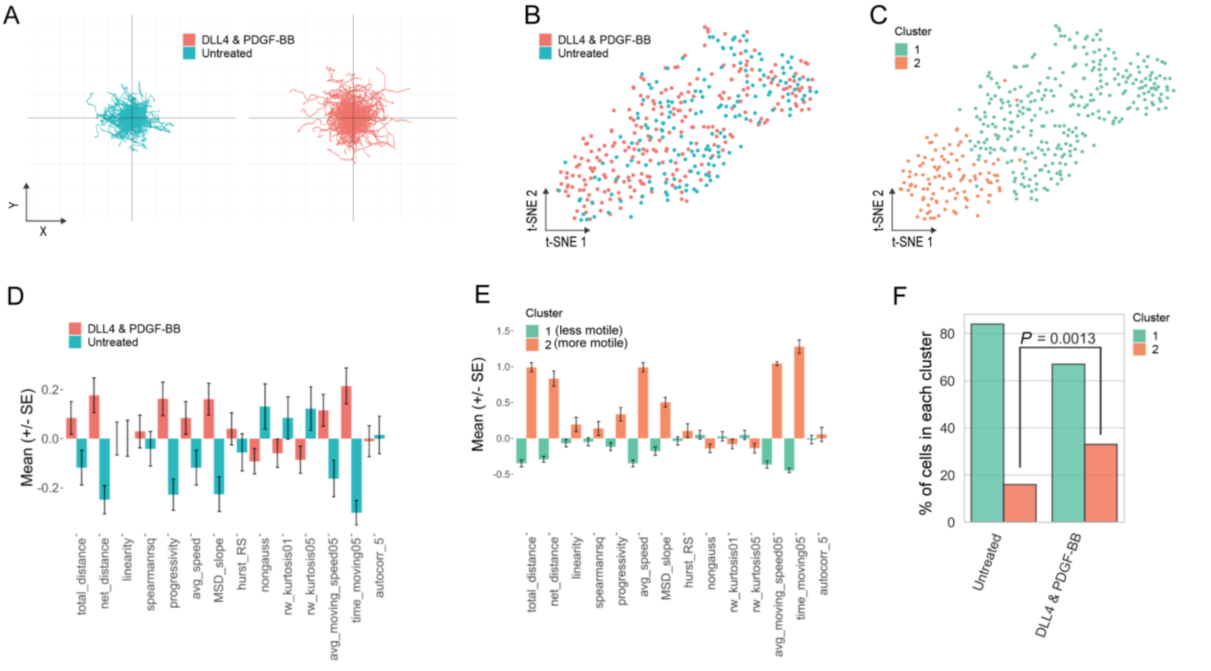

New Figure 4A-F. (A) *Trajectory plots for visualisation of the migratory paths of cells plated on uncoated plastic dishes over the duration of the motility assay. Each line represents the path of a single cell.* ***(B)*** *Visualisation of the motility state space of untreated and DLL4 & PDGF-BB-treated hiMPs using t-SNE plots (perplexity = 35). Top t-SNE plot represents continued exposure to treatment conditions whilst the bottom t-SNE plot represents discontinued exposure to treatment conditions.* ***(C)*** *Unsupervised hierarchical clustering (Ward's method) visualised with a t-SNE plot showing two clusters (Silhouette S_i = 0.22). t-SNE plot on the top represents hiMPs under continued exposure to coatings and treatment whilst the t-SNE plot on the bottom displays hiMPs plated on plastic.* ***(D)*** *Bar*

charts demonstrating normalised values for comparison of motility features between conditions (untreated and DLL4 & PDGF-BB) and ***(E)*** *clusters (mean ±SEM). (****F)*** *Bar graph demonstrating proportions of control and DLL4 & PDGF-BB-treated cells within each cluster for both conditions of continued exposure and discontinued exposure. Hypothesis testing was performed using the chi-squared (χ^2) test.*

(3) Would it be possible to take some of the parameters in Figure 4D and show the distribution in treated vs untreated and perform the statistical analysis? (eg is there a significant difference for the parameter total distance between control and treated?). Or, may be just show some of the results in figure S4C and E in the main text.

We thank the Reviewer for this comment. Following this suggestion, we have moved graphs of figure S4C into the main text (Figure 4D-E).

(4) Why pooling the 3 independent experiment together? Looking at the data in Figure S4, it seems that one treated sample is very similar to the control, thus weakening the conclusion. The replicates in this figure are biological replicates. Yet the papers present 4/5 different cell lines, so why only 3 of them are used here? Is there some explanation regarding the outsider (cell line age, number of division etc). Might be worth adding data from the other cell lines (1 or 2 more).

We thank the Reviewer for this point. The experiment shown in previous figure S4E (current figure EV2 J) has been performed with one cell line (N5) and independent experimental replicates were assessed for the statistical analysis. We are not sure why there appears to be an outlier in some cases, and this is why it was important to replicate this experiment three times. Following this reviewer suggestion, we have performed a similar experiment with an additional cell line (N1) applying more stringent conditions such as absence of the inducing molecules and quantified this with more accurate tools such as Heteromotility rather than Trackmate (new Figure 4A-F, and EV2, below). Data show preferential clustering of treated cells in the more motile cluster with higher distances travelled, average speed and proportion of time spent moving.

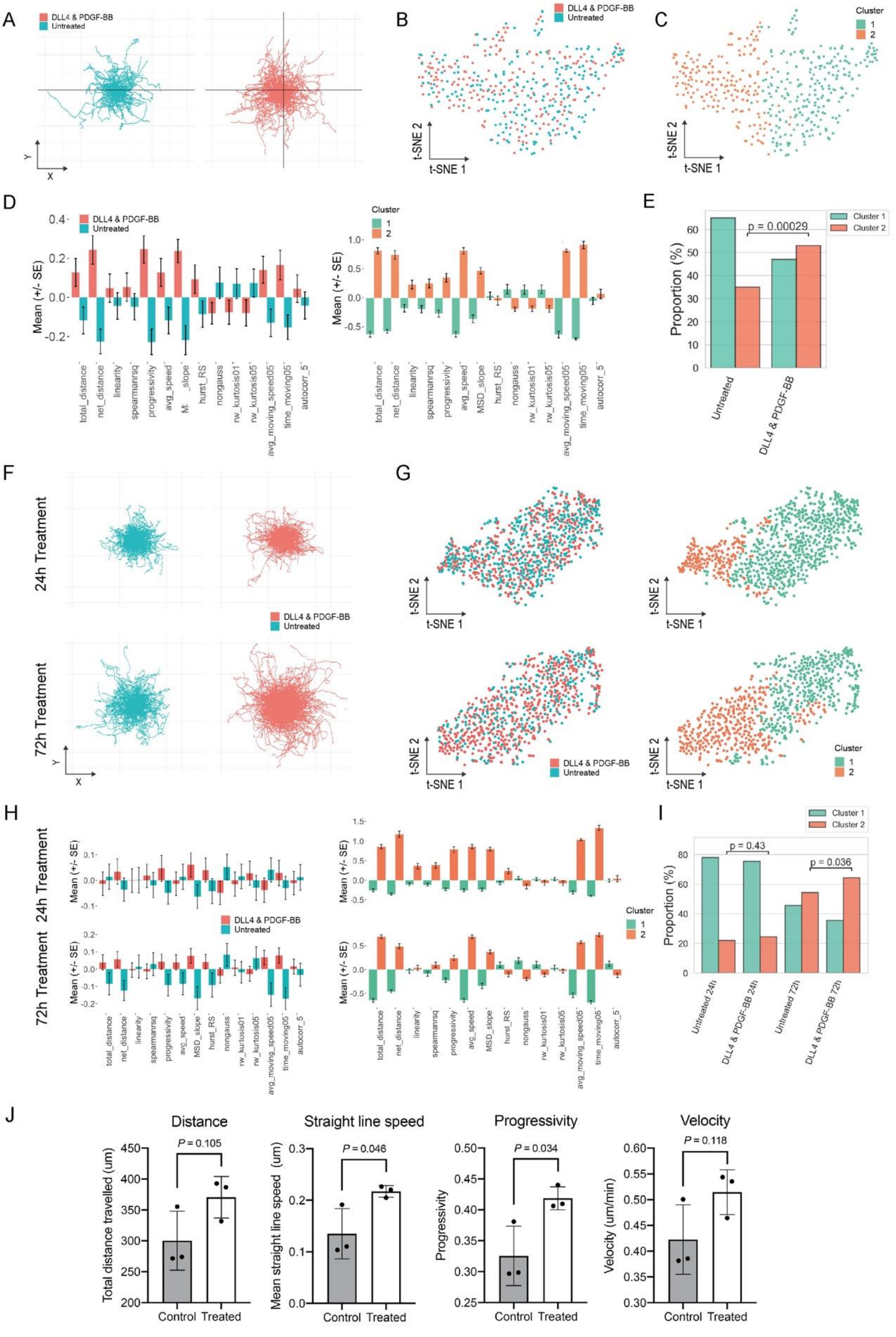

Figure EV2. Additional in vitro motility and migration analyses of treated and untreated hiMPs
(A) *Trajectory plots for visualisation of the migratory paths of untreated and treated cells that were exposed to either 1% BSA or DLL4 & PDGF-BB, respectively, over the course of the motility assay.*

Each line depicts the path of an individual cell. ***(B)*** *Visualisation of the motility state space of untreated and treated hiMPs using t-SNE (perplexity = 35).* ***(C)*** *Hierarchical clustering of the first 30 principal components visualised with a t-SNE plot showing two clusters (Silhouette S_i = 0.19).* ***(D)*** *Bar charts displaying the normalised motility feature values for comparison between conditions: untreated and DLL4 & PDGF-BB (left), cluster 1 and cluster 2 (right).* ***(E)*** *Bar graph showing proportions of untreated and treated hiMPs within the two clusters. Hypothesis testing was performed with a chi-squared (χ^2) test.* ***(F)*** *Trajectory plots for visualisation of hiMP migration after 24 hours of treatment (top row), or 72 hours of treatment (bottom row).* ***(G)*** *t-SNE plots (perplexity = 35) for visualisation of the motility state space of hiMPs in two-dimensions (left). Cluster assignments after hierarchical clustering (S_i = 0.13 (24h); S_i = 0.18 (72h))* ***(H)*** *Bar plots showing normalised motility features for both 24h (top row) and 72h (bottom row) conditions.* ***(I)*** *Bar graph displaying proportions of untreated and DLL4 & PDGF-BB-treated hiMPs treated for 24h and 72h. Hypothesis testing was performed with a chi-squared (χ^2) test* ***(J)*** *Bar graphs depict quantification of parameters obtained from single cell tracking analysed using TrackMate. Motility statistics were calculated for untreated (grey bars) and treated (white bars) hiMPs for 3 biological replicates (n = 3). P values within figure: t-test.*

(5) Figure 4 H and I: What are the statistic actually comparing: treated v untreated for each cell lines or different cell lines against each other? If the former, then how is it possible to have a 139 fold change with such a weak p value of 0.042? If the latter, then why is a p-value given for each of the 3 cell lines? Also, the number and source of replicates is unclear - N=3 is stated, so was each cell line done in triplicate? If so, how many fields per replicate?

We are happy to clarify this point for the Reviewer. The statistical analysis compares treated vs. untreated samples within the same genotype. The high fold change observed is likely due to the large standard deviation of the dataset, which was also highlighted as raw data in the figure panel (bottom part of each picture in white colour font). For this reason, we have repeated this experiment multiple times and validated it across three independent cell lines.

Would additional experiments be essential to support the claims of the paper? Request additional experiments only where necessary for the paper as it is, and do not ask authors to open new lines of experimentation.
It would important to also show the migratory capacity of these cells in vivo.

Are the suggested experiments realistic in terms of time and resources? It would help if you could add an estimated cost and time investment for substantial experiments.
Human muscle cells engraftment and tracking in immunodeficient mice could be easily done. Engrafted muscle can be harvested 2-3 weeks after engraftment, and measurement of the distance from the engraftment point could be done (Site of injection could be labelled with tattoo die). This would be a month/month and half of work. Immunodeficient mice would cost around £1500 (n=6 mice per group => total of 12 mice) plus the cost of housing.

Are the data and the methods presented in such a way that they can be reproduced? Are the experiments adequately replicated and statistical analysis adequate?
See comments in first paragraph. The authors should probably be able to answer easily to the different concerns raised above.

We thank the Reviewer for these comments. We have performed two different in vivo experiments to assess engraftment/differentiation and migration. The engraftment experiment (new figure 3J-K) indicate that treatment does not negatively impact on engraftment upon transplantation. We have also performed the suggested *in vivo* migration experiment (without the tattoo dye due to animal license constraints) but we did not obtain definitive results. For this experiment, we firstly generated two GFP-expressing hiMP lines that were subsequently either exposed to 1% BSA or combined DLL4 and PDGF-BB

treatment for 7 days. 3 x 10^5 hiMPs were intramuscularly injected into each tibialis anterior (TA) of NSG mice (untreated and DLL4 & PDGF-BB-treated hiMPs in the right and left TA, respectively). TAs were collected either 3 or 7 days post-injection to analyse GFP area and intensity. Although a trend was observed in some samples, we were not able to observe significant differences of area or intensity, as measured by corrected total cell fluorescence (CTCF), a measure of intensity normalised by local background intensity, in both cell lines analysed (figure R1 below). This prompted us to retrospectively check if the transplanted cells responded to treatment in the first place by means of gene expression analysis. Real time PCR analysis did not show the usual gene expression changes we normally detect upon treatment, possibly due to the large-scale expansion required by the cells prior to transplantation changing they responsiveness. Due to funding and staffing limitations, we could not repeat this in vivo experiment again with new treated cells. However, this challenge prompted us to develop a novel *quasi vivo*, humanised assay in which time-lapse imaging was performed on acutely injured 3D human artificial muscles grafted with hiMPs, to model migratory behaviour of human cells within a regenerating human 3D environment (new figure 6, details below). This new experiment together with the novel exciting data shown in the new figure 6 (details below) with the organ-on-chip microfluidic device have contributed to significantly reshape the paper towards a more technology-oriented work with a broader remit than just NOTCH and PDGF signalling, as we now describe tools for the community that can be utilised to test a number of other potential treatments.

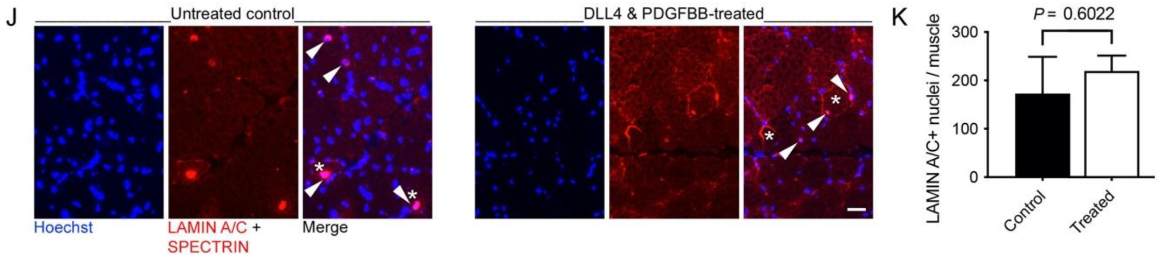

New Figure 3J-K. *Immunofluorescence panels showing human specific LAMIN A/C+ (nuclei) and SPECTRIN (sarcolemma) staining in tibialis anterior muscles of immunodeficient mice (N = 3) transplanted with treated (n = 3 muscles) and untreated (n = 3 muscles) N5 hiMPs.* ***(K)*** *Quantification of LAMIN A/C+ grafted human cells across each muscle.*

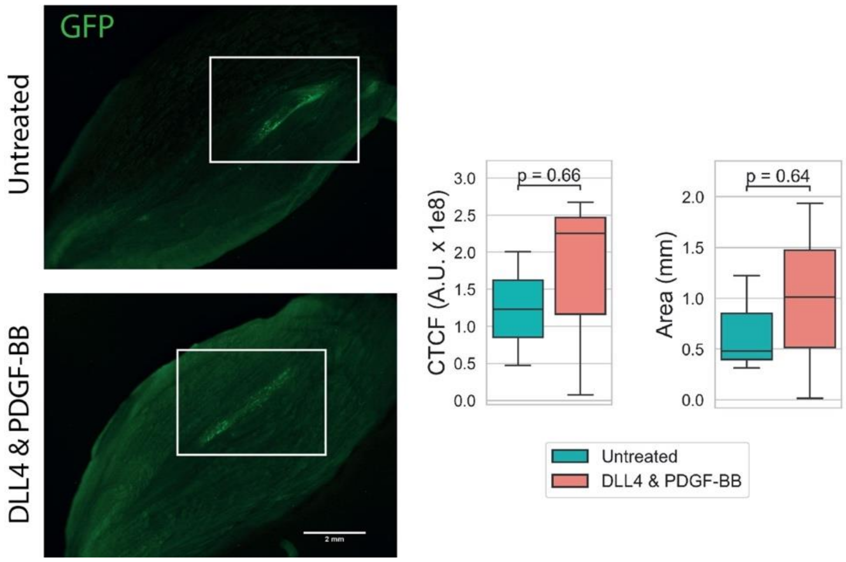

Figure R1. *Assessment of cell migration in vivo of treated and untreated hiMPs upon transplantation*

in NSG mice (not included in revised manuscript). 3 x 10^5 hiMPs were intramuscularly injected into each tibialis anterior muscles (TA) of immunodeficient NSG mice (n = 6; untreated and DLL4 & PDGF-BB-treated hiMPs in the right and left TA, respectively). TAs were collected either 3 or 7 days post-injection to analyse GFP area and intensity.

Minor comments:
typo "Onthology" should be "Ontology" in figure 2E.
Some of the data in Figure S4E should be moved to the main text.

Thanks for highlighting these minor comments. We have corrected the typo and moved some data from figure S4 into the main figure 4.

Reviewer #2 (Evidence, reproducibility and clarity (Required)):
In this manuscript, Ferrari and colleagues provide solid data indicating that the Notch ligand DLL4 and PDGF-BB regulate the migration of myogenic progenitors derived from human pluripotent stem cells (PSC). These studies built from recent work by the same group (Gerli et al, Stem Cell Reports, 12:461, 2019), in which the authors documented that Notch and PDGF-BB signaling enhances migration and expression of stem cell markers while inducing perivascular cell features in muscle satellite cells. Here the authors perform similar in vitro studies in PSC-derived myogenic progenitors and conclude that the same effect is observed in this population of cells. The results are clear and well presented.

Throughout the manuscript, the authors emphasize the importance of such findings for the future therapeutic application of a PSC-based therapy to treat patients with muscular dystrophy since multiples skeletal muscles need to be targeted in this group of diseases. Unfortunately, the authors do not provide transplantation data. The results would be highly meaningful if they show that observed in vitro changes (transcriptomes and chamber assay) result in meaningful migration in vivo using the systemic delivery, but as it is, the data do not support the claims and conclusions.

We thank Reviewer 2 for their comments. We were pleased to read that they found our study and data solid, clear and well presented. Although we agree with the Reviewer that in vivo evidence would strengthen our findings, we would like to highlight that our study did not aim to be a translational investigation of the therapeutic potential of treated hPSC derivatives for muscle cell therapy (we believe our previous and current manuscript's title reflect this). We see this work more as a foundational study to establish the required evidence for future, follow up transplantation studies focused on the therapeutic potential of this approach (something requiring a dedicated project, funding and months/years of work). Nonetheless, we have performed two different in vivo experiments to assess engraftment/differentiation and migration. The engraftment experiment (new figure 3J-K, above) indicate that treatment does not negatively impact on engraftment upon transplantation. However, we believe that xenogeneic transplants are of limited use to investigate a complex species-specific phenomenon such as transendothelial cell migration. For this very reason we moved back to intraspecific (i.e., murine cells into mouse recipients) transplants in past studies (e.g., Tedesco et al Sci Transl Med 2012). However, as a key aim of our study is to obtain data specific to human cells and given that we already performed mouse-in-mouse *in vivo* intra-arterial delivery experiments using DLL4 and PDGFBB treated primary cells in Gerli *et al.* Stem Cell Reports 2018, we have:

1) Investigated trans-endothelial migration within an organ-on-chip system to model intraspecific (human-in-human) trans-endothelial migration under perfusion.
2) Assessed migration of hiMPs within an acutely injured humanised, *quasi vivo* 3D muscle platform.

To investigate the effect of DLL4 & PDGF-BB on hiMPs during trans-endothelial migration under flow conditions, we utilised the OrganoPlate platform (Mimetas). Each chip of the OrganoPlate is comprised of three channels, a top perfusion channel, central ECM channel and bottom perfusion channel (new Figure 5). 3D blood vessels were generated on a collagen gel within the central ECM channel. Additionally, a rocker-based system facilitated induction of consistent flow within the chips. This system allowed us to independently investigate two aspects of trans-endothelial migration, namely adhesion and extravasation and revealed that the increased trans-endothelial migration is mediated by enhanced extravasation, and not adhesion.

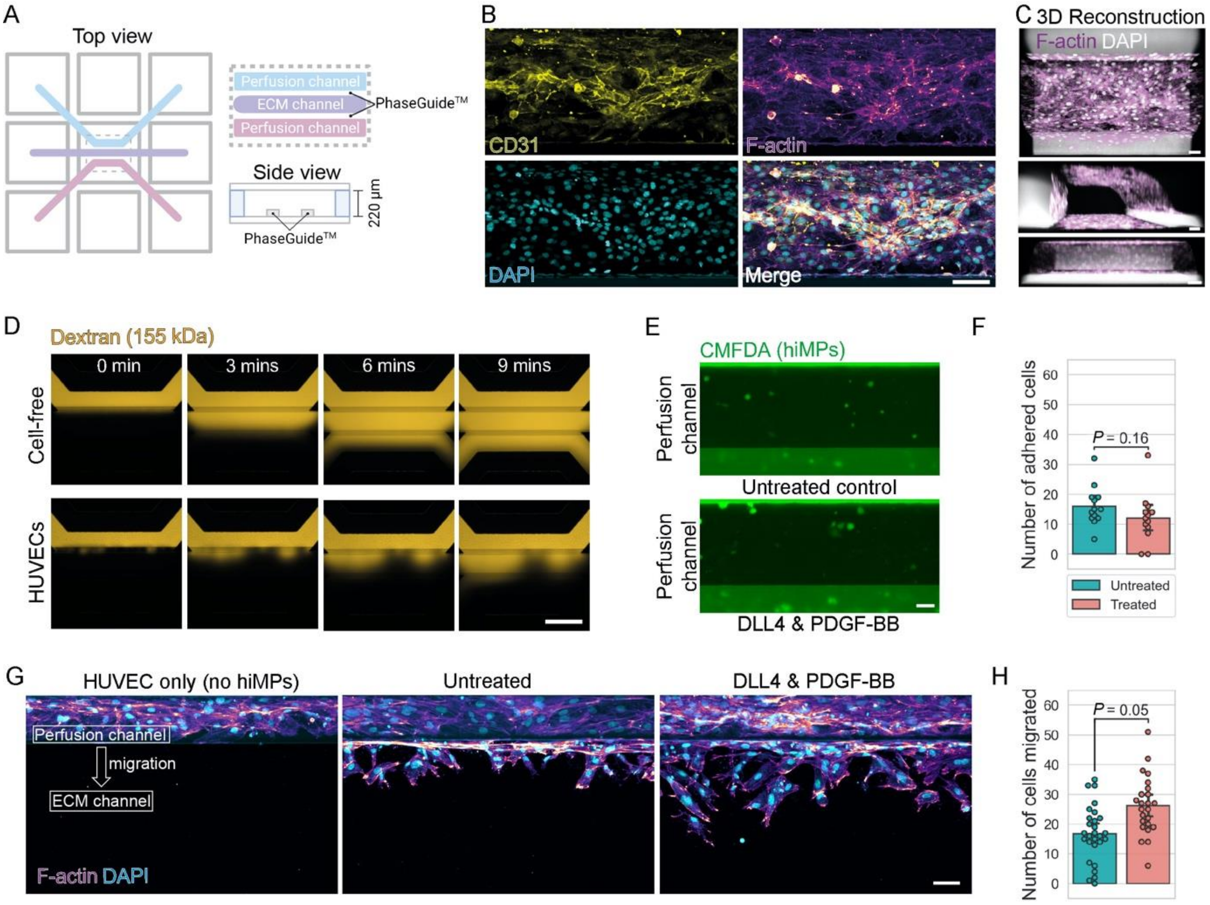

New Figure 5. Modelling extravasation of hiMPs using microfluidic, organ-on-chip devices. ***(A)*** *Graphical representation of an individual chip of the OrganoPlate®. Each chip consists of a top perfusion channel, central ECM channel and bottom perfusion channel. Phase guides between channels allows for generation of surface tension after deposition of collagen-I within the ECM channel so that there is no physical barrier between the collagen gel and perfusion channels. This facilitates generation of a 3D blood vessel that is in direct contact with the ECM channel.* ***(B)*** *Maximum intensity projections of the top perfusion channel, 48h after seeding HUVECs, immunostained for CD31 and F-actin. Scale bar: 100µm.* ***(C)*** *3D projections of blood vessel-like tubules of the top perfusion channel stained for F-actin. Scale bar: 50µm.* ***(D)*** *Representative fluorescence images of 150 kDa TRITC-conjugated dextran added to the top perfusion channel of OrganoPlate® chips with and without 3D endothelial monolayers generated by HUVECs. Chips were imaged every 3 minutes. See Figure S5 for extended panel and quantification. Scale bar: 100 µm* ***(E)*** *Representative fluorescence images of CMFDA-stained untreated and DLL4 & PDGF-BB-treated hiMPs within the top perfusion channel, 15 minutes after delivery and kept on the OrganoFlow®. Scale bar: 50µm.* ***(F)*** *Bar graph quantifying adhesion images in (E). Statistical significance was calculated based on a paired t-test (n = 3). Each point on the plot represents the number of adhered cells after 15 minutes within a single chip to visualise the distribution of data.* ***(G)*** *Maximum intensity projections of DLL4 & PDGF-BB-treated and untreated hiMPs as well as HUVECs stained for F-actin. Scale bar: 50µm.* ***(H)*** *Bar chart quantifying the number of nuclei within the central, ECM channel.*

Each point represents the number of nuclei within the ECM channel for a single chip. Hypothesis testing was performed with a paired t-test, with experimental replicates as datapoints (N = 3).

We have additionally set up a novel assay to evaluate the migratory properties of hiMPs within a 3D environment. This involved live-imaging of hiMPs deposited on 3D artificial muscles that were acutely injured with cardiotoxin (see Materials and Methods). Single-cell tracking of untreated and DLL4 & PDGF-BB-treated hiMPs revealed the treatment resulted in increased total distance travelled for hiMPs. Additional clustering analysis also suggested the existence of much larger proportion of migratory hiMPs after treatment with DLL4 & PDGF-BB which is consistent with results obtained in conventional bidimensional motility assays (new figure 6).

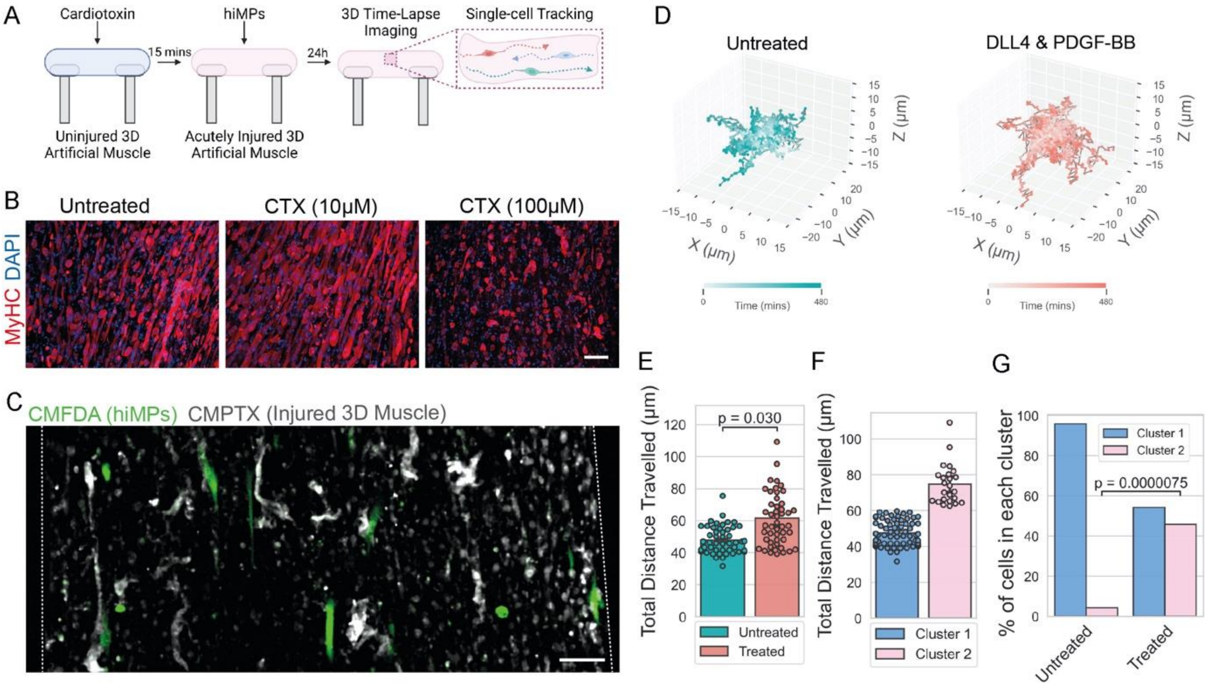

***New Figure 6. Quasi vivo modelling of hiMP tissue migration using 3D human bioengineered muscles. (A)** Schematic representation of the experimental setup. **(B)** Immunofluorescence images of 3D artificial muscles stained for myosin heavy chain (MyHC), after 15 minutes exposure to PBS (uninjured) or 10μM/100μM cardiotoxin. Scale bar: 50μm. **(C)** Maximum intensity projections of fluorescence images of 3D muscles after background subtraction, stained with CMPTX deposited with CMFDA-stained hiMPs. Dotted lines demarcate the outline of the 3D construct. Scale bar: 100 μm. **(D)** 3D trajectory plots for visualisation of single-cell tracks of hiMPs on 3D muscles for 8 hours for both untreated (left) and DLL4 & PDGF-BB-treated (right) conditions. **(E)** Bar chart representing the total distances travelled of single-cells tracked for DLL4 & PDGF-BB and untreated hiMPs. Statistical testing was performed with an independent t-test with each experimental replicate as data points (n = 3). Velocities of individual cells are displayed as single points to visualise the distribution of data. **(F)** Bar chart displaying the velocity of cells within clusters generated using hierarchical clustering of cells using total distance travelled as a feature (S_i = 0.67). Each point represents a single cell. **(G)** Bar plots showing the proportions of untreated and DLL4 & PDGF-BB-treated cells within the two clusters shown in (I). Statistical test performed with a Chi-squared (χ^2) test.*

Reviewer #2 (Significance (Required)):
Significance is limited if only in vitro data are provided. However if the authors are able to show enhanced engraftment upon systemic transplantation of human PSC-derived myogenic progenitors upon DLL4 and PDGF-BB treatment, the significance would be high.

Please see our reply to the previous point.

In terms of existing literature, there are publications reporting systemic delivery of murine PSC-derived myogenic progenitors as well as transcriptome and in vitro migration studies. It would probably be appropriate to cite them.

We apologise to the Reviewer for this oversight. We have added the following papers which include systemic delivery of murine PSC-derived myogenic progenitors as well as transcriptome and migration studies: Matthias N *et al*., Exp Cell Res 2015; Incitti T *et al*., PNAS 2019.

If systemic engraftment is observed, the manuscript would be of interest to the skeletal muscle and stem cell biology/regenerative medicine community.

Please see our reply to the initial point.

Reviewer #3 (Evidence, reproducibility and clarity (Required)):

In this manuscript, the authors exploited the signal-mediated activation of NOTCH and PDGF pathways, by one week-long delivery of DLL4 and PDGF-BB to cultures of hiPSC-derived myogenic progenitors in vitro, to improve their migration ability. They performed transcriptomic and functional analyses across human and mouse primary muscle stem cells and human hiPSC-derived myoblasts, including genetically corrected hiPSC derivatives, to show that DLL4 and PDGF-BB treatment modulates pathways involved in cell migration, including enhanced trans-endothelial migration in transwell assays.
The increased migratory ability, and in particular enhancing extravasation, is a fundamental property required for optimal performance of hiPSC myogenic derivatives, upon their intravascular delivery; hence, the finding reported here are of extremely high potential interest in term of solution of one of the major bottle-neck of cell therapy.
However, there are important issues that need to be resolved by the authors with additional experimentation, that I recommend performing, in order to improve this manuscript.

We sincerely thank the Reviewer for acknowledging the extremely high relevance and potential of our paper for muscle gene and cell therapies and for providing constructive feedback to improve our manuscript.

1) The most critical issue here is that the authors fail to provide evidence that DLL4/PDGF-BB-treated cultures of hiPSC-derived myogenic progenitors do not lose their myogenic potential and are able to form myotubes, upon interruption of treatment. It would be also important to determine when (how many days after withdrawal of DLL4/PDGF-BB) the full myogenic properties of these cells are recovered. From the RNAseq datasets shown by the authors, it appears that DLL4/PDGF-BB-treated hiPSC-derived myoblasts do not express the key genes of myogenic identity (MyoD) and early differentiation (myogenin), while expressing genes of mesenchymal/vessel-derived lineages. It is imperative that the authors

show that these changes are reversible, upon withdrawal of DLL4/PDGF-BB. This should be show by an unbiased transcriptomic analysis (RNAseq) of hiPSC-derived myoblasts after withdrawal of DLL4/PDGF-BB, that should be integrated with functional evidence showing that these cells can resume their ability to form differentiated myotubes, upon exposure to myogenic culture cues in vitro.

We thank the Reviewer for this comment. We agree that this is an important experiment for our work. We performed similar work in our previous study and already observed phenotype reversion of treated cells upon release of the stimuli within a few passages in cultures. To investigate the dynamics of phenotype reversion of DLL4 & PDGF-BB treatment on hiMPs, we performed a reversal assay in which hiMPs were transferred onto uncoated surfaces after 7 days of exposure to either 1% BSA or DLL4 and PDGF-BB. We subsequently performed immunofluorescence staining of cultures differentiated after 3 days in proliferation without DLL4 and PDGF-BB to determine the extent of phenotype reversal after ceasing treatment. These new data indicate that reversion of differentiation impairment takes place spontaneously upon removal of DLL4 and PDGF-BB, with increasing myogenic differentiation noticeable from day 3 of removal of the stimuli onwards (new Appendix Fig S3, below).

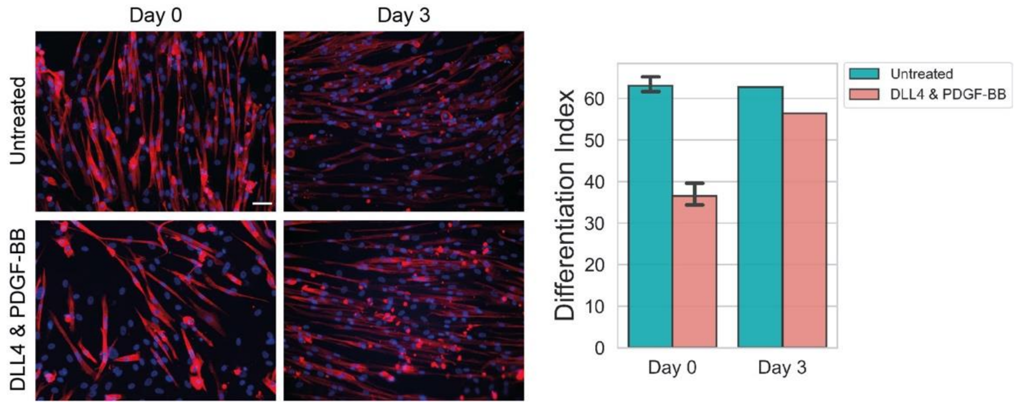

Appendix Figure S3. Assessment of spontaneous differentiation of DLL4 & PDGF-BB treated hiMPs. (A) *Representative immunofluorescence images of untreated and DLL4 & PDGF-BB-treated hiMPs differentiated into myotubes for 4 days either immediately after 7 days of treatment or after proliferation of uncoated plastic dishes for 3 days.* ***(B)*** *Bar graph quantifying the differentiation of images in* ***(A)*** *using the differentiation index (n = 3 for Day 0, n = 1 for Day 3). Scale bar = 50µm.*

2) A parallel evidence in vivo should be also provided, showing that DLL4/PDGF-BB-treated hiPSC-derived myoblasts do not express MyoD and myogenin when delivered intravascularly, but regain their expression after they have crossed the vessel endothelium and have entered the skeletal muscles.

We thank the Reviewer for suggesting this experiment. We agree that this would be a very interesting point to address; however, it might be very challenging to address this question with the proposed *in vivo* experiment. Nonetheless, we believe that new *in vitro* and *in vivo* data provided in the revised manuscript help addressing this point. Specifically, the aforementioned reversal experiment provide in vitro evidence that the cells do not lose their myogenic capacity. Additionally, the engraftment experiment shown in the new figure 3J-K indicate that treatment does not negatively impact on engraftment upon transplantation.

If these experiments could firmly demonstrate that DLL4/PDGF-BB-treatment reversibly promotes migratory properties of hiPSC-derived myoblasts (as predicted, but not demonstrated in previous works from the same group, using mouse or human primary muscle stem cells - Cappellari et al. 2013; Gerli et al. 2019), then this work could be a great interest in term of basic and translational biology and clearly suitable for publication in a top journal.

We thank the Reviewer for this constructive feedback and for seeing the great potential of our work in terms of basic and translational biology. We assume there was a typo in the sentence in brackets with a missing “as” (“..not demonstrated as in previous work...”): we indeed demonstrated the effect of DLL4 and PDGFBB *in vivo* extensively in our previous work.

Other points:

- Fig. 2A. it looks like there are some outlier RNAseq sample replicates that might negatively impact at the statistical level on the subsequent analysis. This issue is likely due to the heterogeneity of the samples (both untreated and treated) and could be resolved by replacing outlier samples with new replicates.

We thank the Reviewer for this comment. Although we agree that replacing those samples with new replicates might improve our statistical analyses, this will be financially challenging at this stage and perhaps also not completely reflecting the real variability of the experimental setup. Overall this level of variability has been taken into account by having at least 3 biological replicates, and we believe that presenting the full spectrum of biological variability is of scientific value for future replications.

- Along the same line as above, sample heterogeneity following treatment might be resolved by a better understanding of optimal doses of DLL4/PDGF-BB and time of exposure, which I recommend the authors to define by additional experiments.

We thank the Reviewer for this comment. This is a potentially interesting experiment, which we have not performed initially as we took advantage of previous knowledge and dose-response on primary mouse and human myoblasts. To reduce the number of variables to investigate we limited this analysis to time of exposure. The new data provided as a novel extended figure and pasted also below show that there is indeed a time dependent effect noticeable up to 7 days of treatment, with statistically significant differences noticeable from day 3 of treatment when assessing cells for their motility properties.

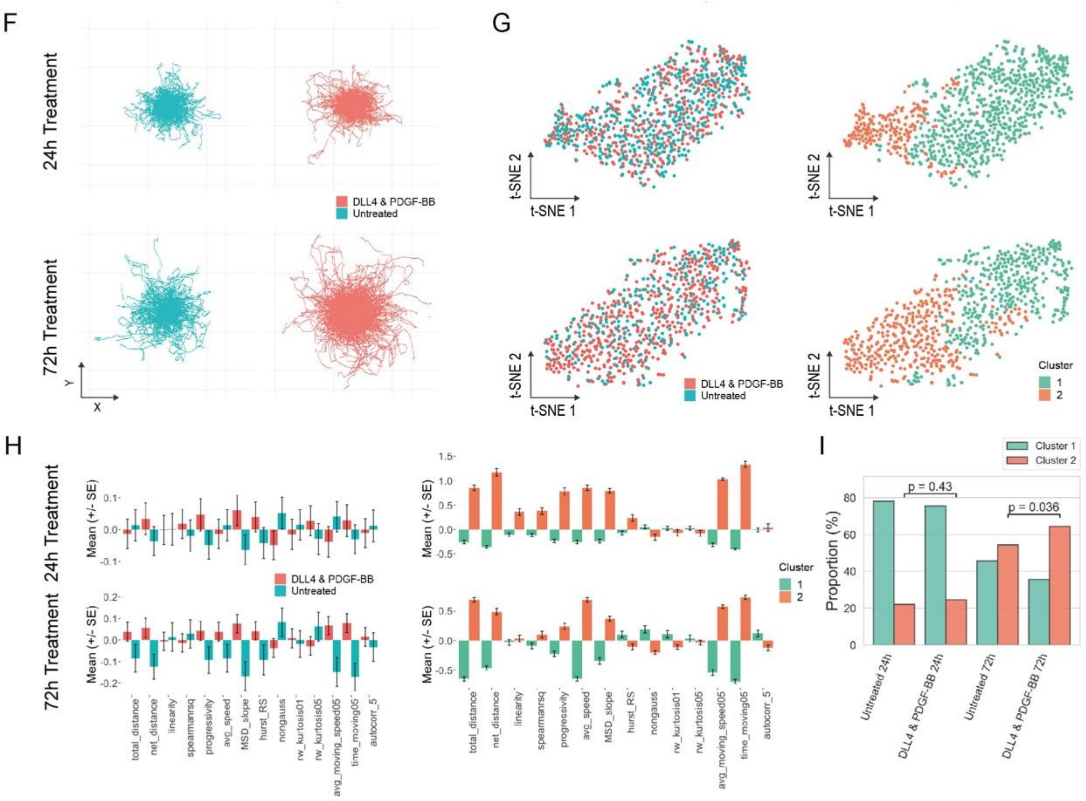

From new Figure EV2. Additional in vitro motility and migration analyses of treated and untreated hiMPs

(F) *Trajectory plots for visualisation of hiMP migration after 24 hours of treatment (top row), or 72 hours of treatment (bottom row).* ***(G)*** *t-SNE plots (perplexity = 35) for visualisation of the motility state space of hiMPs in two-dimensions (left). Cluster assignments after hierarchical clustering (S_i = 0.13 (24h); S_i = 0.18 (72h))* ***(H)*** *Bar plots showing normalised motility features for both 24h (top row) and 72h (bottom row) conditions.* ***(I)*** *Bar graph displaying proportions of untreated and DLL4 & PDGF-BB-treated hiMPs treated for 24h and 72h. Hypothesis testing was performed with a chi-squared (χ^2) test.*

Reviewer #3 (Significance (Required)):

If these experiments could firmly demonstrate that DLL4/PDGF-BB-treatment reversibly promotes migratory properties of hiPSC-derived myoblasts (as predicted, but not demonstrated in previous works from the same group, using mouse or human primary muscle stem cells - Cappellari et al. 2013; Gerli et al. 2019), then this work could be a great interest in term of basic and translational biology and clearly suitable for publication in a top journal and could be interesting for a wide audience in regenerative medicine.

We thank the Reviewer once again for this constructive feedback and for seeing the great potential of our work in terms of basic and translational biology, as well as for regenerative medicine.

Once again, we sincerely thank all Reviewers for their positive, constructive and insightful comments, which motivated us to further improve our work. We also thank the Review Commons and EMBO Molecular Medicine editorial teams for guidance and assistance.

Sincerely,

Prof. Francesco Saverio Tedesco
University College London, Great Ormond Street Hospital and The Francis Crick Institute, London, UK

5th Apr 2022

Dear Prof. Tedesco,

Thank you for the submission of your manuscript to EMBO Molecular Medicine. I am pleased to inform you that we will be able to accept your manuscript pending the following final amendments:

1) In the main manuscript file, please do the following:
- Correct/answer the track changes suggested by our data editors by working from the attached document.
- Add up to 5 keywords.
- Make sure that all special characters display well.
- In M&M, provide the antibody dilutions that were used for each antibody.
- In M&M, a statistical paragraph should reflect all information that you have filled in the Authors Checklist, especially regarding randomization, blinding, replication.
- Raw data from large-scale datasets (RNA sequencing) should be deposited in one of the relevant databases and made freely available prior the publication of the manuscript. Use the following format to report the accession number of your data:

The datasets produced in this study are available in the following databases:
[data type]: [full name of the resource] [accession number/identifier] ([doi or URL or identifiers.org/DATABASE:ACCESSION])

Please check "Author Guidelines" for more information.
https://www.embopress.org/page/journal/17574684/authorguide#availabilityofpublishedmaterial
2) Tables: Table with primers should be labeled Table 2 with a short description.
3) Dataset: "Supplemental File" should be renamed to Dataset EV1, and its legend removed legends from the main manuscript and added to the file. Please also update callouts in the main text.
4) Movies: Please rename movie files to Movie EV1 and Movie EV2, remove their legends from the main manuscript and zipp them with the corresponding movie file.
5) Funding: Please make sure that information about all sources of funding are complete in both our submission system and in the manuscript. Duchenne Children's Trust / Duchenne UK and Duchenne Research Fund;
19GRO-PS48-0188; 17GRO-PS48-0093-1 is missing in our submission system.
6) Conflict of interest: Rename "Conflict of interest" to "Disclosure Statement & Competing Interests". We updated our journal's competing interests policy in January 2022 and request authors to consider both actual and perceived competing interests. Please review the policy https://www.embopress.org/competing-interests and update your competing interests if necessary.
7) Synopsis: Every published paper now includes a 'Synopsis' to further enhance discoverability. Synopses are displayed on the journal webpage and are freely accessible to all readers. They include separate synopsis image and synopsis text.
- Synopsis text: Please provide a short stand first (maximum of 300 characters, including space) as well as 2-5 one sentence bullet points that summarise the paper as a .doc file. Please write the bullet points to summarise the key NEW findings. They should be designed to be complementary to the abstract - i.e. not repeat the same text. We encourage inclusion of key acronyms and quantitative information (maximum of 30 words / bullet point). Please use the passive voice.
- Synopsis image: Please provide a striking image or visual abstract as a high-resolution jpeg file 550 px-wide x (250-400)-px high to illustrate your article.
- Please check your synopsis text and image and submit their final versions with your revised manuscript. Please be aware that in the proof stage minor corrections only are allowed (e.g., typos).
8) Source data: We encourage you to include the source data for figure panels that show essential data. Numerical data should be provided as individual .xls or .csv files (including a tab describing the data). For blots or microscopy, uncropped images should be submitted (using a zip archive if multiple images need to be supplied for one panel). Please check "Author Guidelines" for more information. https://www.embopress.org/page/journal/17574684/authorguide#sourcedata
9) As part of the EMBO Publications transparent editorial process initiative (see our Editorial at http://embomolmed.embopress.org/content/2/9/329), EMBO Molecular Medicine will publish online a Review Process File (RPF) to accompany accepted manuscripts. This file will be published in conjunction with your paper and will include the anonymous referee reports, your point-by-point response and all pertinent correspondence relating to the manuscript. Let us know whether you agree with the publication of the RPF and as here, if you want to remove or not any figures from it prior to publication. Please note that the Authors checklist will be published at the end of the RPF.
10) Please provide a point-by-point letter INCLUDING my comments as well as the reviewer's reports and your detailed responses (as Word file).

I look forward to reading a new revised version of your manuscript as soon as possible.

Yours sincerely,

Zeljko Durdevic

Zeljko Durdevic
Editor
EMBO Molecular Medicine

***** Reviewer's comments *****

Referee #1 (Comments on Novelty/Model System for Author):

The authors used adequate in vitro and in vivo models to show that DLL4 and PDGFBB-treated myogenic precursors present a greater capacity to migrate, while the capacity to differentiate is restitute when DDL4-PDGFBB is removed. These findings could be transferred toward future therapeutic application of a PSC-based therapy to treat patients with muscular dystrophy.

Referee #1 (Remarks for Author):

The article by Choi et al is now a solid paper showing that the activation of NOTCH - PDFG-BB pathways can improve the migration capacity of hiPSC-derived myogenic progenitors.

I can see that the paper has been greatly improved by the addition of new data, and that the authors made a great effort to answer all my concerns.

I also appreciate that the authors added two sets of experiments and explained in their response the limitations they encounter.

I also agree with the authors that xenogeneic transplants are very limited models, and that it is important to work on human-human model to study the migratory capacity of human muscle progenitor. In this context, the experiment using an organ-on-chip microfluidic model is very elegant, and convincing.

Referee #2 (Remarks for Author):

The authors have performed an excellent revision and have been responsive to all reviewer's comments. The manuscript is, in the opinion of this reviewer, suitable for publication

***** Reviewer's comments *****

Referee #1 (Comments on Novelty/Model System for Author):

The authors used adequate in vitro and in vivo models to show that DLL4 and PDGFBB-treated myogenic precursors present a greater capacity to migrate, while the capacity to differentiate is restitute when DDL4-PDGFBB is removed. These findings could be transferred toward future therapeutic application of a PSC-based therapy to treat patients with muscular dystrophy.

Referee #1 (Remarks for Author):

The article by Choi et al is now a solid paper showing that the activation of NOTCH - PDFG-BB pathways can improve the migration capacity of hiPSC-derived myogenic progenitors.

I can see that the paper has been greatly improved by the addition of new data, and that the authors made a great effort to answer all my concerns.

I also appreciate that the authors added two sets of experiments and explained in their response the limitations they encounter.

I also agree with the authors that xenogeneic transplants are very limited models, and that it is important to work on human-human model to study the migratory capacity of human muscle progenitor. In this context, the experiment using an organ-on-chip microfluidic model is very elegant, and convincing.

We thank Reviewer 1 for their positive assessment of our revised manuscript and for acknowledging the potential of our work for future therapeutic application of PSC-based therapies of muscle disorders.

Referee #2 (Remarks for Author):

The authors have performed an excellent revision and have been responsive to all reviewer's comments. The manuscript is, in the opinion of this reviewer, suitable for publication.

We also thank Reviewer 2 for their kind words and positive feedback.

2nd Revision - Editorial Decision 19th Aug 2022

We are pleased to inform you that your manuscript is accepted for publication and is now being sent to our publisher to be included in the next available issue of EMBO Molecular Medicine.

EMBO Press Author Checklist

Corresponding Author Name: F.S. Tedesco	
Journal Submitted to: EMBo Mol Med	
Manuscript Number: EMM-2021-14526 | [RC-2021-00693] [REV]	

USEFUL LINKS FOR COMPLETING THIS FORM
The EMBO Journal - Author Guidelines
EMBO Reports - Author Guidelines
Molecular Systems Biology - Author Guidelines
EMBO Molecular Medicine - Author Guidelines

Reporting Checklist for Life Science Articles (updated January 2022)
This checklist is adapted from Materials Design Analysis Reporting (MDAR) Checklist for Authors. MDAR establishes a minimum set of requirements in transparent reporting in the life sciences (see Statement of Task: 10.31222/osf.io/9sm4x). Please follow the journal's guidelines in preparing your manuscript.
Please note that a copy of this checklist will be published alongside your article.

Abridged guidelines for figures
1. Data
The data shown in figures should satisfy the following conditions:
- the data were obtained and processed according to the field's best practice and are presented to reflect the results of the experiments in an accurate and unbiased manner.
- ideally, figure panels should include only measurements that are directly comparable to each other and obtained with the same assay.
- plots include clearly labeled error bars for independent experiments and sample sizes. Unless justified, error bars should not be shown for technical replicates.
- if n<5, the individual data points from each experiment should be plotted. Any statistical test employed should be justified.
- Source Data should be included to report the data underlying figures according to the guidelines set out in the authorship guidelines on Data Presentation.

2. Captions
Each figure caption should contain the following information, for each panel where they are relevant:
- a specification of the experimental system investigated (eg cell line, species name).
- the assay(s) and method(s) used to carry out the reported observations and measurements.
- an explicit mention of the biological and chemical entity(ies) that are being measured.
- an explicit mention of the biological and chemical entity(ies) that are altered/varied/perturbed in a controlled manner.
- the exact sample size (n) for each experimental group/condition, given as a number, not a range;
- a description of the sample collection allowing the reader to understand whether the samples represent technical or biological replicates (including how many animals, litters, cultures, etc.).
- a statement of how many times the experiment shown was independently replicated in the laboratory.
- definitions of statistical methods and measures:
  - common tests, such as t-test (please specify whether paired vs. unpaired), simple χ2 tests, Wilcoxon and Mann-Whitney tests, can be unambiguously identified by name only, but more complex techniques should be described in the methods section;
  - are tests one-sided or two-sided?
  - are there adjustments for multiple comparisons?
  - exact statistical test results, e.g., P values = x but not P values < x;
  - definition of 'center values' as median or average;
  - definition of error bars as s.d. or s.e.m.

Please complete ALL of the questions below.
Select "Not Applicable" only when the requested information is not relevant for your study.

Materials

Newly Created Materials	Information included in the manuscript?	In which section is the information available? (Reagents and Tools Table, Materials and Methods, Figures, Data Availability Section)
New materials and reagents need to be available; do any restrictions apply?	Not Applicable	European Nucleotide Archive (ENA) study accession number: PRJEB43338

Antibodies	Information included in the manuscript?	In which section is the information available? (Reagents and Tools Table, Materials and Methods, Figures, Data Availability Section)
For **antibodies** provide the following information:<br>- Commercial antibodies: RRID (if possible) or supplier name, catalogue number and or/clone number<br>- Non-commercial: RRID or citation	Yes	M&M

DNA and RNA sequences	Information included in the manuscript?	In which section is the information available? (Reagents and Tools Table, Materials and Methods, Figures, Data Availability Section)
**Short novel DNA or RNA including primers, probes**: provide the sequences.	Yes	M&M

Cell materials	Information included in the manuscript?	In which section is the information available? (Reagents and Tools Table, Materials and Methods, Figures, Data Availability Section)
**Cell lines:** Provide species information, strain. Provide accession number in repository **OR** supplier name, catalog number, clone number, and/**OR** RRID.	Yes	M&M
**Primary cultures:** Provide species, strain, sex of origin, genetic modification status.	Yes	M&M
Report if the cell lines were recently **authenticated** (e.g., by STR profiling) and tested for mycoplasma contamination.	Not Applicable	STR and mycoplasma done routinely in house at the Francis Crick Institute

Experimental animals	Information included in the manuscript?	In which section is the information available? (Reagents and Tools Table, Materials and Methods, Figures, Data Availability Section)
**Laboratory animals or Model organisms:** Provide species, strain, sex, age, genetic modification status. Provide accession number in repository **OR** supplier name, catalog number, clone number, **OR** RRID.	Yes	M&M
**Animal observed in or captured from the field:** Provide species, sex, and age where possible.	Not Applicable	
Please detail **housing and husbandry conditions**.	Yes	Home Office Licence number provided in M&M

Plants and microbes	Information included in the manuscript?	In which section is the information available? (Reagents and Tools Table, Materials and Methods, Figures, Data Availability Section)
**Plants:** provide species and strain, ecotype and cultivar where relevant, unique accession number if available, and source (including location for collected wild specimens).	Not Applicable	
**Microbes:** provide species and strain, unique accession number if available, and source.	Not Applicable	

Human research participants	Information included in the manuscript?	In which section is the information available? (Reagents and Tools Table, Materials and Methods, Figures, Data Availability Section)
If collected and within the bounds of privacy constraints report on age, sex and gender or ethnicity for all study participants.	Not Applicable	

Core facilities	Information included in the manuscript?	In which section is the information available? (Reagents and Tools Table, Materials and Methods, Figures, Data Availability Section)
If your work benefited from core facilities, was their service mentioned in the acknowledgments section?	Yes	Acknowledgements

Design

Study protocol	Information included in the manuscript?	In which section is the information available? (Reagents and Tools Table, Materials and Methods, Figures, Data Availability Section)
If study protocol has been **pre-registered, provide DOI in the manuscript**. For clinical trials, provide the trial registration number **OR** cite DOI.	Not Applicable	
Report the **clinical trial registration number** (at ClinicalTrials.gov or equivalent), where applicable.	Not Applicable	

Laboratory protocol	Information included in the manuscript?	In which section is the information available? (Reagents and Tools Table, Materials and Methods, Figures, Data Availability Section)
Provide DOI OR other citation details if **external detailed step-by-step protocols** are available.	Not Applicable	

Experimental study design and statistics	Information included in the manuscript?	In which section is the information available? (Reagents and Tools Table, Materials and Methods, Figures, Data Availability Section)
Include a statement about **sample size** estimate even if no statistical methods were used.	Yes	M&M
Were any steps taken to minimize the effects of subjective bias when allocating animals/samples to treatment (e.g. **randomization procedure**)? If yes, have they been described?	Yes	Blinding
Include a statement about **blinding** even if no blinding was done.	Yes	M&M
Describe **inclusion/exclusion criteria** if samples or animals were excluded from the analysis. Were the criteria pre-established?<br><br>If sample or data points were omitted from analysis, report if this was due to attrition or intentional exclusion and provide justification.	Not Applicable	
For every figure, are **statistical tests** justified as appropriate? Do the data meet the assumptions of the tests (e.g., normal distribution)? Describe any methods used to assess it. Is there an estimate of variation within each group of data? Is the variance similar between the groups that are being statistically compared?	Yes	M&M

Sample definition and in-laboratory replication	Information included in the manuscript?	In which section is the information available? (Reagents and Tools Table, Materials and Methods, Figures, Data Availability Section)
In the figure legends: state number of times the experiment was **replicated** in laboratory.	Yes	Figure legends
In the figure legends: define whether data describe **technical or biological replicates**.	Yes	Figure legends

Ethics

Ethics	Information included in the manuscript?	In which section is the information available? (Reagents and Tools Table, Materials and Methods, Figures, Data Availability Section)
Studies involving **human participants**: State details of **authority granting ethics approval** (IRB or equivalent committee(s), provide reference number for approval.	Yes	M&M
Studies involving **human participants**: Include a statement confirming that **informed consent** was obtained from all subjects and that the experiments conformed to the principles set out in the WMA Declaration of Helsinki and the Department of Health and Human Services Belmont Report.	Not Applicable	
Studies involving **human participants:** For publication of **patient photos**, include a statement confirming that consent to publish was obtained.	Not Applicable	
Studies involving experimental **animals**: State details of **authority granting ethics approval** (IRB or equivalent committee(s), provide reference number for approval. Include a statement of compliance with ethical regulations.	Yes	M&M
Studies involving **specimen and field samples:** State if relevant **permits** obtained, provide details of authority approving study; if none were required, explain why.	Not Applicable	

Dual Use Research of Concern (DURC)	Information included in the manuscript?	In which section is the information available? (Reagents and Tools Table, Materials and Methods, Figures, Data Availability Section)
Could your study fall under dual use research restrictions? Please check biosecurity documents and list of **select agents and toxins** (CDC): https://www.selectagents.gov/sat/list.htm	Not Applicable	
If you used a select agent, is the security level of the lab appropriate and reported in the manuscript?	Not Applicable	
If a study is subject to dual use research of concern regulations, is the name of the **authority granting approval and reference number** for the regulatory approval provided in the manuscript?	Not Applicable	

Reporting

The MDAR framework recommends adoption of discipline-specific guidelines, established and endorsed through community initiatives. Journals have their own policy about requiring specific guidelines and recommendations to complement MDAR.

Adherence to community standards	Information included in the manuscript?	In which section is the information available? (Reagents and Tools Table, Materials and Methods, Figures, Data Availability Section)
State if relevant guidelines or checklists (e.g., **ICMJE, MIBBI, ARRIVE, PRISMA**) have been followed or provided.	Not Applicable	
For **tumor marker prognostic studies,** we recommend that you follow the **REMARK** reporting guidelines (see link list at top right). See author guidelines, under 'Reporting Guidelines'. Please confirm you have followed these guidelines.	Not Applicable	
For **phase II and III randomized controlled trials**, please refer to the **CONSORT** flow diagram (see link list at top right) and submit the CONSORT checklist (see link list at top right) with your submission. See author guidelines, under 'Reporting Guidelines'. Please confirm you have submitted this list.	Not Applicable	

Data Availability

Data availability	Information included in the manuscript?	In which section is the information available? (Reagents and Tools Table, Materials and Methods, Figures, Data Availability Section)
Have **primary datasets** been deposited according to the journal's guidelines (see 'Data Deposition' section) and the respective accession numbers provided in the Data Availability Section?	Yes	Will be deposited immediately upon acceptance of manuscript
Were **human clinical and genomic datasets** deposited in a public access-controlled repository in accordance to ethical obligations to the patients and to the applicable consent agreement?	Yes	Study registered on ENA. Specific datesets will be deposited immediately upon acceptance of manuscript
Are **computational models** that are central and integral to a study available without restrictions in a machine-readable form? Were the relevant accession numbers or links provided?	Not Applicable	
If publicly available data were reused, provide the respective **data citations in the reference list**.	Not Applicable	
